# NRhFluors: Quantitative Revealing the Interaction between Protein Homeostasis and Mitochondria Dysfunction via Fluorescence Lifetime Imaging

**DOI:** 10.1021/acscentsci.3c01532

**Published:** 2024-03-21

**Authors:** Yubo Huang, Meiyi Chang, Xiaochen Gao, Jiabao Fang, Wenjing Ding, Jiachen Liu, Baoxing Shen, Xin Zhang

**Affiliations:** † School of Food Science and Pharmaceutical Engineering, Nanjing Normal University, 1 Wenyuan Road, Nanjing 210023, China; ‡ Department of Chemistry and Research Center for Industries of the Future, Westlake University, 600 Dunyu Road, Hangzhou 310030, Zhejiang China; § Westlake Laboratory of Life Sciences and Biomedicine, 18 Shilongshan Road, Hangzhou 310024, Zhejiang China

## Abstract

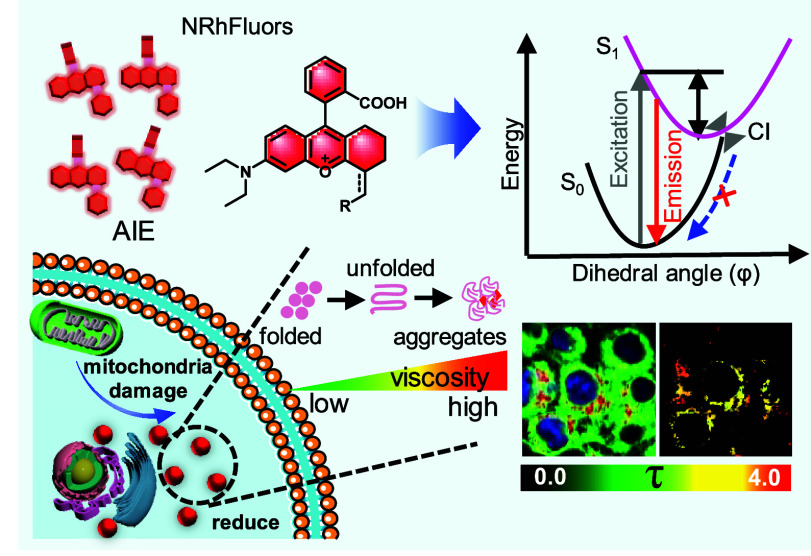

Degenerative diseases are closely related to the changes of protein conformation beyond the steady state. The development of feasible tools for quantitative detection of changes in the cellular environment is crucial for investigating the process of protein conformational variations. Here, we have developed a near-infrared AIE probe based on the rhodamine fluorophore, which exhibits dual responses of fluorescence intensity and lifetime to local viscosity changes. Notably, computational analysis reveals that NRhFluors fluorescence activation is due to inhibition of the RACI mechanism in viscous environment. In the chemical regulation of rhodamine fluorophores, we found that variations of electron density distribution can effectively regulate CI states and achieve fluorescence sensitivity of NRhFluors. In addition, combined with the AggTag method, the lifetime of probe A9-Halo exhibits a positive correlation with viscosity changes. This analytical capacity allows us to quantitatively monitor protein conformational changes using fluorescence lifetime imaging (FLIM) and demonstrate that mitochondrial dysfunction leads to reduced protein expression in HEK293 cells. In summary, this work developed a set of near-infrared AIE probes activated by the RACI mechanism, which can quantitatively detect cell viscosity and protein aggregation formation, providing a versatile tool for exploring disease-related biological processes and therapeutic approaches.

## Abstract 2

A series of near-infrared AIE probes activated by the RACI mechanism based on rhodamine fluorophores, which can quantitatively reveal viscosity changes in protein aggregate under mitochondrial damage.

## Introduction

Protein misfolding and aggregation are closely related to various diseases, including neurodegenerative diseases, cardiovascular diseases, and cancers, etc.^1−3^ In the past, investigations were mainly focused on the biophysical and biochemical properties of protein aggregation, especially the microenvironment such as changes in pH, viscosity or polarity.^4,5^ These previous studies provide abundant information about the structural morphology and protein aggregation mechanisms.^6−9^ However, the interaction between imbalance of protein homeostasis and organelles’ dysfunction is rarely investigated, in particular the intervention between proteins and mitochondria.^10,11^

During the process of protein misfolding and aggregation, the protein microenvironment viscosity increased and polarity decreased.^12,13^ These property alternations can be qualitatively and quantitatively studied through fluorescence analysis using sensitive probes to detect microenviormental changes. These probes are usually mounted on molucules with characteristics including fluorescent molecular rotors, solvation discoloration, aggregation induced emission (AIE),^14,15^ and they have been widely applied in biomolecular detection and bioimaging.^16,17^ For instance, AIE molecules had been utilized to traverse blood-brain barrier to monitor amyloid protein aggregation.^18,19^ Liu et al. also employed AIEgens to detect amorphous protein aggregates.^20^ However, probes for detecting protein aggregates in spatiotemporal resolution are rarely reported. In particular, there is a deficiency in methods for quantitative investigation of the connection between proteostasis imbalance and mitochondrial dysfunction.

**Figure 1:**
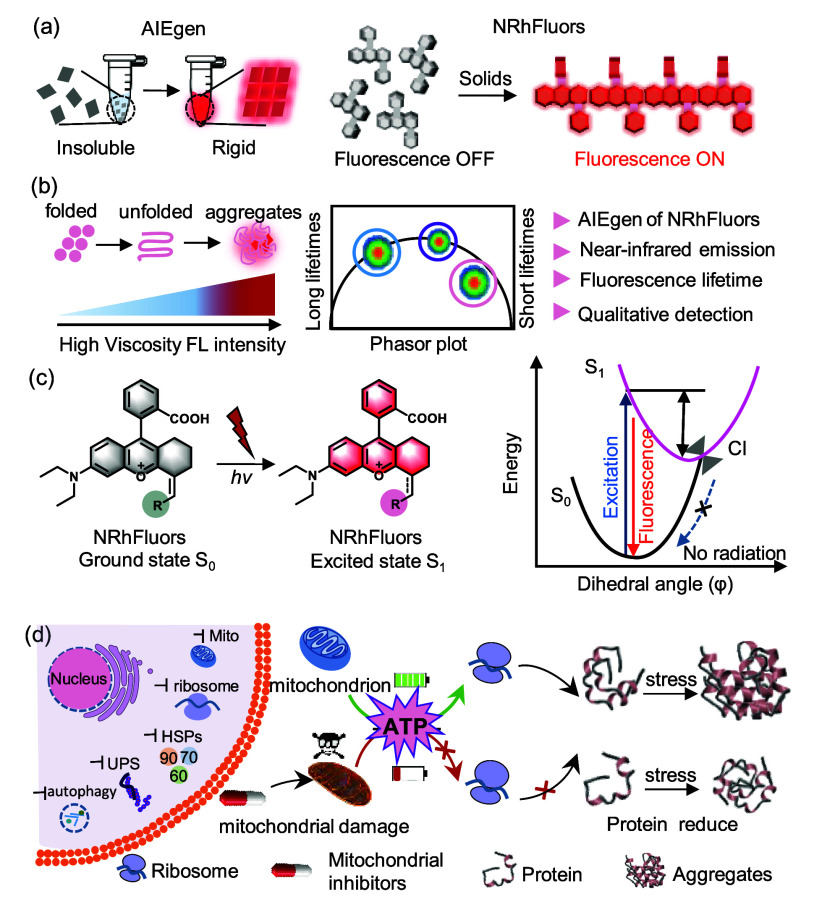
(a) NRhFluors exhibit AIE properties, expressing low fluorescence in dispersed environments and strong fluorescence in rigid environments. (b) Protein aggregation is a multistep process leading to changes in environmental viscosity. Near-infrared NRhFluors for qualitative and quantitative detection of protein aggregates through fluorescence intensity and lifetime detection. (c) The luminescence mechanism of NRhFluors based on RACI. Under excited state, the C=C bond exhibits properties of C–C bond, which undergoes nonradiative decay. (d) Mitochondria dysfunction leads to reduced protein expression.

Herein, we report a set of near-infrared (NIR) rhodamine-based AIE fluorophores, named NRhFluors (Figure 1a), that enables both quanlitaive and quantitative monitoring of local viscosity changes (Figure 1b). Spectacularly, computational analysis suggests that the AIE effect of NRhFluors is caused by the restricted access to a conical intersection (RACI) mechanism (Figure 1c). Further, we demonstrate that the electron density of electron density regulators (referred to as EDR) conjugated to rhodamine fluorophore exhibited a positive correlation with fluorescence intensity of NRhFluors, without affecting viscosity sensitivity. Based on these findings, we constructed a AggTag probe A9-Halo for investigation of local viscosity changes during aggregation of specific protein of interest (POI) using fluorescence lifetime imaging (FLIM). Lastly, we found that dysfunction of mitochondria reduced protein expression (Figure 1d). In summary, this work established a connection between proteostasis imbalance and mitochondrial dysfunction, providing insights for causes of aging and agingrelated diseases as well as theoretical guidance for disease treatment and prevention.

## Results and Discussion

### Rational Design and Exploration of Photophysical Properties of NRhFluors

**Figure 2:**
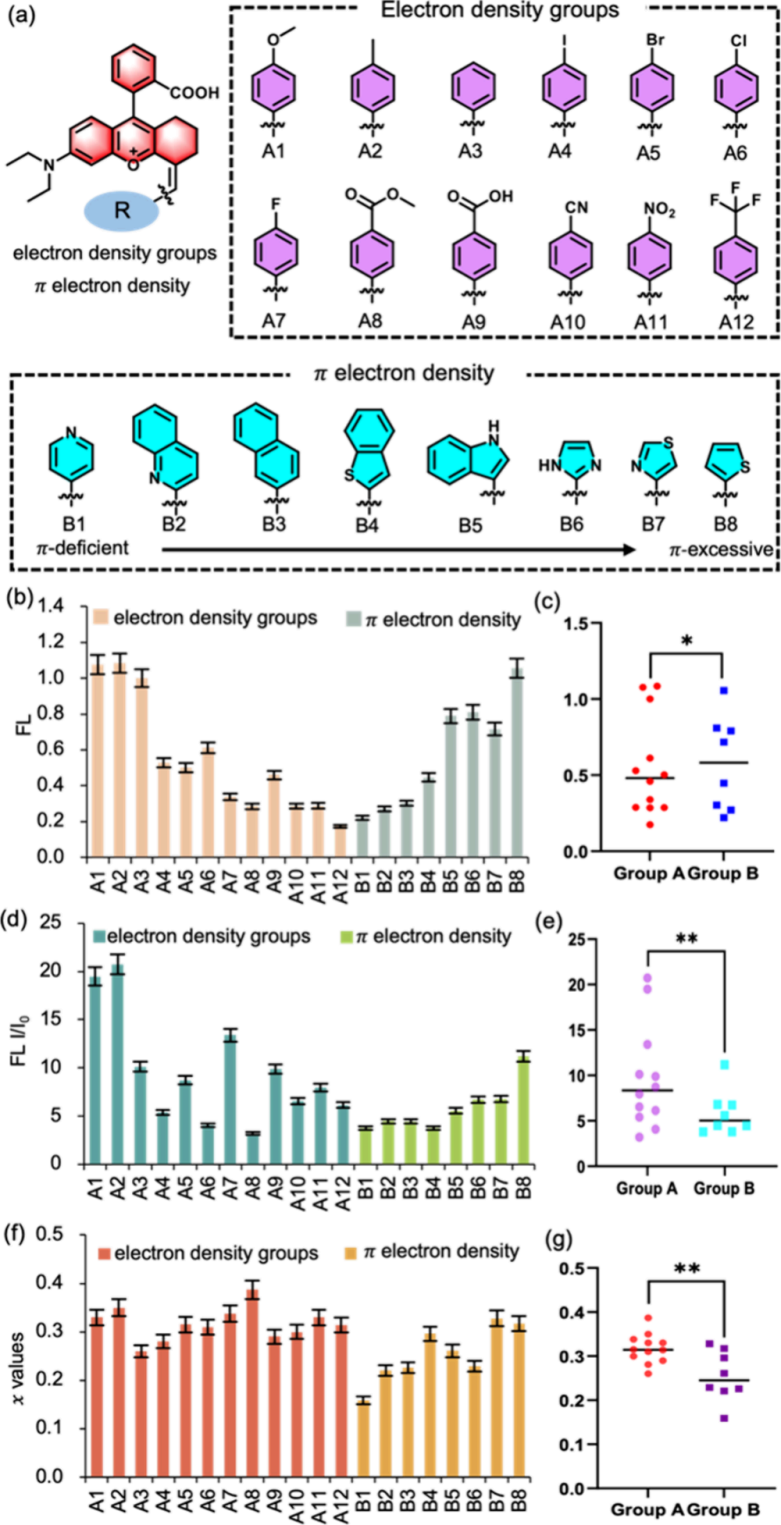
(a) Design of NRhFluors probes conjugating differenct EDRs. Group A is attributed to electron push–pull effect, while Group B regulates π-electron density. (b) Fluorescence intensity of NRhFluors probes conjugated with different EDR in 80% glycerol and 20% ethylene glycol. Values were normalized using the fluorescence intensity of A3. (c) The statistical significance of fluorescence intensity corresponding to (b). (d) Fluorescence intensity ratio (*F*_H_/*F*_T_) of NRhFluors probes conjugated with different EDR. *F*_T_ and *F*_H_ represent fluorescence intensity in 100% and 30% tetrahydrofuran solutions, respectively. (e) Electron push–pull effect had a significant impact on AIE properties of NRhFluors. (f) Viscosity sensitivity (*x* value) of NRhFluors probes conjugated with different EDR. (g) Statistical significance of NRhFluors viscosity sensitivity.

Inspired by abnormal protein microenvironment changes during aggregation formation, we aimed to sensitively monitor these viscosity changes through chemical signals transmissions. We synthesized two series of probes based on rhodamine scaffold for the purpose of obtaining adjustable physiological properties, specifically on fluorescence intensity in response to environmental viscosity changes. We first modulated the effect of electron donating ability of EDR on fluorescence activation by conjugating groups with varying electron donating or withdrawing capabilities to NRhFluors (Figure 2a, A1–A12, B1–B8). Collect spectral data of all compounds in 80% glycerol and 20% ethylene glycol (Table S1 and Figure S1–S21). To test whether these probes exhibit differences in fluorescence activation in viscous environment, we measured their fluorescence intensity in a mixture of ethylene glycol (20%) and glycerol (80%) (Figure 2b, 2c).

In addition, to evaluate the fluorescence response of the probe at different aggregation levels, we calculated the fold increase of fluorescence intensity (*F*_H_/*F*_T_) when water ratio in H_2_O-THF mixture increased from 0% (F_T_) to 70% (*F*_H_) (Figure 2d, 2e). We found that fluorescence intensity of probes (A1–A12) had a positive correlation with electron donating capacity of EDRs, as the relative fluorescence intensity of A1 was 5-fold higher than A12 (Figure S22–S25). Consistently, the *F*_H_/*F*_T_ value increased between 3.2- to 19.5-fold when electron donating capacity of EDR increased, indicating an enhancement in AIE property (Figure 2d, group A). Moreover, we also replaced EDR with heterocycles with varying π-electron densities and aromaticities (Figure 2a, group B). As predicted, a similar trend in relative fluorescence intensity and *F*_H_/*F*_T_ values were found in group B, as there was a 5-fold difference in fluorescence intensity between the most π deficient (B1) and π excessive heterocycles (B8) (Figure 2d, group B and S26–S29). Together, these results indicate that electron densities of EDR form extended π-conjugation that regulates the physiological properties of NRhFluors.

To test whether NRhFluors could detect environmental viscosity changes, we quantified fluorescence intensities of NRhFluors at varying viscosities using ethylene glycol and glycerol mixtures (Figure 2d and S30–S33). The slope of logarithmic fluorescence intensity as a function of logarithmic viscosity was determined as the viscosity sensitivity (*x*). We found that *x*-values of group A probes fluctuated around 0.3, whereas group B probes exhibited a positive correlation between π-electron density of EDRs and *x*-value (0.16 for B1 and 0.32 for B8). Collectively, modulating the electron density of EDR conjugated to rhodamine fluorophore allowed for the development of NRhFluors with regulatable physiological properties.

### Revealing AIE Activation Mechanism of NRhFluors

**Figure 3:**
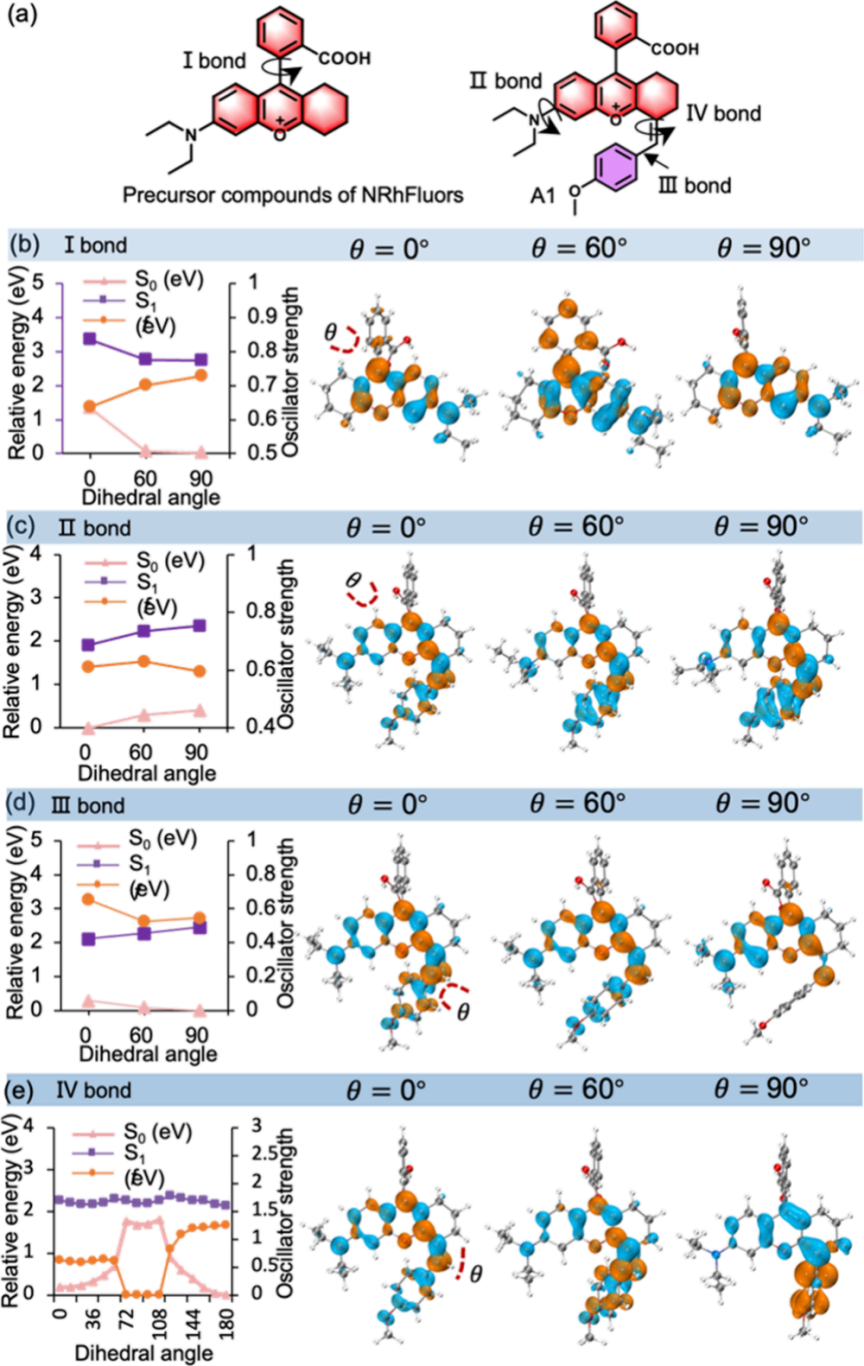
Exploring the fluorescence activating mechanism of NRhFluors using A1 as a model molecule. (a) Scheme of NRhFluors-A1 with indications of I–IV bond. (b–d) The relative energy (eV) and oscillator strength (*f*) of I, II and III bond. S_0_ and S_1_ energy from potential energy surface scanning, as well as electron–hole maps at three angles (0°, 60°, and 90°) were recorded. There was no significant change in S_1_ and S_0_ energy under 0–90° torsion, and the oscillator strength remains stable. (e) S_0_ and S_1_ energy from potential energy surface scanning, as well as electron–hole diagrams in multiple angles were recorded for IV bond. Around 90° torsion angle, S_0_ and S_1_ approached and the oscillator strength was almost zero, indicating that the excited state molecule was undergoing the RACI process.

Next, we conducted a detailed investigation based on density functional theory to inspect the AIE activation mechanism of NRhFluors. To inquire into the potential effect of specific chemical bond on the nature of AIE, we divided chemical bonds conjugated to rhodamine scaffold into four categories using NRhFluors-A1 as a model molecule (Figure 3a, I–IV bond). First, we calculated the potential energy distribution in ground state (S_0_) and excited state (S_1_) at three angles (0°, 60° and 90°) for I bond in the precursor compounds of NRhFluors (Figure 3b and Table S2). The activation energy of both ground state and excited state decreased to stabilization when I bond rotated from 0° to 90°. At the initial position (0°), the energy of ground state S_0_ (1.6 eV) and excited state S_1_ (3.5 eV) was in a highly disordered state, which could be de-excited by radiation conversion to obtain a stable state state at any time. This indicates that the ortho-carboxylbenzene guided by I bond prefers to be in a more stable 90° state and is difficult to undergo bond twist or rotation, as the rotational energy barrier (2.6 eV) is thermodynamically difficult to cross.

For II bond, the activation energy of both ground state and excited state increased when the dihedral angle increased from 0° to 90°. The energy of S_0_ increased to 0.35 eV from the initial infinitesimal value, while the energy of S_1_ increased by 2.2 eV (Figure 3c and Table S3). At 0° dihedral angle, there were a relatively stable ground state and excited state structure, indicating that twist of II bond poses a hindrance in maintaining stable molecular structure under excitation. Regardless of changes in the rotation angle, the oscillator strength remained relatively stable (*f* = 0.6), indicating that the molecule can be excited. No electron migration process was observed in the electron–hole diagram; thus, there was no fluorescence deactivation caused by TICT. The III bond exhibited the most stable ground and excited state energy, as they only showed an alternation of 0.3 and 0.28 eV respectively in response to bond rotation (Figure 3d). Although the electron–hole diagram of the conjugated structure guided by the III bond exhibited an appreciable electron transfer, the negligible energy difference and stable oscillator strength indicated that the electron transfer did not affect the fluorescence emission of the molecule.

**Figure 4:**
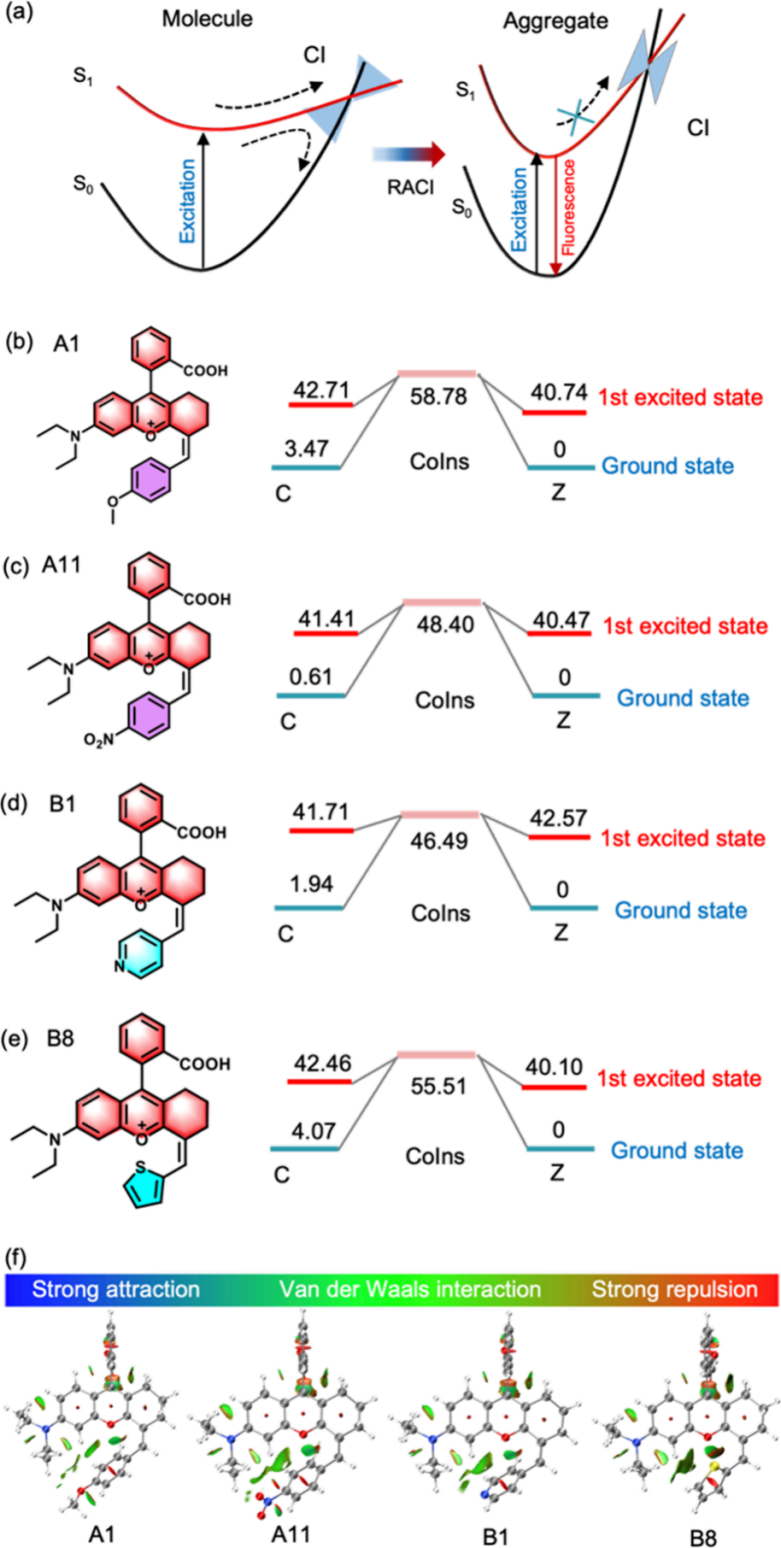
(a) Overview of RACI decay pathways possessed by AIEgens. The internal conversion process of the ground state and excited state of fluorescent molecules was illustrated. (b–e) Structures and distribution of CI energy for A1, A11, B1, and B8, respectively. (f) Intramolecular interactions between A1, A11, B1, and B8. Blue represents strong attraction, green represents weak interaction, and red represents strong repulsion.

However, we obtained surprising results in the potential energy surface scanning of the IV bond. We found that the excited state energy S_1_ remain relatively stable and the minimum energy point (2.18 eV) is located in-between 72° to 108°, while the oscillator strength in this range is close to 0 (Figure 3e). At 180°, the structure possesses the highest oscillator strength (*f* = 1.23), indicating its more capable of residing in S_1_ and activating fluorescence. On the other end, the relative energy of S_0_ reaches the peak (1.76 eV) around 90°, while the oscillator strength is close to 0. The electron–hole diagram in the excited state shows that the IV bond has an evident electron transfer within the 0–180° twist. The structure of the S_0_ and S_1_ states of the I–IV bond in A1 shows that only the IV bond exhibits significant dihedral angle changes (Figure S34–S37). Moreover, the Ball-and-stick model shows that the bond length of IV bond extends more at 90°, proving the effect of electron transfer on C=C bond (Figure S38–S41). Similar result was obtained for molecules A11, B1, and B8 (Figure S42 and Table S4–S6), excluding fortuity of the single molecule. Therefore, we speculated that the NRhFluors have a conical intersection (CI) of S_1_ and S_0_ between 72° to 108°, resulting in nonradiative de-excitation of the molecules (Figure 4a).

The spin-flip time-dependent density functional theory (SF-TDDFT) method provides an alternative to describe the internal conversion process between the ground state and the excited state entering the conical intersection. SF-TDDFT show that A1 cannot spontaneously reach the CI point through IV bond rotation with the ground-state structure, since the high energy barrier (55.3 kal/mol, Figure 4b) makes this process impractical to spontaneously carry out at room temperature. On the contrary, A11 exhibits a much lower rotational energy barrier (6.99 kal/mol, Figure 4c), which makes it easy to achieve nonradiative de-excitation through cis–trans isomerization conversion. At this point, the CI threshold is 48.4 kal/mol, indicating that the electron withdrawing A11 is easy to enter the CI state for internal conversion. This also explains the low fluorescence intensity of the conjugated electron withdrawing structure. In group B, the π-deficient B1 only needs 4.78 kal/mol to enter the CI state (Figure 4d), while the π-excessive B8 possesses a high rotational energy barrier of 13.05 kal/mol (Figure 4e), leading to CI blockage and strong fluorescence. Therefore, π excessive EDG can significantly enhance fluorescence emission. In addition, we excluded the possibility of strong covalent interactions existing between molecules using the reduced density gradient (RDG) method (Figure 4f, S43–S46). In summary, through DFT calculations we revealed that the AIE of NRhFluors molecules originates from the RACI mechanism led by the IV bond, rather than the widely known TICT effect. A RACI based fluorescence sensitivity regulation strategy of Rhodamine chromophore was proposed, and it was proved that reasonable design of electron cloud density distribution can effectively regulate the CI state to achieve the fluorescence sensitivity of such molecules.

### A9-Halo Quantifies Local Viscosity of Protein Aggregates Using Fluorescence Intensity and Lifetime *in Vitro*

**Figure 5:**
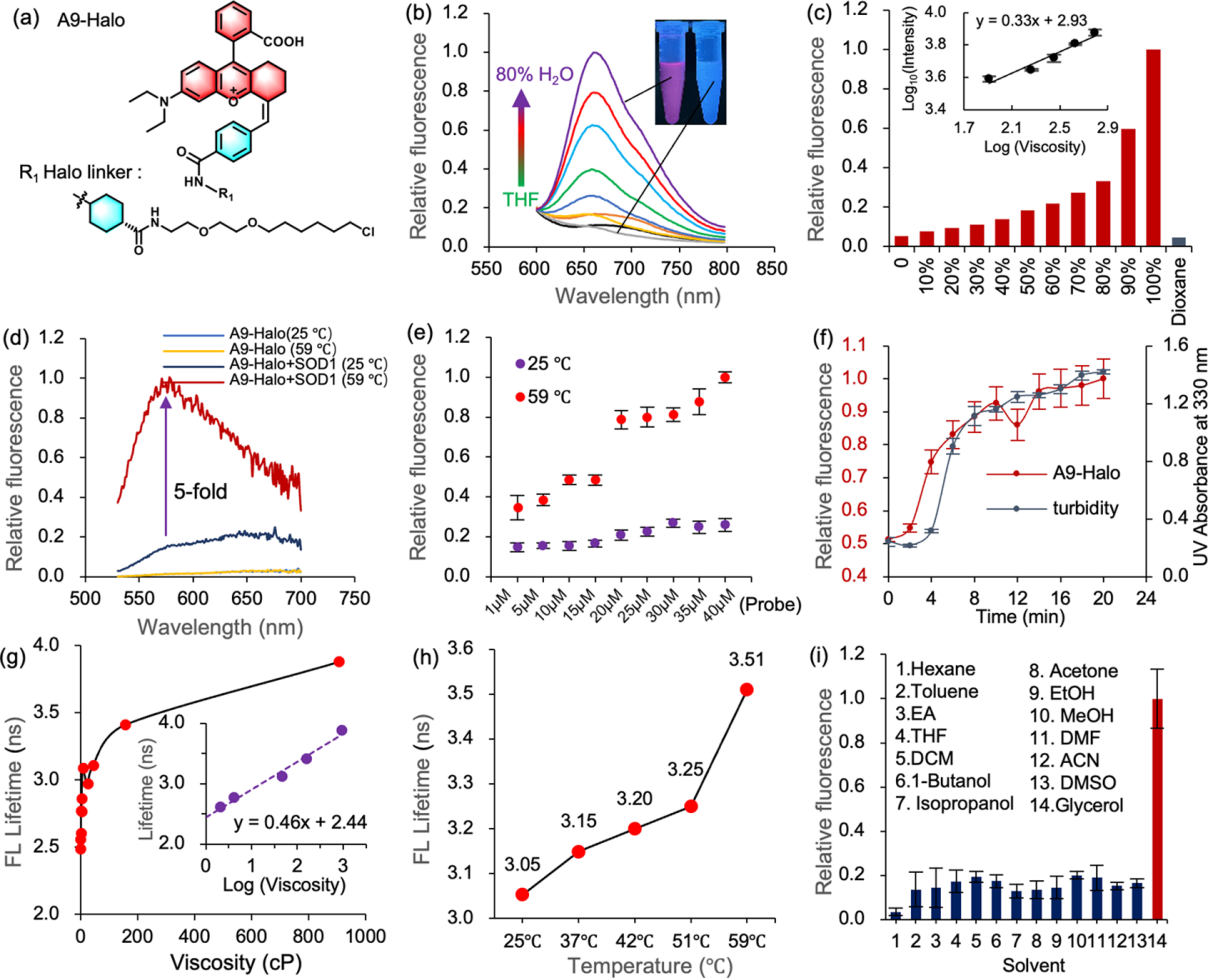
A9-Halo detects protein aggregation through dual response of both fluorescence intensity and lifetime. (a) The chemical structure of probe molecule A9-Halo. (b) Fluorescence response of A9-Halo measured in a mixed solvent of H_2_O and THF. Increase the proportion of H_2_O from 0% to 80%. (c) Fluorescence intensity values and viscosity sensitivity (*x* value) of A9-Halo under extensive viscosity changes in a mixture of ethylene glycol and glycerol. (d) Heat-induced aggregation of SOD1(A4 V)-Halo increased the fluorescence intensity of A9-Halo. The A9-Halo (20 μM) and protein (40 μM) were incubated at 59 °C for 10 min, while the control group was placed at room temperature of 25 °C. (e) Fluorescence intensity of SOD1(A4 V)-Halo aggregates (40 μM) in the presence of varying concentrations of A9-Halo (1, 5, 10, 15, 20, 25, 30, 35, 40 μM). SOD1(A4 V)-Halo aggregation was induced at 59 °C for 10 min in buffer (50 mM Tris-HCl, pH 7.5, 100 mM NaCl, 80 mM EDTA). (f) Fluorescence and turbidity kinetics during heat-induced SOD1(A4 V)-Halo aggregation at 59 °C. (g) Fluorescence lifetime of A9-Halo under a wide range of viscosity changes in a mixed system of H_2_O and glycerol. (h) The fluorescence lifetime changes of A9-Halo during heat-induced SOD1(A4 V)-Halo misfolding. (i) The fluorescence property of A9-Halo was not affected by polarity changes.

Guided by these findings, we chose the A9 molecule to demonstrate the ability of NRhFluor probes to detect the local viscosity changes upon protein aggregation. To this end, we anchored the Halo-linker warhead on A9 to obtain the AggTag probe A9-Halo (Figure 5a). The attached Halo-linker did not affect the AIE properties of A9, as demonstrated by the fluorescence response in a mixture of water and tetrahydrofuran (Figure 5b), and has good stability within the pH range of 4.5–8.5 (Figure S47). The *x* value of A9-Halo was determined as 0.33 (Figure 5c). Next, we evaluated whether A9-Halo could report on the aggregation of a POI through both fluorescence intensity and lifetime parameters. We chose the HaloTag fused superoxide dismutase 1 mutant A4 V (SOD1(A4 V)-Halo), whose aggregation is associated with amyotrophic lateral sclerosis (ALS) disease. Upon *in vitro* heating stress, we observed a 5-fold increase of fluorescence intensity when SOD1(A4 V)-Halo formed protein aggregates (Figure 5d), while the fluorescence intensity changes also exhibited a linear dependence on A9-Halo concentrations (Figure 5e and S48). Protein aggregation is a time-dependent multistep process, the specific aggregation state can be monitored through turbidity signals. Therefore, we monitored both the turbidity of SOD1(A4 V)-Halo and fluorescence signal of A9-Halo during protein misfolding. As a result, the fluorescence kinetics was fairly consistent with the turbidity assay response during SOD1(A4 V)-Halo misfolding, suggesting the fluorescence signal originated from insoluble aggregates (Figure 5f). Next, we further examined whether fluorescence lifetime of A9-Halo would respond to SOD1(A4 V)-Halo aggregation. As shown in Figure 5g, the fluorescence lifetime of A9-Halo exhibits a positive linearity with viscosity changes. When the SOD1(A4 V)-Halo·A9-Halo conjugate aggregated during heat stress, we observed evident fluoresence lifetime changes of A9-Halo (Figure 5h), from 3.05 ns (∼18 cP) at 25 °C to 3.51 ns (∼169 cP) at 59 °C. More importantly, local polarity was demonstrated to have negligible effect on A9-Halo fluorescence response (Figure 5i). Collectively, these result suggest that the A9-Halo is able to detect protein aggregation with both fluorescence intensity and lifetime.

### Detection and Visualization of Protein Aggregates *in Vivo*

**Figure 6:**
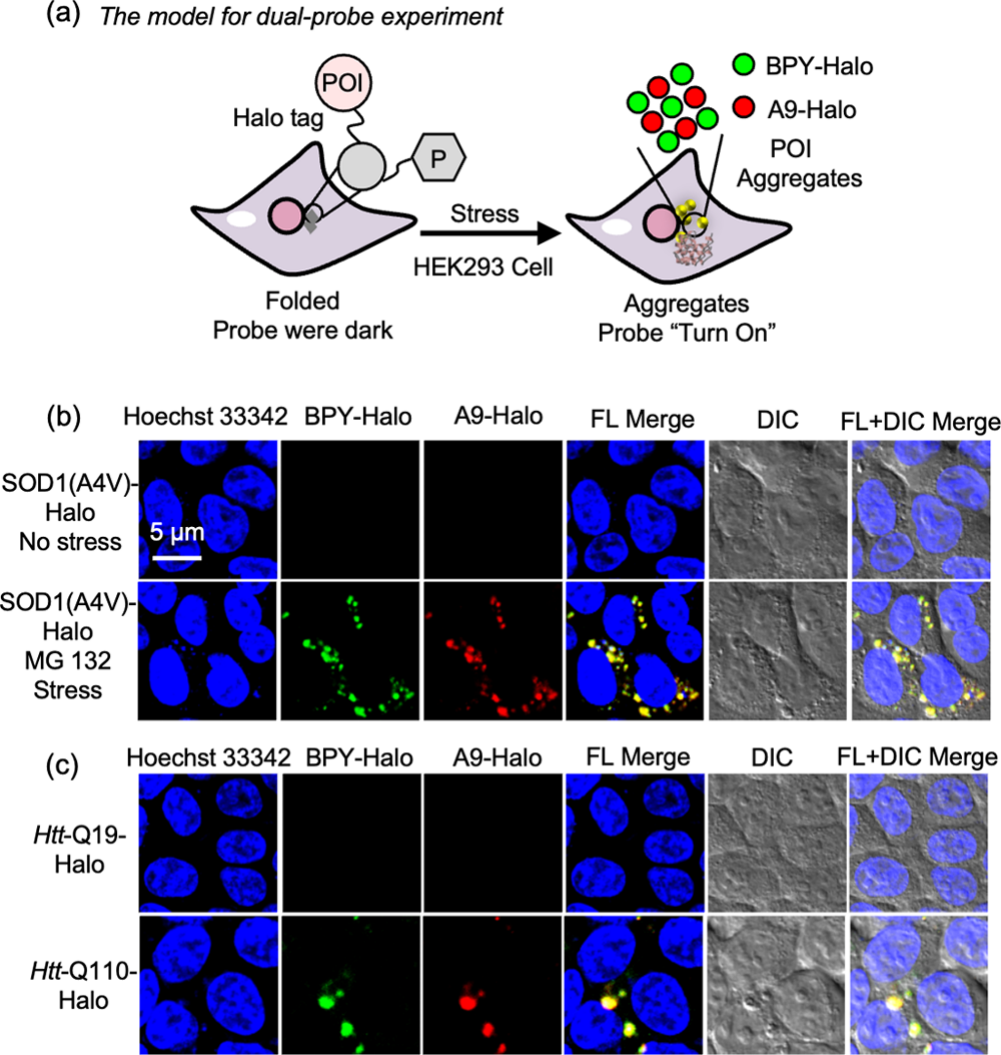
Colocalization imaging demonstrates the labeling performance of A9-Halo for protein aggregates in living cells. (a) A demonstration of the dual color imaging method using green channel and red channel probes working together, demonstrating the biocompatibility of A9-Halo probes as a tool for visualizing protein aggregates. (b) SOD1(A4 V)-Halo was transiently transfected and expressed in HEK293 cells, and 2 μM BPY-Halo and 2 μM A9-Halo were used as a costain together for 24 h. The control cell group without any intervention and MG132 (1 μM) were used to induce 24 h. (c) Images of *Htt*-Q19-Halo and *Htt*-Q110-Halo using A9-Halo and BPY-Halo. During the transfection of the *Htt*-Q19-Halo and *Htt*-Q110-Halo, HEK293 cells were also treated with 2 μM A9-Halo and BPY-Halo, then incubated for a further 48 h. Blue: Hoechst 33342, Green: BPY-Halo, Red: A9-Halo, Scale bar: 5 μm.

Given the impressive *in vitro* performance, we next investigated whether the developed A9-Halo could depict the viscosity changes during protein aggregation *in vivo*. In previous works, such properties have been examined in terms of the entire misfolded and aggregated proteins upon cellular stresses.^15,18,21,22^ However, the interaction between suborganelles and protein expression or aggregation has not yet been fully studied. In particular, there is a lack of research on mitochondria, which is the primary site for many biological processes including energy metabolism,^23−25^ cell signal transmission,^26−28^ protein synthesis^29−33^ and so on. The near-infrared probe has satisfactory sample penetration, high signal-to-noise ratio, high-quality resolution, and sensitive response. These features make it highly favored in the field of biological imaging.^34−38^ Using the A9-Halo (NIR, λ_ex_= 561 nm) and a previously reported AggTag probe BPY-Halo (Green, λ_ex_= 510 nm),^39^ we aimed to provide a dual-probe imaging strategy to quantitatively detect local viscosity changes of protein aggregation associated with mitochondria dysfunction (Figure 6a).

To this end, we costained cells expressing SOD1(A4 V)-Halo with A9-Halo (2 μM) and BPY-Halo (2 μM). Upon treatment of the protease inhibitor MG132 (1 μM) for 24 h, we observed fluorescence activation from both BPY-Halo and A9-Halo at protein aggregates around the nucleus (Figure 6b and S49a). To validate that A9-Halo can be widely utilized to detect protein aggregation, we also expressed Huntingtin exon 1 (*Htt*) in cells. With expansion of a polyglutamine tract within its N-terminal domain (*Htt*-polyQ), *Htt* is well-known for its spontaneous protein aggregation ability and association with Huntington’s disease.^40,41^*Htt*-polyQ with repeats higher than Q78 would form insoluble protein aggregates in the perinuclear region.^42−44^ When cells expressing *Htt*-Q110-Halo were costained with A9-Halo (2 μM) and BPY-Halo (2 μM), we observed punctate fluorescence signal from both A9-Halo and BPY-Halo (Figure 6c, bottom panel). In contrast, there was no fluorescence in cells expressing *Htt*-Q19-Halo, since it only formed soluble oligomers (Figure 6c, top panel and S49b). This observation is consistent with *in vitro* result and previous reports, suggesting that A9-Halo can be applied to detect protein aggregates in live cells.

**Figure 7:**
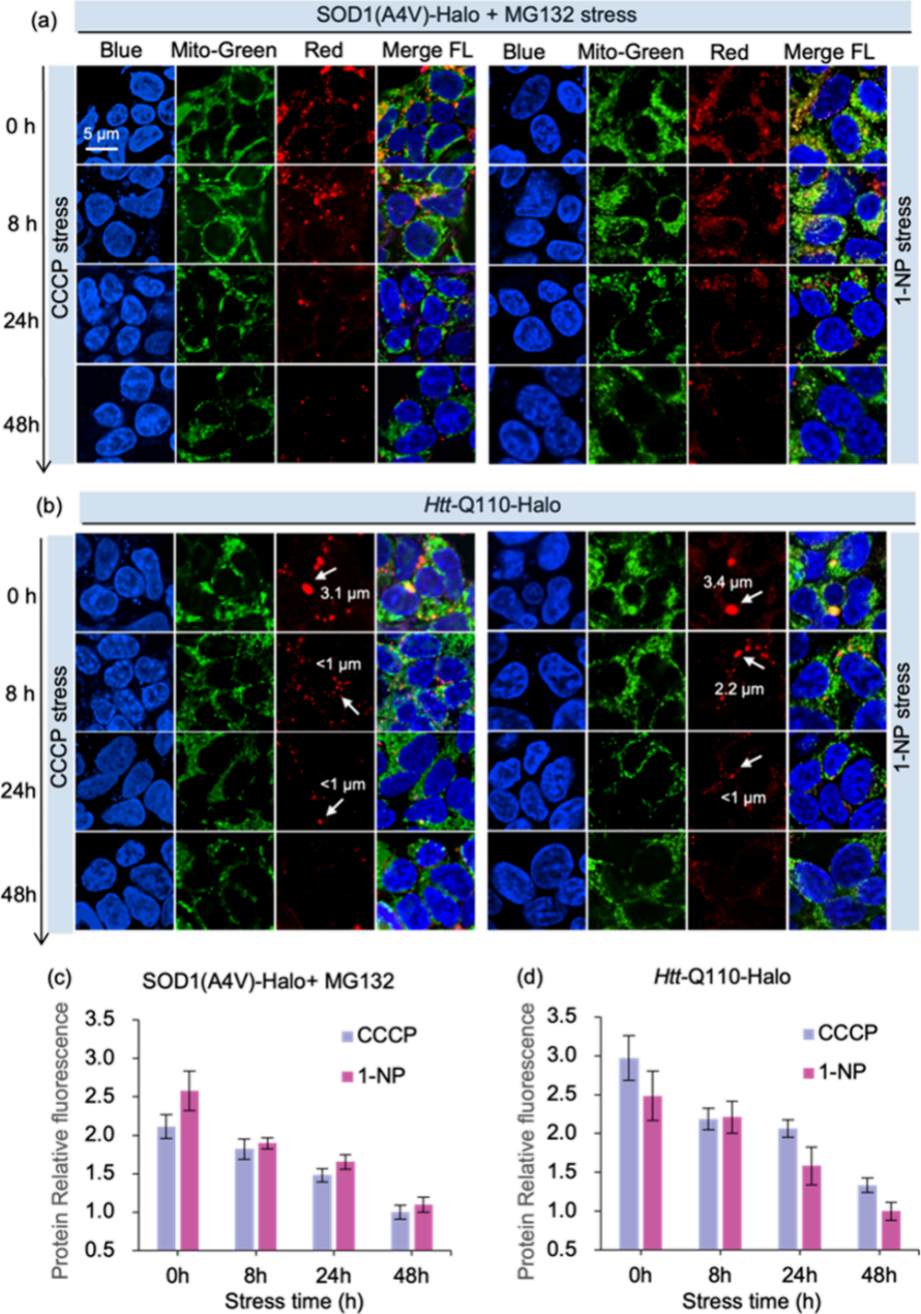
Status of mitochondria is closely related to the level of protein expression. (a) HEK293 cells were treated with mitochondrial damaging agents (CCCP or 1-NP) for 8, 24, and 48 h respectively, followed by induction of transfection and complete expression of SOD1(A4 V)-Halo for 24 h. Then add MG132 to induce protein aggregation for 24 h. (b) HEK293 cells that treated with CCCP or 1-NP (1 μM) for 8, 24, and 48 h were cultured, and subsequently overexpressed *Htt*-Q110-Halo and stained with commercial mitochondrial labeling reagent (Mito-Tracker Green, 2 μM) and A9-Halo (2 μM). (c) Extraction of relative fluorescence intensity of SOD1(A4 V)-Halo aggregates.(d) Extraction of relative fluorescence intensity of *Htt*-Q110-Halo aggregates. Blue: Hoechst 33342, Green: Mito green, Red: A9-Halo, Scale bar: 5 μm.

The excellent imaging capability allowed us to explore how mitochondrial damage intervened in protein expression and aggregate viscosity. Mitochondrial damage will activate the mitochondrial apoptosis program, which greatly limits the normal physiological function including protein synthesis. We herein used commercially available time-dependent mitochondrial damage agent 1-Nitropyrene (1-NP) and Carbonyl cyanide 3-chlorophenylhydrazone (CCCP) to induce mitochondrial apoptosis, atempting to explore the effect of mitochondria dysfuntion on protein expression.^45,46^ To test whether mitochondrial dysfunction leads to decreased protein expression, we treated HEK293 cells with CCCP and 1-NP (1 μM) for 8, 24, and 48 h, respectively, before expressed SOD1(A4 V)-Halo and costaining with commercial mitochondrial labeling reagent (MitoTracker Green, 2 μM) and A9-Halo (2 μM). After treatment with MG132 (1 μM) for 24 h, a decreased fluorescent signal from A9-Halo was observed comparing to cells without treatment, suggesting there was a reduced level of protein expression (Figure 7a). SDS-page electrophoresis analysis of cell lysates showed that mitochondrial dysfunction led to a decrease in protein expression (Figure S50). In addition, the labeling capacity of MitoTracker Green decreased, as reflected by increasingly diffused green fluorescence. In cells treated with 1 μM CCCP or 1-NP for 24 h, the fluorescent intensity of A9-Halo was significantly reduced, and large aggresomes were absent. The dispersed fluorescence signal of MitoTracker Green indicated a poor labeling efficiency due to the further mitochondrial damage. Especially after 48 h of drug action, almost no fluorescence signals from protein aggregates were observed. The weakening of green and red channel signals indicates a close correlation between the production of protein aggregates and mitochondrial homeostasis changes (Figure 7a, 48 h). Interestingly, restricting mitochondrial function after full expression of SOD1 and *Htt* proteins, the drug-treated cells exhibited a low level of fluorescence that was close to that of the negative control (Figure S51). Therefore, we hypothesized that the correlation between mitochondrial dysfunction and the degree of aggregation of proteins may not be significant under conditions that ensure adequate protein expression.

Subsequently, we examined our probe system with Huntingtin exon 1 protein. When cells expressing *Htt*-Q110-Halo were costained with MitoTracker Green (2 μM) and A9-Halo (2 μM), we observed both green signal from mitochondria and red fluorescence of A9-Halo in punctate structures. Interestingly, the *Htt*-Q110-Halo puncta showed an average diameter of 3.4 ± 0.1 μm, measured via A9-Halo fluorescence signal (Figure 7b, 0 h). The cells were pretreated with CCCP (1 μM) or 1-NP (1 μM) for 8 h before expressing *Htt*-Q110-Halo, we observed that the size of aggregates labeled by A9-Halo decreased with time (the aggregates less than 1 um after CCCP treatment, 2.2 μm treated with 1-NP), compared to that without treatment (Figure 7b, 8 h). After pretreatment was extended to 24 and 48 h, the fluorescence intensity of A9-Halo was significantly decreased and the large aggregates became absent (Figure 7b, 24 and 48 h). Subsequently, we extracted the fluorescence density function of aggregates labeled by A9-Halo in the cell images of different drug treatment times during their formation and maturation over a period of 48 h. We observed that the fluorence intensity of the aggregates decreased with time owing to mitochondrial damage degree. For instance, we found the average fluorescence intensity of SOD1(A4 V)-Halo aggregates after being treated with drugs for 48 h exhibited a 2.5 fold regular reduction (Figure 7c), indicating mitochondrial dysregulation reduced protein expression. Further, the average fluorence intensity of *Htt*-Q110-Halo aggregates after being treated with drugs for 48 h showed a 3-fold gradual reduction (Figure 7d). Collectively, these results suggest that destabilization of mitochondria is correlative with the process of protein expression.

### FLIM Quantitative Monitoring of Protein Aggregates in Living Cells

**Figure 8:**
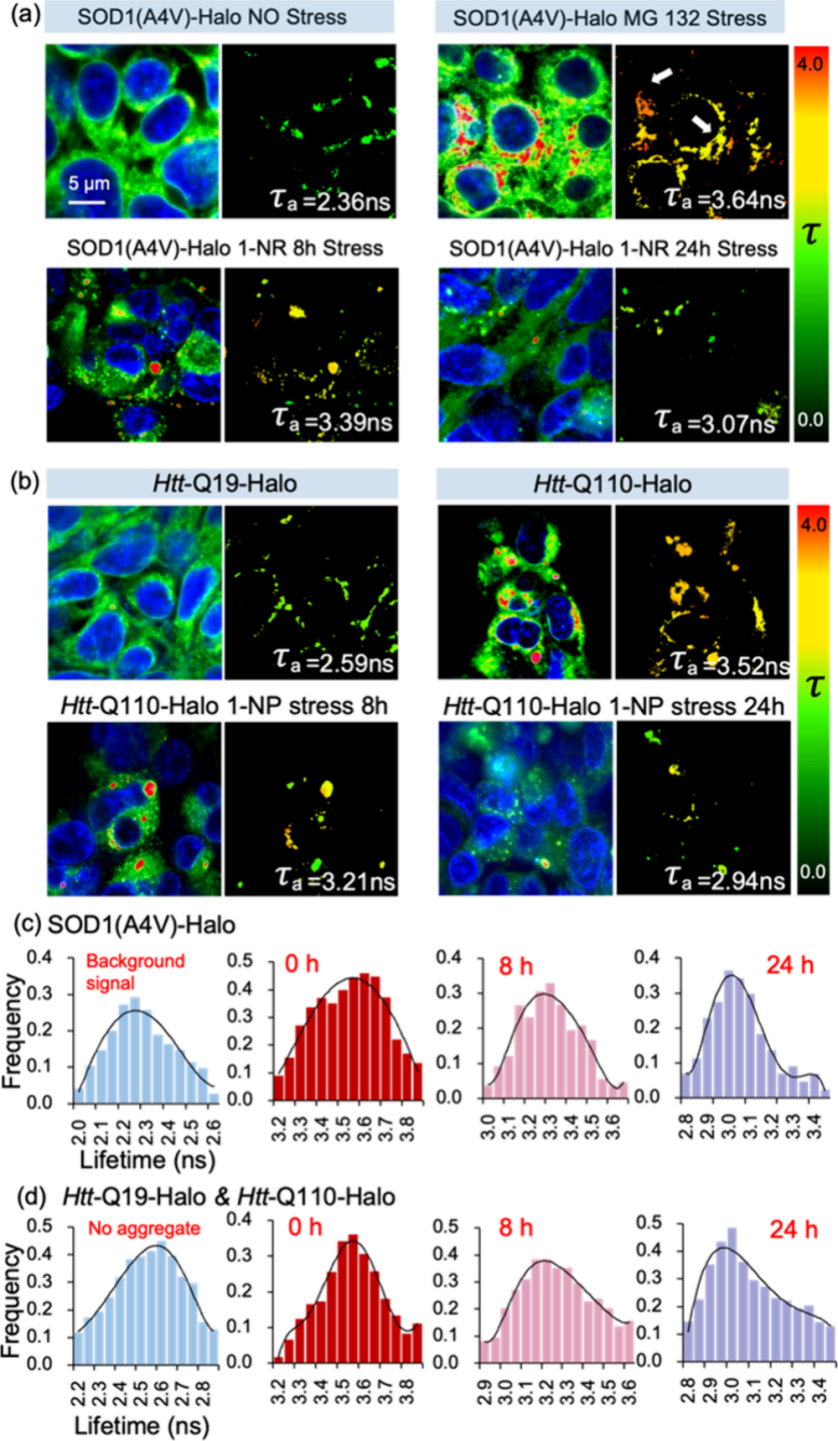
A9-Halo probe can quantitatively measure the viscosity changes of various protein aggregates through fluorescence lifetime. (a) Lifetime imaging of SOD1(A4 V)-Halo aggregates induced by MG132. The viscosity changes of SOD1(A4 V)-Halo protein under different degrees of mitochondrial damage were observed. Using 1-NP (1 μM) treatment of cells for 8 and 24 h induced varying degrees of mitochondrial damage before protein transfection. Subsequently, SOD1(A4 V)- Halo was transfected and fully expressed for 24 h. Finally, MG132 was used to induce protein aggregation. (b) *Htt*-Q19- Halo and *Htt*-Q110-Halo were expressed in HEK293 cells, respectively. The *Htt*-Q19-Halo without aggregates only exhibits background fluorescence lifetime signals, while the *Htt*-Q110-Halo with self-aggregation can observe fluorescence lifetime signals from A9-Halo in aggregates. Cells were treated with 1-NP for 8 and 24 h, respectively, and then transfected with *Htt*-Q110 Halo to provide sufficient expression time. After 24 h of 1-NP treatment, protein aggregate lifetime imaging showed low lifetime signals. (c) The distribution of intracellular background fluorescence signals and lifespan signals of SOD1 (A4 V)-Halo protein aggregates under varying degrees of mitochondrial damage. (d) The distribution of intracellular fluorescence signals and lifespan signals of *Htt*-Q110-Halo protein aggregates under different degrees of mitochondrial damage expressing *Htt*-Q19-Halo. Note: All vertical coordinates represent the frequency of lifetime distribution, while the horizontal axis represents lifetime (ns). Scale bar: 5 μm.

Lastly, we utilized A9-Halo to dissect the viscosity changes of protein aggregates in live cells using FLIM. In cells expressing SOD(A4 V)-Halo without any further treatment, we found that the average lifetime of A9-Halo was 2.36 ns (viscosity of ∼2 cP, Figure 8a and S52a) and a broad range of fluorescence signal was exhibited. When cells expressing SOD1(A4 V)-Halo were treated with MG132 (1 μM) for 24 h, we observed both diffusive and punctate fluorescent signals. Here, the average lifetime of A9-Halo was 3.64 ns, and the local viscosity of the misfolded SOD(A4 V)-Halo was determined to be 316 cP (Figure 8a and S52b). In contrast, when cells were first treated with 1-NP for 8 h, before conducting the same experiment, we observed a diffusive fluorescence and a decreased lifetime of 3.39 ns (viscosity of ∼95 cP, Figure 8a and S52c). This suggested that misfolded SOD(A4 V)-Halo existed in a low viscosity and loosely packed conformation. When the pretreatment of 1-NP was extended to 24 h before expressing SOD1(A4 V)-Halo and stressing with MG132, we observed a reduction of misfolded SOD1(A4 V)-Halo, while the fluorescence lifetime was 3.07 ns (viscosity of ∼20 cP, Figure 8a and S52d). The decreased lifetime (3.64 to 3.07 ns) and local viscosity (316 to 20 cP) of misfolded SOD1(A4 V)-Halo suggested that mitochondrial damage would reduce protein expression. The above lifetime changes are further determined based on the fitted lifetime distribution frequency. Without MG132 stress, only intracellular background lifespan signals were collected and concentrated in the short term range of 2.2–2.4 ns in SOD1. The aggregate lifetime distribution of normal cells without mitochondrial damage ranges from 3.5–3.7 ns. With the stress of 1-NP 8 h lifetime distribution range is wider (3.2–3.5 ns), and there is only a small amount of lifetime distribution in the 3.2–3.5 ns range after 24 h of damage (frequency <0.1, Figure 8c). The change in viscosity corresponding to different lifetimes is reflected in Figure S54a.

In addition to the cells analyzed in Figure 8a, we observed similar lifetime changing tendencies of *Htt*-Q110-Halo punctuates in other cells, supporting the general inhibitory effect of mitochondrial dysfunction on protein expression. We expressed *Htt*-Q110-Halo in HEK293 cells and observed insoluble perinuclear aggregates, which exhibited a higher local viscosity than that of *Htt*-Q19-Halo (diffusive fluorescence), reflected by the fluorescence lifetime differences of 3.52 ns (viscosity of ∼178 cP) and 2.59 ns (viscosity of ∼2 cP), respectively (Figure 8b, S53a and S53b). To further demonstrate that mitochondrial damage would reduce protein expression, cells were pretreated with 1-NP for 8h, before expressing *Htt*-Q110-Halo. The quantity and average diameters of *Htt*-Q110-Halo aggregates were smaller compared to aggresomes in cells without the 1-NP treatment. The corresponding lifetime was calculated as 3.21 ns (∼40 cP). Moreover, if 1-NP treatment was extended to 24 h, the lifetime of *Htt*-Q110-Halo was reduced even further (2.94 ns, viscosity of ∼11 cP) and large aggregates were absent (Figure 8b, S53c and S53d). Compared to SOD1, the *Htt* protein still maintains a wide range of lifespan distribution within 24 h of drug action (3.1–3.5 ns, Figure 8d). A wide range of viscosity changes in *Htt*-proteins have also been recorded (Figure S54b). It is likely related to the unique self-aggregation of *Htt* protein, reducing dependence on mitochondria. These results are consistent with the intensity imaging, suggesting that induction of reasonable level of mitochondrial dysfunction may be a feasible method to prevent diseases associated with protein aggregates. Hence, this work provides a potential direction and a fluorogenic toolbox for future development of therapeutic methods and specific drugs.

## Conclusion

In summary, we designed a series of near-infrared AIE probes based on Rhodamine fluorophore for quantitative detection of local viscosity changes. The fluorescence activation of these probes in viscous environment are resulted from restriction of the RACI mechanism. Importantly, when reporting on local viscosity changes, both the fluorescence intensity and lifetime of probe A9-Halo exhibit a positive linear dependence on viscosity changes. Thus, this property allows us to use fluorescence intensity and FLIM imaging to detect protein aggregation in living cells, in particular enabling quantitative monitoring the viscosity changes in aggregate. Moreover, we showed that reasonable level of mitochondrial damage could reduce protein expression. Finally, the fluorogenic toolbox presented in this work is a promising platform for exploring mechanism of diseases related to protein denaturation.

## Supporting Information Available

Detailed experimental procedures, supplementary tables, supplementary figures, synthetic methods, and NMR and MS characterizations are provided in Supporting Information. The main content includes plasmids construction, protein expression and purification, absorbance and fluorescence spectra measurement, viscosity dependence measurement and calculation, density functional theory (DFT) computational experiment, in vitro experiments using purified proteins, live cell imaging, general synthetic and chromatographic methods, etc (PDF)

## References

[ref1] Hartl F. U.; Hayer-Hartl M. Protein folding - Molecular chaperones in the cytosol: from nascent chain to folded protein. Science 2002, 295 (5561), 1852 10.1126/science.1068408. 11884745

[ref2] Kaushik S.; Cuervo A. M. Proteostasis and aging. Nature Medicine 2015, 21 (12), 1406 10.1038/nm.4001. 26646497

[ref3] Linse S.; Linse B. Protein folding through kinetic discrimination. J. Am. Chem. Soc. 2007, 129 (27), 8481 10.1021/ja070386e. 17564448

[ref4] Fu L.; Ma G.; Yan E. C. Y. In Situ Misfolding of Human Islet Amyloid Polypeptide at Interfaces Probed by Vibrational Sum Frequency Generation. J. Am. Chem. Soc. 2010, 132 (15), 5405 10.1021/ja909546b. 20337445

[ref5] Shahnawaz M.; Mukherjee A.; Pritzkow S.; Mendez N.; Rabadia P.; Liu X.; Hu B.; Schmeichel A.; Singer W.; Wu G.; et al. Discriminating alpha-synuclein strains in Parkinson’s disease and multiple system atrophy. Nature 2020, 578 (7794), 273 10.1038/s41586-020-1984-7. 32025029 PMC7066875

[ref6] Barz B.; Liao Q.; Strodel B. Pathways of Amyloid-beta Aggregation Depend on Oligomer Shape. J. Am. Chem. Soc. 2018, 140 (1), 319 10.1021/jacs.7b10343. 29235346

[ref7] Liu L.; Zhang L.; Niu L.; Xu M.; Mao X.; Yang Y.; Wang C. Observation of Reduced Cytotoxicity of Aggregated Amyloidogenic Peptides with Chaperone-like Molecules. ACS Nano 2011, 5 (7), 6001 10.1021/nn201773x. 21682328

[ref8] Sant’Anna R.; Gallego P.; Robinson L. Z.; Pereira-Henriques A.; Ferreira N.; Pinheiro F.; Esperante S.; Pallares I.; Huertas O.; Rosario Almeida M. Repositioning tolcapone as a potent inhibitor of transthyretin amyloidogenesis and associated cellular toxicity. Nat. Commun. 2016, 7, 10787 10.1038/ncomms10787. 26902880 PMC4766415

[ref9] Zhu L.; Song Y.; Cheng P.-N.; Moore J. S. Molecular Design for Dual Modulation Effect of Amyloid Protein Aggregation. J. Am. Chem. Soc. 2015, 137 (25), 8062 10.1021/jacs.5b01651. 26043045

[ref10] Sebastian R. M.; Shoulders M. D. In Annual Review of Biochemistry, Vol 89; Kornberg R. D., Ed., 2020; Vol. 89. 10.1146/annurev-biochem-013118-111552 PMC7311290 32097570

[ref11] Chen X.; Ji B.; Hao X.; Li X.; Eisele F.; Nystrom T.; Petranovic D. FMN reduces Amyloid-beta toxicity in yeast by regulating redox status and cellular metabolism. Nat. Commun. 2020, 11 (1), 867 10.1038/s41467-020-14525-4. 32054832 PMC7018843

[ref12] Owyong T. C.; Subedi P.; Deng J.; Hinde E.; Paxman J. J.; White J. M.; Chen W.; Heras B.; Wong W. W. H.; Hong Y. A Molecular Chameleon for Mapping Subcellular Polarity in an Unfolded Proteome Environment. Angew. Chem., Int. Ed. 2020, 59 (25), 10129 10.1002/anie.201914263. 31826303

[ref13] Zhang S.; Liu M.; Tan L. Y. F.; Hong Q.; Pow Z. L.; Owyong T. C.; Ding S.; Wong W. W. H.; Hong Y. A Maleimide-functionalized Tetraphenylethene for Measuring and Imaging Unfolded Proteins in Cells. Chemistry-an Asian Journal 2019, 14 (6), 904 10.1002/asia.201900150. 30768765

[ref14] Bai Y.; Huang Y.; Wan W.; Jin W.; Shen D.; Lyu H.; Zeng L.; Liu Y. Derivatizing merocyanine dyes to balance their polarity and viscosity sensitivities for protein aggregation detection. Chem. Commun. 2021, 57 (98), 13313 10.1039/D1CC05200D. 34812440

[ref15] Ye S.; Hsiung C.-H.; Tang Y.; Zhang X. Visualizing the Multistep Process of Protein Aggregation in Live Cells. Acc. Chem. Res. 2022, 55 (3), 381 10.1021/acs.accounts.1c00648. 35040316 PMC9098262

[ref16] Hu F.; Xu S.; Liu B. Photosensitizers with Aggregation-Induced Emission: Materials and Biomedical Applications. Adv. Mater. 2018, 30 (45), 1801350 10.1002/adma.201801350. 30066341

[ref17] Qian J.; Tang B. Z. AIE Luminogens for Bioimaging and Theranostics: from Organelles to Animals. Chem. 2017, 3 (1), 56 10.1016/j.chempr.2017.05.010.

[ref18] Fu W.; Yan C.; Guo Z.; Zhang J.; Zhang H.; Tian H.; Zhu W.-H. Rational Design of Near-Infrared Aggregation-Induced-Emission Active Probes: In Situ Mapping of Annyloid-beta Plaques with Ultrasensitivity and High-Fidelity. J. Am. Chem. Soc. 2019, 141 (7), 3171 10.1021/jacs.8b12820. 30632737

[ref19] Huang J.; He B.; Zhang Z.; Li Y.; Kang M.; Wang Y.; Li K.; Wang D.; Tang B. Z. Aggregation-Induced Emission Luminogens Married to 2D Black Phosphorus Nanosheets for Highly Efficient Multimodal Theranostics. Adv. Mater. 2020, 32 (37), 2003382 10.1002/adma.202003382. 32761671

[ref20] Shen D.; Jin W.; Bai Y.; Huang Y.; Lyu H.; Zeng L.; Wang M.; Tang Y.; Wan W.; Dong X.; et al. Rational Design of Crystallization-Induced-Emission Probes To Detect Amorphous Protein Aggregation in Live Cells. Angew. Chem., Int. Ed. 2021, 60 (29), 16067 10.1002/anie.202103674. 33991044

[ref21] Klymchenko A. S. Solvatochromic and Fluorogenic Dyes as Environment-Sensitive Probes: Design and Biological Applications. Acc. Chem. Res. 2017, 50 (2), 366 10.1021/acs.accounts.6b00517. 28067047

[ref22] Bai Y.; Wan W.; Huang Y.; Jin W.; Lyu H.; Xia Q.; Dong X.; Gao Z.; Liu Y. Quantitative interrogation of protein co-aggregation using multi-color fluorogenic protein aggregation sensors. Chemical Science 2021, 12 (24), 8468 10.1039/D1SC01122G. 34221329 PMC8221170

[ref23] Liesa M.; Shirihai O. S. Mitochondrial Networking in T Cell Memory. Cell 2016, 166 (1), 9 10.1016/j.cell.2016.06.035. 27368094

[ref24] Lowell B. B.; Spiegelman B. M. Towards a molecular understanding of adaptive thermogenesis. Nature 2000, 404 (6778), 652 10.1038/35007527. 10766252

[ref25] Ozcan U. Mitofusins: Mighty Regulators of Metabolism. Cell 2013, 155 (1), 17 10.1016/j.cell.2013.09.013. 24074856

[ref26] Kang J.-S.; Tian J.-H.; Pan P.-Y.; Zald P.; Li C.; Deng C.; Sheng Z.-H. Docking of axonal mitochondria by syntaphilin controls their mobility and affects short-term facilitation. Cell 2008, 132 (1), 137 10.1016/j.cell.2007.11.024. 18191227 PMC2259239

[ref27] Latorre-Pellicer A.; Moreno-Loshuertos R.; Victoria Lechuga-Vieco A.; Sanchez-Cabo F.; Torroja C.; Acin-Perez R.; Calvo E.; Aix E.; Gonzalez-Guerra A.; Logan A.; et al. Mitochondrial and nuclear DNA matching shapes metabolism and healthy ageing. Nature 2016, 535 (7613), 561 10.1038/nature18618. 27383793

[ref28] Lum J. J.; Bauer D. E.; Kong M.; Harris M. H.; Li C.; Lindsten T.; Thompson C. B. Growth factor regulation of autophagy and cell survival in the absence of apoptosis. Cell 2005, 120 (2), 237 10.1016/j.cell.2004.11.046. 15680329

[ref29] Cioni J.-M.; Lin J. Q.; Holtermann A. V.; Koppers M.; Jakobs M. A. H.; Azizi A.; Turner-Bridger B.; Shigeoka T.; Franze K.; Harris W. A.; et al. Late Endosomes Act as mRNA Translation Platforms and Sustain Mitochondria in Axons. Cell 2019, 176 (1–2), 56 10.1016/j.cell.2018.11.030. 30612743 PMC6333918

[ref30] Itoh Y.; Andrell J.; Choi A.; Richter U.; Maiti P.; Best R. B.; Barrientos A.; Battersby B. J.; Amunts A. Mechanism of membrane-tethered mitochondrial protein synthesis. Science 2021, 371 (6531), 846 10.1126/science.abe0763. 33602856 PMC7610362

[ref31] Lewis S. C.; Uchiyama L. F.; Nunnari J. ER-mitochondria contacts couple mtDNA synthesis with mitochondrial division in human cells. Science 2016, 353 (6296), aaf5549 10.1126/science.aaf5549. 27418514 PMC5554545

[ref32] Meinecke M.; Wagner R.; Kovermann P.; Guiard B.; Mick D. U.; Hutu D. P.; Voos W.; Truscott K. N.; Chacinska A.; Pfanner N.; et al. Tim50 maintains the permeability barrier of the mitochondrial inner membrane. Science 2006, 312 (5779), 1523 10.1126/science.1127628. 16763150

[ref33] Saurer M.; Leibundgut M.; Nadimpalli H. P.; Scaiola A.; Schonhut T.; Lee R. G.; Siira S. J.; Rackham O.; Dreos R.; Lenarcic T.; et al. Molecular basis of translation termination at noncanonical stop codons in human mitochondria. Science 2023, 380 (6644), 531 10.1126/science.adf9890. 37141370

[ref34] Filonov G. S.; Krumholz A.; Xia J.; Yao J.; Wang L. V.; Verkhusha V. V. Deep-Tissue Photoacoustic Tomography of a Genetically Encoded Near-Infrared Fluorescent Probe. Angew. Chem., Int. Ed. 2012, 51 (6), 1448 10.1002/anie.201107026. PMC3293502 22213541

[ref35] Gong Q.; Zhang X.; Li W.; Guo X.; Wu Q.; Yu C.; Jiao L.; Xiao Y.; Hao E. Long-Wavelength Photoconvertible Dimeric BODIPYs for Super-Resolution Single-Molecule Localization Imaging in Near-Infrared Emission. J. Am. Chem. Soc. 2022, 144 (48), 21992 10.1021/jacs.2c08947. 36414278

[ref36] Liu K.; Kong X.; Ma Y.; Lin W. Rational Design of a Robust Fluorescent Probe for the Detection of Endogenous Carbon Monoxide in Living Zebrafish Embryos and Mouse Tissue. Angew. Chem., Int. Ed. 2017, 56 (43), 13489 10.1002/anie.201707518. 28851036

[ref37] Lu H.; Zheng Y.; Zhao X.; Wang L.; Ma S.; Han X.; Xu B.; Tian W.; Gao H. Highly Efficient Far Red/Near-Infrared Solid Fluorophores: Aggregation-Induced Emission, Intramolecular Charge Transfer, Twisted Molecular Conformation, and Bioimaging Applications. Angew. Chem., Int. Ed. 2016, 55 (1), 155 10.1002/anie.201507031. 26576818

[ref38] Xu Z.; Huang X.; Han X.; Wu D.; Zhang B.; Tan Y.; Cao M.; Liu S. H.; Yin J.; Yoon J. A Visible and Near-Infrared, Dual-Channel Fluorescence-On Probe for Selectively Tracking Mitochondrial Glutathione. Chem. 2018, 4 (7), 1609 10.1016/j.chempr.2018.04.003.

[ref39] Shen B.; Jung K. H.; Ye S.; Hoelzel C. A.; Wolstenholme C. H.; Huang H.; Liu Y.; Zhang X. A dual-functional BODIPY-based molecular rotor probe reveals different viscosity of protein aggregates in live cells. Aggregate 2023, 4 (3), e301 10.1002/agt2.301.

[ref40] Baeuerlein F. J. B.; Saha I.; Mishra A.; Kalemanov M.; Martinez-Sanchez A.; Klein R.; Dudanova I.; Hipp M. S.; Hartl F. U.; Baumeister W.; et al. In Situ Architecture and Cellular Interactions of PolyQ Inclusions. Cell 2017, 171 (1), 179 10.1016/j.cell.2017.08.009. 28890085

[ref41] Lecerf J. M.; Shirley T. L.; Zhu Q.; Kazantsev A.; Amersdorfer P.; Housman D. E.; Messer A.; Huston J. S. Human single-chain Fv intrabodies counteract in situ huntingtin aggregation in cellular models of Huntington’s disease. Proc. Natl. Acad. Sci. U.S.A. 2001, 98 (8), 4764 10.1073/pnas.071058398. 11296304 PMC31908

[ref42] Kim Y. E.; Hosp F.; Frottin F.; Ge H.; Mann M.; Hayer-Hartl M.; Hartl F. U. Soluble Oligomers of PolyQ-Expanded Huntingtin Target a Multiplicity of Key Cellular Factors. Mol. Cell 2016, 63 (6), 951 10.1016/j.molcel.2016.07.022. 27570076

[ref43] Krobitsch S.; Lindquist S. Aggregation of huntingtin in yeast varies with the length of the polyglutamine expansion and the expression of chaperone proteins. Proc. Natl. Acad. Sci. U.S.A. 2000, 97 (4), 1589 10.1073/pnas.97.4.1589. 10677504 PMC26479

[ref44] Monsellier E.; Redeker V.; Ruiz-Arlandis G.; Bousset L.; Melki R. Molecular Interaction between the Chaperone Hsc70 and the N-terminal Flank of Huntingtin Exon 1 Modulates Aggregation. J. Biol. Chem. 2015, 290 (5), 2560 10.1074/jbc.M114.603332. 25505179 PMC4317008

[ref45] Yu X.; Meng F.; Huang J.; Li W.; Zhang J.; Yin S.; Zhang L.; Wang S. 1-Nitropyrene exposure induces mitochondria dysfunction and impairs oocyte maturation in mice. Ecotoxicology and Environmental Safety 2022, 242, 113921 10.1016/j.ecoenv.2022.113921. 35908531

[ref46] Zhang W.-w.; Li X.-l.; Liu Y.-l.; Liu J.-y.; Zhu X.-x.; Li J.; Zhao L.-l.; Zhang C.; Wang H.; Xu D.-x.; Gao L. 1-Nitropyrene disrupts testosterone biogenesis via AKAP1 degradation promoted mitochondrial fission in mouse Leydig cell. Environ. Pollut. 2022, 307, 119484 10.1016/j.envpol.2022.119484. 35613681

