# Supplementary material for: NRhFluors: Quantitative Revealing the Interaction between Protein Homeostasis and Mitochondria Dysfunction via Fluorescence Lifetime Imaging
Source: ACS Cent Sci. 2024 Mar 21;10(4):842–51. doi: 10.1021/acscentsci.3c01532 (PMC11046461; doi:10.1021/acscentsci.3c01532)
Supplement: Supplementary file 1 — oc3c01532_si_001.pdf [file oc3c01532_si_001.pdf]

Supporting Information

# NRhFluors: Quantitative Revealing the Interaction between Protein Homeostasis and Mitochondria Dysfunction via Fluorescence Lifetime Imaging

Yubo Huang [‡, a], Meiyi Chang [‡, a], Xiaochen Gao [a], Jiabao Fang [a], Wenjing Ding [a], Jiachen Liu [a], Baoxing Shen [*, a], and Xin Zhang [*, b]

[a] School of Food Science and Pharmaceutical Engineering, Nanjing Normal University, 1 Wenyuan Road, Nanjing 210023, China

[b] Department of Chemistry, Research Center for Industries of the Future, Westlake University, 600 Dunyu Road, Hangzhou 310030, Zhejiang, China.; Westlake Laboratory of Life Sciences and Biomedicine, 18 Shilongshan Road, Hangzhou 310024, Zhejiang, China.

[‡] These authors contributed equally.

Corresponding authors E-mail: shenbx@njnu.edu.cn; zhangxin@westlake.edu.cn

**ABSTRACT:** Degenerative diseases are closely related to the changes of protein conformation beyond the steady state. The development of feasible tools for quantitative detection of changes in cellular environment is crucial for investigating the process of protein conformational variations. Here, we have developed a near-infrared AIE probe based on the rhodamine fluorophore, which exhibits dual responses of fluorescence intensity and lifetime to local viscosity changes. Notably, computational analysis reveal that NRhFluors fluorescence activation is due to inhibition of the RACI mechanism in viscous environment. In the chemical regulation of rhodamine fluorophores, We found that variations of electron density distribution can effectively regulate CI states and achieve fluorescence sensitivity of NRhFluors. In addition, combined with the AggTag method, the lifetime of probe A9-Halo exhibit a positive correlation with viscosity changes. This analytical capacity allows us to quantitatively monitor protein conformational changes using fluorescence lifetime imaging (FLIM) and demonstrate that mitochondrial dysfunction lead to reduced protein expression in HEK293 cells. In summary, this work developed a set of near-infrared AIE probes activated by the RACI mechanism, which can quantitatively detect cell viscosity and protein aggregation formation, providing a versatile tool for exploring disease-related biological processes and therapeutic approaches.

## Table of Contents


1. **Experimental Procedures**
    - 1.1. Plasmids
    - 1.2. Protein expression and purification
    - 1.3. Absorbance spectra and fluorescence spectra measurement
    - 1.4. Viscosity dependence measurement and calculation
    - 1.5. Computational calculation
    - 1.6. Imaging: Confocal
    - 1.7. Proteomic confocal imaging under sustained chemical-induced mitochondrial damage
    - 1.8. Lifetime Imaging
    - 1.9. In vitro experiments
2. **Supplementary Tables**
3. **Supplementary Figures**
    - 3.1. Absorbance Spectra, Fluorescence Spectra
    - 3.2. Aggregation induced luminescence effect in water and tetrahydrofuran systems
    - 3.3. Viscosity sensitivity
    - 3.4. Theoretical calculation of $S_0$ and $S_1$ state.
    - 3.5. pH dependence of A9-Halo
    - 3.6. In vitro Protein Aggregation Assays
    - 3.7. A9-Halo and BPY-Halo visualizes protein aggregates via fluorogenic signals in live cells.
    - 3.8. Fluorescence lifetime imaging
4. **Synthetic Methods**
5. **HRMS and H/C NMR spectra**
6. **References**

## 1. Experimental methods

### 1.1 Plasmids

***Mammalian expression***: The SOD-1 gene was amplified from the pF146 pSOD1WTAcGFP1, respectively. pHTN vector (Promega, Inc) with a stop codon added to the C-terminal of Halo-Tag. By QuickChange PCR, the SOD1(A4V) mutations were created. The *Htt*-Q110 and *Htt*-Q19 genes were sub-cloned into a pHTC HaloTag CMV-neo vector by the PIPE cloning method.

***Protein expression***: pET29b vectors were constructed to encode Halo-Tag, Snap-Tag, SOD1(A4V)-Halo. A4V mutations were constructed from the SOD1(WT) using the QuickChange PCR.

### *1.2 Protein expression and purification*

Halo Tag, SOD1(A4V)-Halo: Plasmids were transformed into E. coli BL21DE3* competent cells harboring a pBAD vector encoding σ32-I54N. Expression and purification was carried out as previously described. In brief, cells expressing recombinant proteins were thawed and lysed by sonication at 4 ºC in buffer A (50 mM Tris-HCl, pH 7.5, 100 mM NaCl) with addition of a protease inhibitor (1 mM PMSF). Lysed cells were centrifuged at 13000 rpm for 60 min at 4 ºC. The supernatant was collected and loaded onto a 6 mL BioRad Nuvia Ni-IMAC column and washed with buffer A. The protein was then eluted by gradient addition of buffer B containing 50 mM Tris • HCl (pH 7.5), 100 mM NaCl, and 500 mM imidazole. The protein fractions were identified by SDS-PAGE analysis, pooled, and concentrated. After that, the gel filtration column (120 mL HiPrepTM 16/60 SephacrylTM S200 HR) was used to allow further protein purification and buffer exchange (50 mM Tris-HCl, pH 7.5, 100 mM NaCl). No significant impurities were identified and purity was estimated to be > 98% based on SDS-PAGE.

### *1.3 Absorbance Spectra, Fluorescence Spectra*

Spectral measurements were obtained from a 20 μM probe solution in the mixture of 80% glycerol and 20% ethylene glycol. After thoroughly mixing the glycerol and the probe, 200 μL of the sample was transferred to a quartz spectrophotometer cell, and the absorption spectrum was recorded by Cary 5000 UV-Vis Spectrophotometer. While 200 μL of the sample was transferred to a fluorescent quartz cuvette, and the excitation and emission spectra were recorded using HITACHI F-7100 fluorescence spectrophotometer.

### *1.4 Viscosity Sensitivity Measurements*

All probes were prepared as 20 μM in a series of ethylene glycol/ glycerol (EG/G) solution in the following mixing ratios: EG/G = 70/30 (81cP), 50/50 (183 cP), 40/60 (283 cP), 30/70 (426 cP), 20/80 (621 cP). All samples were mixed thoroughly before transferring to 96-well plates. First, the excitation and emission spectra of all probes and their corresponding maximum wavelengths were measured in 80% glycerol system. The fluorescence intensities of all probes in EG/G were then measured at the maximum emission wavelength. Three independent measurements were conducted to produce an average value for each data. Logarithm plot of emission intensity as a function of viscosity was used to determine the value

of viscosity sensitivity $(x)$ based on the Förster-Hoffmann equation $logI = xlog\eta + C$, wherein η is viscosity (cP), I is the fluorescence intensity, χ is the viscosity sensitivity.

***1.5 Computational calculation***

We conducted detailed investigation based on density functional theory to inspect the AIE activation mechanism of NRhFluors. To inquire into the potential effect of specific chemical bond on the nature of AIE, we divided chemical bonds conjugated to rhodamine scaffold into four categories using NRhFluors-A1 as a model molecule (I-IV bond). The RACI (Restriction of access to conical intersection) process was studied by SF-TDDFT (Spin-flip time-dependent density functional theory) method. All SF-TDDFT calculations were performed with the ORCA quantum chemistry software (Version 5.0.4) using the PBE0 functional and the def2-SVP basis set. Grimme's D3BJ dispersion correction was used to improve calculation accuracy. The CPCM (Conductor-like Continuum Polarization Model) implicit solvation model was used to account for the solvation effect. The SMD implicit solvation model was used to account for the solvation effect.

The SMD implicit solvation model [1] was used to account for the solvation effect. Hole-electron analysis is performed by Multiwfn package [2].

***1.6 Imaging: Confocal***

The HEK293 cells were seeded at 25% confluency 24 hours prior to transfection in poly-D-lysine coated 20 mm glass bottom dishes. Cells were grown in DMEM media supplemented with 10% FBS and Penicillin-Streptomycin antibiotics until they reached 50-60% confluency. Transfection was carried out using X-tremeGene™ 9 DNA transfection reagent (Roche) according to the manufacturer’s instructions. Proteins were expressed for 24 hours prior to analyses. To label proteins with Halo-Tag fusion, protein expression was carried out in the presence of probe (BPY-Halo, A9-Halo) to form covalent conjugate with the Halo-Tag domain. Cells were stained with 5 μM Hoechst 33342 nuclear dye and incubated at 37 °C for 30 min. After incubation, excess probes and unbound Halo ligands were washed off with PBS and DMEM. Finally, add 2 mL DMEM and stabilize in a 37 °C incubator for 30min before confocal imaging. Confocal images were obtained using Nikon Ti-E-A1R confocal microscope. A9-Halo were visualized using the red laser (561 nm). BPY-Halo were visualized using the green (488 nm) laser. Hoechst 33342 was visualized using the blue laser (403 nm). For all imaging experiments, laser intensities, gain and other settings were kept identical to exclude artifacts and ensure robustness of data.

***1.7 Proteomic confocal imaging under sustained chemical-induced mitochondrial damage***

The HEK293 cultures were seeded at 25% confluency 24 hours prior to transfection in 20 mm glass bottom culture dishes. Cells were grown in DMEM medium supplemented with 10% FBS and penicillin-streptomycin antibiotics until they reached 50-60% confluency. To test whether mitochondrial dysfunction leads to decreased protein expression, we treated HEK293 cells with CCCP and 1-NP (1 μM) for 8h, 24h and 48h. respectively, before expressed SOD1(A4V)-Halo and co-staining with commercial

mitochondrial labelling reagent (MitoTracker Green, 2 μM) and A9-Halo (2 μM). Transfection was carried out using X-tremeGene™ 9 DNA transfection reagent (Roche) according to the manufacturer's instructions. During the transfection of the POI, 2 μM probe and 2 μM MitoTracker Green were used simultaneously to act on HEK293 cells to fully express the protein for 24 hours. Then, use 1 μM MG132 to induce protein aggregation for 24 hours. It is noting that all *Htt* proteins do not require MG132 treatment. Cells were stained with 5 μM Hoechst 33342 nuclear dye and incubated at 37°C for 30 min. After incubation, excess probes were washed off with PBS and DMEM. Finally, add 2 mL DMEM and stabilize in a 37 °C incubator for 30 min before confocal imaging.

***1.8 Lifetime Imaging***

The cell culture process is consistent with cell confocal imaging (part of 1.7). Fluorescent lifetime signals are recorded by VistaVision FastFLIM.

***1.9 In vitro experiments***

Fluorescence of probes are insensitive to pH. To determine whether fluorescence of probes studied in this work is sensitive to pH, we measured fluorescence excitation and emission spectra in solvents with different pH values. The pH value of glycerol solution in our measurement is ~ 6.0, therefore a 90% glycerol and 10% water mixture resulted in a pH 6.0 solution. The acidic solution was produced by a mixture of 90% glycerol and 300 mM NaOAc•HCl at pH 4.5. The basic solution was produced by a mixture of 90% glycerol and 100 mM Tris•HCl at pH 8.5. The neutral solution at pH 7.5 was produced by a mixture of 90% glycerol in PBS buffer. Thus, these solutions collectively give us the measurement of pH values at 4.5, 6.0, 7.5, and 8.5. All probes were insensitive to pH values and exhibited almost identical excitation and emission spectra.

Fluorescence response of A9-Halo measured in a mixed solvent of water and tetrahydrofuran. Record the fluorescence intensity of A9-Halo in a wide range of water and tetrahydrofuran mixed solvents.

Fluorescence response of probe in $H_2O$ with increasing concentrations of glycerol. Probe (20 μM) were prepared in glycerol: $H_2O$ mixture with increasing glycerol concentrations. All readings were normalized against the fluorescence intensity in 100% glycerol as 1.

Heat induced purified SOD1-A4V conjugated with probe. The temperature gradient from 25 ºC to 59 ºC (25, 37, 38.9, 41.8, 45.6, 50.7, 54.5, 57.2 and 59 ºC) was utilized in this experiment to induce protein misfolding and aggregation. Each sample contains of protein and probe for conjugation (the concentration is determined by experiment). The concentration of EDTA was 80 mM for chelation of metal ions in SOD1 protein. Each sample was incubated for 10 minutes under different temperatures. The fluorescence intensity was recorded using the corresponding excitation wavelength. All the fluorescence intensity was normalized. The fluorescence of protein conjugate was also visualized by UV transilluminator. Fluorescent lifetime signals are recorded by VistaVision FastFLIM.

Heat induced (25 ºC and 59 ºC) purified SOD1-A4V conjugated with A9-Halo. The probe (20 μM) and protein (40 μM) were incubated at 59 ºC for 10 minutes, and the control group was placed at room temperature of 25 ºC. Buffer condition: 50 mM Tris-HCl, pH 7.5, 100 mM NaCl, 83 mM EDTA. The testing condition: A9-Halo (Ex = 549 nm).

Aggregation Turbidity Assays. SOD1(A4V)-Halo: Aggregation was carried out with buffer containing indicated concentrations of EDTA that was used to chelate the structural metal ion of SOD1. Aggregation solution contained 40 μM SOD1(A4V)-Halo and 20 μM of probe was added to the solution at the beginning of reaction. Reaction was carried out quiescently at 54.5 ºC.

Simulate a wide range of viscosity changes using different proportions of water and glycerol. And measure the A9-Halo fluorescence lifetime of multi-viscosity.

The fluorescence property of A9-Halo in a wide range of dielectric constants. We compared the fluorescence emission in 13 solvents with different dielectric constants and glycerol systems, include: hexane, toluene, EA, THF, DCM, 1-Butanol, isopropanol, acetone, EtOH, MeOH, DMF, ACN, DMSO.

## 2. Supplementary Tables

**Table S1:** Photophysical properties of NRhFluors.

| Compounds | $\lambda_{abs}$ [a] (nm) | $\lambda_{ex}$ [b] (nm) | $\lambda_{em}$ [c] (nm) | Stoke's Shift [d] (nm) | Viscosity Sensitivity [e] ($\chi$) |
|---|---|---|---|---|---|
| A1 | 572 | 564 | 652 | 80 | 0.211 |
| A2 | 553 | 543 | 649 | 96 | 0.232 |
| A3 | 545 | 548 | 645 | 100 | 0.272 |
| A4 | 544 | 543 | 652 | 108 | 0.257 |
| A5 | 544 | 522 | 656 | 112 | 0.322 |
| A6 | 548 | 546 | 653 | 105 | 0.332 |
| A7 | 542 | 542 | 652 | 110 | 0.257 |
| A8 | 544 | 517 | 654 | 110 | 0.317 |
| A9 | 542 | 545 | 655 | 113 | 0.263 |
| A10 | 542 | 544 | 665 | 123 | 0.299 |
| A11 | 541 | 544 | 670 | 129 | 0.332 |
| A12 | 539 | 535 | 652 | 113 | 0.318 |
| B1 | 537 | 536 | 651 | 114 | 0.159 |
| B2 | 546 | 536 | 655 | 109 | 0.221 |
| B3 | 551 | 553 | 650 | 99 | 0.226 |
| B4 | 564 | 573 | 664 | 100 | 0.296 |
| B5 | 649 | 634 | 689 | 40 | 0.261 |
| B6 | 558 | 562 | 655 | 97 | 0.229 |
| B7 | 545 | 547 | 636 | 91 | 0.328 |
| B8 | 564 | 575 | 660 | 96 | 0.317 |
| A9-Halo | 549 | 549 | 656 | 107 | 0.331 |

[a] The maximum absorbance wavelength. [b] The maximum emission wavelength. [c] The maximum emission wavelength. [d] Stokes shift was calculated using $\Delta\lambda = \lambda_{em} - \lambda_{abs}$. [e] Viscosity Sensitivity (*x* value) (measured in a mixture of Glycerol and Ethylene glycol).

**Table S2:** The optimized geometry of precursor compounds of NRhFluors at the ground state ($S_1$). The geometry was optimized using Gaussian computational package version 16.

| Center number | Atomic number | X (Å) | Y (Å) | Z (Å) |
|---|---|---|---|---|
| 1 | C | -4.1892 | 0.2923 | 0.2234 |
| 2 | C | -4.2297 | -1.1261 | 0.0371 |
| 3 | C | -3.0004 | -1.7641 | -0.2157 |
| 4 | C | -1.8251 | -1.0450 | -0.2756 |
| 5 | C | -1.7901 | 0.3621 | -0.0745 |
| 6 | C | -3.0312 | 0.9982 | 0.1809 |
| 7 | O | -0.6844 | -1.7146 | -0.5402 |
| 8 | C | 0.4820 | -1.0836 | -0.6218 |
| 9 | C | 0.6057 | 0.2765 | -0.4284 |
| 10 | C | -0.5555 | 1.0164 | -0.1262 |
| 11 | C | 1.6275 | -1.9820 | -0.9431 |
| 12 | C | 2.9667 | -1.3332 | -0.6046 |
| 13 | C | 3.0019 | 0.0825 | -1.1664 |
| 14 | C | 1.9489 | 0.9439 | -0.4767 |
| 15 | C | -0.4792 | 2.4720 | 0.0955 |
| 16 | C | -0.2941 | 3.0126 | 1.3728 |
| 17 | C | -0.2638 | 4.3965 | 1.5376 |
| 18 | C | -0.4052 | 5.2385 | 0.4520 |
| 19 | C | -0.5868 | 4.7023 | -0.8133 |
| 20 | C | -0.6263 | 3.3297 | -0.9893 |
| 21 | N | -5.3793 | -1.8174 | 0.1436 |
| 22 | C | -5.3594 | -3.2577 | 0.3564 |
| 23 | C | -6.6753 | -1.2320 | -0.1523 |
| 24 | C | -7.2500 | -1.7801 | -1.4594 |
| 25 | C | -6.4642 | -3.7356 | 1.2950 |
| 26 | C | -0.1236 | 2.1847 | 2.5903 |
| 27 | O | -0.1399 | 0.9839 | 2.5938 |
| 28 | O | 0.0421 | 2.8952 | 3.7259 |
| 29 | H | -5.0959 | 0.8245 | 0.4582 |
| 30 | H | -2.9402 | -2.8204 | -0.4139 |
| 31 | H | -3.0376 | 2.0633 | 0.3533 |
| 32 | H | 1.5877 | -2.2094 | -2.0140 |
| 33 | H | 1.4950 | -2.9294 | -0.4164 |
| 34 | H | 3.0987 | -1.3001 | 0.4778 |

| | | | | |
|---|---|---|---|---|
| 35 | H | 3.7747 | -1.9312 | -1.0241 |
| 36 | H | 3.9855 | 0.5254 | -1.0139 |
| 37 | H | 2.8112 | 0.0537 | -2.2406 |
| 38 | H | 1.8606 | 1.9093 | -0.9775 |
| 39 | H | 2.2551 | 1.1473 | 0.5540 |
| 40 | H | -0.1253 | 4.7858 | 2.5346 |
| 41 | H | -0.3765 | 6.3075 | 0.5897 |
| 42 | H | -0.7024 | 5.3517 | -1.6671 |
| 43 | H | -0.7836 | 2.9142 | -1.9740 |
| 44 | H | -5.4321 | -3.7870 | -0.6003 |
| 45 | H | -4.4066 | -3.5130 | 0.8210 |
| 46 | H | -7.3625 | -1.4296 | 0.6724 |
| 47 | H | -6.5759 | -0.1547 | -0.2569 |
| 48 | H | -8.1608 | -1.2400 | -1.7017 |
| 49 | H | -6.5410 | -1.6408 | -2.2722 |
| 50 | H | -7.4929 | -2.8356 | -1.3844 |
| 51 | H | -6.2776 | -4.7766 | 1.5445 |
| 52 | H | -6.4574 | -3.1601 | 2.2173 |
| 53 | H | -7.4496 | -3.6750 | 0.8440 |
| 54 | H | 0.1139 | 2.2785 | 4.4730 |

**Table S3:** The optimized geometry of A1 at the ground state ($S_1$). The geometry was optimized using Gaussian computational package version 16.

| Center number | Atomic number | X (Å) | Y (Å) | Z (Å) |
|---|---|---|---|---|
| 1 | C | 2.0619 | -0.9789 | -0.7016 |
| 2 | C | 2.7337 | 0.1236 | -0.1148 |
| 3 | C | 1.9698 | 1.2582 | 0.2272 |
| 4 | C | 0.5955 | 1.2262 | 0.0929 |
| 5 | C | -0.1421 | 0.0845 | -0.2798 |
| 6 | C | 0.6606 | -0.9940 | -0.7643 |
| 7 | O | -0.0650 | 2.3617 | 0.3957 |
| 8 | C | -1.3682 | 2.4842 | 0.1315 |
| 9 | C | -2.1572 | 1.4324 | -0.2904 |
| 10 | C | -1.5613 | 0.1189 | -0.3440 |
| 11 | C | -1.8228 | 3.8746 | 0.4410 |
| 12 | C | -3.3375 | 3.9960 | 0.5143 |
| 13 | C | -3.9333 | 3.2443 | -0.6645 |
| 14 | C | -3.6173 | 1.7515 | -0.5753 |
| 15 | C | -2.3396 | -1.1246 | -0.0656 |
| 16 | C | -1.7152 | -2.3718 | 0.2688 |
| 17 | C | -2.3836 | -3.5830 | 0.1279 |
| 18 | C | -3.6933 | -3.6359 | -0.3458 |
| 19 | C | -4.3352 | -2.4502 | -0.6525 |
| 20 | C | -3.6540 | -1.2377 | -0.5540 |
| 21 | N | 4.0769 | 0.0850 | 0.0718 |
| 22 | C | 4.7551 | 1.3269 | 0.4849 |
| 23 | C | 4.8806 | -0.9720 | -0.5504 |
| 24 | C | 5.1676 | -0.7111 | -2.0244 |
| 25 | C | 6.2306 | 1.1754 | 0.8145 |
| 26 | C | -0.5341 | -2.4451 | 1.1908 |
| 27 | O | -0.3651 | -1.6848 | 2.1299 |
| 28 | O | 0.2577 | -3.4934 | 0.9606 |
| 29 | H | 2.5560 | -1.8135 | -1.1325 |
| 30 | H | 2.4135 | 2.1823 | 0.5695 |
| 31 | H | 0.1760 | -1.8274 | -1.2591 |
| 32 | H | -1.4274 | 4.5231 | -0.3521 |
| 33 | H | -1.3359 | 4.1818 | 1.3714 |
| 34 | H | -3.7035 | 3.5666 | 1.4542 |

| | | | | |
|---|---|---|---|---|
| 35 | H | -3.6197 | 5.0517 | 0.5008 |
| 36 | H | -5.0198 | 3.3611 | -0.7024 |
| 37 | H | -3.5292 | 3.6544 | -1.5984 |
| 38 | H | -3.8916 | 1.3003 | -1.5312 |
| 39 | H | -4.2480 | 1.3018 | 0.2004 |
| 40 | H | -1.8887 | -4.4959 | 0.4385 |
| 41 | H | -4.1971 | -4.5906 | -0.4497 |
| 42 | H | -5.3554 | -2.4515 | -1.0205 |
| 43 | H | -4.1727 | -0.3782 | -0.9342 |
| 44 | H | 4.6309 | 2.0843 | -0.3006 |
| 45 | H | 4.2479 | 1.6994 | 1.3788 |
| 46 | H | 5.8131 | -1.0529 | 0.0061 |
| 47 | H | 4.3768 | -1.9289 | -0.4112 |
| 48 | H | 5.7845 | -1.5179 | -2.4315 |
| 49 | H | 4.2413 | -0.6595 | -2.6054 |
| 50 | H | 5.7074 | 0.2323 | -2.1532 |
| 51 | H | 6.5878 | 2.1417 | 1.1813 |
| 52 | H | 6.3978 | 0.4355 | 1.6027 |
| 53 | H | 6.8337 | 0.9092 | -0.0575 |
| 54 | H | 0.9457 | -3.5257 | 1.6452 |

**Table S4:** The optimized geometry of A11 at the ground state ($S_1$). The geometry was optimized using Gaussian computational package version 16.

| Center number | Atomic number | X (Å) | Y (Å) | Z (Å) |
|---|---|---|---|---|
| 1 | C | -0.8605 | 3.0298 | -0.0798 |
| 2 | C | 0.5503 | 2.7779 | -0.0996 |
| 3 | C | 0.9663 | 1.4296 | -0.2717 |
| 4 | C | 0.0315 | 0.4279 | -0.3710 |
| 5 | C | -1.3630 | 0.6526 | -0.3228 |
| 6 | C | -1.7664 | 2.0055 | -0.1841 |
| 7 | O | 0.5203 | -0.8249 | -0.5512 |
| 8 | C | -0.3007 | -1.9180 | -0.5371 |
| 9 | C | -1.7080 | -1.7439 | -0.5283 |
| 10 | C | -2.2448 | -0.4642 | -0.4207 |
| 11 | C | 0.3501 | -3.1671 | -0.6111 |
| 12 | C | -0.5393 | -4.3536 | -0.9145 |
| 13 | C | -1.8749 | -4.2389 | -0.1849 |
| 14 | C | -2.5952 | -2.9588 | -0.5935 |
| 15 | C | -3.7176 | -0.2466 | -0.4202 |
| 16 | C | -4.4449 | 0.0647 | 0.7441 |
| 17 | C | -5.8197 | 0.3118 | 0.6650 |
| 18 | C | -6.4832 | 0.2332 | -0.5541 |
| 19 | C | -5.7695 | -0.0777 | -1.7085 |
| 20 | C | -4.3979 | -0.3072 | -1.6388 |
| 21 | N | 1.4537 | 3.7758 | 0.0464 |
| 22 | C | 1.0371 | 5.1747 | 0.1707 |
| 23 | C | 2.8929 | 3.5094 | -0.0139 |
| 24 | C | 3.4347 | 3.3645 | -1.4353 |
| 25 | C | 0.7287 | 5.8420 | -1.1682 |
| 26 | C | 1.7086 | -3.4278 | -0.3725 |
| 27 | C | 2.8156 | -2.6053 | -0.0047 |
| 28 | C | 4.1080 | -2.9989 | -0.4499 |
| 29 | C | 5.2401 | -2.2669 | -0.1588 |
| 30 | C | 5.1306 | -1.1244 | 0.6587 |
| 31 | C | 3.8766 | -0.7607 | 1.1906 |
| 32 | C | 2.7511 | -1.4791 | 0.8693 |
| 33 | O | 6.1582 | -0.3440 | 1.0037 |
| 34 | C | 7.4689 | -0.6711 | 0.5395 |

| | | | | |
|---|---|---|---|---|
| 35 | C | -3.7730 | 0.0959 | 2.0745 |
| 36 | O | -2.7642 | -0.5119 | 2.3682 |
| 37 | O | -4.4163 | 0.8735 | 2.9598 |
| 38 | H | -1.2377 | 4.0396 | 0.0140 |
| 39 | H | 2.0090 | 1.1500 | -0.3386 |
| 40 | H | -2.8256 | 2.2404 | -0.1632 |
| 41 | H | -0.7212 | -4.3947 | -1.9982 |
| 42 | H | -0.0277 | -5.2783 | -0.6374 |
| 43 | H | -2.5036 | -5.1037 | -0.4149 |
| 44 | H | -1.6996 | -4.2374 | 0.8975 |
| 45 | H | -2.9570 | -3.0574 | -1.6272 |
| 46 | H | -3.4802 | -2.7963 | 0.0278 |
| 47 | H | -6.3721 | 0.5525 | 1.5664 |
| 48 | H | -7.5518 | 0.4158 | -0.6007 |
| 49 | H | -6.2774 | -0.1367 | -2.6660 |
| 50 | H | -3.8387 | -0.5330 | -2.5419 |
| 51 | H | 0.1756 | 5.2329 | 0.8406 |
| 52 | H | 1.8508 | 5.7049 | 0.6690 |
| 53 | H | 3.3915 | 4.3411 | 0.4868 |
| 54 | H | 3.1148 | 2.6157 | 0.5766 |
| 55 | H | 2.9303 | 2.5617 | -1.9809 |
| 56 | H | 3.3094 | 4.2932 | -1.9983 |
| 57 | H | 4.5031 | 3.1306 | -1.3937 |
| 58 | H | 1.6163 | 5.8694 | -1.8060 |
| 59 | H | -0.0652 | 5.3141 | -1.7048 |
| 60 | H | 0.3994 | 6.8713 | -0.9952 |
| 61 | H | 2.0063 | -4.4314 | -0.6760 |
| 62 | H | 4.1988 | -3.8854 | -1.0714 |
| 63 | H | 6.1976 | -2.5835 | -0.5539 |
| 64 | H | 3.8231 | 0.0840 | 1.8698 |
| 65 | H | 1.8097 | -1.2092 | 1.3293 |
| 66 | H | 8.1235 | 0.0992 | 0.9451 |
| 67 | H | 7.7801 | -1.6516 | 0.9132 |
| 68 | H | 7.5115 | -0.6515 | -0.5540 |
| 69 | H | -3.9460 | 0.8066 | 3.8059 |

**Table S5:** The optimized geometry of B1 at the ground state ($S_1$). The geometry was optimized using Gaussian computational package version 16.

| Center number | Atomic number | X (Å) | Y (Å) | Z (Å) |
|---|---|---|---|---|
| 1 | C | -0.5493 | 3.0067 | -0.1288 |
| 2 | C | 0.8147 | 2.5638 | -0.1801 |
| 3 | C | 1.0452 | 1.1682 | -0.3849 |
| 4 | C | -0.0190 | 0.3064 | -0.4675 |
| 5 | C | -1.3631 | 0.7125 | -0.3739 |
| 6 | C | -1.5814 | 2.1113 | -0.2185 |
| 7 | O | 0.2995 | -0.9958 | -0.6689 |
| 8 | C | -0.6356 | -1.9699 | -0.5666 |
| 9 | C | -2.0136 | -1.6252 | -0.5267 |
| 10 | C | -2.3862 | -0.2867 | -0.4336 |
| 11 | C | -0.1351 | -3.2978 | -0.5839 |
| 12 | C | -1.1649 | -4.3800 | -0.8331 |
| 13 | C | -2.4644 | -4.0721 | -0.0935 |
| 14 | C | -3.0345 | -2.7293 | -0.5418 |
| 15 | C | -3.8202 | 0.1106 | -0.4124 |
| 16 | C | -4.5000 | 0.5020 | 0.7577 |
| 17 | C | -5.8412 | 0.8947 | 0.6836 |
| 18 | C | -6.5157 | 0.8912 | -0.5318 |
| 19 | C | -5.8481 | 0.5027 | -1.6898 |
| 20 | C | -4.5108 | 0.1207 | -1.6267 |
| 21 | N | 1.8489 | 3.4121 | -0.0427 |
| 22 | C | 1.6512 | 4.8672 | -0.0482 |
| 23 | C | 3.2338 | 2.9356 | 0.0539 |
| 24 | C | 3.9389 | 2.8668 | -1.2982 |
| 25 | C | 1.3217 | 5.4197 | -1.4340 |
| 26 | C | 1.1762 | -3.6661 | -0.3373 |
| 27 | C | 2.3422 | -2.8672 | -0.0122 |
| 28 | C | 3.5795 | -3.2009 | -0.6044 |
| 29 | C | 4.7138 | -2.4461 | -0.3623 |
| 30 | C | 4.6293 | -1.3745 | 0.5273 |
| 31 | C | 3.4489 | -1.0727 | 1.2051 |
| 32 | C | 2.3162 | -1.8170 | 0.9339 |
| 33 | C | -3.8140 | 0.4743 | 2.0807 |
| 34 | O | -2.7695 | -0.0972 | 2.3207 |

| | | | | |
|---|---|---|---|---|
| 35 | O | -4.4792 | 1.1556 | 3.0261 |
| 36 | N | 5.8058 | -0.5520 | 0.7639 |
| 37 | O | 6.8594 | -0.8734 | 0.2234 |
| 38 | O | 5.6870 | 0.4353 | 1.4853 |
| 39 | H | -0.7818 | 4.0550 | 0.0033 |
| 40 | H | 2.0402 | 0.7643 | -0.5085 |
| 41 | H | -2.6001 | 2.4803 | -0.1610 |
| 42 | H | -1.3652 | -4.4407 | -1.9115 |
| 43 | H | -0.7649 | -5.3470 | -0.5204 |
| 44 | H | -3.1988 | -4.8599 | -0.2821 |
| 45 | H | -2.2741 | -4.0518 | 0.9860 |
| 46 | H | -3.4137 | -2.8178 | -1.5703 |
| 47 | H | -3.8901 | -2.4432 | 0.0761 |
| 48 | H | -6.3606 | 1.1934 | 1.5868 |
| 49 | H | -7.5577 | 1.1908 | -0.5722 |
| 50 | H | -6.3649 | 0.4990 | -2.6443 |
| 51 | H | -3.9865 | -0.1713 | -2.5317 |
| 52 | H | 0.8703 | 5.1241 | 0.6734 |
| 53 | H | 2.5780 | 5.3109 | 0.3164 |
| 54 | H | 3.7585 | 3.6276 | 0.7159 |
| 55 | H | 3.2433 | 1.9645 | 0.5514 |
| 56 | H | 3.4022 | 2.2120 | -1.9915 |
| 57 | H | 4.0225 | 3.8591 | -1.7492 |
| 58 | H | 4.9484 | 2.4706 | -1.1550 |
| 59 | H | 2.1258 | 5.2009 | -2.1414 |
| 60 | H | 0.3912 | 5.0019 | -1.8278 |
| 61 | H | 1.2068 | 6.5054 | -1.3659 |
| 62 | H | 1.4018 | -4.7115 | -0.5456 |
| 63 | H | 3.6310 | -4.0348 | -1.2976 |
| 64 | H | 5.6501 | -2.6753 | -0.8563 |
| 65 | H | 3.4246 | -0.2728 | 1.9350 |
| 66 | H | 1.4038 | -1.6037 | 1.4786 |
| 67 | H | -3.9870 | 1.0616 | 3.8569 |

**Table S6:** The optimized geometry of B8 at the ground state ($S_1$). The geometry was optimized using Gaussian computational package version 16.

| Center number | Atomic number | X (Å) | Y (Å) | Z (Å) |
|---|---|---|---|---|
| 1 | C | 0.0726 | 2.9901 | 0.0087 |
| 2 | C | 1.4285 | 2.5249 | -0.0166 |
| 3 | C | 1.6395 | 1.1224 | -0.2158 |
| 4 | C | 0.5618 | 0.2826 | -0.3479 |
| 5 | C | -0.7760 | 0.7171 | -0.3073 |
| 6 | C | -0.9723 | 2.1160 | -0.1301 |
| 7 | O | 0.8635 | -1.0255 | -0.5450 |
| 8 | C | -0.1018 | -1.9755 | -0.5243 |
| 9 | C | -1.4702 | -1.5996 | -0.5468 |
| 10 | C | -1.8192 | -0.2548 | -0.4424 |
| 11 | C | 0.3648 | -3.3197 | -0.5425 |
| 12 | C | -0.6779 | -4.3622 | -0.8893 |
| 13 | C | -2.0048 | -4.0461 | -0.2044 |
| 14 | C | -2.5174 | -2.6758 | -0.6378 |
| 15 | C | -3.2413 | 0.1777 | -0.4928 |
| 16 | C | -3.9694 | 0.5865 | 0.6417 |
| 17 | C | -5.2938 | 1.0165 | 0.5013 |
| 18 | C | -5.9058 | 1.0331 | -0.7466 |
| 19 | C | -5.1911 | 0.6277 | -1.8702 |
| 20 | C | -3.8696 | 0.2093 | -1.7403 |
| 21 | N | 2.4726 | 3.3597 | 0.1331 |
| 22 | C | 2.2862 | 4.8161 | 0.1694 |
| 23 | C | 3.8556 | 2.8699 | 0.1861 |
| 24 | C | 4.5045 | 2.7548 | -1.1917 |
| 25 | C | 1.9917 | 5.4196 | -1.2033 |
| 26 | C | 1.6387 | -3.7351 | -0.2198 |
| 27 | C | 2.8189 | -2.9935 | 0.2080 |
| 28 | C | 4.0797 | -3.3775 | -0.2802 |
| 29 | C | 5.2124 | -2.6893 | 0.1335 |
| 30 | N | 5.1900 | -1.6874 | 1.0228 |
| 31 | C | 3.9963 | -1.3575 | 1.5334 |
| 32 | C | 2.8029 | -1.9636 | 1.1663 |
| 33 | C | -3.3537 | 0.5350 | 1.9980 |
| 34 | O | -2.3501 | -0.0820 | 2.2931 |

| | | | | |
|---|---|---|---|---|
| 35 | O | -4.0340 | 1.2525 | 2.9057 |
| 36 | H | -0.1463 | 4.0401 | 0.1481 |
| 37 | H | 2.6294 | 0.6937 | -0.2952 |
| 38 | H | -1.9853 | 2.5035 | -0.0998 |
| 39 | H | -0.8250 | -4.3736 | -1.9778 |
| 40 | H | -0.3184 | -5.3515 | -0.5978 |
| 41 | H | -2.7481 | -4.8079 | -0.4548 |
| 42 | H | -1.8684 | -4.0645 | 0.8832 |
| 43 | H | -2.8550 | -2.7254 | -1.6831 |
| 44 | H | -3.3907 | -2.3819 | -0.0486 |
| 45 | H | -5.8495 | 1.3282 | 1.3781 |
| 46 | H | -6.9357 | 1.3618 | -0.8389 |
| 47 | H | -5.6580 | 0.6397 | -2.8500 |
| 48 | H | -3.3084 | -0.0948 | -2.6189 |
| 49 | H | 1.4904 | 5.0552 | 0.8802 |
| 50 | H | 3.2068 | 5.2401 | 0.5708 |
| 51 | H | 4.4137 | 3.5737 | 0.8060 |
| 52 | H | 3.8743 | 1.9120 | 0.7083 |
| 53 | H | 3.9362 | 2.0841 | -1.8431 |
| 54 | H | 4.5785 | 3.7316 | -1.6762 |
| 55 | H | 5.5145 | 2.3510 | -1.0767 |
| 56 | H | 2.8216 | 5.2454 | -1.8927 |
| 57 | H | 1.0817 | 5.0022 | -1.6431 |
| 58 | H | 1.8549 | 6.4993 | -1.0940 |
| 59 | H | 1.8348 | -4.7908 | -0.4051 |
| 60 | H | 4.1700 | -4.1872 | -0.9979 |
| 61 | H | 6.1874 | -2.9585 | -0.2645 |
| 62 | H | 3.9937 | -0.5667 | 2.2796 |
| 63 | H | 1.8796 | -1.6580 | 1.6446 |
| 64 | H | -3.5903 | 1.1397 | 3.7612 |

# 3. Supplementary Figures
## 3.1 Absorbance Spectra, Fluorescence Spectra

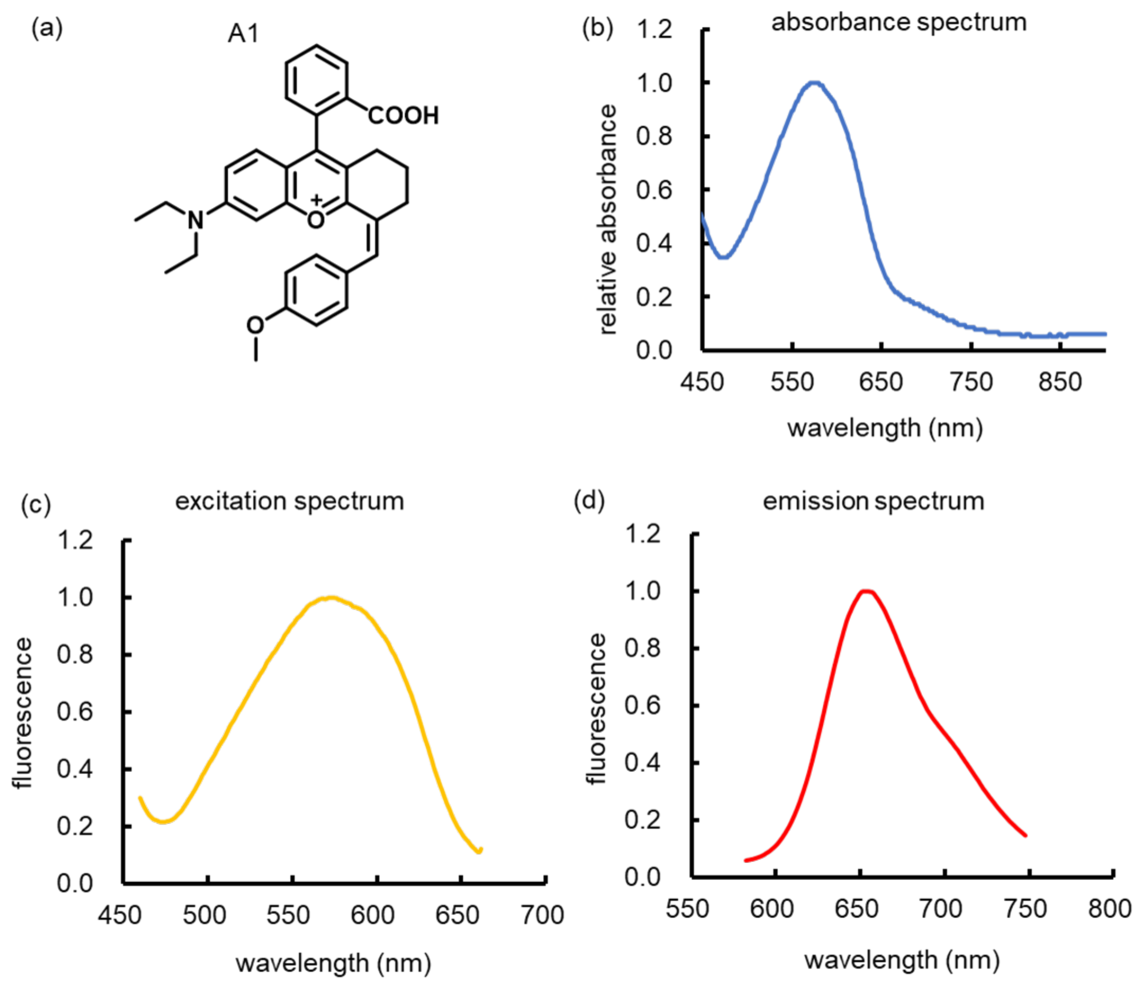


**Figure S1.** (a) Structure of A1. (b) Normalized absorbance spectrum. (c) Normalized excitation spectrum. (d) Normalized emission spectrum. All spectra were collected using 20 μM compound in 80% glycerol and 20% ethylene glycol.

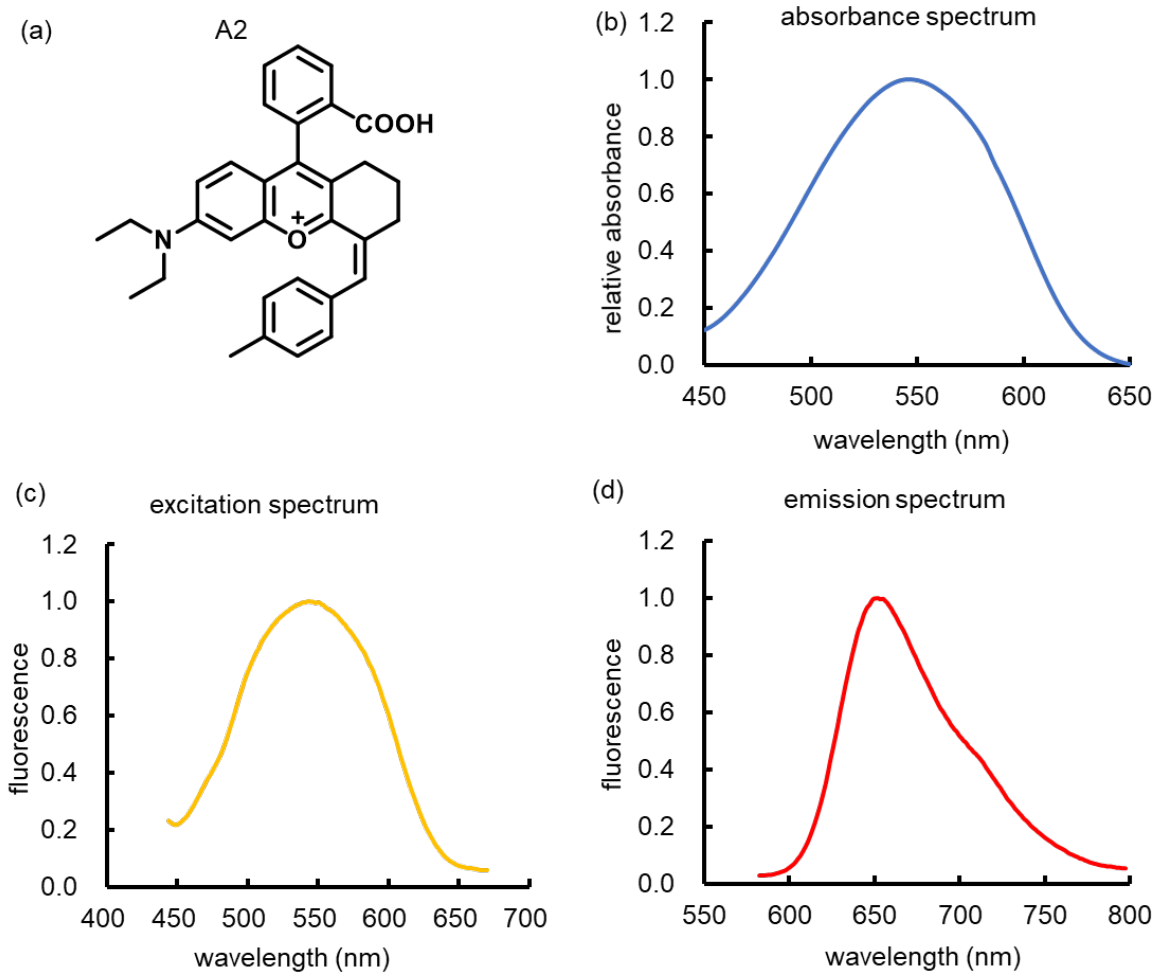


**Figure S2.** (a) Structure of A2. (b) Normalized absorbance spectrum. (c) Normalized excitation spectrum. (d) Normalized emission spectrum. All spectra were collected using 20 μM compound in 80% glycerol and 20% ethylene glycol.

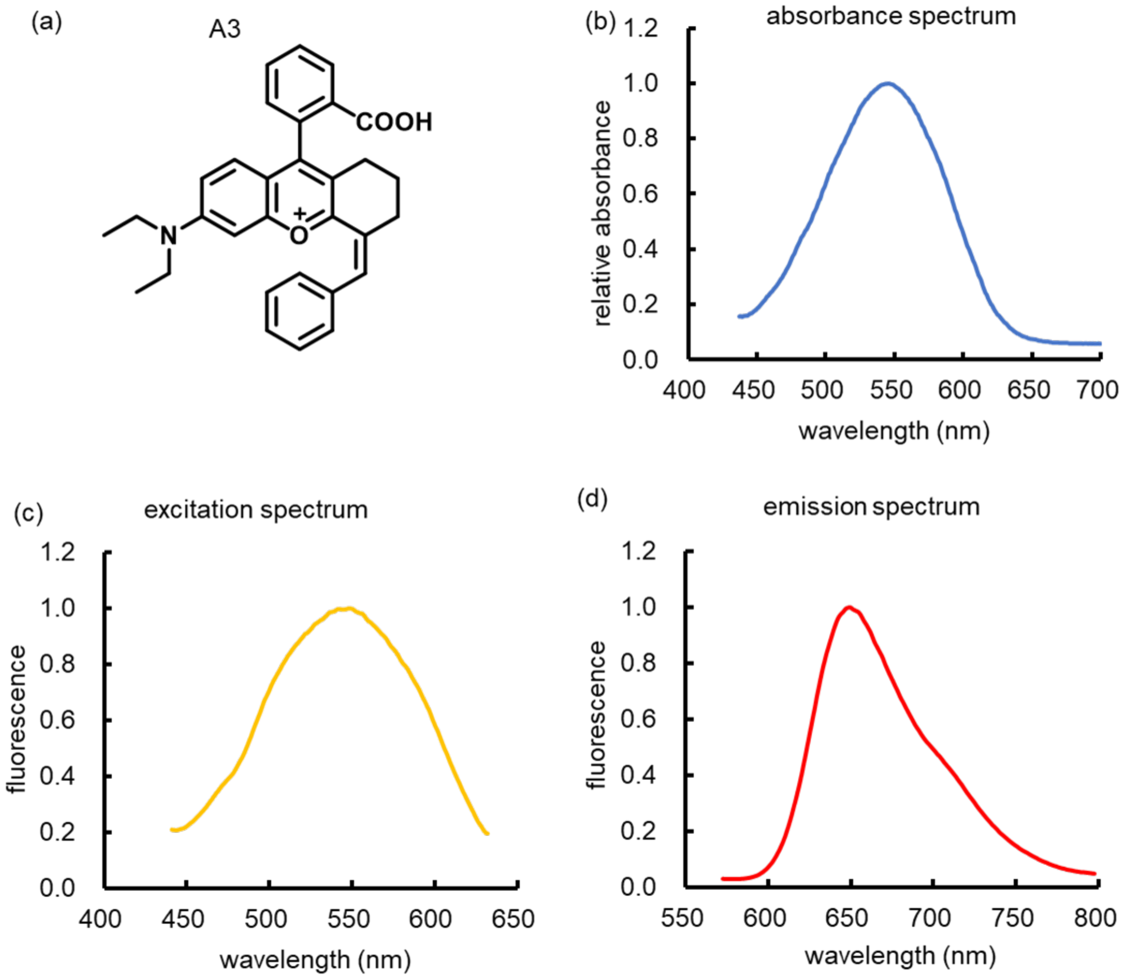


**Figure S3.** (a) Structure of A3. (b) Normalized absorbance spectrum. (c) Normalized excitation spectrum. (d) Normalized emission spectrum. All spectra were collected using 20 μM compound in 80% glycerol and 20% ethylene glycol.

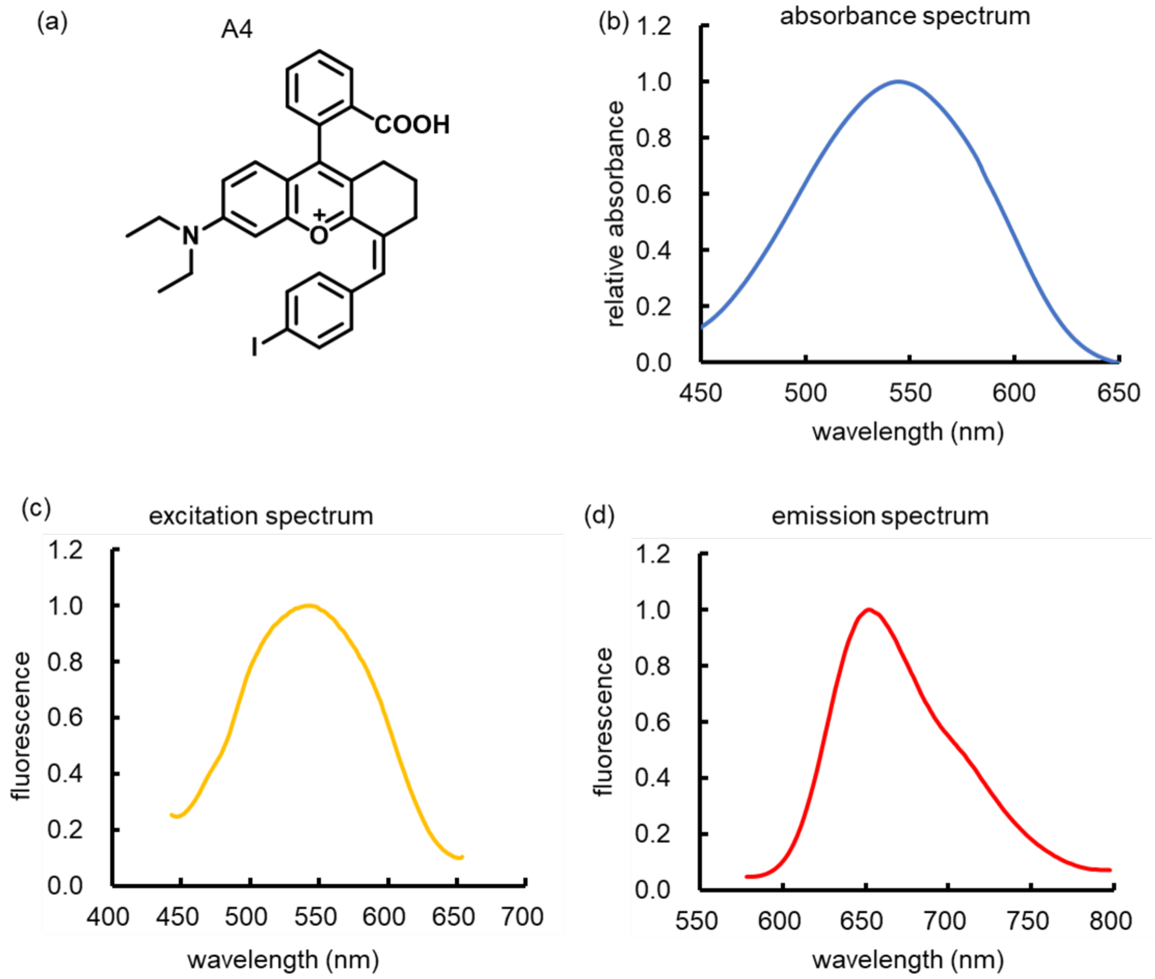


**Figure S4.** (a) Structure of A4. (b) Normalized absorbance spectrum. (c) Normalized excitation spectrum. (d) Normalized emission spectrum. All spectra were collected using 20 µM compound in 80% glycerol and 20% ethylene glycol.

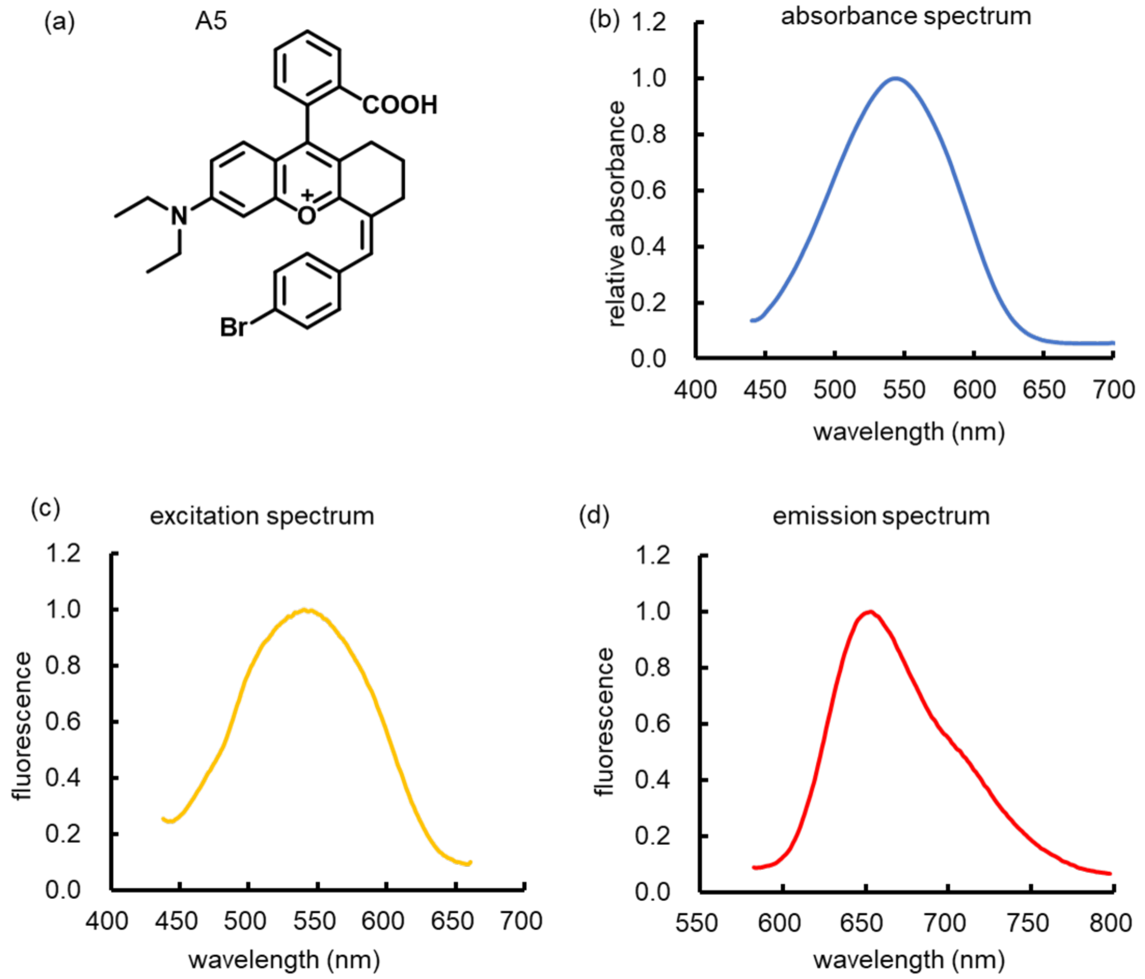


**Figure S5.** (a) Structure of A5. (b) Normalized absorbance spectrum. (c) Normalized excitation spectrum. (d) Normalized emission spectrum. All spectra were collected using 20 µM compound in 80% glycerol and 20% ethylene glycol.

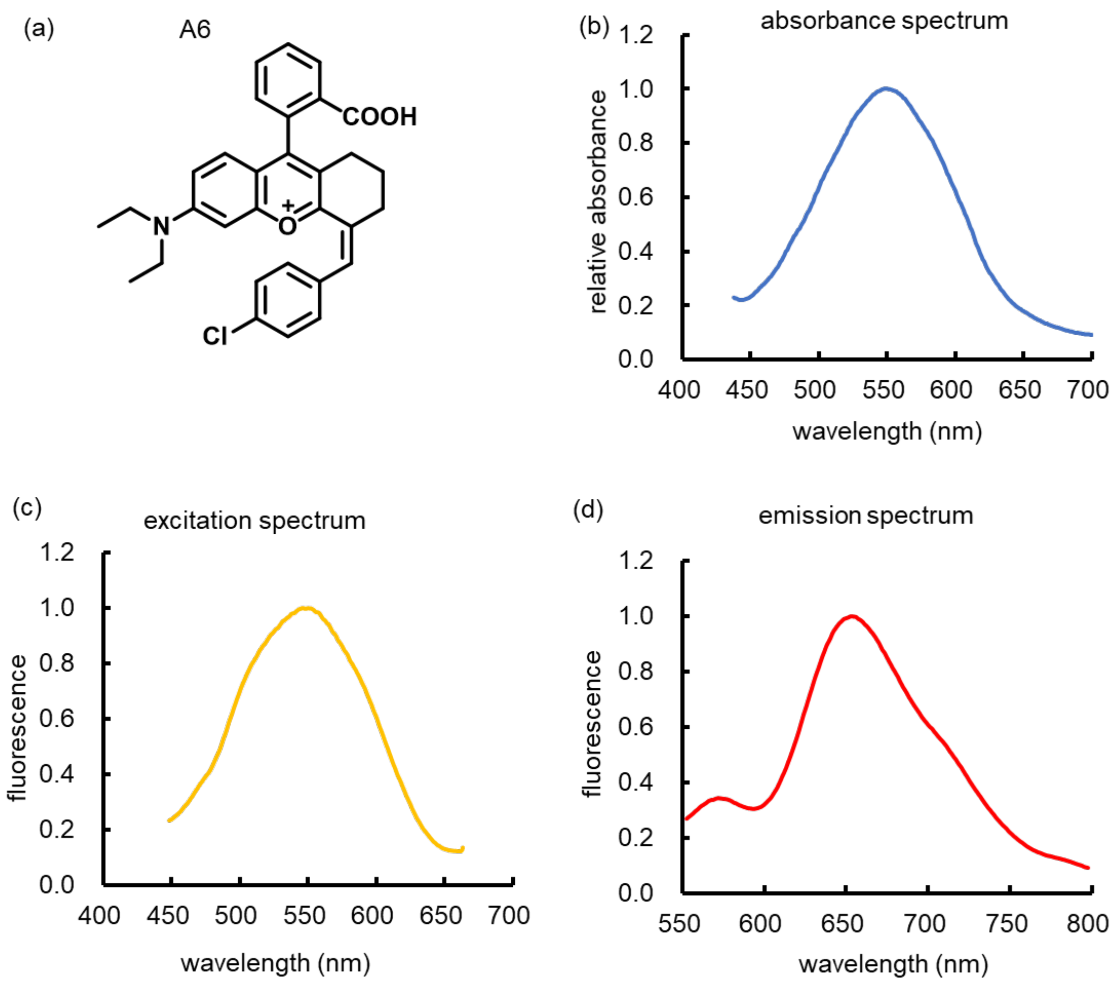


**Figure S6.** (a) Structure of A6. (b) Normalized absorbance spectrum. (c) Normalized excitation spectrum. (d) Normalized emission spectrum. All spectra were collected using 20 μM compound in 80% glycerol and 20% ethylene glycol.

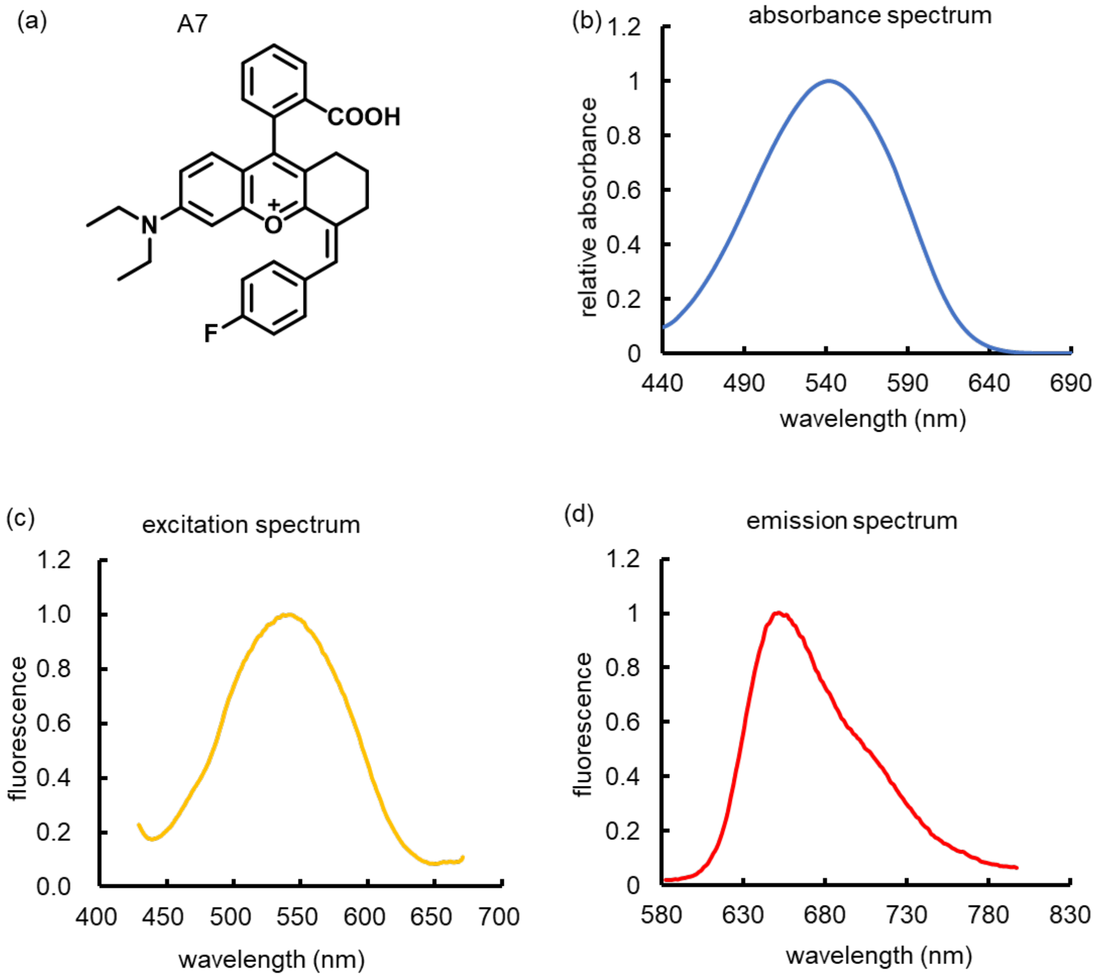


**Figure S7.** (a) Structure of A7. (b) Normalized absorbance spectrum. (c) Normalized excitation spectrum. (d) Normalized emission spectrum. All spectra were collected using 20 μM compound in 80% glycerol and 20% ethylene glycol.

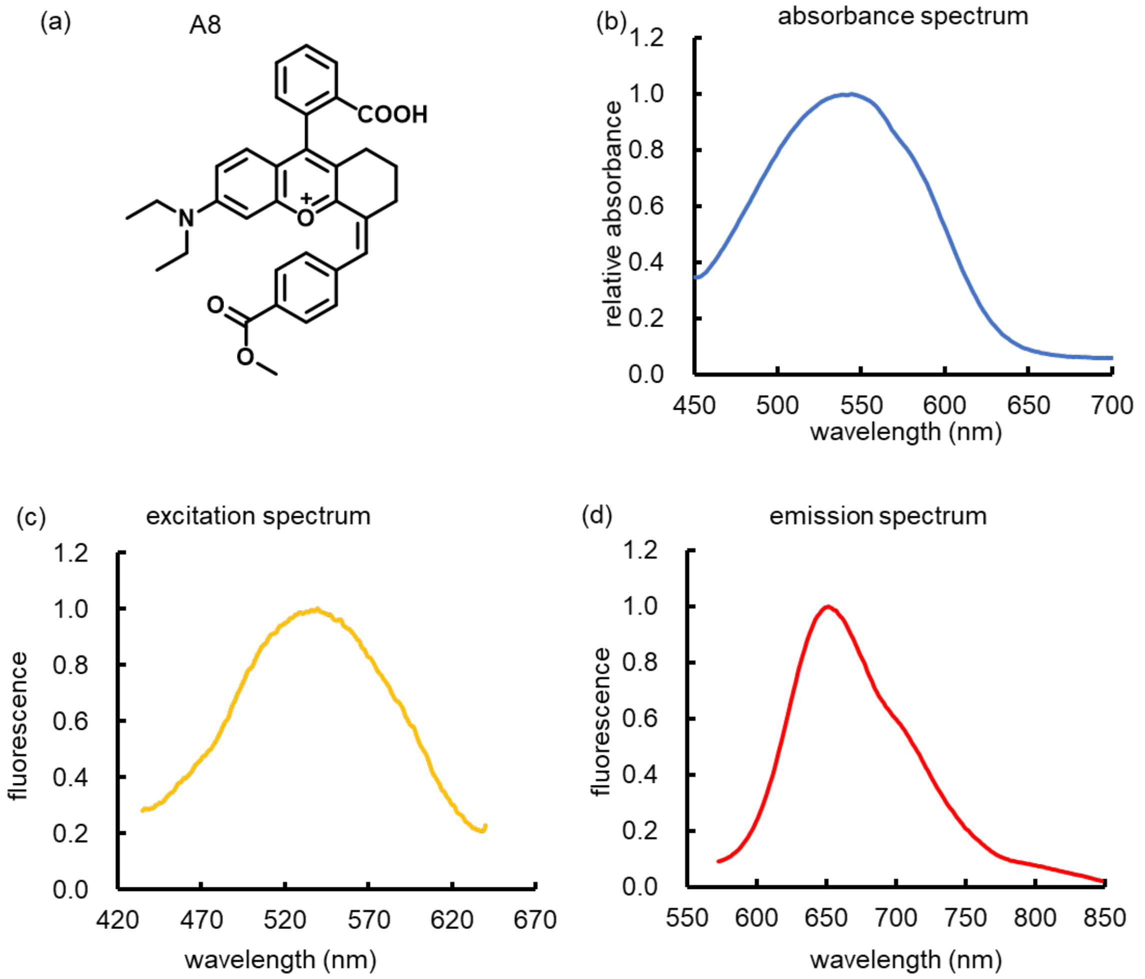


**Figure S8.** (a) Structure of A8. (b) Normalized absorbance spectrum. (c) Normalized excitation spectrum. (d) Normalized emission spectrum. All spectra were collected using 20 μM compound in 80% glycerol and 20% ethylene glycol.

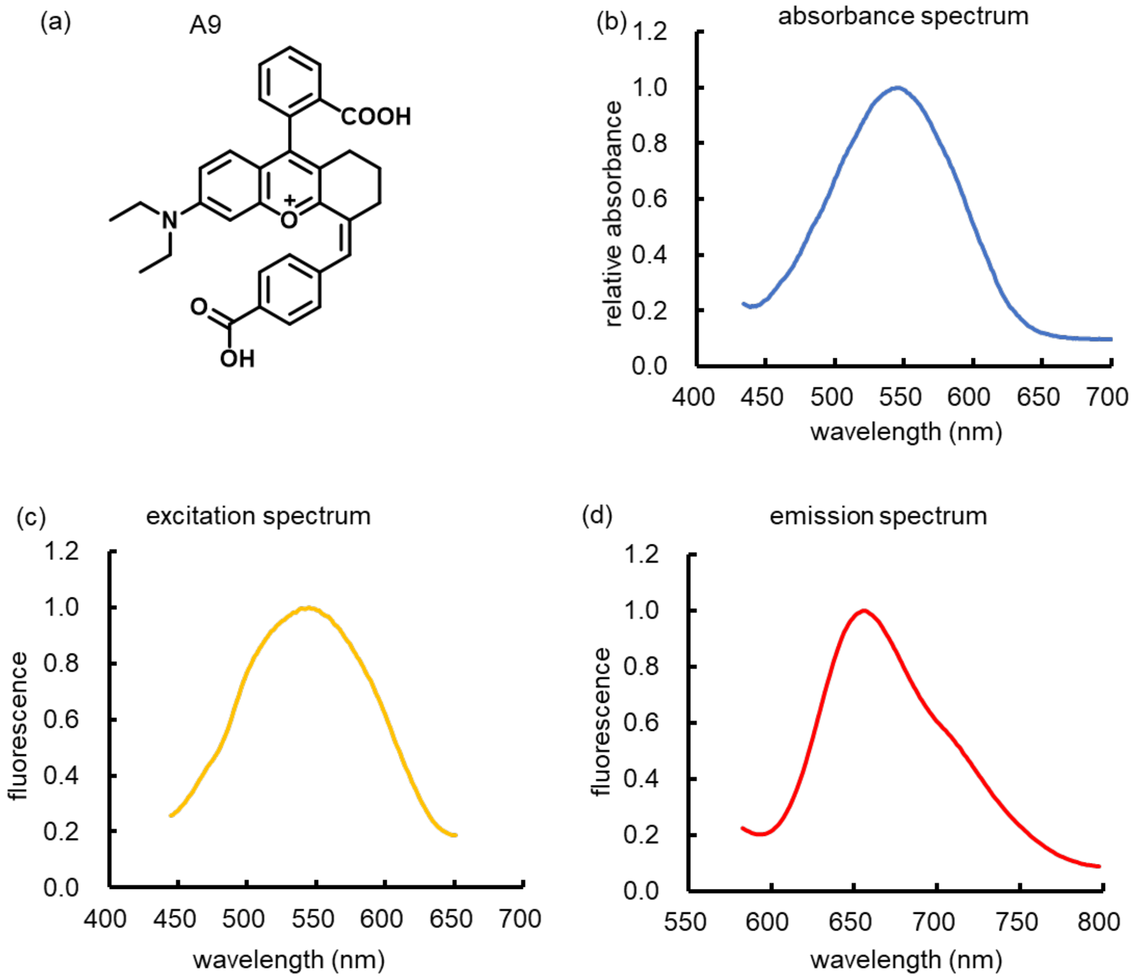


**Figure S9.** (a) Structure of A9. (b) Normalized absorbance spectrum. (c) Normalized excitation spectrum. (d) Normalized emission spectrum. All spectra were collected using 20 μM compound in 80% glycerol and 20% ethylene glycol.

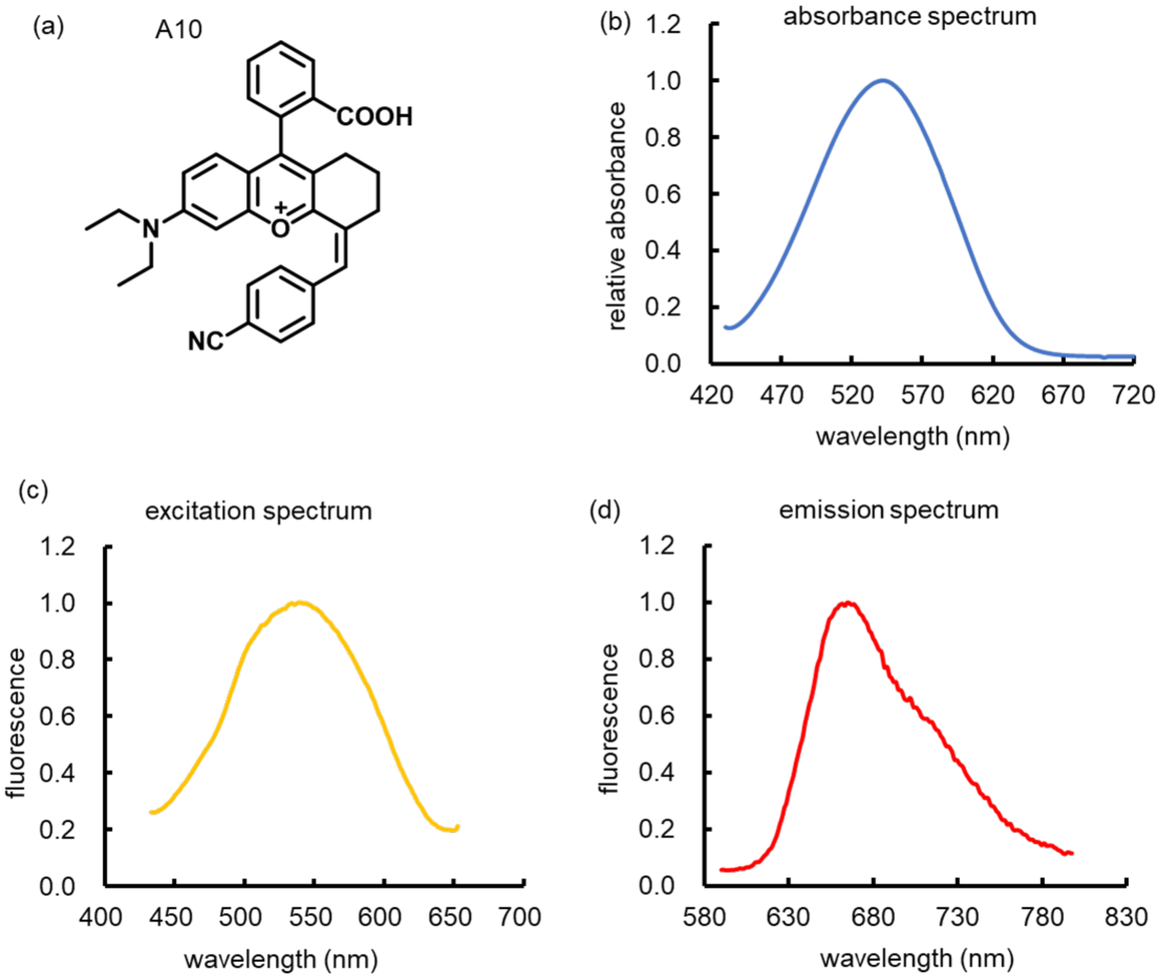


**Figure S10.** (a) Structure of A10. (b) Normalized absorbance spectrum. (c) Normalized excitation spectrum. (d) Normalized emission spectrum. All spectra were collected using 20 μM compound in 80% glycerol and 20% ethylene glycol.

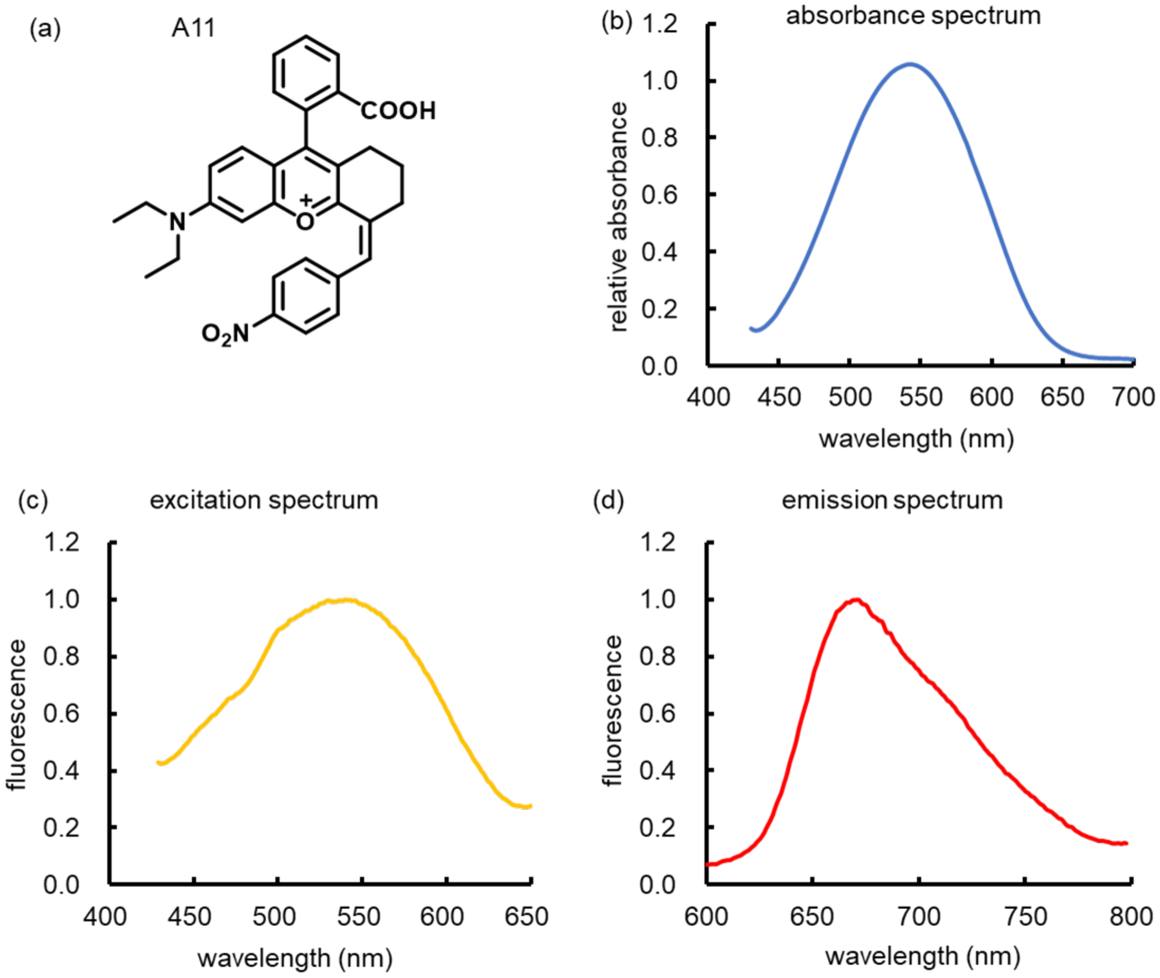


**Figure S11.** (a) Structure of A11. (b) Normalized absorbance spectrum. (c) Normalized excitation spectrum. (d) Normalized emission spectrum. All spectra were collected using 20 μM compound in 80% glycerol and 20% ethylene glycol.

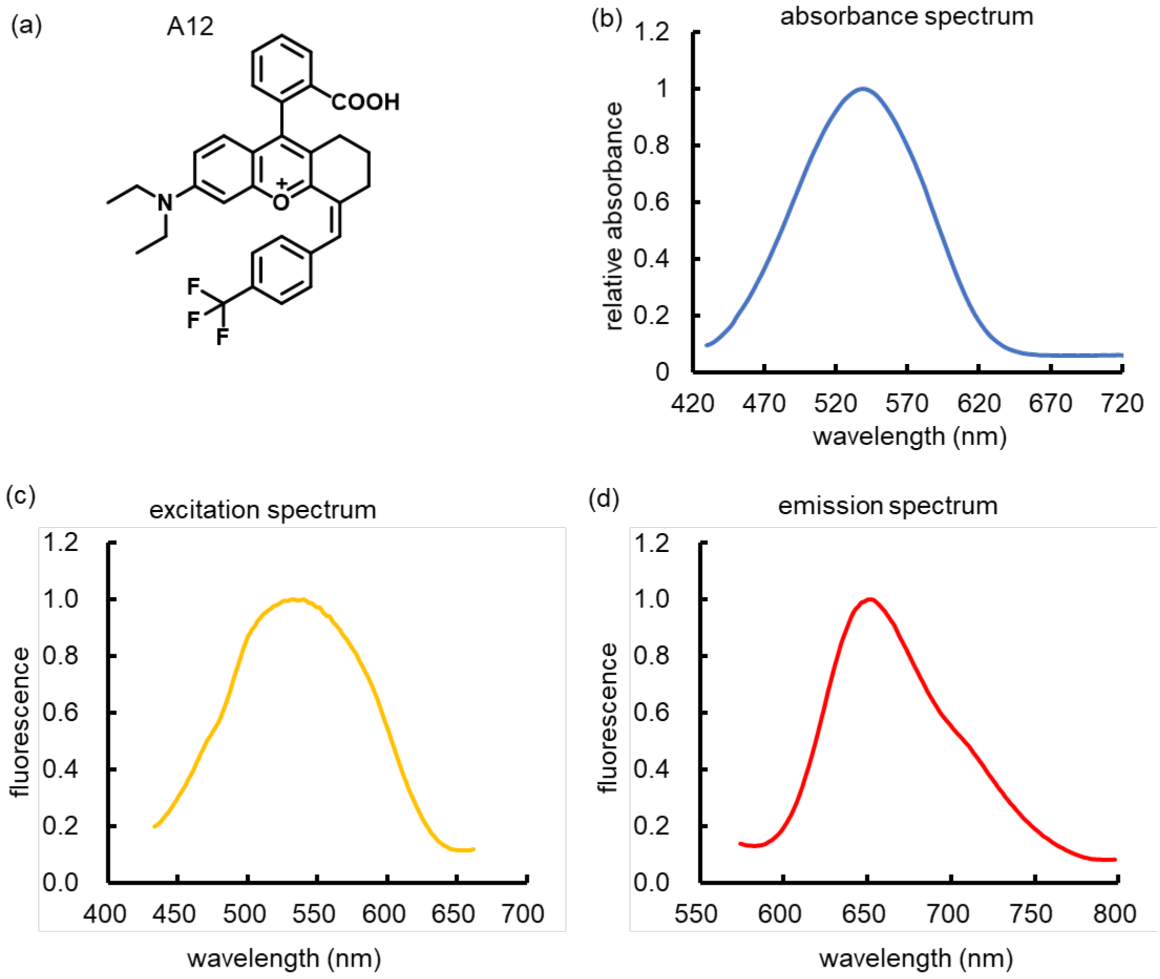


**Figure S12.** (a) Structure of A12. (b) Normalized absorbance spectrum. (c) Normalized excitation spectrum. (d) Normalized emission spectrum. All spectra were collected using 20 μM compound in 80% glycerol and 20% ethylene glycol.

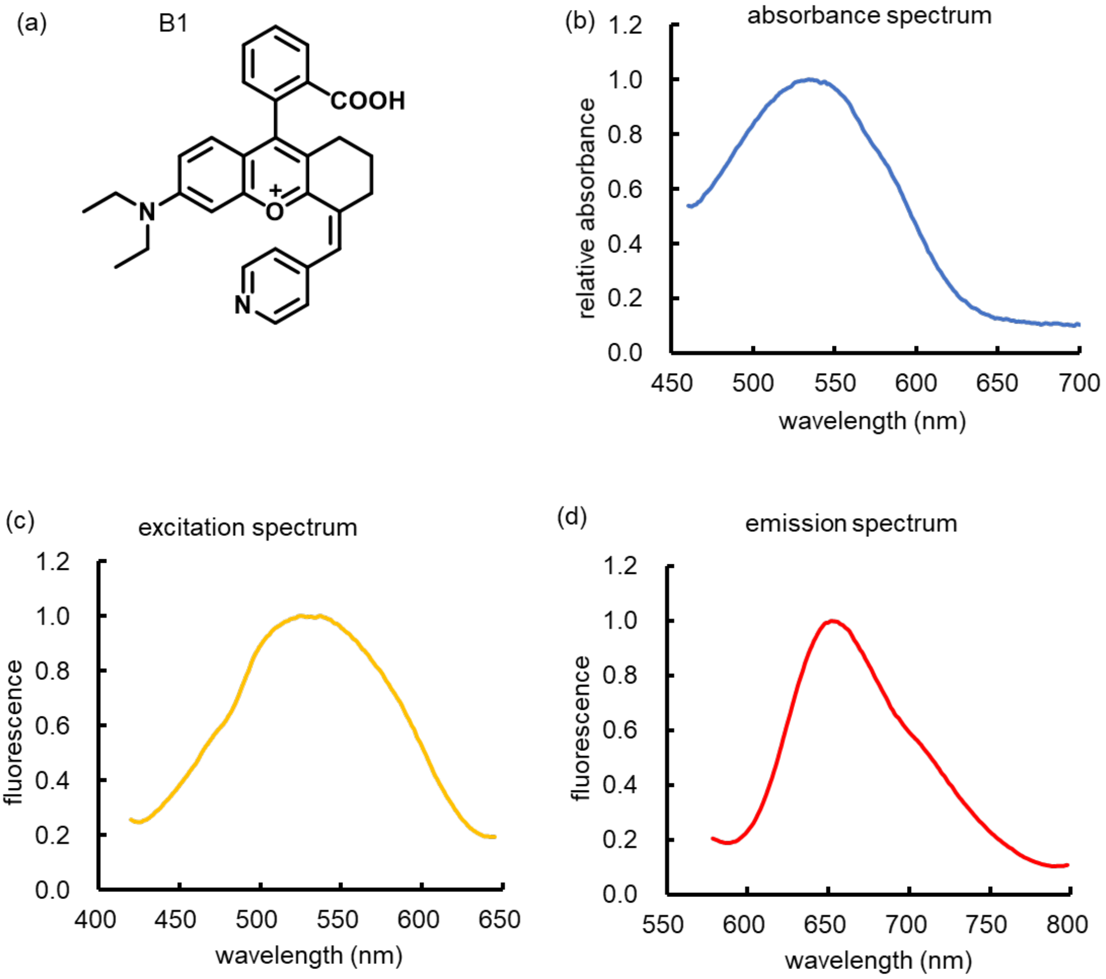


**Figure S13.** (a) Structure of B1. (b) Normalized absorbance spectrum. (c) Normalized excitation spectrum. (d) Normalized emission spectrum. All spectra were collected using 20 µM compound in 80% glycerol and 20% ethylene glycol.

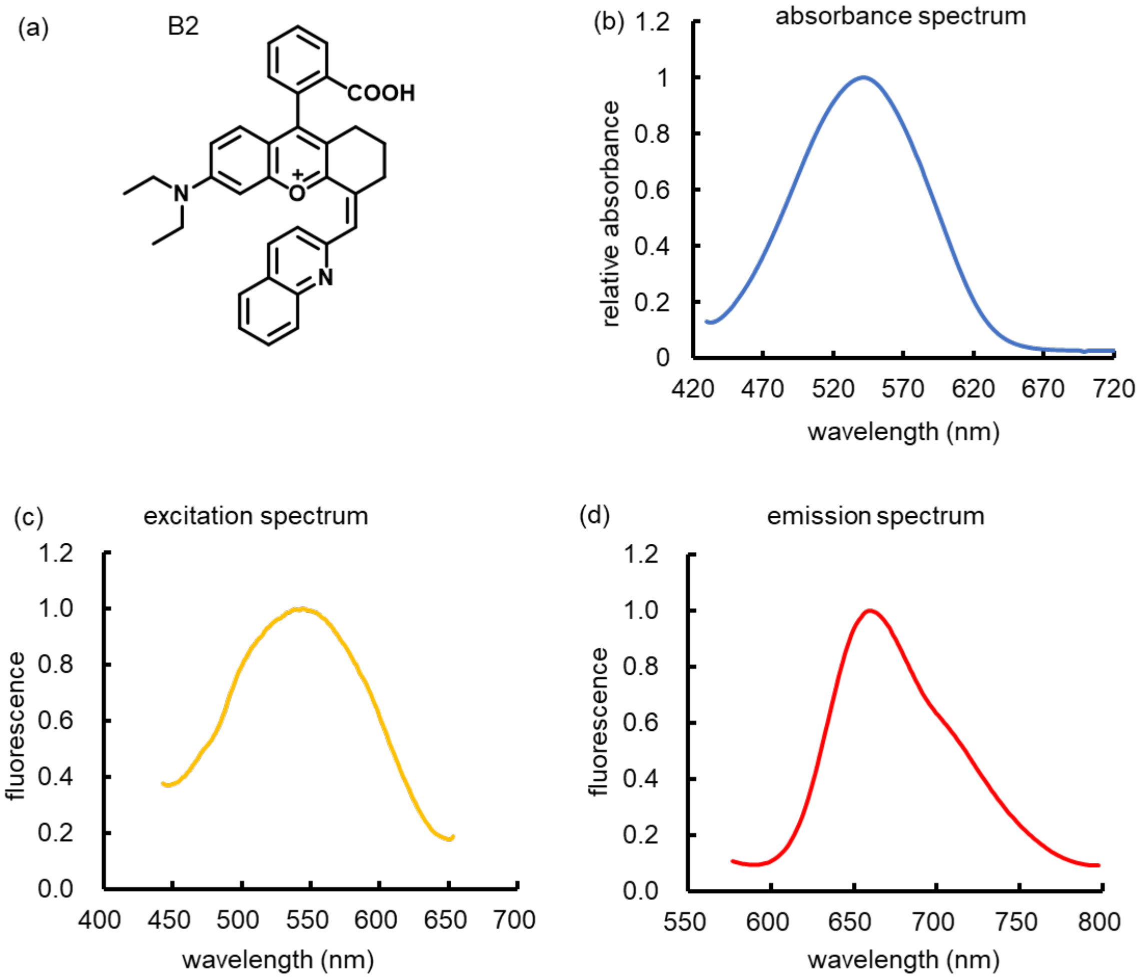


**Figure S14.** (a) Structure of B2. (b) Normalized absorbance spectrum. (c) Normalized excitation spectrum. (d) Normalized emission spectrum. All spectra were collected using 20 μM compound in 80% glycerol and 20% ethylene glycol.

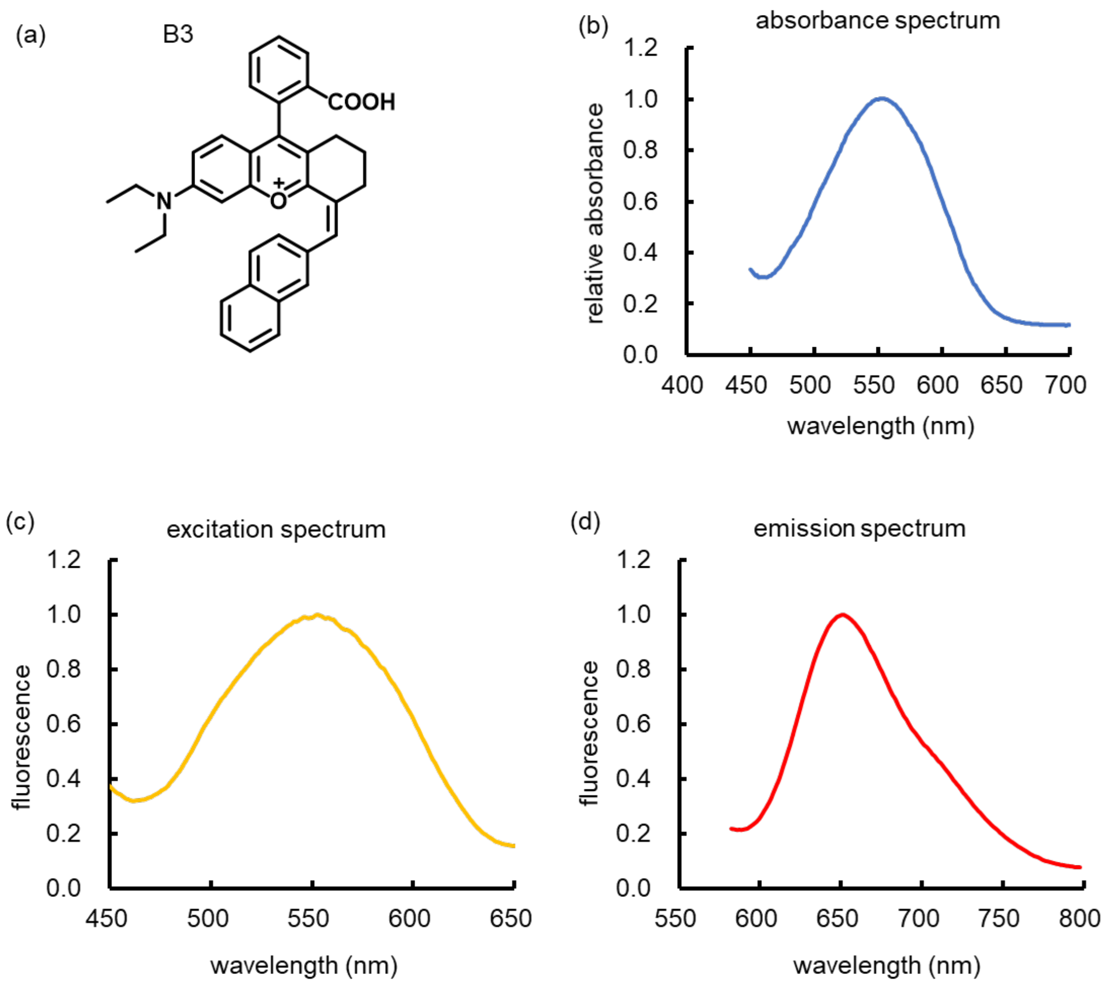


**Figure S15.** (a) Structure of B3. (b) Normalized absorbance spectrum. (c) Normalized excitation spectrum. (d) Normalized emission spectrum. All spectra were collected using 20 µM compound in 80% glycerol and 20% ethylene glycol.

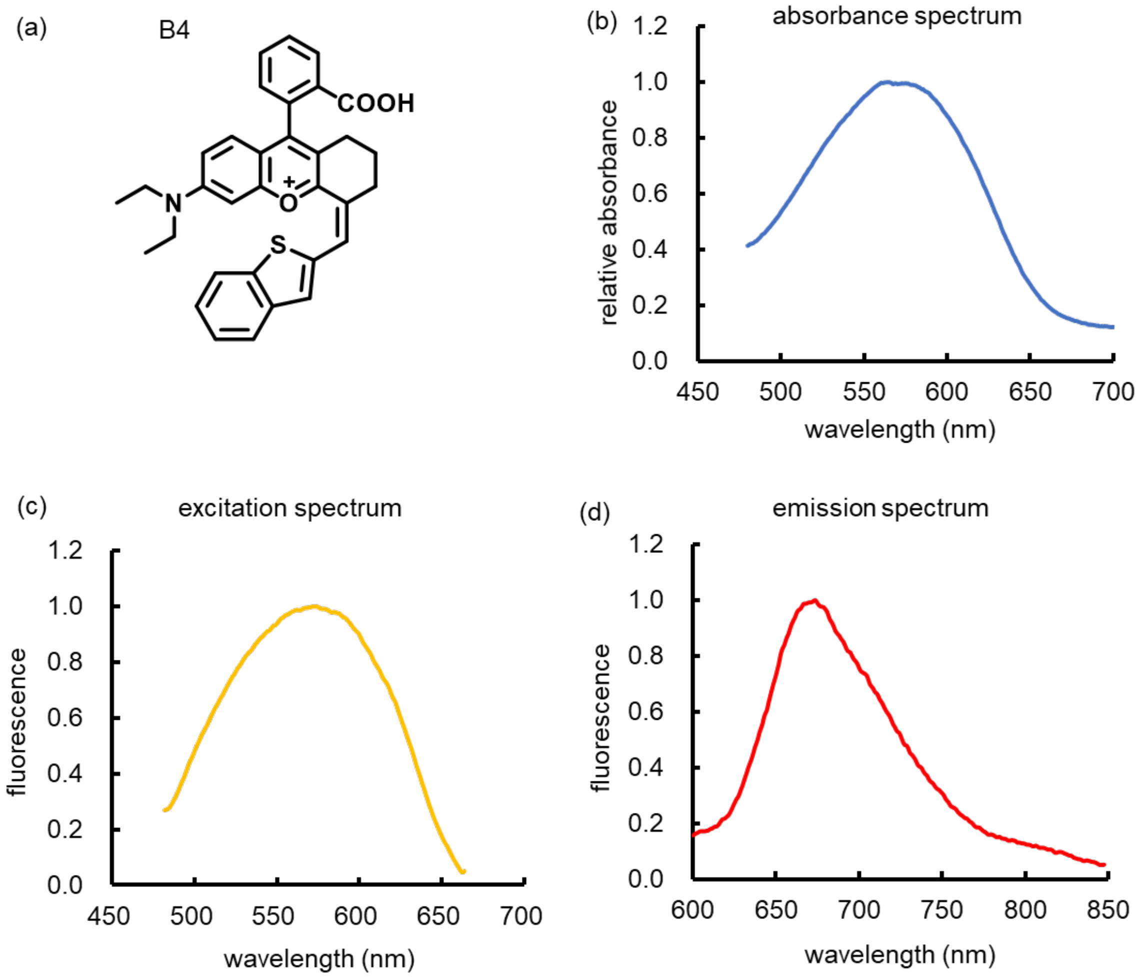


**Figure S16.** (a) Structure of B4. (b) Normalized absorbance spectrum. (c) Normalized excitation spectrum. (d) Normalized emission spectrum. All spectra were collected using 20 µM compound in 80% glycerol and 20% ethylene glycol.

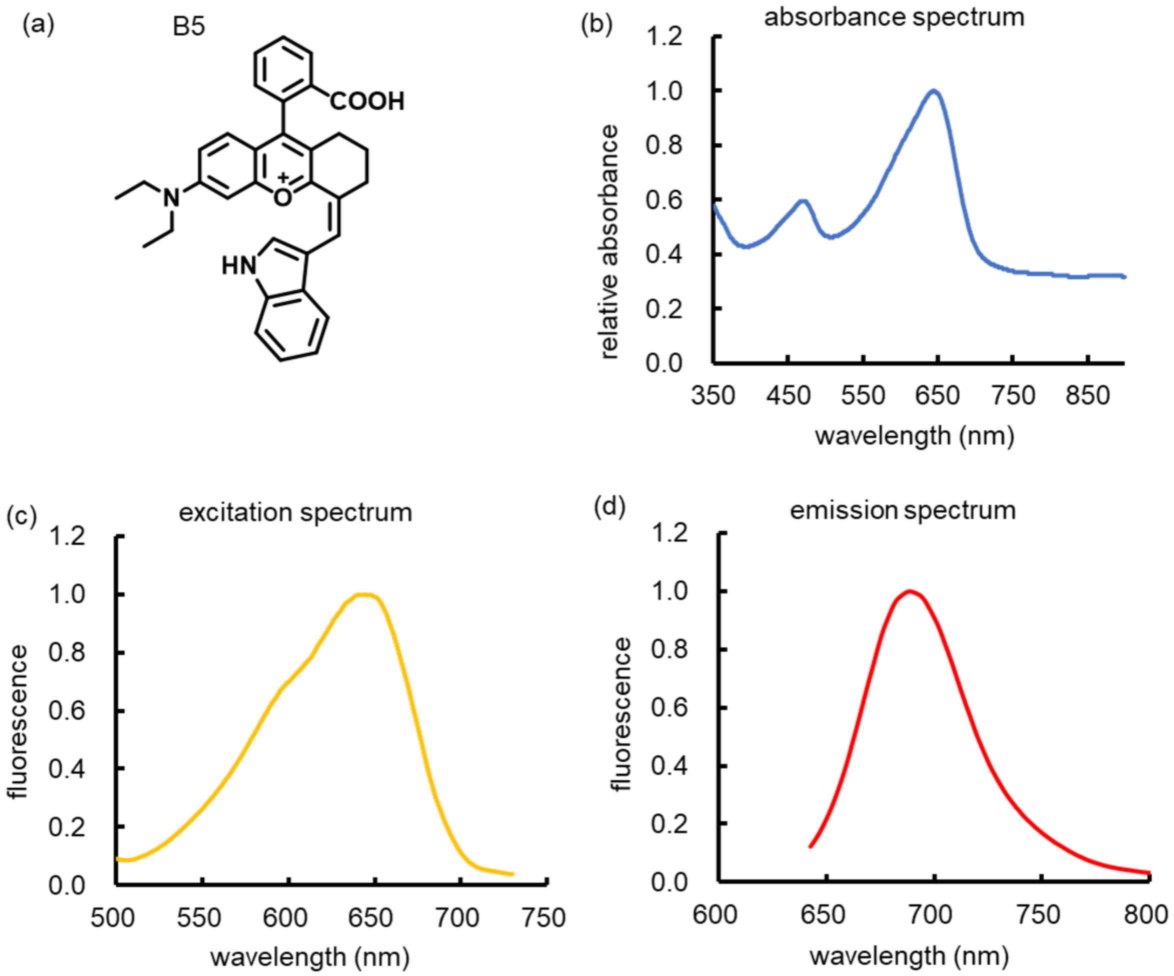


**Figure S17.** (a) Structure of B5. (b) Normalized absorbance spectrum. (c) Normalized excitation spectrum. (d) Normalized emission spectrum. All spectra were collected using 20 µM compound in 80% glycerol and 20% ethylene glycol.

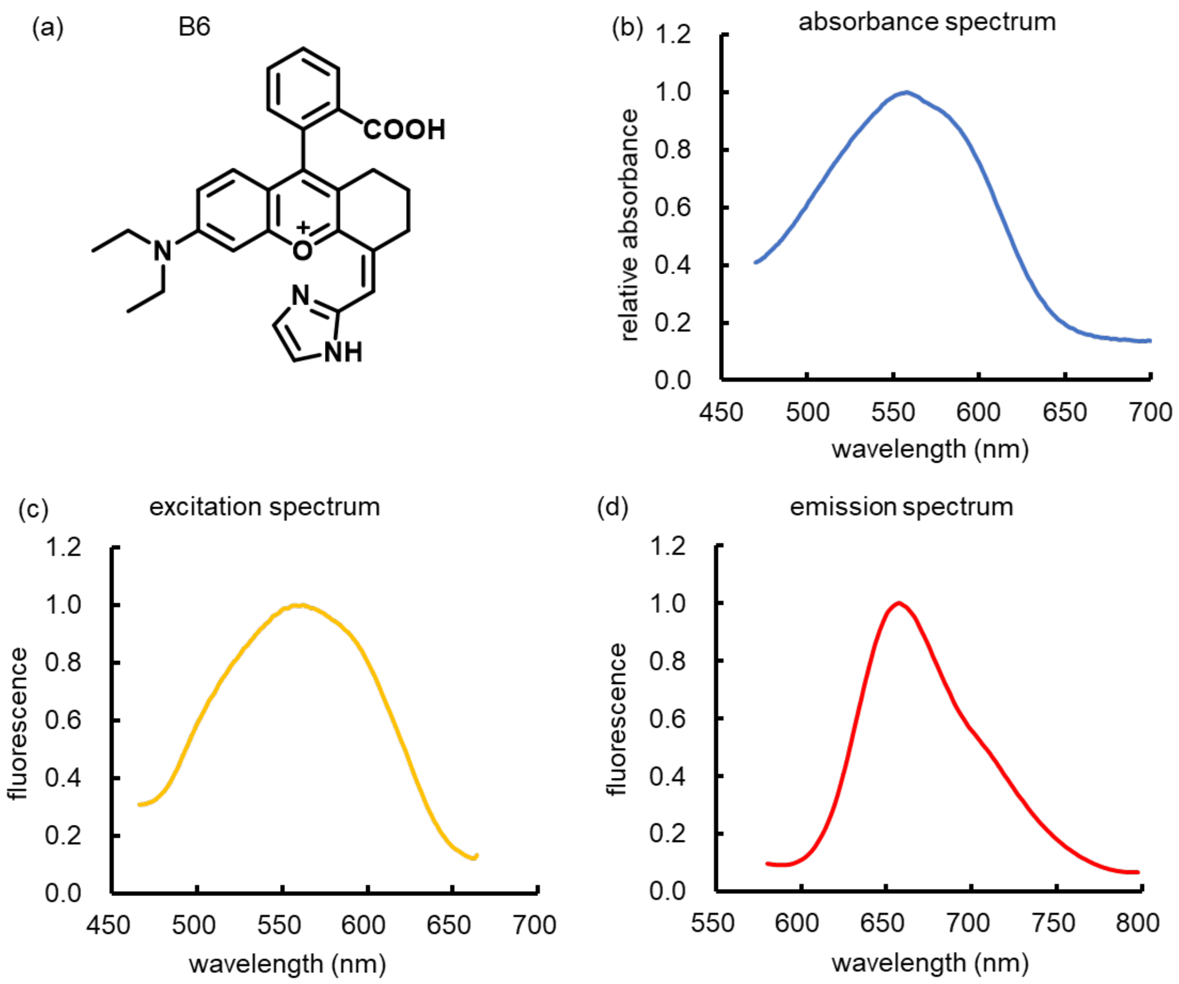


**Figure S18.** (a) Structure of B6. (b) Normalized absorbance spectrum. (c) Normalized excitation spectrum. (d) Normalized emission spectrum. All spectra were collected using 20 μM compound in 80% glycerol and 20% ethylene glycol.

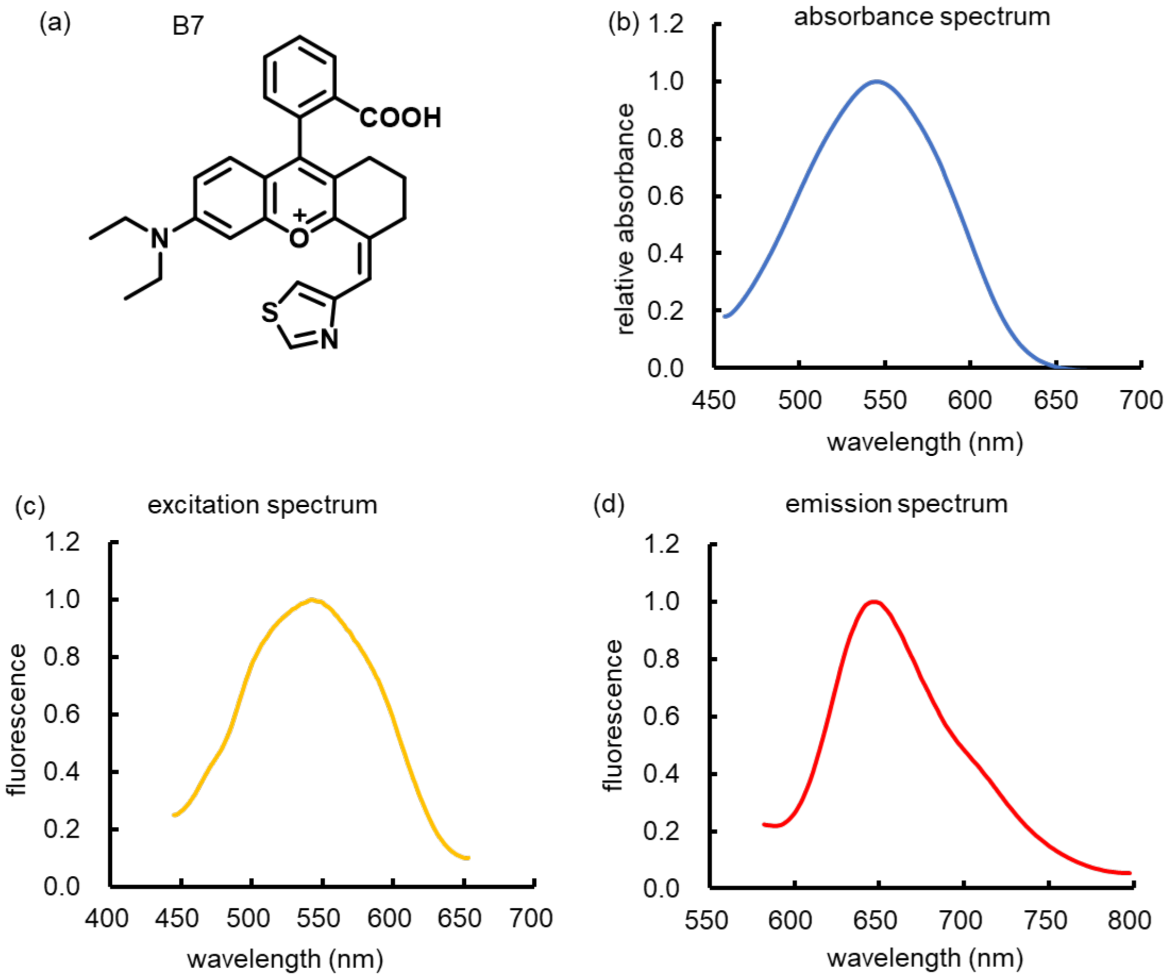


**Figure S19.** (a) Structure of B7. (b) Normalized absorbance spectrum. (c) Normalized excitation spectrum. (d) Normalized emission spectrum. All spectra were collected using 20 µM compound in 80% glycerol and 20% ethylene glycol.

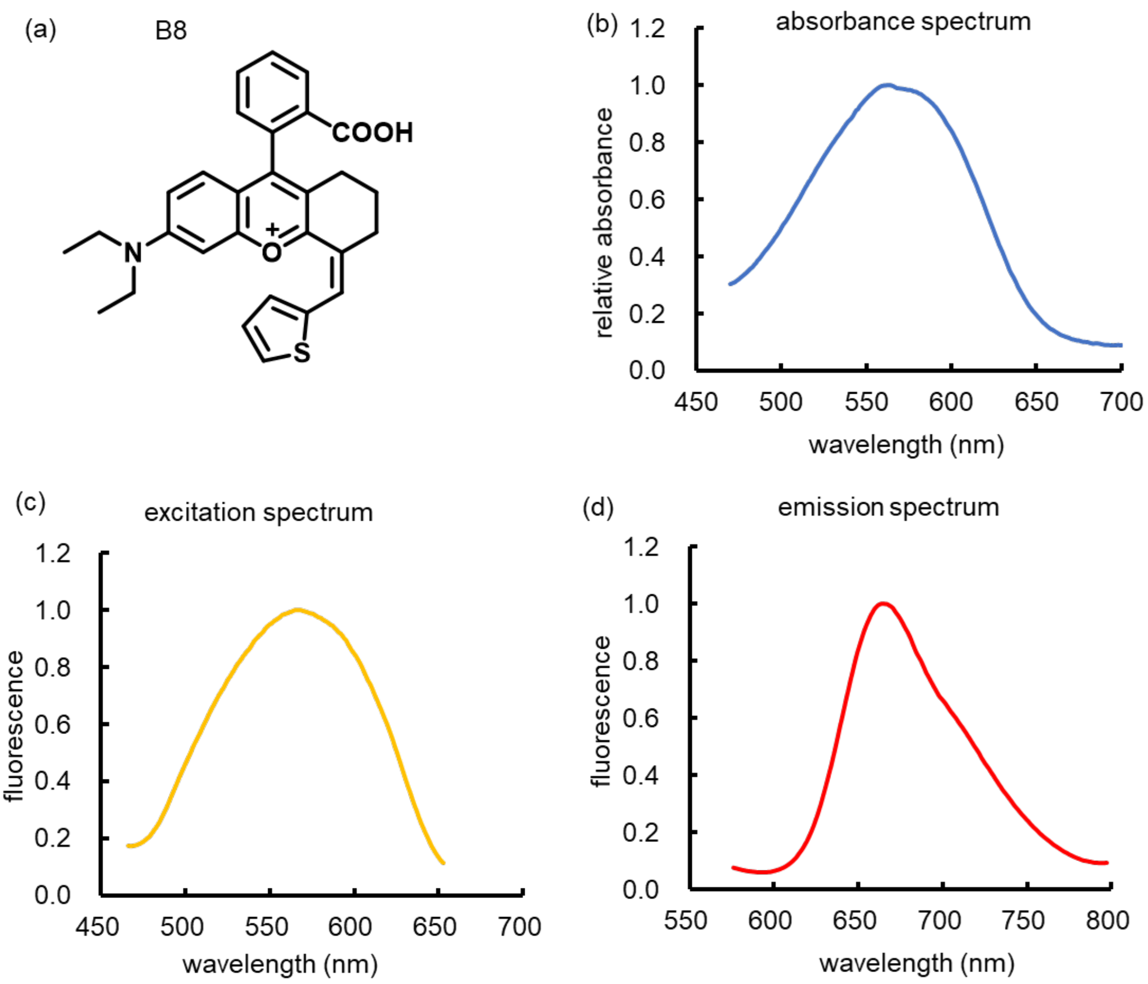


**Figure S20.** (a) Structure of B8. (b) Normalized absorbance spectrum. (c) Normalized excitation spectrum. (d) Normalized emission spectrum. All spectra were collected using 20 µM compound in 80% glycerol and 20% ethylene glycol.

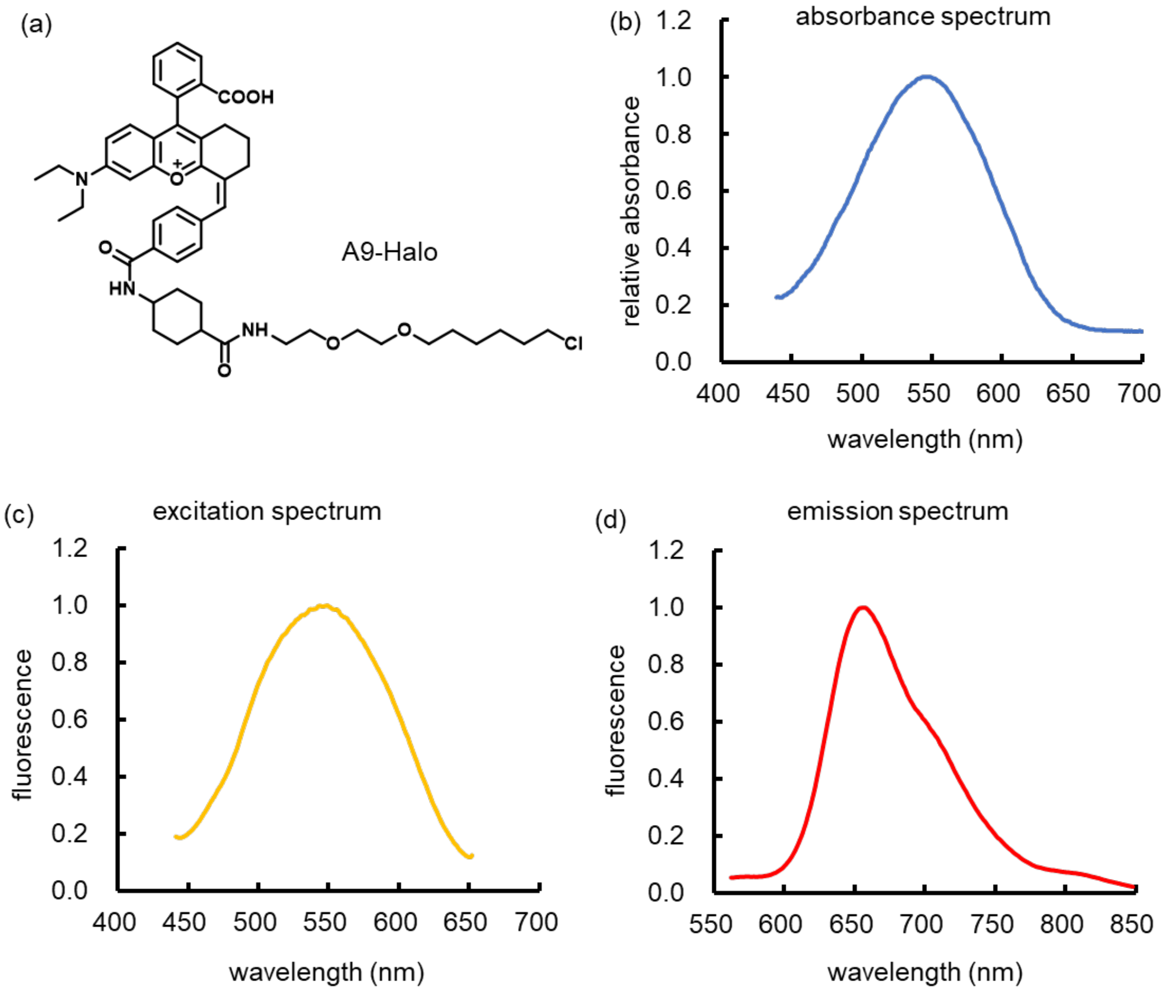


**Figure S21.** (a) Structure of A9-Halo. (b) Normalized absorbance spectrum. (c) Normalized excitation spectrum. (d) Normalized emission spectrum. All spectra were collected using 20 μM compound in 80% glycerol and 20% ethylene glycol.

### 3.2 Aggregation induced luminescence effect in water and tetrahydrofuran systems

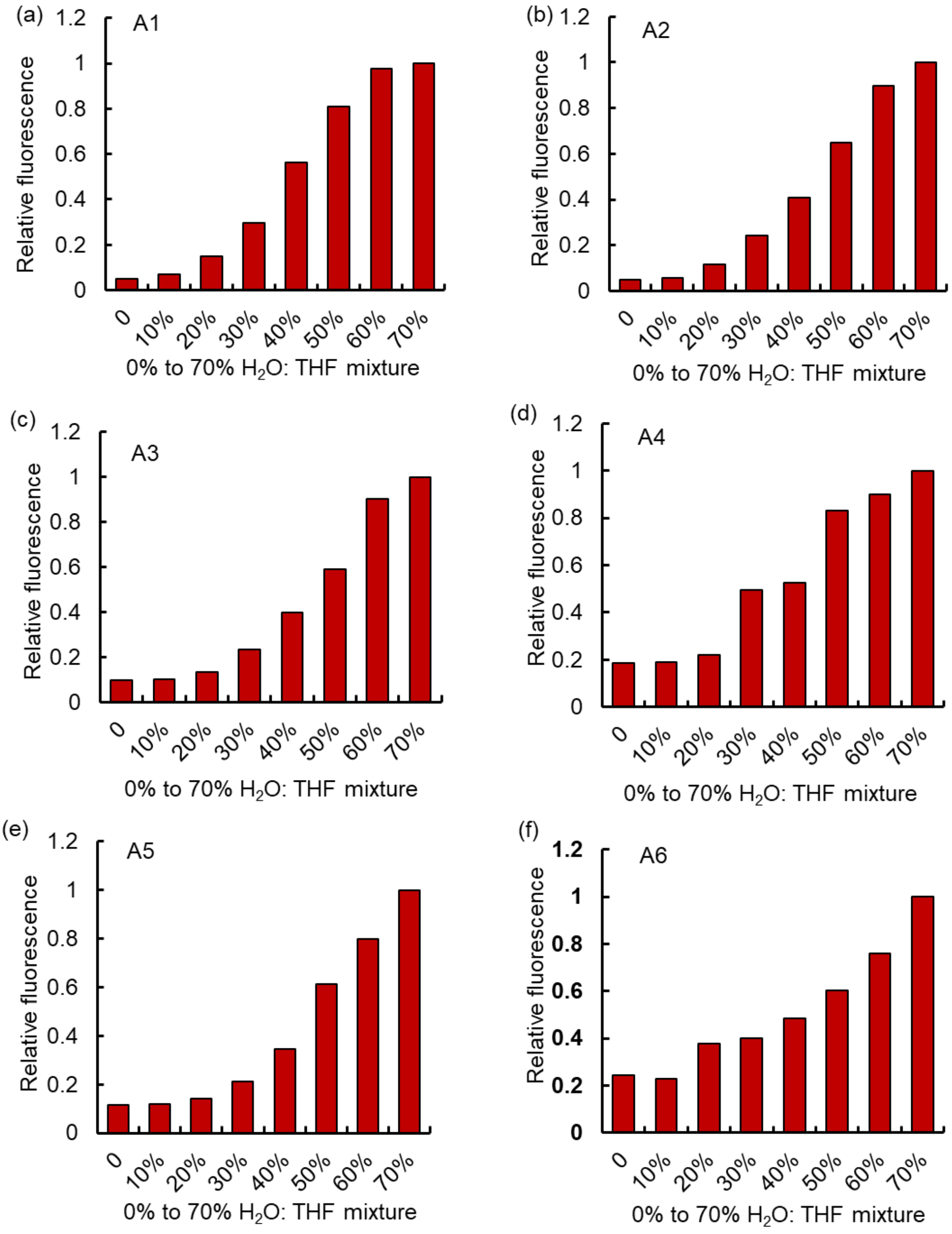


**Figure S22:** Molecular fluorescence response in mixed solvents with different contents of water and THF. 0% to 70% $H_2O$: THF mixture. Normalization was performed using the highest fluorescence intensity, respectively. (a) Fluorescence response of A1. (b) Fluorescence response of A2. (c) Fluorescence response of A3. (d) Fluorescence response of A4. (e) Fluorescence response of A5. (f) Fluorescence response of A6.

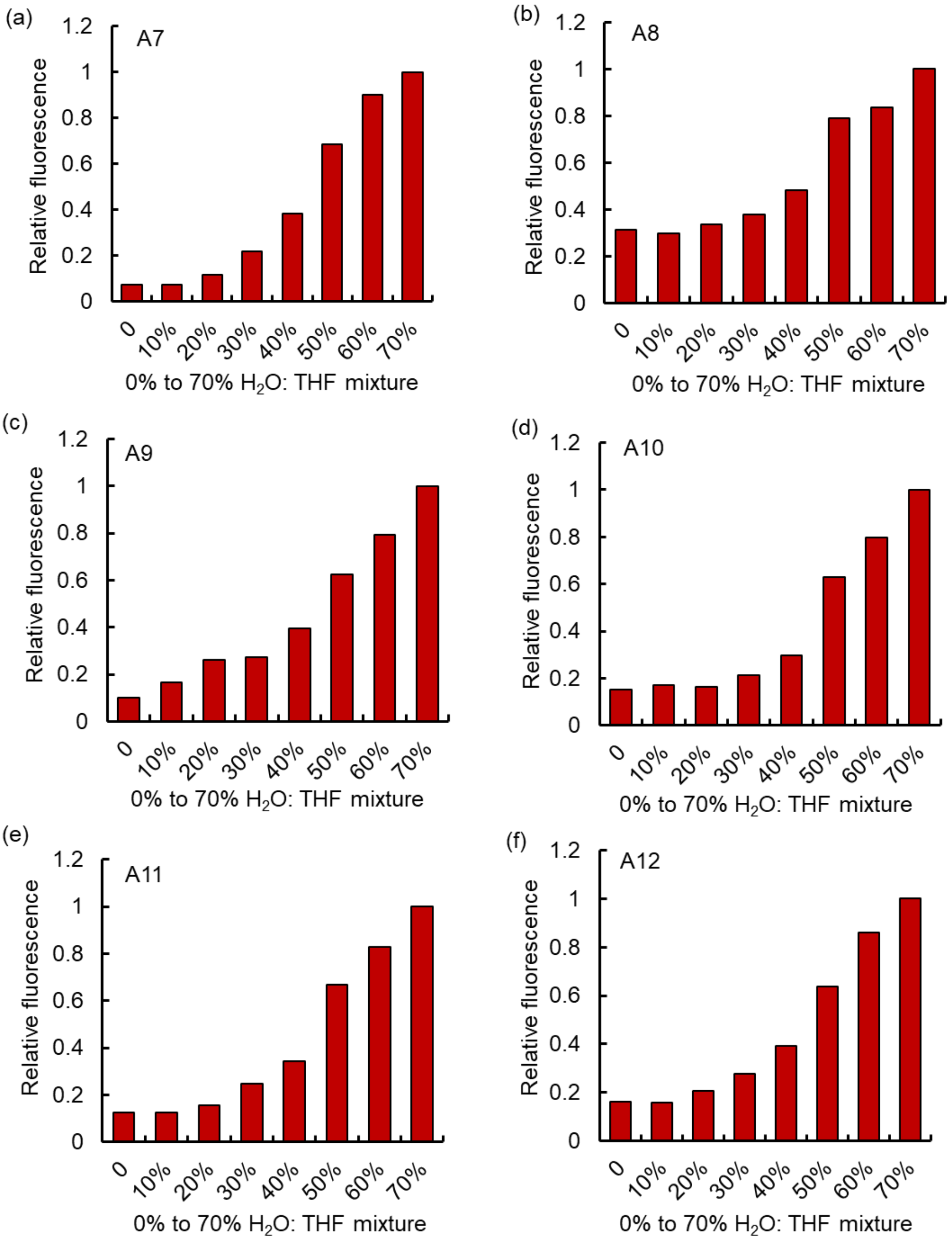


**Figure S23:** Molecular fluorescence response in mixed solvents with different contents of water and THF. 0% to 70% $H_2O$: THF mixture. Normalization was performed using the highest fluorescence intensity, respectively. (a) Fluorescence response of A7. (b) Fluorescence response of A8. (c) Fluorescence response of A9. (d) Fluorescence response of A10. (e) Fluorescence response of A11. (f) Fluorescence response of A12.

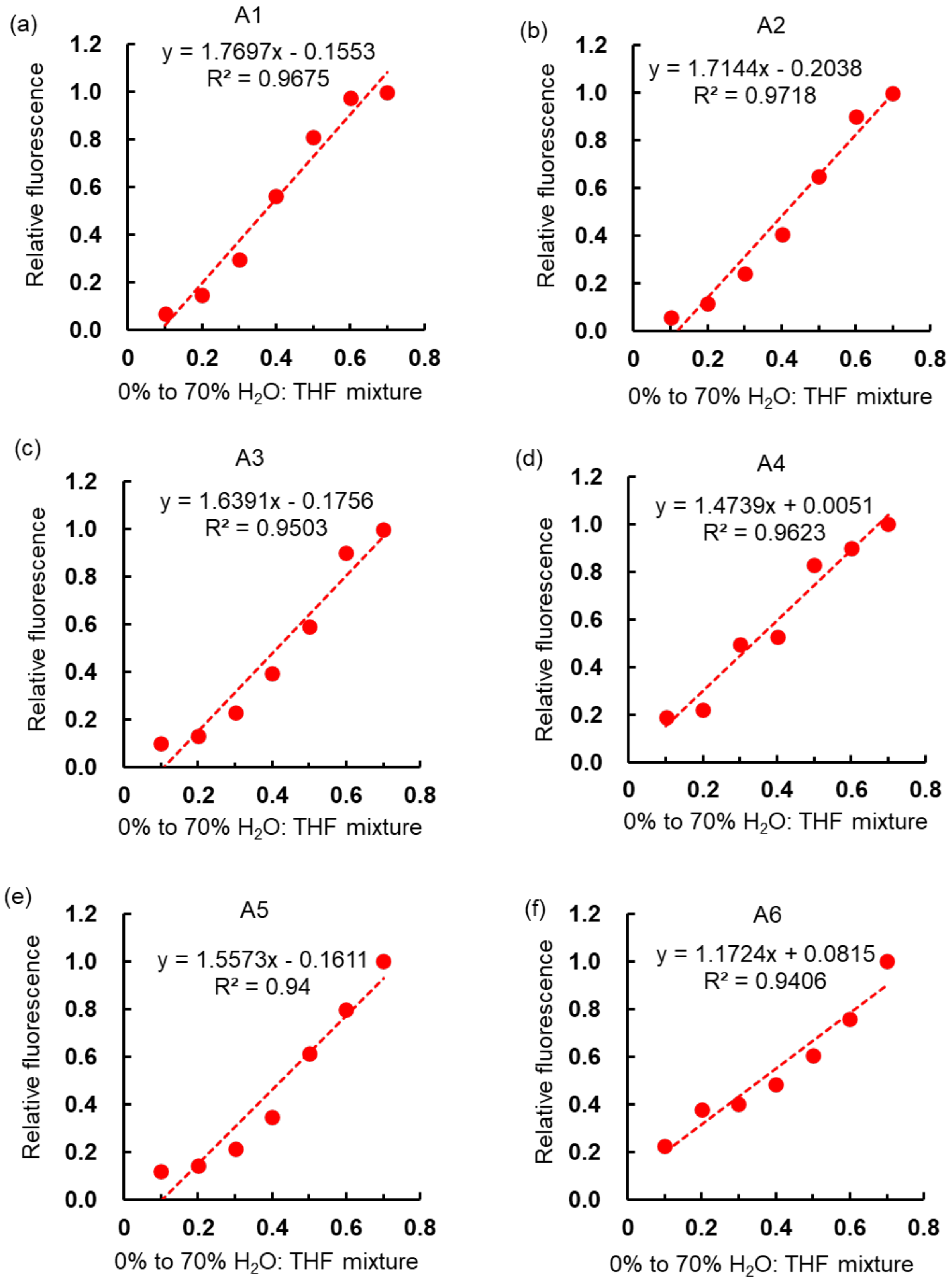


**Figure S24:** Molecular fluorescence response in mixed solvents with different contents of water and THF. 0% to 70% $H_2O$: THF mixture. The highest fluorescence intensity was used for normalization and linear fitting. (a) A1. (b) A2. (c) A3. (d) A4. (e) A5. (f) A6.

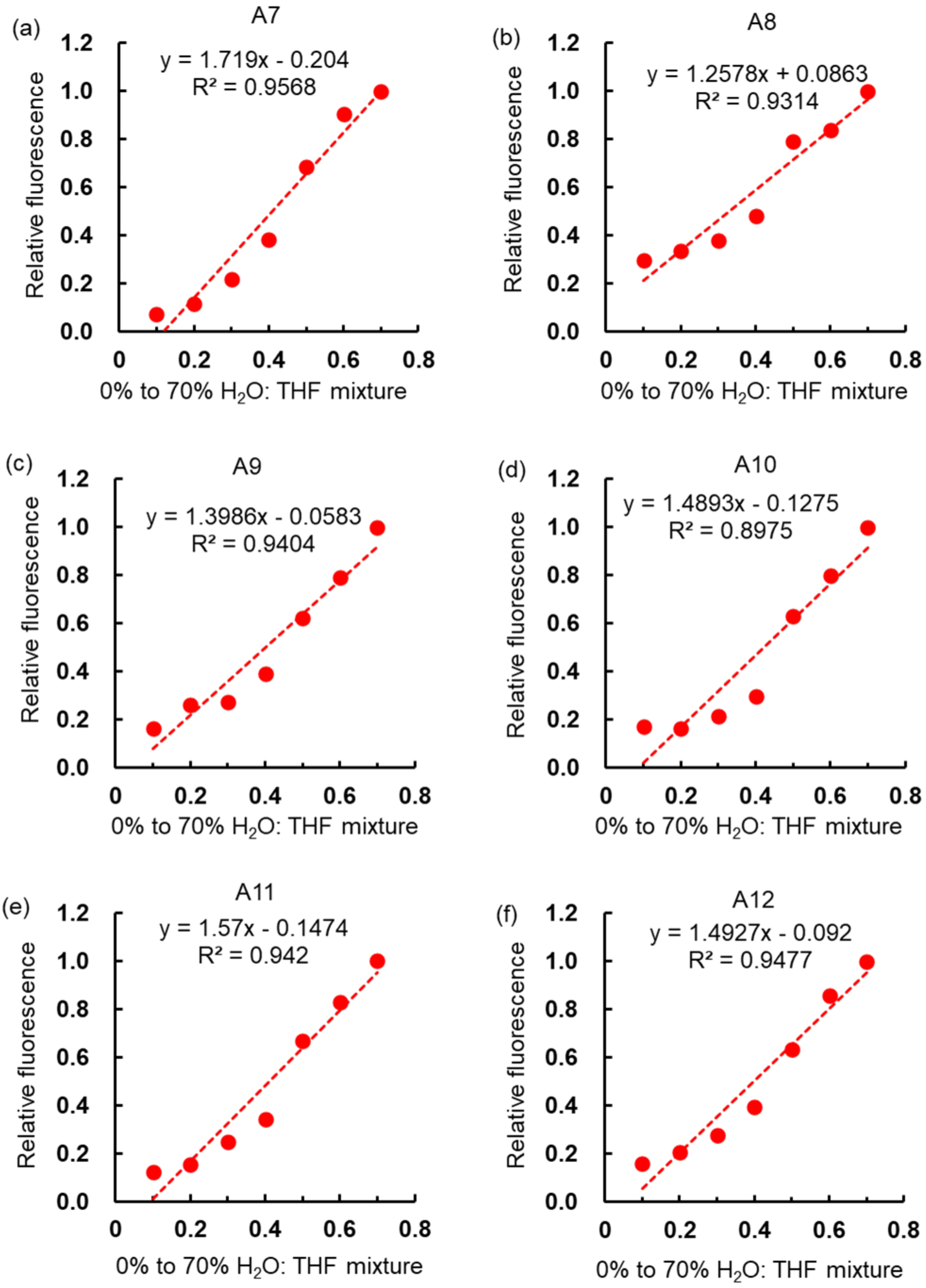


**Figure S25:** Molecular fluorescence response in mixed solvents with different contents of water and THF. 0% to 70% $H_2O$: THF mixture. The highest fluorescence intensity was used for normalization and linear fitting. (a) A7. (b) A8. (c) A9. (d) A10. (e) A11. (f) A12.

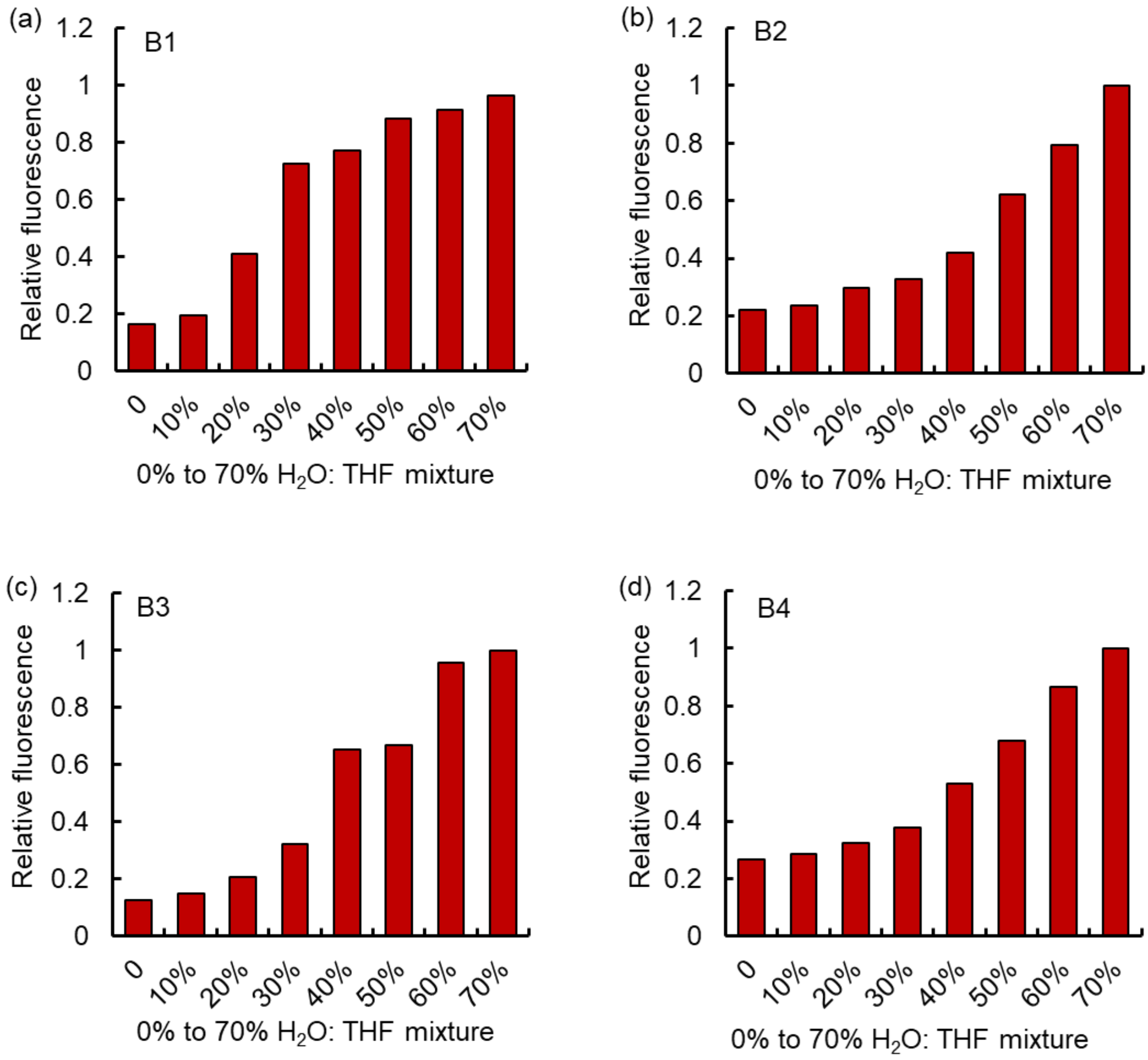


**Figure S26:** Molecular fluorescence response in mixed solvents with different contents of water and THF. 0% to 70% $H_2O$: THF mixture. Normalization was performed using the highest fluorescence intensity, respectively. (a) Fluorescence response of B1. (b) Fluorescence response of B2. (c) Fluorescence response of B3. (d) Fluorescence response of B4.

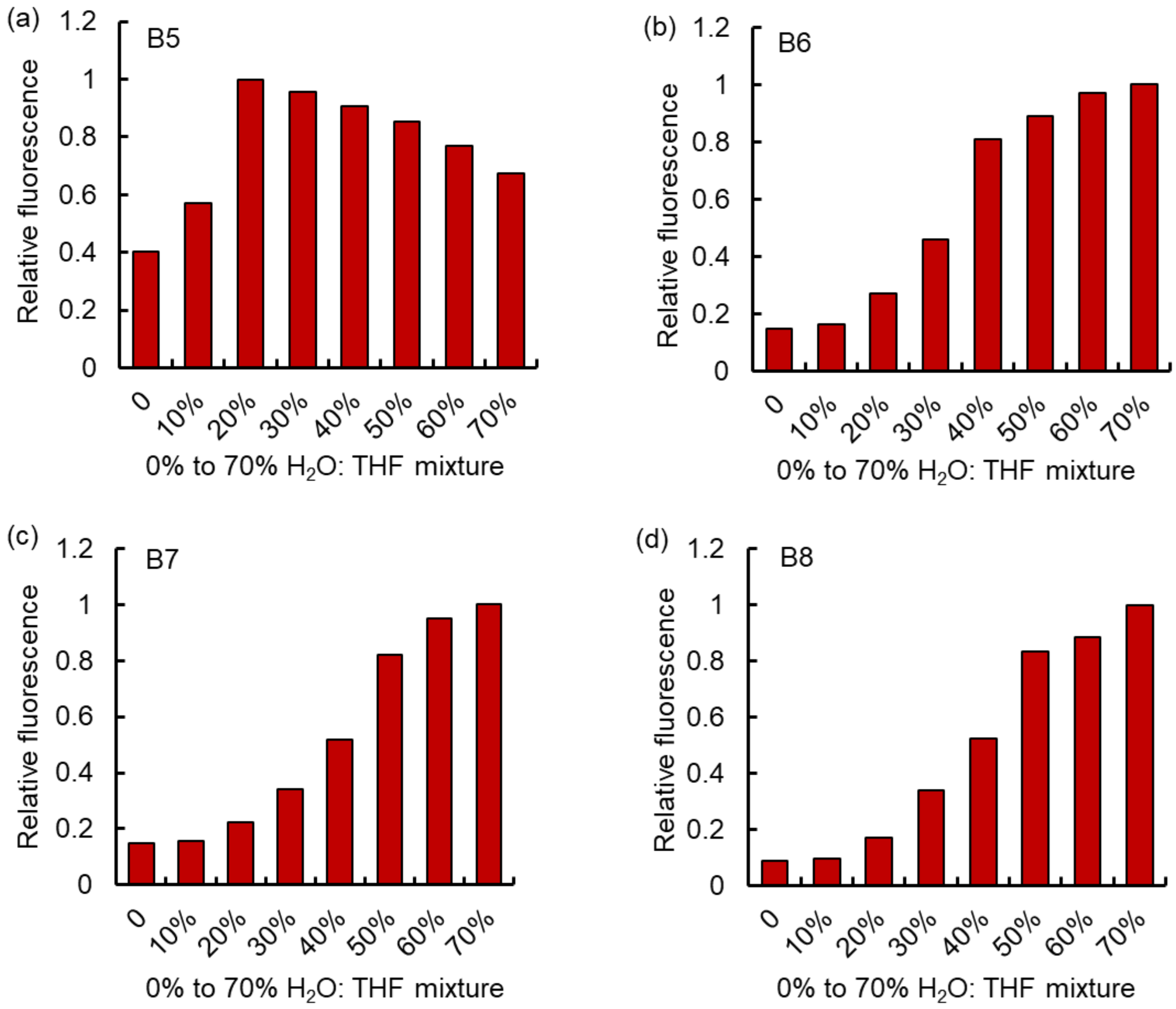


**Figure S27:** Molecular fluorescence response in mixed solvents with different contents of water and THF. 0% to 70% $H_2O$: THF mixture. Normalization was performed using the highest fluorescence intensity, respectively. (a) Fluorescence response of B5. (b) Fluorescence response of B6. (c) Fluorescence response of B7. (d) Fluorescence response of B8.

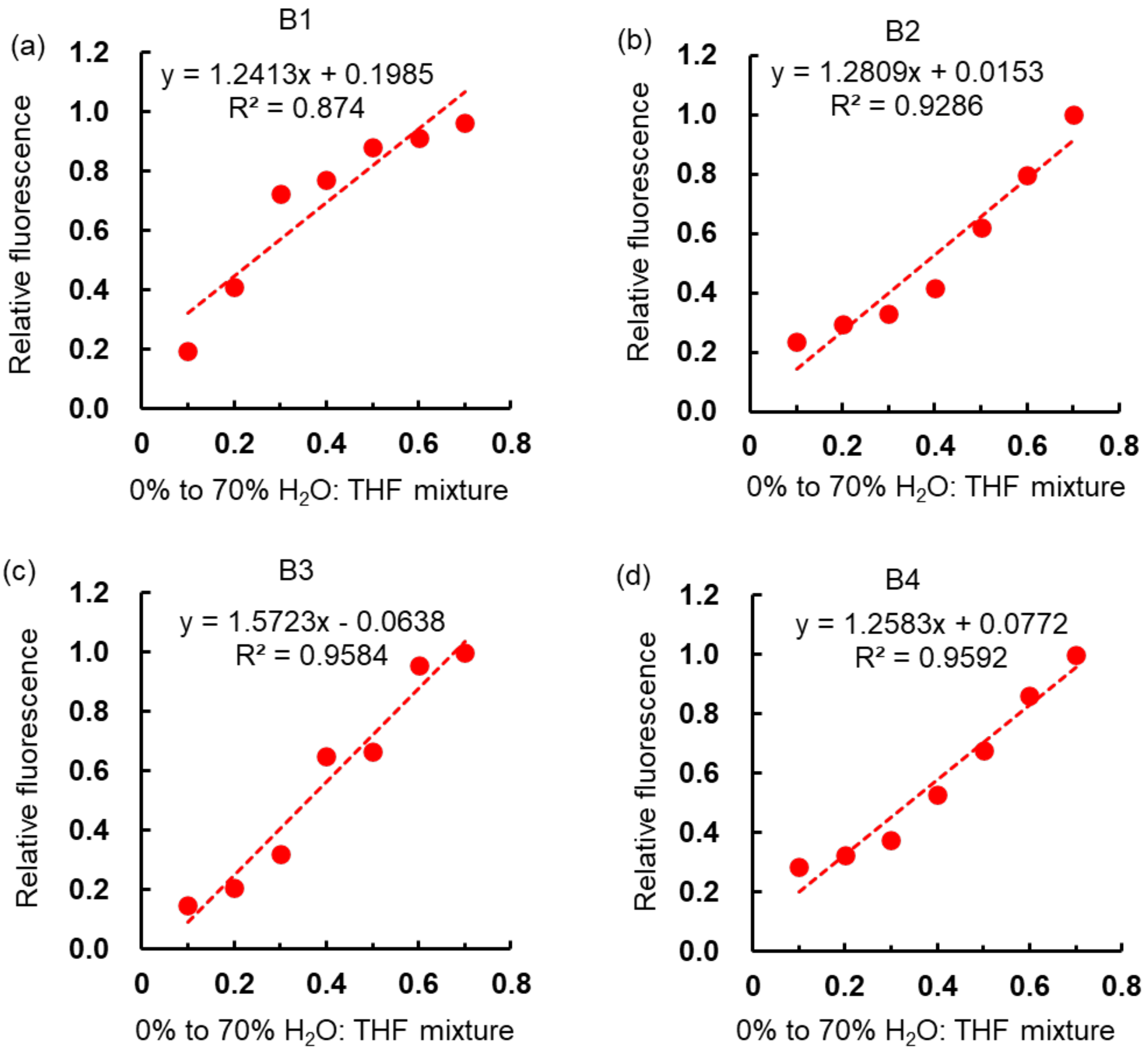


**Figure S28:** Molecular fluorescence response in mixed solvents with different contents of water and THF. 0% to 70% $H_2O$: THF mixture. The highest fluorescence intensity was used for normalization and linear fitting. (a) B1. (b) B2. (c) B3. (d) B4.

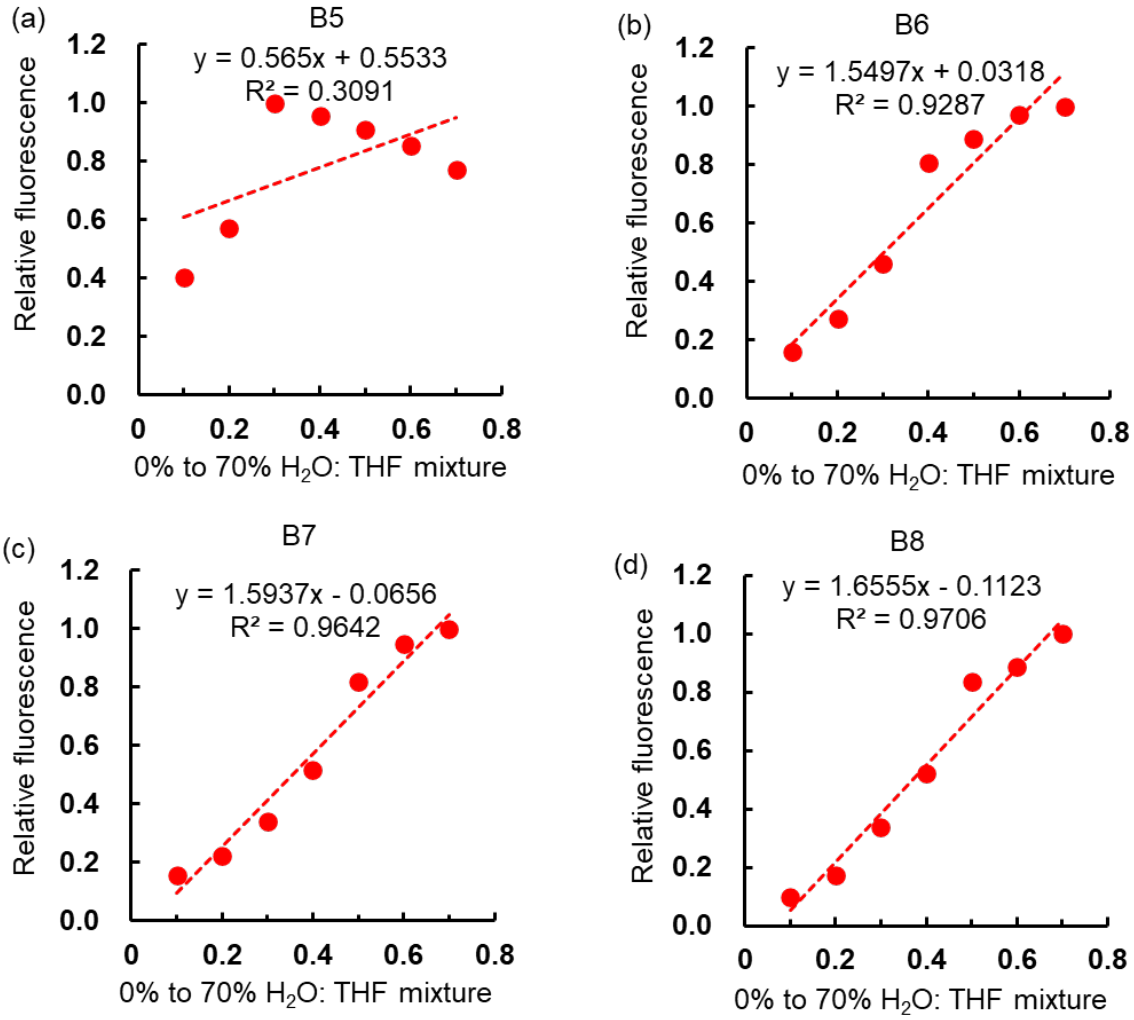


**Figure S29:** Molecular fluorescence response in mixed solvents with different contents of water and THF. 0% to 70% $H_2O$: THF mixture. The highest fluorescence intensity was used for normalization and linear fitting. (a) B5. (b) B6. (c) B7. (d) B8.

### 3.3 Viscosity Sensitivity

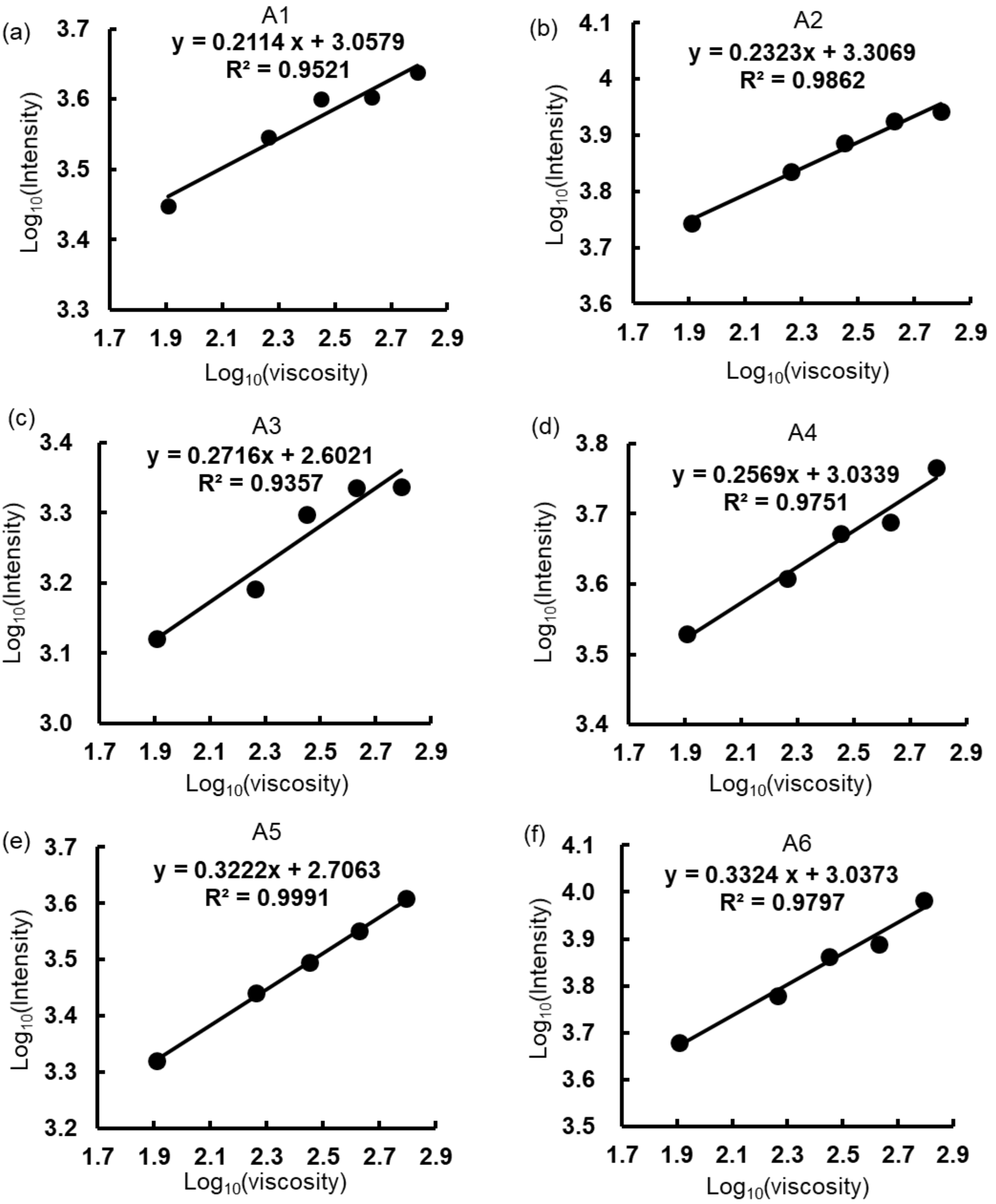


**Figure S30.** Logarithmic plot of fluorescent intensity as a function of solvent viscosity for A1-A6. (a) A1, (b) A2, (c) A3, (d) A4, (e) A5, (f) A6. Fluorescence measurement was carries out using 10 µM compounds in a series of ethylene glycol/glycerol (EG/G) solutions in the following mixing ratios: EG/G = 70/30 (81 cP), 50/50 (183 cP), 40/60 (283 cP), 30/70 (426 cP), 20/80 (621 cP).

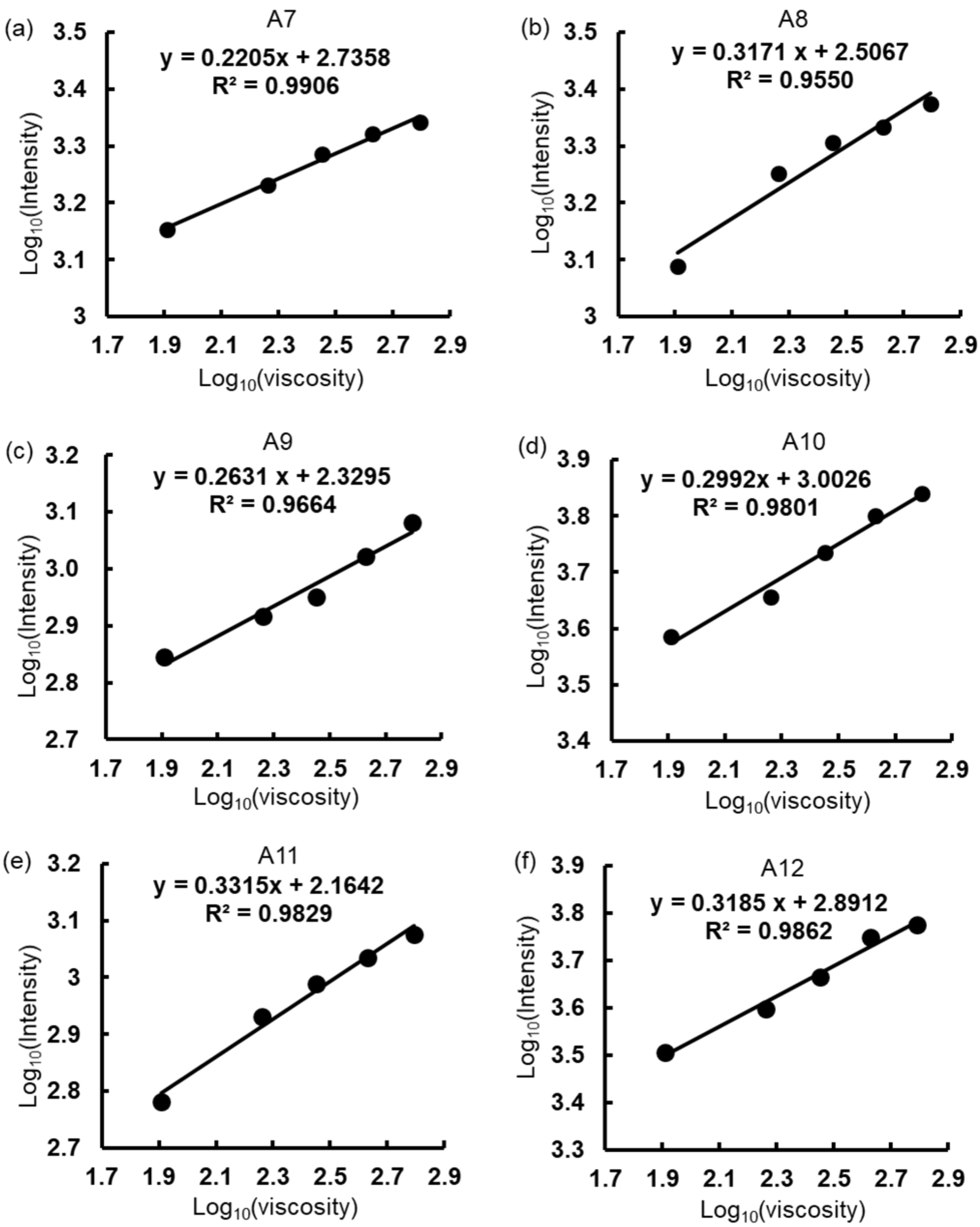


**Figure S31**. Logarithmic plot of fluorescent intensity as a function of solvent viscosity for A7-A12. (a) A7, (b) A8, (c) A9, (d) A10, (e) A11, (f) A12. Fluorescence measurement was carries out using 10 μM compounds in a series of ethylene glycol/glycerol (EG/G) solutions in the following mixing ratios: EG/G = 70/30 (81 cP), 50/50 (183 cP), 40/60 (283 cP), 30/70 (426 cP), 20/80 (621 cP).

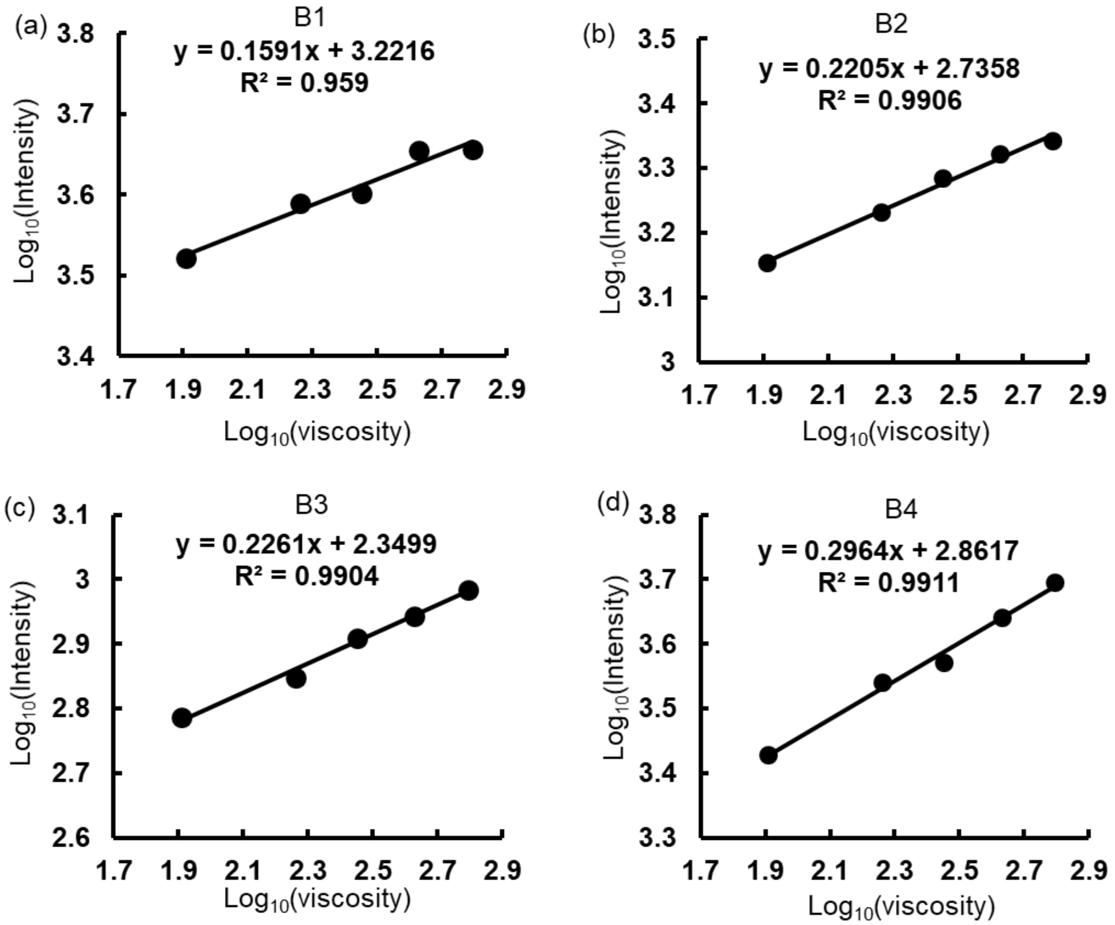


**Figure S32**. Logarithmic plot of fluorescent intensity as a function of solvent viscosity for B1-B4. (a) B1, (b) B2, (c) B3, (d) B4. Fluorescence measurement was carries out using 10 μM compounds in a series of ethylene glycol/glycerol (EG/G) solutions in the following mixing ratios: EG/G = 70/30 (81 cP), 50/50 (183 cP), 40/60 (283 cP), 30/70 (426 cP), 20/80 (621 cP).

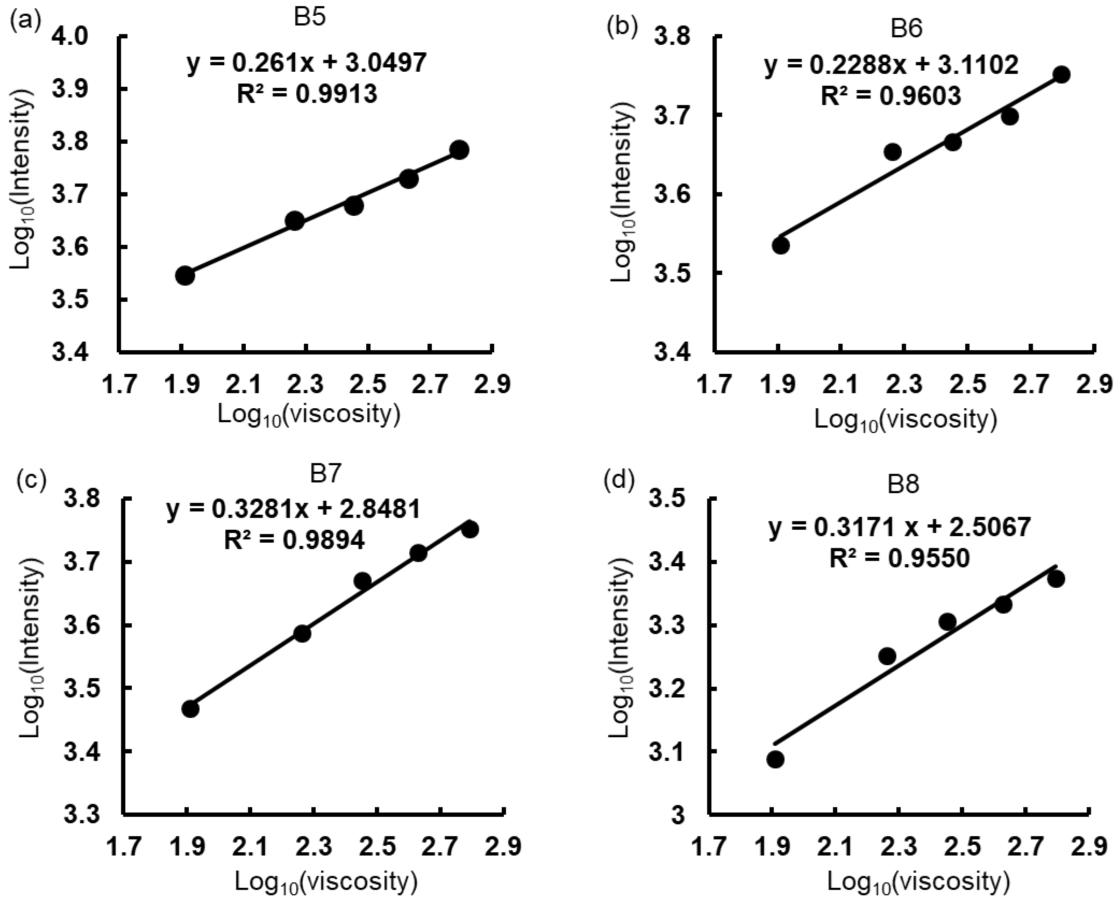


**Figure 33**. Logarithmic plot of fluorescent intensity as a function of solvent viscosity for B5-B8. (a) B5, (b) B6, (c) B7, (d) B8. Fluorescence measurement was carries out using 10 μM compounds in a series of ethylene glycol/glycerol (EG/G) solutions in the following mixing ratios: EG/G = 70/30 (81 cP), 50/50 (183 cP), 40/60 (283 cP), 30/70 (426 cP), 20/80 (621 cP).

### 3.4 Theoretical calculation of $S_0$ and $S_1$ state.

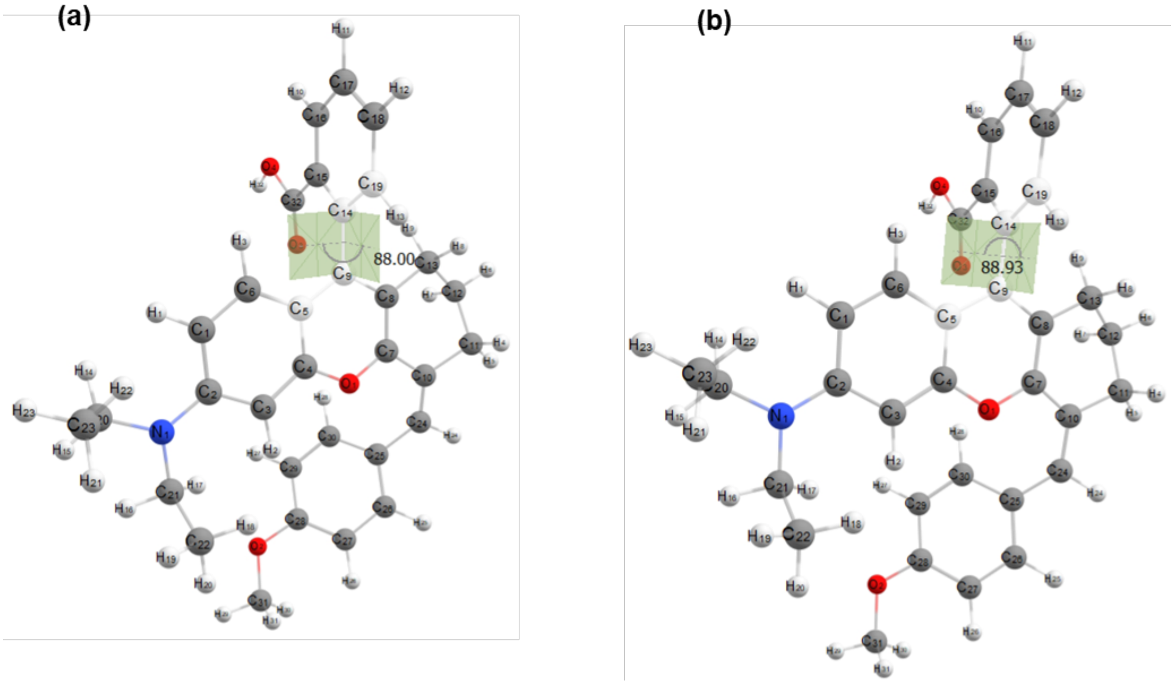


**Figure S34.** The rotation of I bond for A1. (a) The dihedral angle of I bond in the $S_0$ state. (b) The dihedral angle of I bond in the $S_1$ state. There is no significant difference in the dihedral angles of the I bond of A1 between $S_0$ and $S_1$.

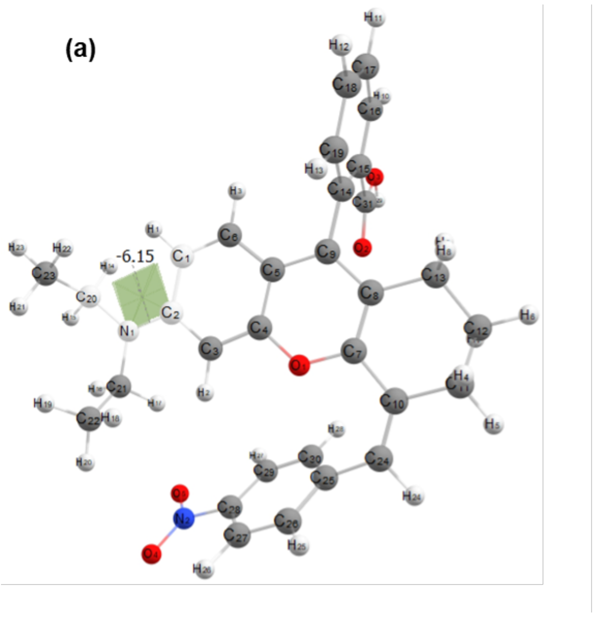


**Figure S35.** The rotation of II bond for A1. (a) The dihedral angle of II bond in the $S_0$ state. (b) The dihedral angle of II bond in the $S_1$ state. There is no significant difference in the dihedral angles of the I bond of A1 between $S_0$ and $S_1$.

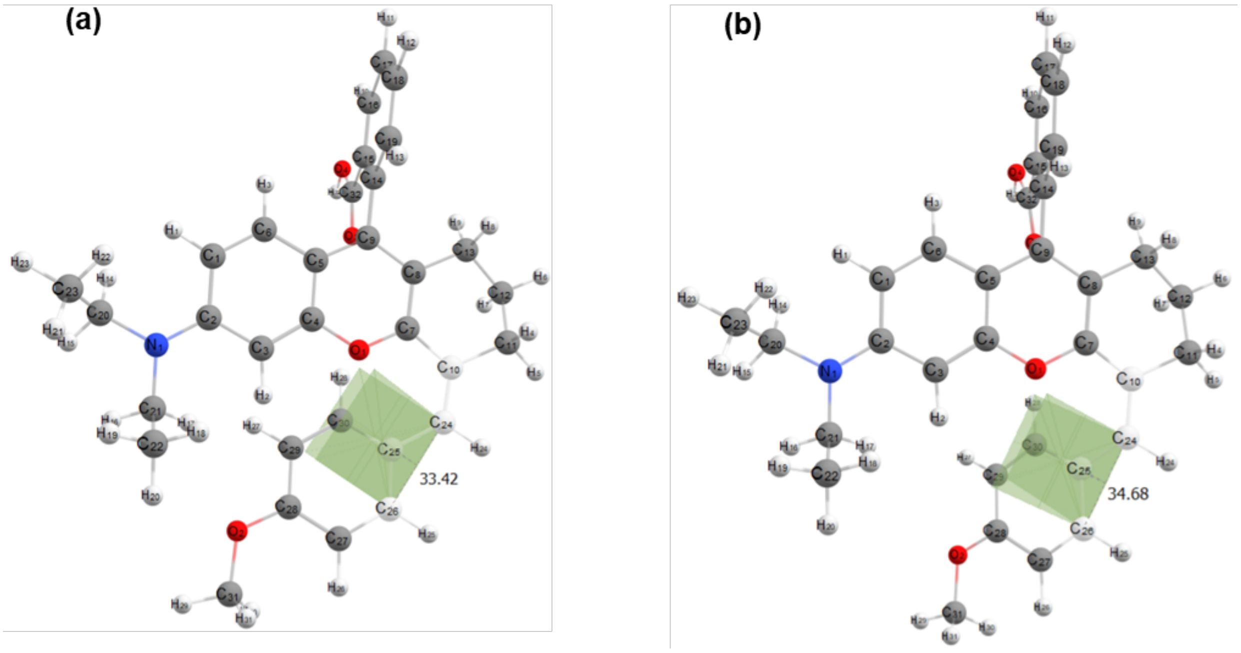


**Figure S36.** The rotation of III bond for A1. (a) The dihedral angle of III bond in the $S_0$ state. (b) The dihedral angle of III bond in the $S_1$ state. There is no significant difference in the dihedral angles of the I bond of A1 between $S_0$ and $S_1$.

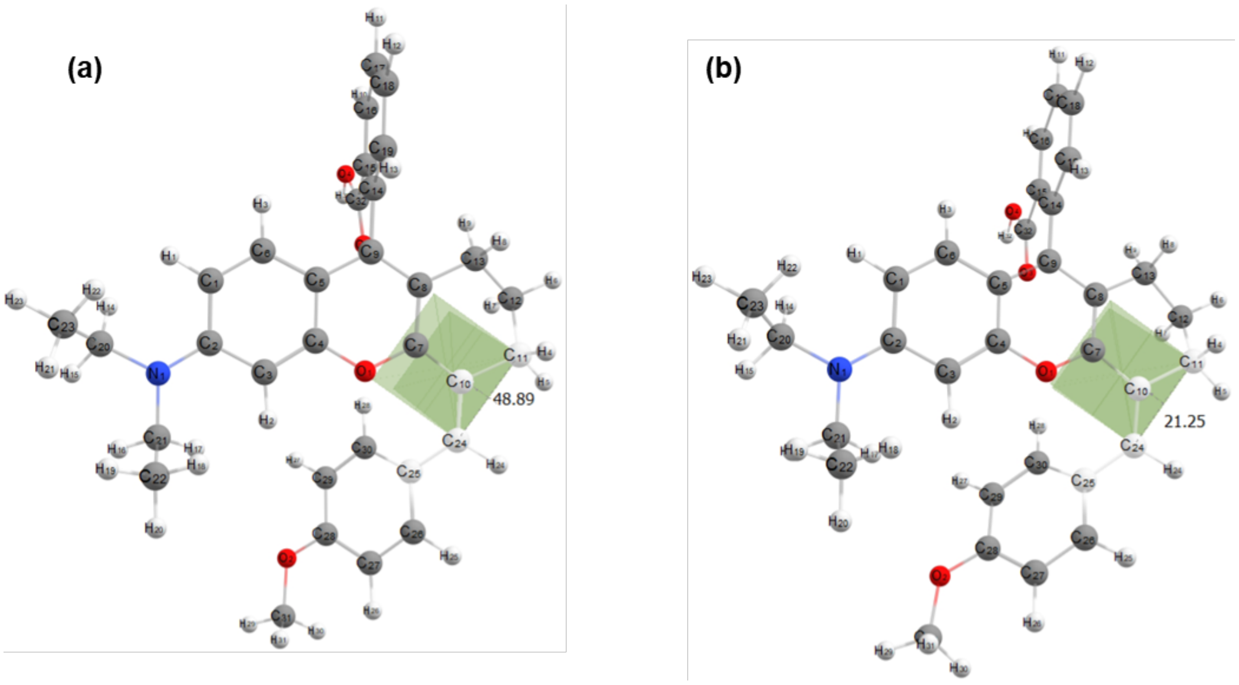


**Figure S37.** The rotation of IV bond for A1. (a) The dihedral angle of I bond in the $S_0$ state. (b) The dihedral angle of I bond in the $S_1$ state. There is a significant difference in the dihedral angles of the I bond of A1 between $S_0$ and $S_1$ (48.89° ~ 21.25°).

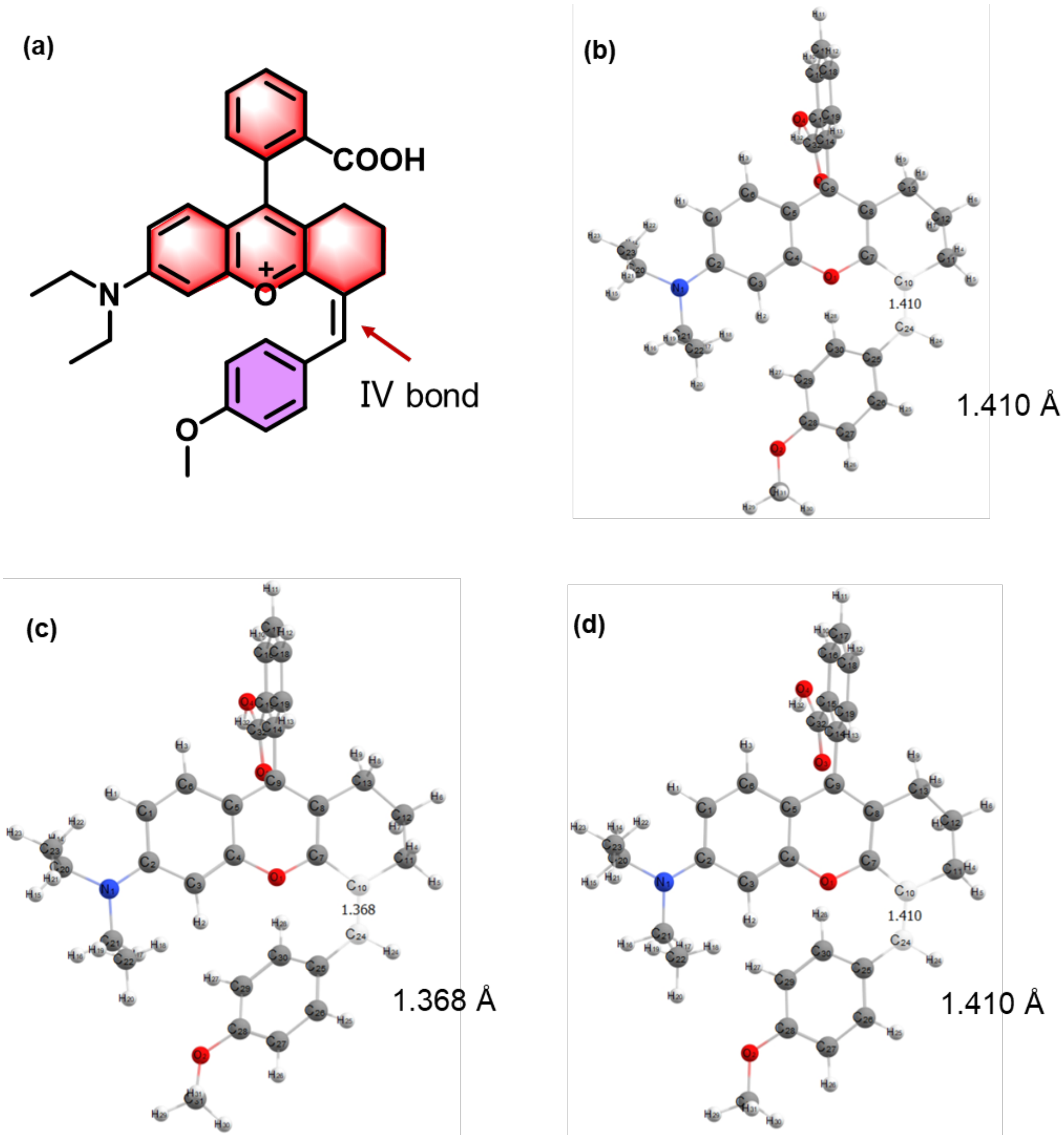


**Figure S38.** (a) The rotation of IV bond for A1. (b) The IV bond length of the A1 structure in the CI-state. (c) The IV bond length of the A1 structure in the $S_0$-state. (d) The IV bond length of the A1 structure in the $S_1$-state.

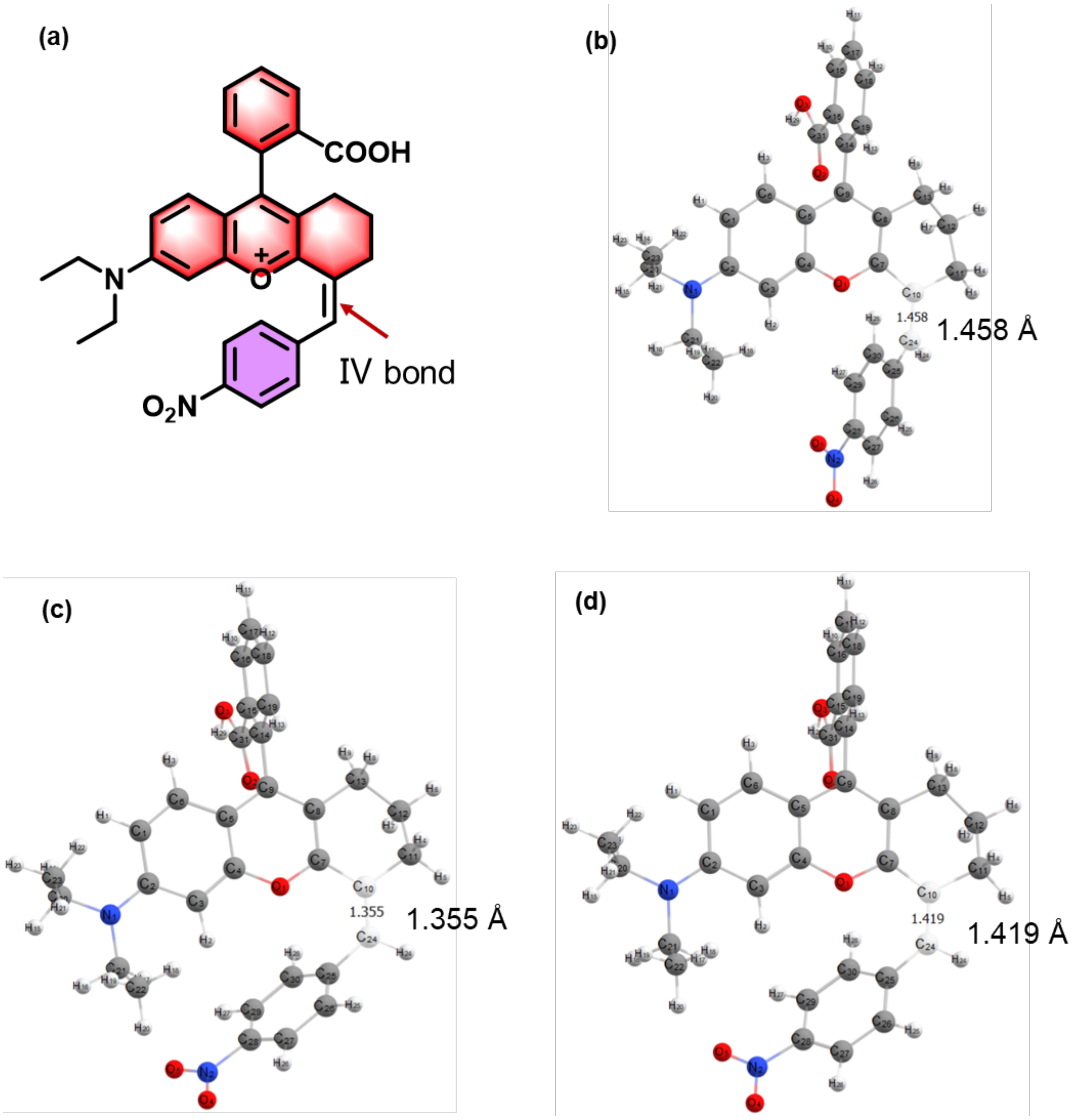


**Figure S39.** (a) The rotation of IV bond for A11. (b) The IV bond length of the A11 structure in the CI-state. (c) The IV bond length of the A11 structure in the $S_0$-state. (d) The IV bond length of the A11 structure in the $S_1$-state.

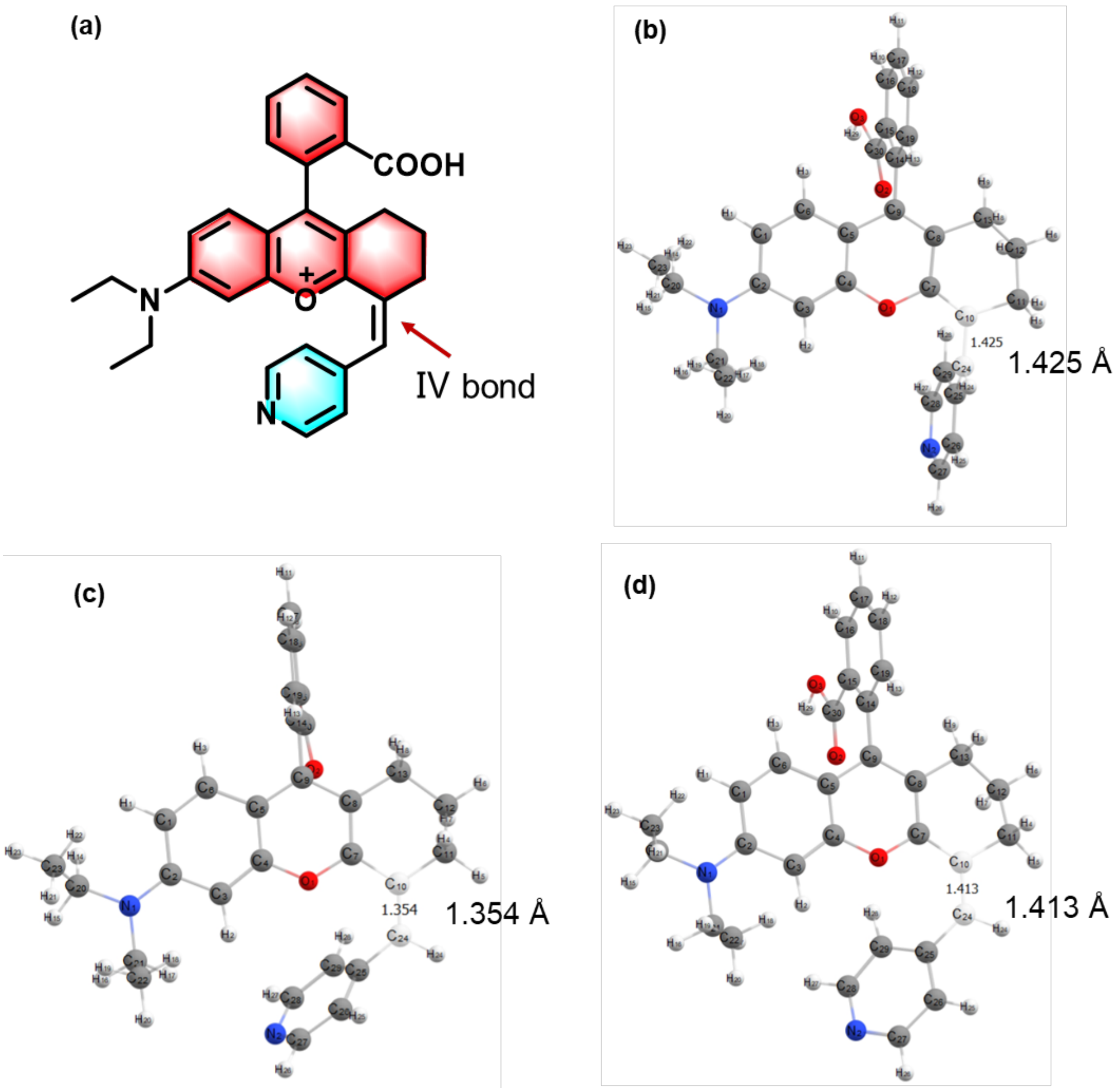


**Figure S40.** (a) The rotation of IV bond for B1. (b) The IV bond length of the B1 structure in the CI-state. (c) The IV bond length of the B1 structure in the $S_0$-state. (d) The IV bond length of the B1 structure in the $S_1$-state.

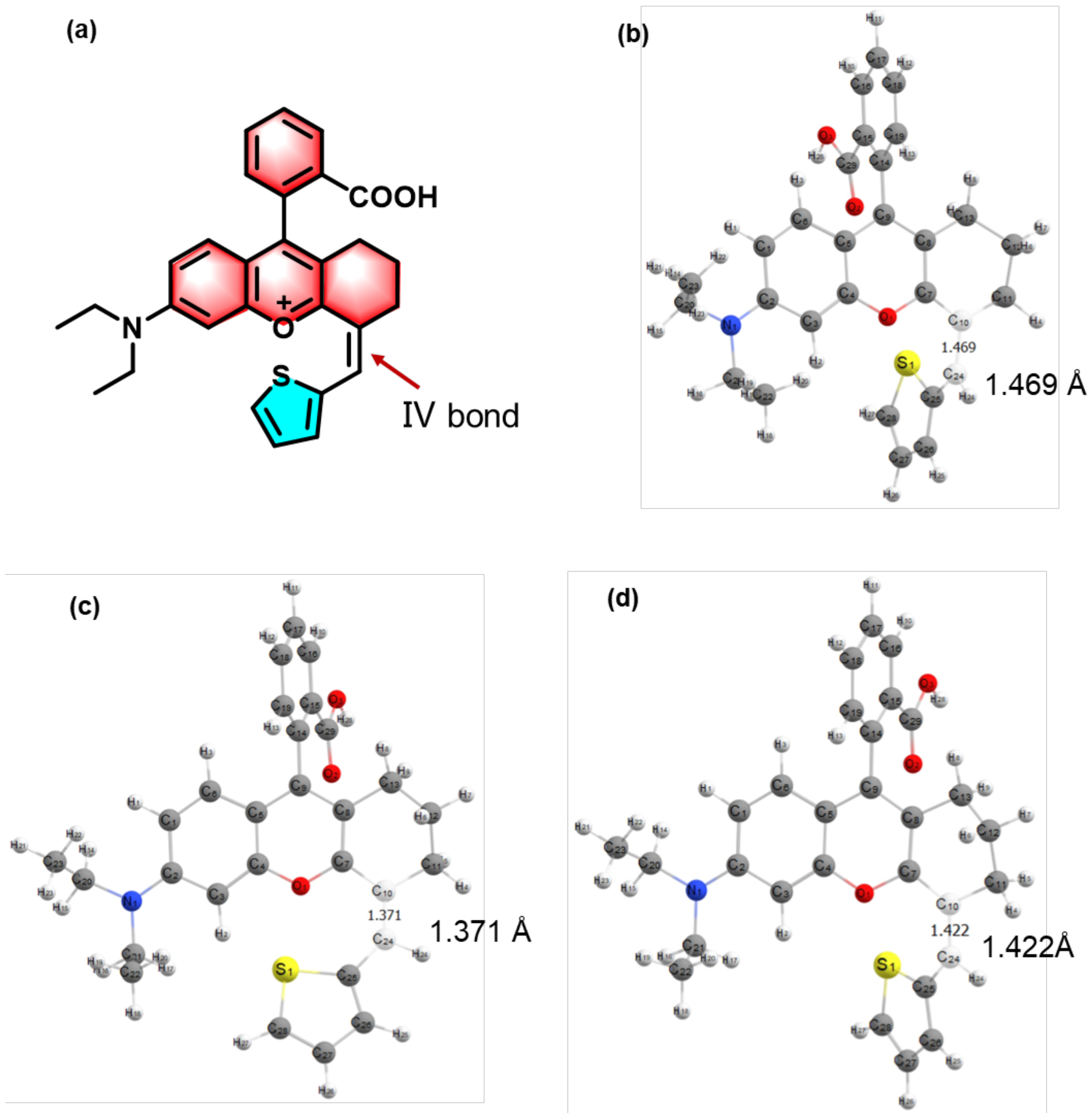


**Figure S41.** (a) The rotation of IV bond for B8. (b) The IV bond length of the B8 structure in the CI-state. (c) The IV bond length of the B8 structure in the $S_0$-state. (d) The IV bond length of the B8 structure in the $S_1$-state.

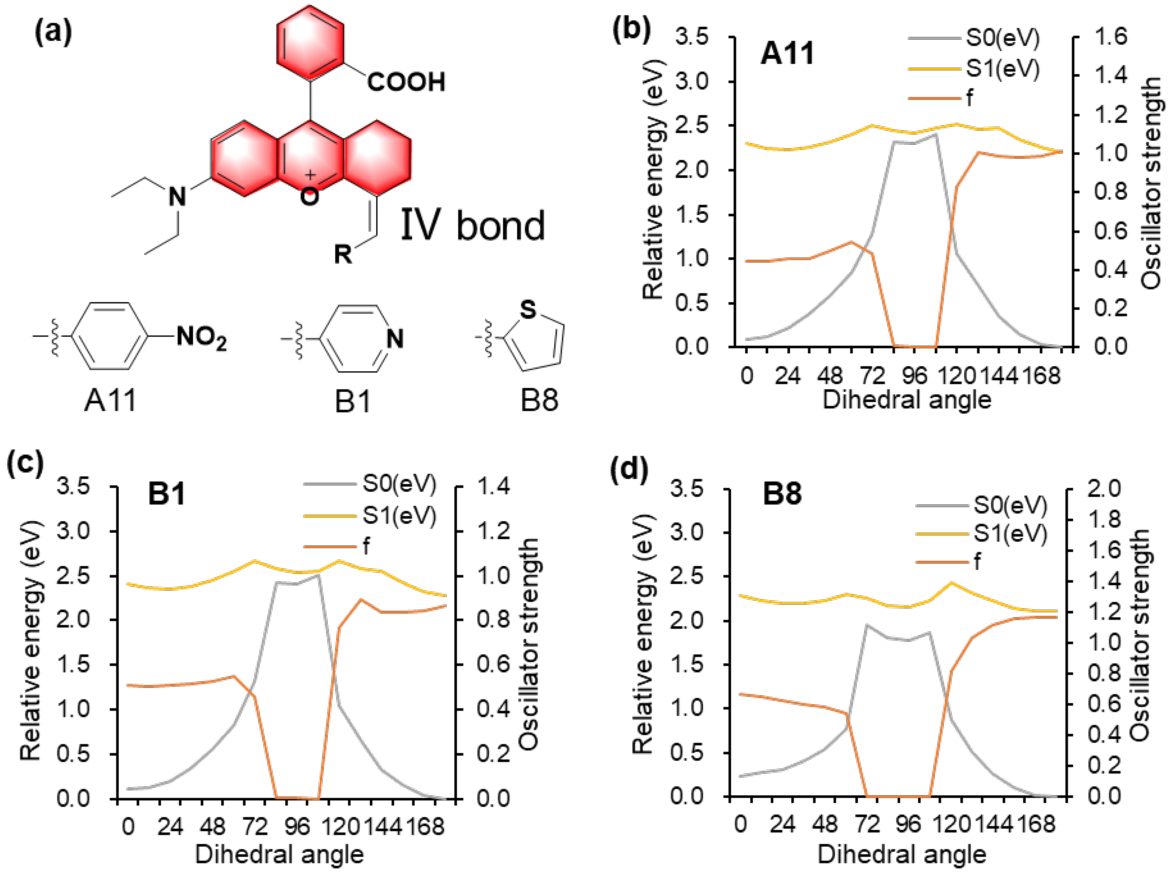


**Figure S42.** (a) Scheme of NRhFluors-A11, B1 and B8 with indications of IV bond. (b) The $S_0$ and $S_1$ energies of the multi-angle potential energy surface scanning of the IV bond in the A11 structure were recorded, as well as the changes in oscillation intensity. (c) The $S_0$ and $S_1$ energies of the multi-angle potential energy surface scanning of the IV bond in the B1 structure were recorded, as well as the changes in oscillation intensity. (d)The $S_0$ and $S_1$ energies of the multi-angle potential energy surface scanning of the IV bond in the B8 structure were recorded, as well as the changes in oscillation intensity. Around 90° torsion angle, S0 and S1 approached and the oscillator strength was almost zero, indicating that the excited state molecule was undergoing the RACI process.

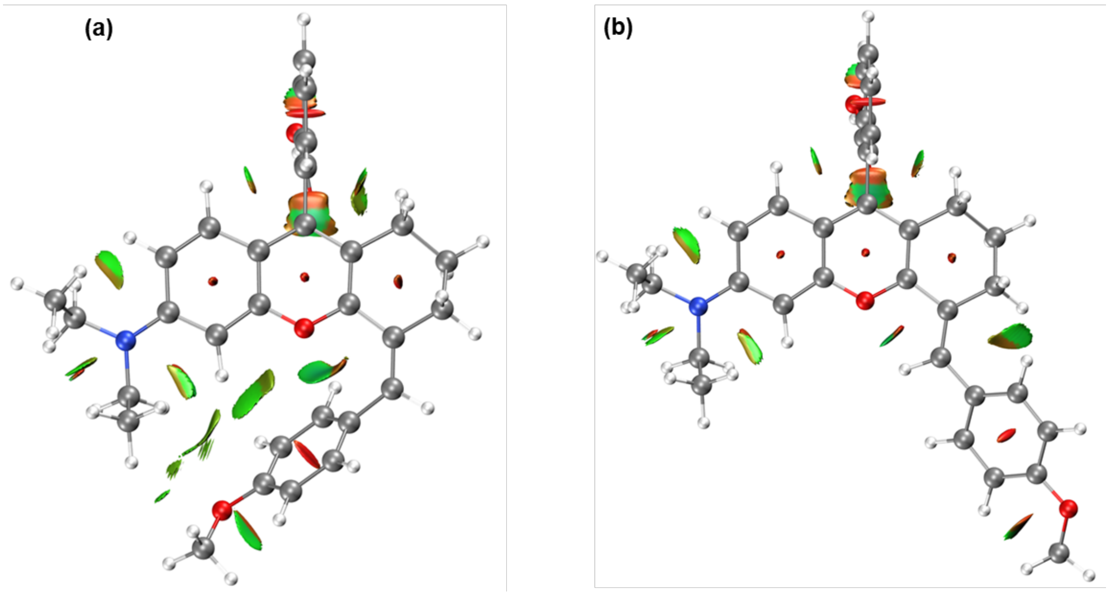


**Figure S43.** Intramolecular interactions of cis-trans isomerism of A1. (a) The cis structured molecules of A1. (b) The trans structured molecules of A1. The reduced density gradient (RDG) method was used to study intramolecular weak interaction.

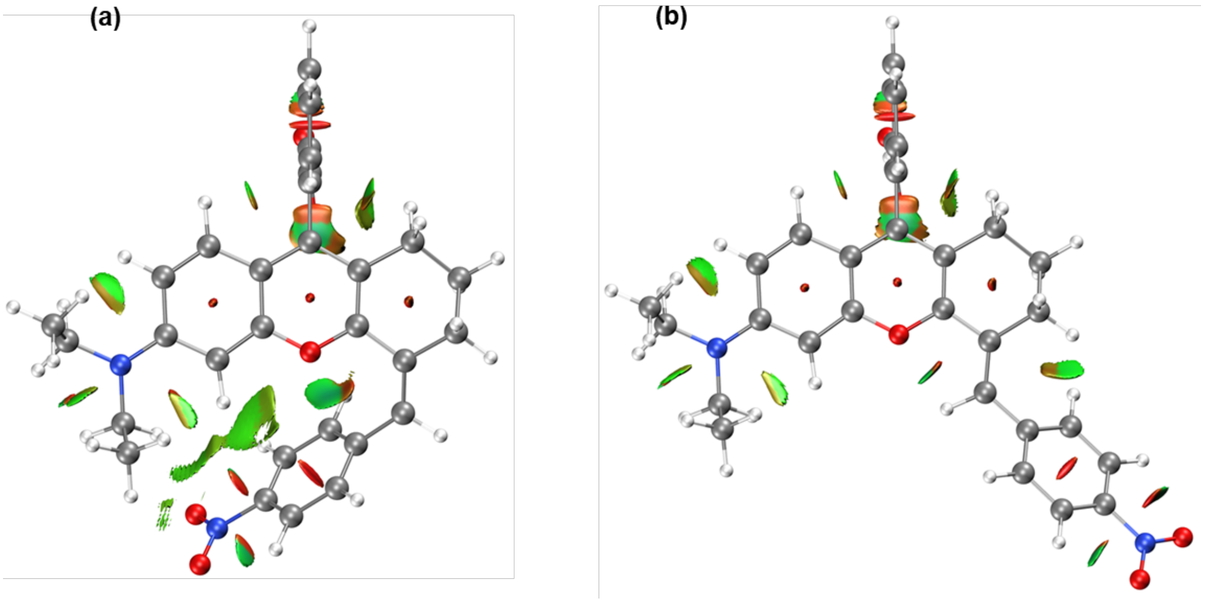


**Figure S44.** Intramolecular interactions of cis-trans isomerism of A11. (a) The cis structured molecules of A11. (b) The trans structured molecules of A11. The reduced density gradient (RDG) method was used to study intramolecular weak interaction.

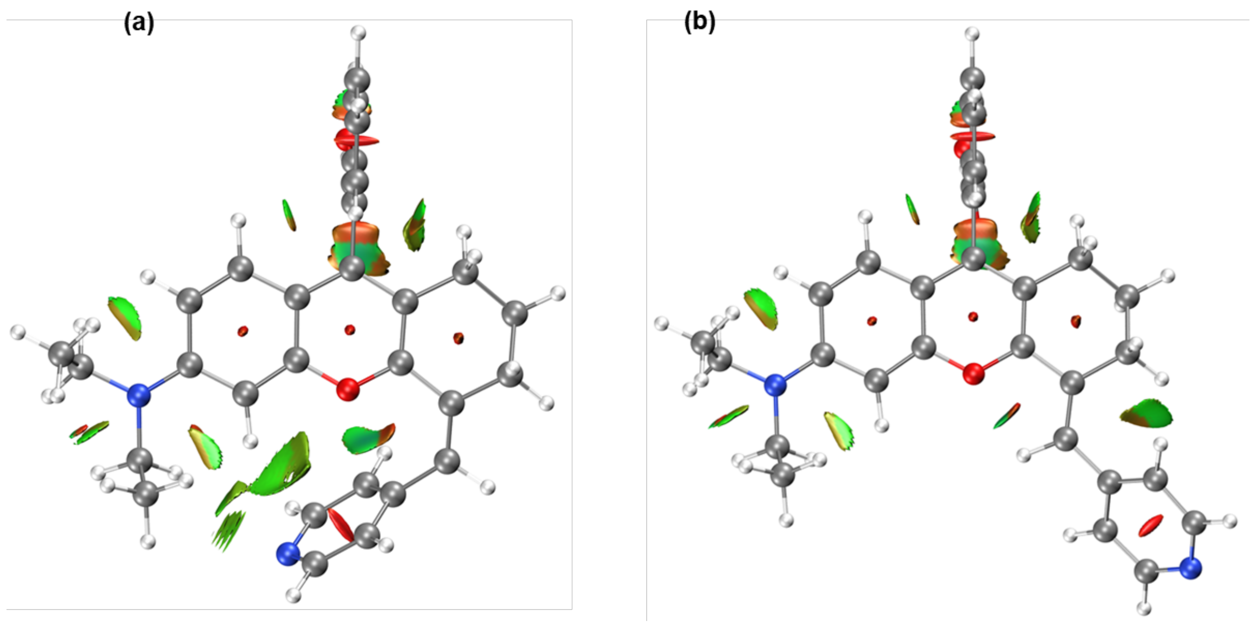


**Figure S45.** Intramolecular interactions of cis-trans isomerism of B1. (a) The cis structured molecules of B1. (b) The trans structured molecules of B1. The reduced density gradient (RDG) method was used to study intramolecular weak interaction.

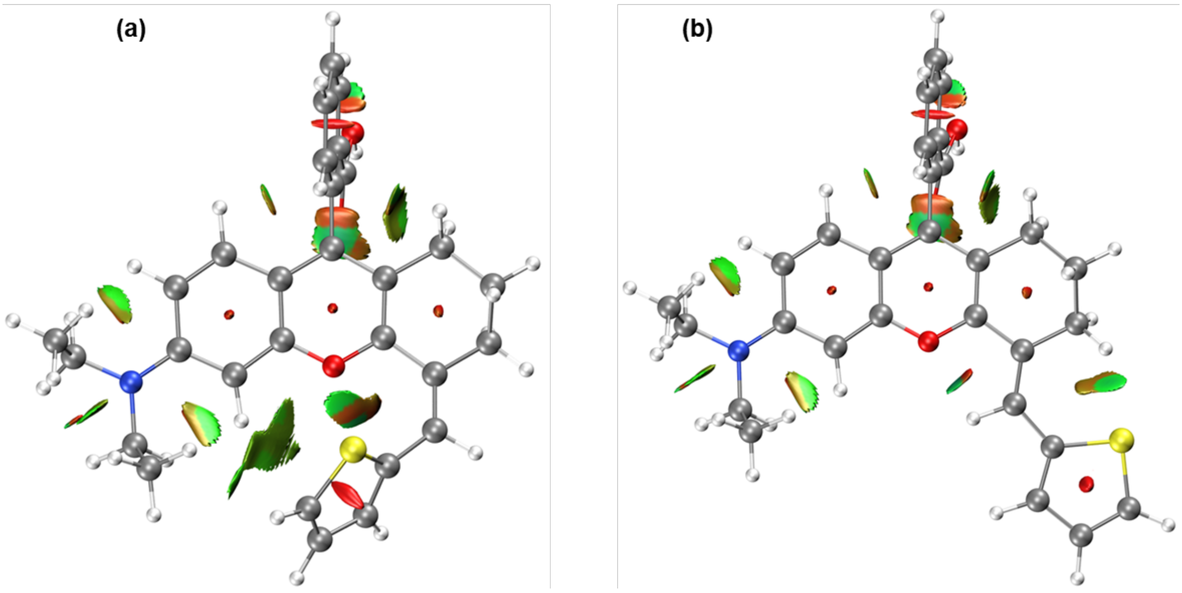


**Figure S46.** Intramolecular interactions of cis-trans isomerism of B8. (a) The cis structured molecules of B8. (b) The trans structured molecules of B8. The reduced density gradient (RDG) method was used to study intramolecular weak interaction.

### 3.5 pH dependence of A9-Halo

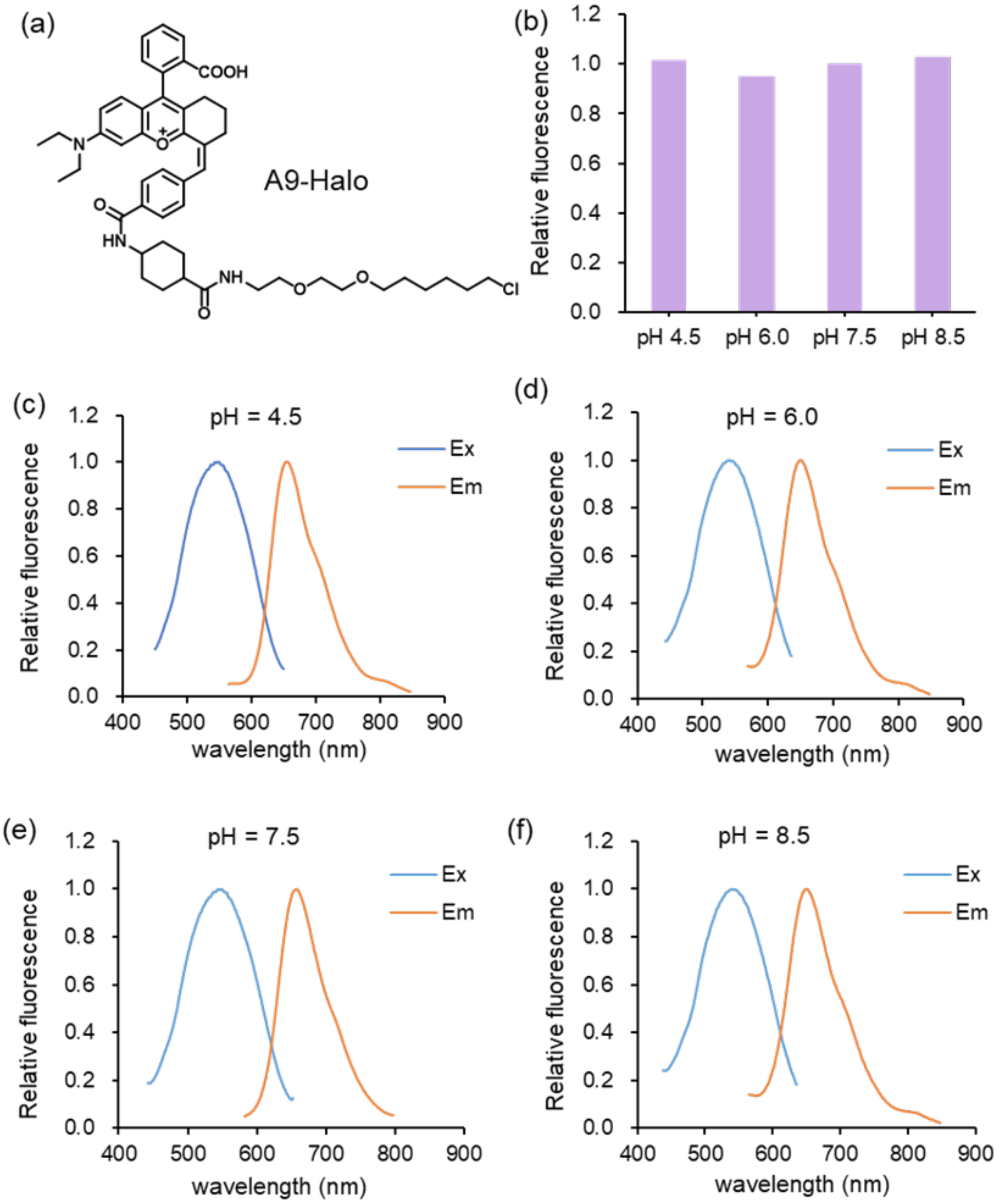


**Figure S47:** Excitation and emission wavelength of A9-Halo are insensitive to pH in glycerol-buffer mixtures. A9-Halo (20 µM) was prepared in indicated buffer-glycerol mixtures. (a) A9-Halo. (b) Bar graph of A9-Halo fluorescence response in different pH values. The fluorescence intensity of A9-Halo in different pH value glycerol-buffer mixtures are almost the same. (c) Excitation and emission spectrum in 90% glycerol/ 10% sodium acetate buffer pH=4.5. (d) Excitation and emission spectrum in 90% glycerol/ 10% water (pH 6.0). (e) Excitation and emission spectrum in 90% glycerol/ 10% PBS (pH=7.5). (f) Emission spectrum and emission in 90% glycerol/ 10% Tris (pH=8.5). Ex = 549 nm.

### 3.6 In vitro Protein Aggregation Assays

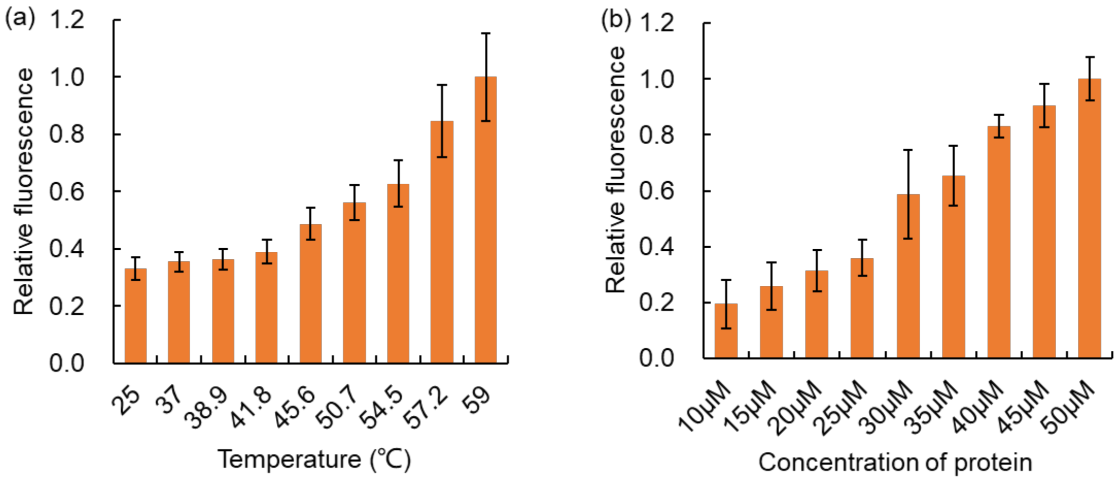


**Figure S48:** (a) Heat-induced aggregation of SOD1(A4V) increases fluorescence intensity of probe. The probe (20 µM) and protein (40 µM) were incubated at a series of temperatures for 10 minutes, and the control group was placed at room temperature of 25 ºC. (b) Fluorescence intensity of SOD1(A4V) aggregates (10, 15, 20, 25, 30, 35, 40, 45, 50 µM) in the presence of 20 µM of probe. SOD1(A4V) aggregation was induced at 59 ºC for 20 min in buffer (50 mM Tris-HCl, pH 7.5, 100 mM NaCl, 80 mM EDTA) (Ex = 549 nm). Error bars: standard error (n=3).

### 3.7 A9-Halo and BPY-Halo visualizes protein aggregates via fluorogenic signals in live cells.

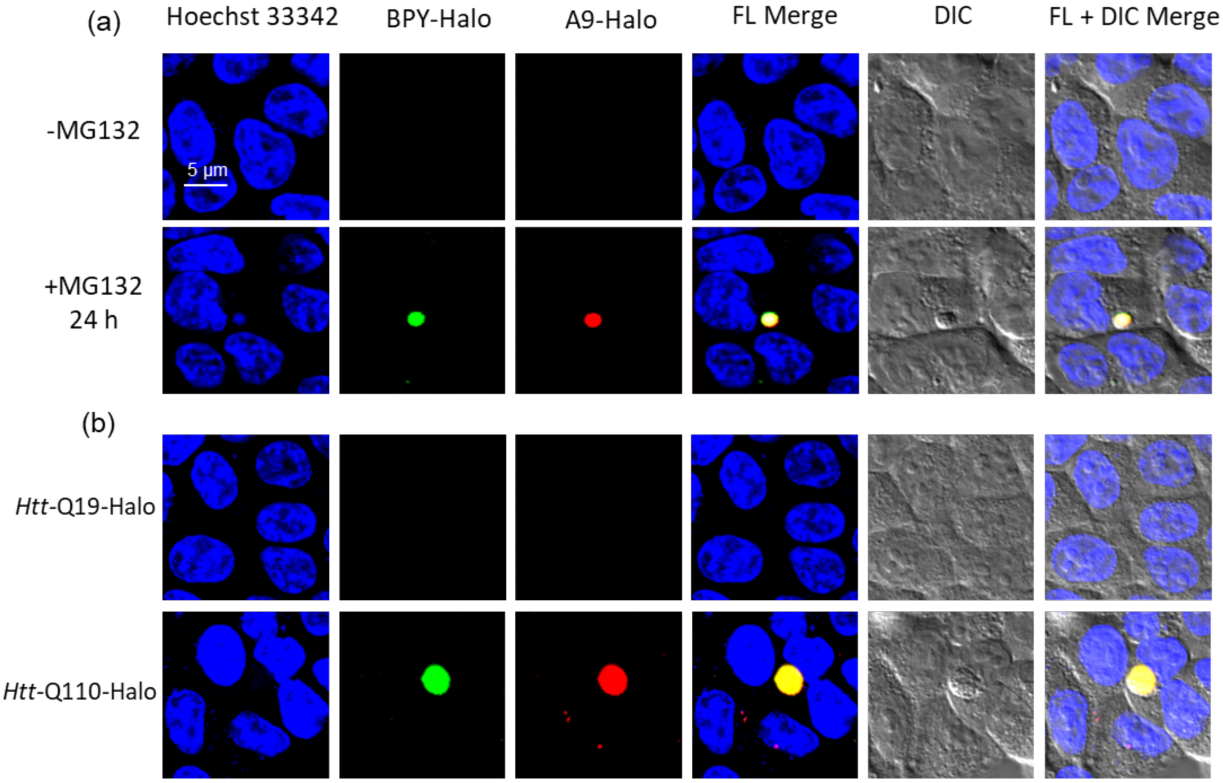


**Figure S49:** (a) During the transfection of the SOD1(A4V)-Halo, 2 μM BPY-Halo and 2 μM A9-Halo were used simultaneously to act on HEK293 cells to fully express the protein for 24 hours. MG132 (1 μM) were used to induce 24 hours respectively. Before confocal fluorescence imaging, the cells were incubated with fresh DMEM containing 5 μM Hoechst 33342 at 37 ºC for 30 minutes, and the redundant probes were washed off. Both induction methods can promote the transition of protein from misfolding to aggregation, which is manifested by the fluorescence activation of A9-Halo and BPY-Halo aggregates around the nucleus, and the superposition of green and yellow channels is yellow. (b) During the transfection of *Htt* protein in HEK293 cells, 2 μM BPY-Halo and 2 μM A9-Halo were added at the same time to fully express for 48 hours. No processing required. Blue: Hoechst 33342. Green: BPY-Halo. Red: A9-Halo. Scale bar: 5 μm.

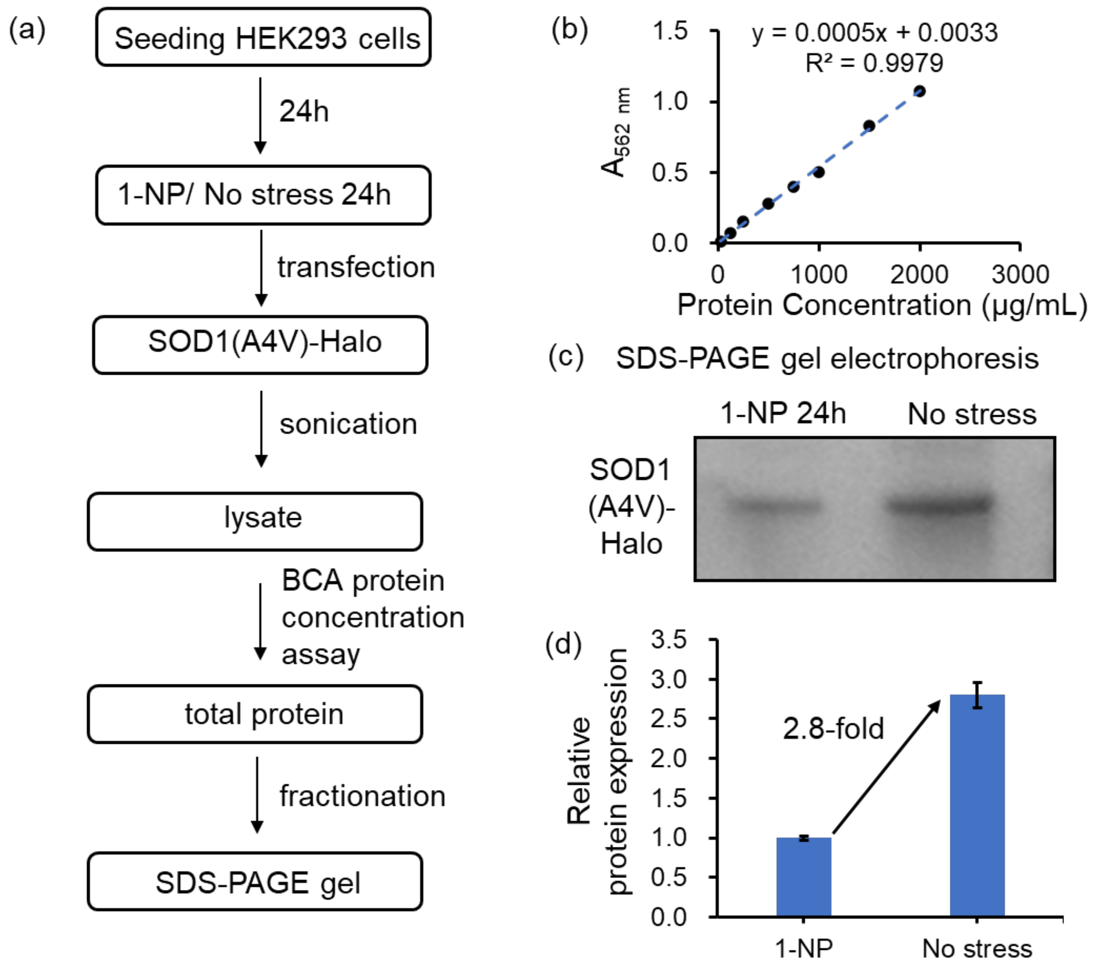


**Figure S50:** (a) HEK293 cells were treated with mitochondrial damaging agents (1-NP) for 24 hours (the other group did not undergo any induction), followed by induction of transfection and complete expression of SOD1(A4V)-Halo for 24 hours. Harvest cells with or without 1-NP stress and the concentration of lysates was calculated by BCA protein concentration assay after sonication lysis and normalized to the same concentration. Finally, lysate samples were analyzed using SDS-PAGE gel electrophoresis. (b) Standard curve for BCA protein concentration determination. The total protein concentration of the blank group was 297.9 μg/ml and the total protein concentration of the 1-NP treated group was 183.9 μg/ml. (c) The SDS-PAGE gel electrophoresis showed that mitochondrial damage reduced SOD1(A4V)-Halo expression. Normalize the protein concentration of blank group to 183.9 μg/ml before SDS-PAGE gel. (d) Relative protein expression statistics. The SDS-PAGE results were normalized by image gray scale analysis.

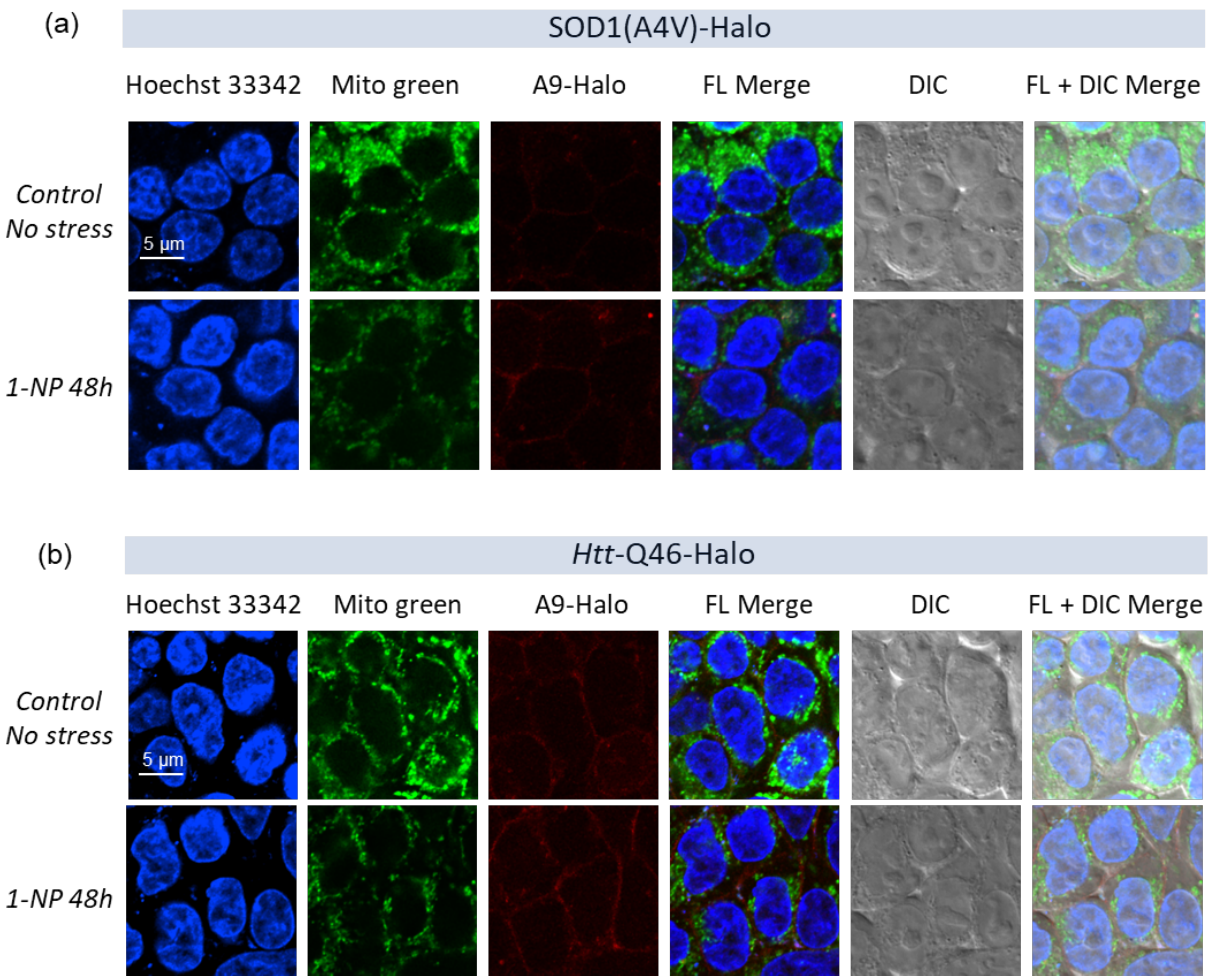


**Figure S51:** (a) SOD1 (A4V)-Halo was transfected into healthy HEK293 cells and fully expressed for 24 hours. Subsequently, mitochondrial damaging agent 1-NP was used in the experimental group for 48 hours, while no induction was performed in the control group. Finally, add 2 µM Mito-Tracker Green and 2 µM A9-Halo for co-incubation for 12 hours. (b) *Htt*-Q46-Halo was transfected into healthy HEK293 cells and fully expressed for 24 hours. Subsequently, mitochondrial damaging agent 1-NP was used in the experimental group for 48 hours, while no induction was performed in the control group. Finally, add 2 µM Mito-Tracker Green and 2 µM A9-Halo for co-incubation for 12 hours. No collective fluorescence signal was observed in the red channel, indicating that insoluble aggregates were not formed in the cells. Blue: Hoechst 33342. Green: Mito-Tracker Green. Red: A9-Halo. Scale bar: 5 µm.

### 3.8 Fluorescence lifetime imaging

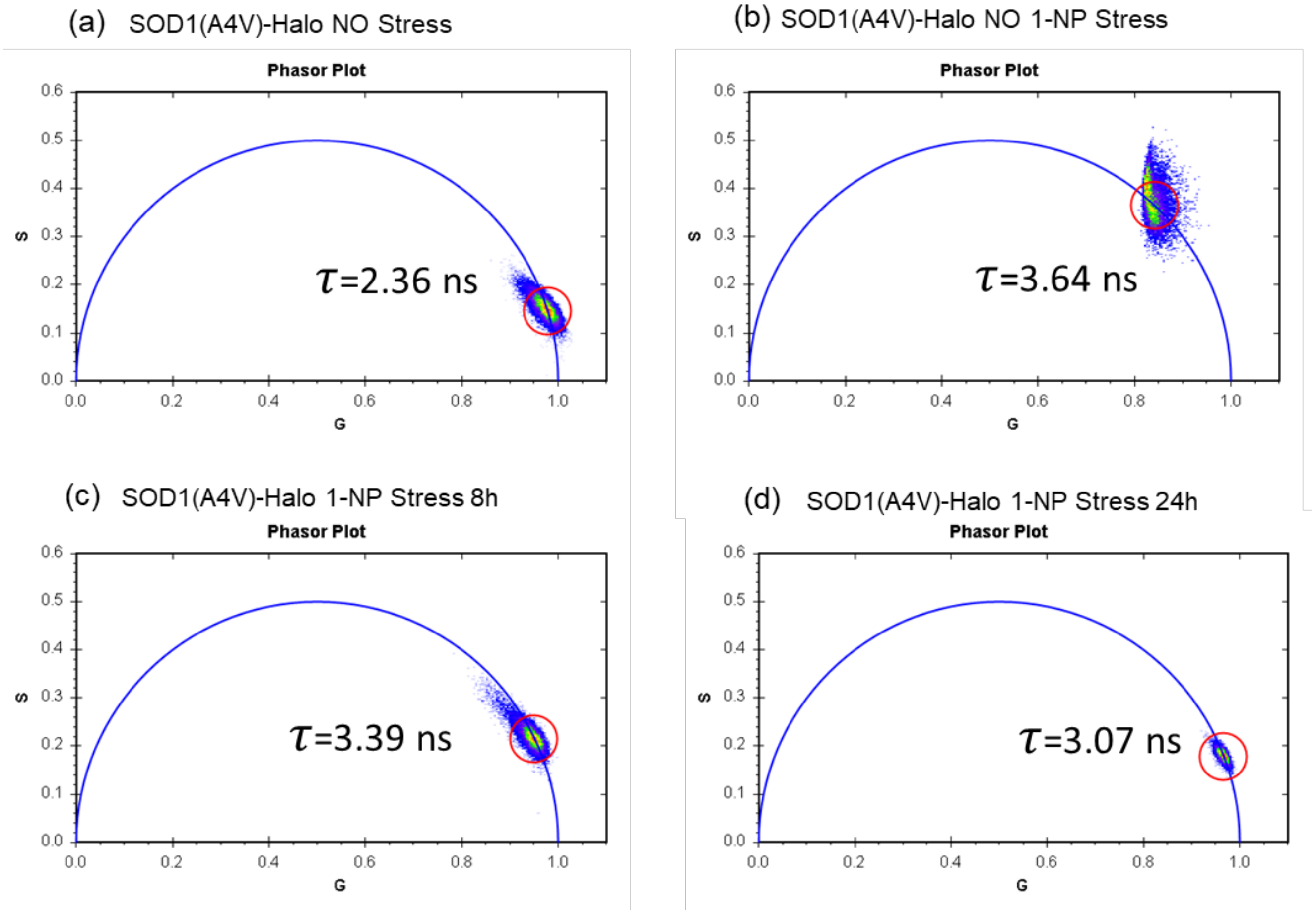


**Figure S52:** The lifetime phasor plot of A9-Halo in SOD1(A4V)-Halo. (a) The phasor plot of no aggregates. (b) The phasor plot of MG132 stress 24h in SOD1(A4V)-Halo. (c) Apply 1-NP (1μM) to induces mitochondrial damage 8h before protein transfection in cells. (d) Apply 1-NP (1μM) to induces mitochondrial damage 24h before protein transfection in cells.

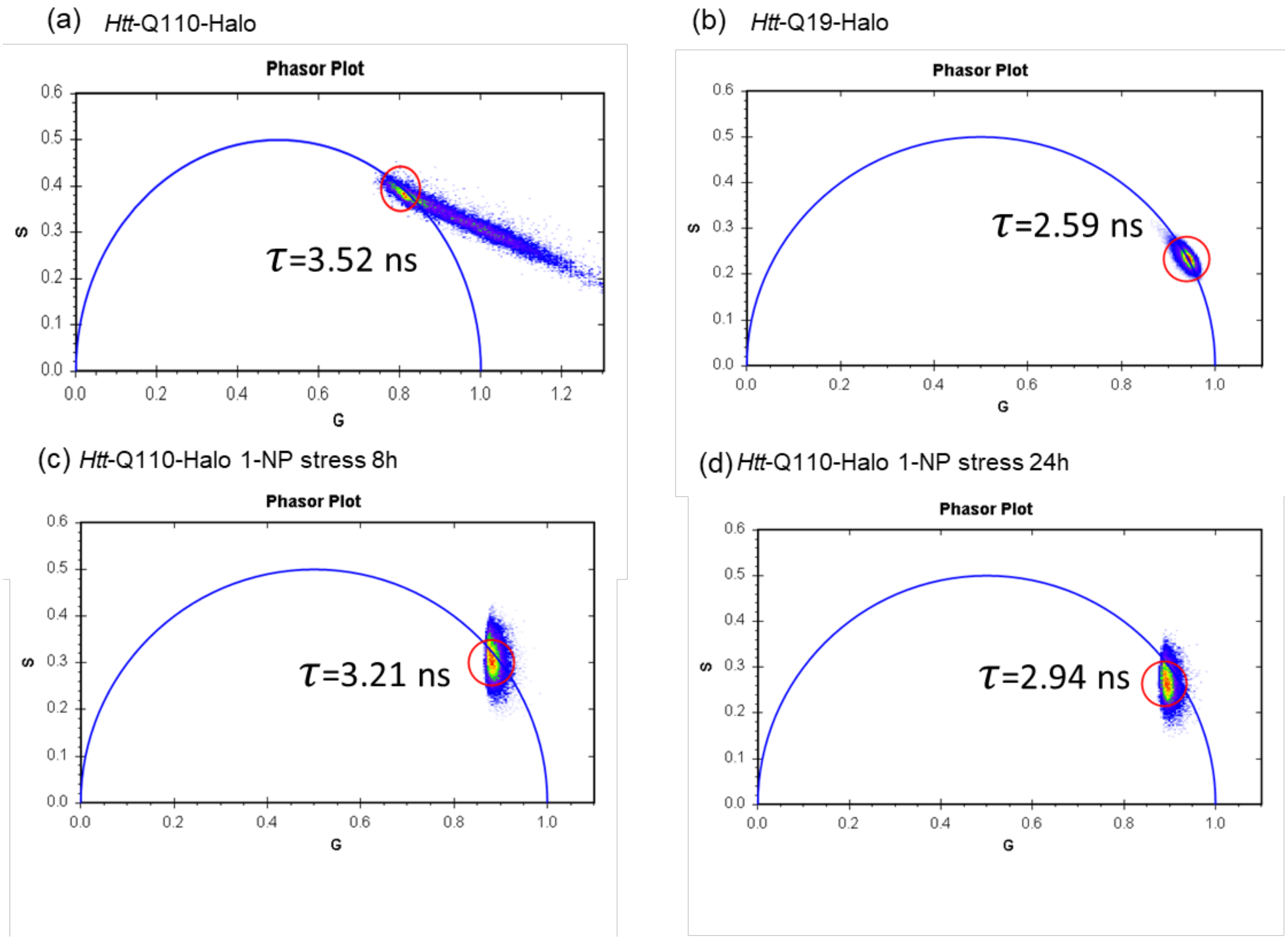


**Figure S53:** The lifetime phasor plot of A9-Halo in *Htt*- protein. (a) The phasor plot of A9-Halo in *Htt*-Q110-Halo. (b) The phasor plot of A9-Halo in *Htt*-Q19-Halo. (c) Apply 1-NP (1μM) to induces mitochondrial damage 8h before *Htt*-Q110-Halo protein transfection in cells. (d) Apply 1-NP (1μM) to induces mitochondrial damage 24h before *Htt*-Q110-Halo protein transfection in cells.

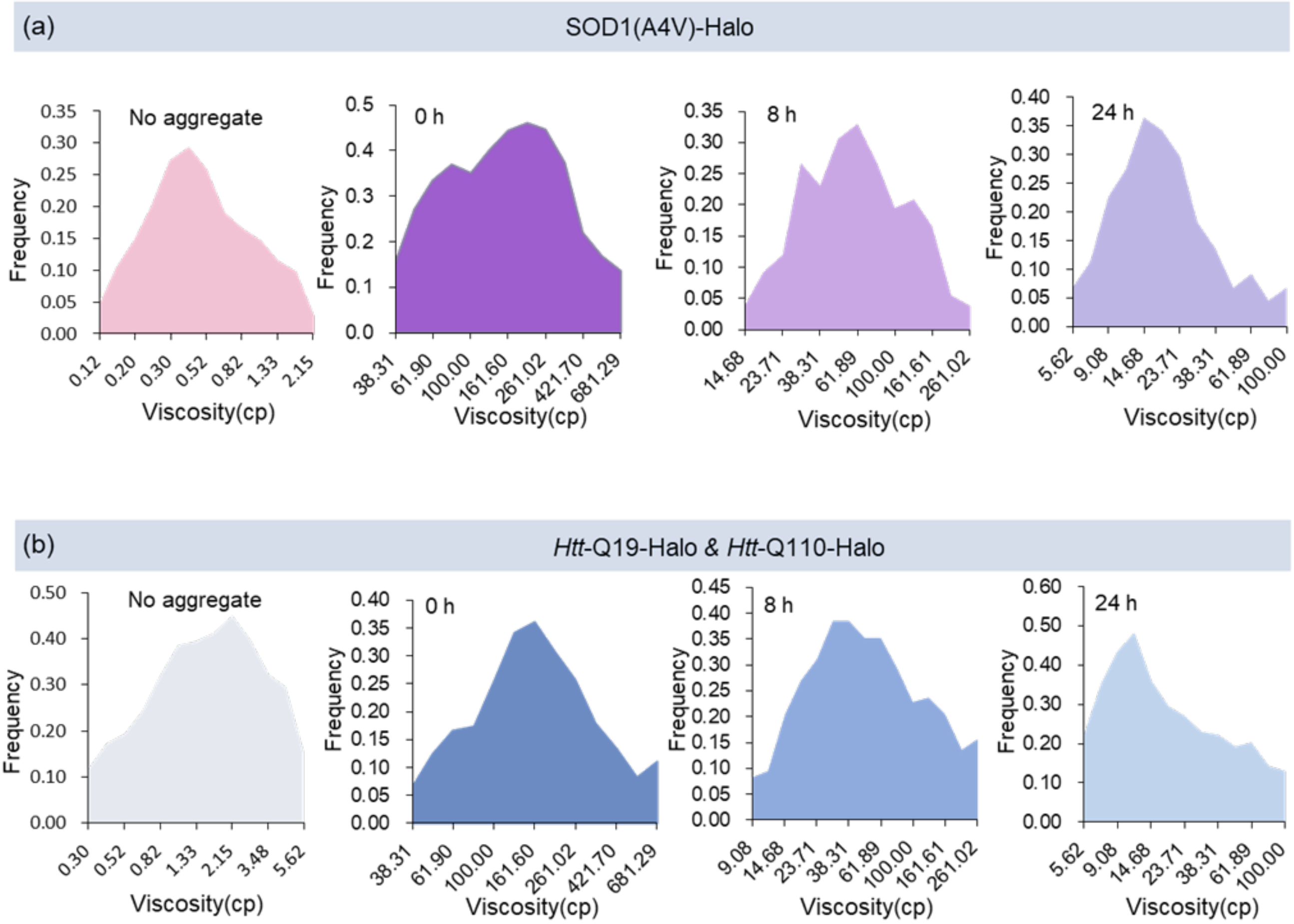


**Figure S54:** (a) The distribution of intracellular viscosity signals in SOD1(A4V)-Halo protein aggregates under varying degrees of mitochondrial damage. (b) The distribution of intracellular viscosity signals in *Htt*- protein aggregates under varying degrees of mitochondrial damage.

**4. Synthetic Methods**

All reagents and anhydrous solvents of commercial grade were used as received unless otherwise states. Reaction progress of the compound was monitored by ultraviolet lamp in 254 nm or 365 nm. All compounds were isolated and purified by chromatographic column and monitored by thin layer chromatography (TLC). All absorption spectra involved in this study were scanned by Cary 5000 UV-Vis Spectrophotometer. All fluorescence spectra including excitation and emission spectra involved are scanned by HITACHI F7100 Fluorescence spectrophotometer. $^{1}$H-NMR and $^{13}$C-NMR characterization of isolated products was completed in chloroform-d ($CDCl_3$), d6-dimethylsulfoxide (DMSO-$d_6$) and methanol-d ($CD_3OD$).

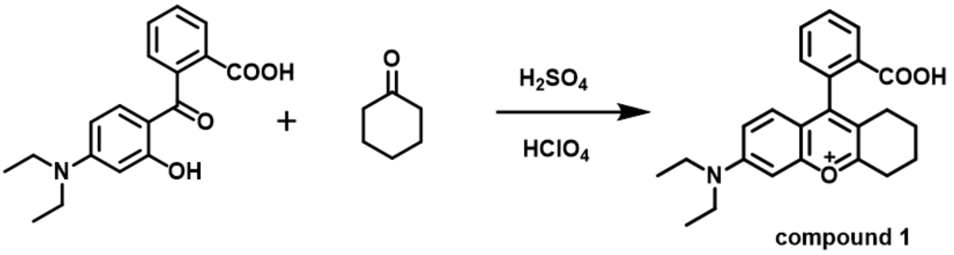


**Scheme S1.** Synthesis of compound 1.

Carefully remove 14 mL of $H_2SO_4$ and pour it into a round-bottomed flask containing appropriate magnetons. Cyclohexanone (1.32 mL, 12.78 mmol) was added drop by drop in an ice bath stirring condition. After a few minutes, 2-(4-(Diethylamino)-2-hydroxybenzoyl)benzoic acid (2.00 g, 6.40 mmol) was weighed and added into the round-bottom flask several times. After full stirring, the reaction was removed from the ice bath. After returning to room temperature, the reaction was heated to 90 °C for 1.5 hours. Prepare a certain amount of the mixture of crushed ice and water in a beaker. Pour the cooled mixture into the beaker after the reaction. $HClO_4$ (70%, 1.40 mL) was added to the beaker and stirred well with a glass rod. The solution was filtered through a Brinner funnel, and the orange-red solid was left to dry in a vacuum drying oven for several hours. After column purification, compound 1 (1.60 g) was finally obtained with a yield of 80%.[3]

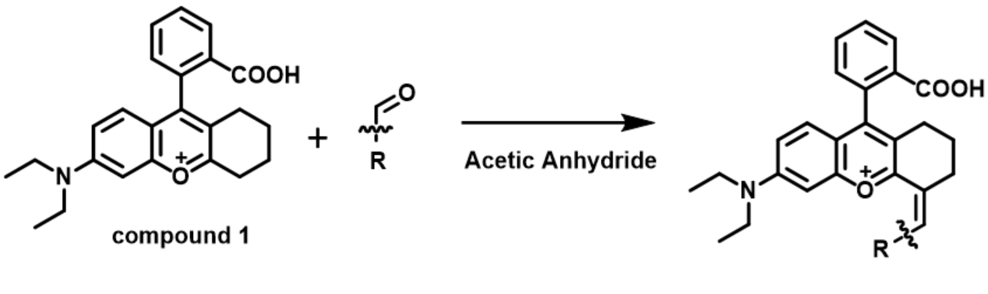


**Scheme S2.** Synthesis of A1-A12, B1-B8.

Compound 1 (1 eq) and a range of aldehyde groups (2 eq) were dissolved in 8 mL acetic anhydride. The reaction system was heated to 90°C and stirred for 2h. After the reaction, 10 mL water was added to quench the reaction and cooled to room temperature. The reaction mixture was extracted three times with DCM and water respectively, and the organic layer was collected and dried with anhydrous $Na_2SO_4$. The

solvent was then removed by a rotary evaporator and the crude products were collected. The crude product was purified by silica gel column chromatography with $CH_2Cl_2/CH_3CH_2OH$ (200:1-30:1, V/V).

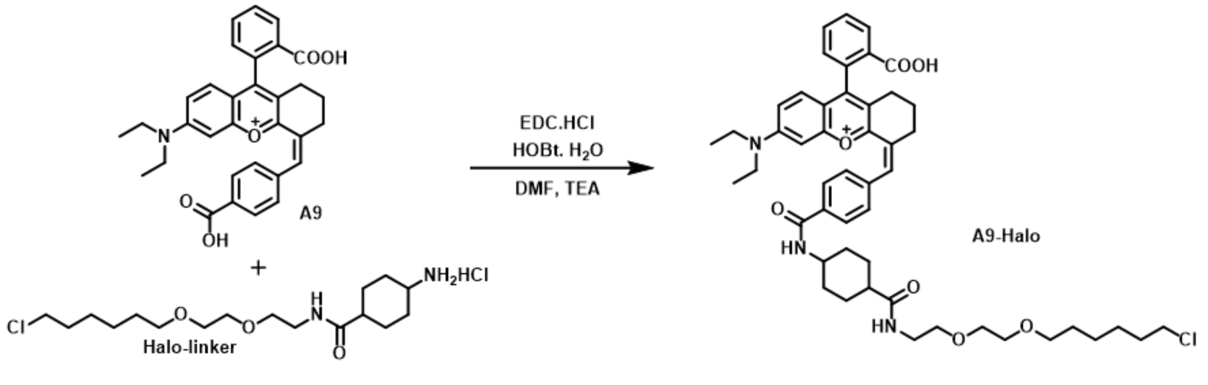


**Scheme S3.** Synthesis of A9-Halo

In 10 mL of DMF, compound A9 (1 eq), Halo-linker (2 eq), EDC.HCl (1.2 eq), HOBt.$H_2O$ (1.2 eq), and finally triethylamine (1.2 eq) were added dropwise. Stirring was done overnight at room temperature. Purification by thin layer chromatography yielded the purple solid A9-Halo.

## 5. HRMS and H/C NMR spectra

(*Z*)-9-(2-carboxyphenyl)-6-(diethylamino)-4-(4-methoxybenzylidene)-1,2,3,4-tetrahydroxanthylium **(A1)**:

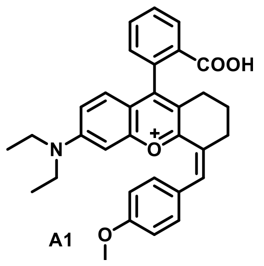


A1: $^{1}$H NMR (400 MHz, MeOD) δ 8.14 (dd, *J* = 5.7, 3.2 Hz, 1H), 8.07 (s, 1H), 7.66 – 7.61 (m, 4H), 7.19 – 7.12 (m, 4H), 7.05 (d, *J* = 8.7 Hz, 2H), 3.87 (s, 3H), 3.69 (q, *J* = 6.9 Hz, 4H), 3.05 – 2.87 (m, 2H), 2.54 (dt, *J* = 11.3, 5.4 Hz, 1H), 2.45 – 2.32 (m, 1H), 1.83 (d, *J* = 5.7 Hz, 2H), 1.33 (t, *J* = 7.2, 3.8 Hz, 6H). $^{13}$C NMR (101 MHz, MeOD) δ 171.49, 160.84, 137.87, 135.05, 133.25, 133.18, 131.66, 131.60, 131.06, 130.92, 130.84, 130.14, 130.07, 129.41, 129.31, 129.23, 128.49, 117.02, 116.97, 114.78, 114.74, 113.19, 113.14, 95.97, 55.33, 53.89, 48.28, 48.07, 47.86, 47.64, 47.43, 47.22, 47.01, 45.32, 26.83, 25.49, 21.30, 12.12, 10.85. TOF (m/z) Anal. Calc'd for $C_{32}H_{32}NO_4^+$: 494.2326, Found: 494.2337.

(Z)-9-(2-carboxyphenyl)-6-(diethylamino)-4-(4-methylbenzylidene)-1,2,3,4-tetrahydroxanthylium **(A2)**:

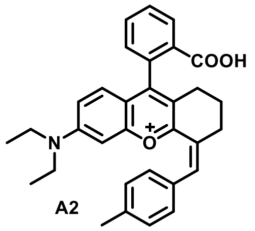


A2: $^{1}$H NMR (400 MHz, Chloroform-*d*) δ 7.87 (d, *J* = 7.6 Hz, 1H), 7.55 (t, *J* = 7.5 Hz, 1H), 7.46 (t, *J* = 7.4 Hz, 1H), 7.29 (s, 1H), 7.22 (d, *J* = 8.0 Hz, 2H), 7.14 (dt, *J* = 15.0, 7.8 Hz, 3H), 6.41 (d, *J* = 8.9 Hz, 1H), 6.35 (d, *J* = 2.4 Hz, 1H), 6.26 (dd, *J* = 8.9, 2.5 Hz, 1H), 3.27 (q, *J* = 7.0 Hz, 4H), 2.77 – 2.68 (m, 1H), 2.54 (dd, *J* = 14.3, 9.1 Hz, 1H), 2.29 (s, 3H), 1.96 (dd, *J* = 10.7, 5.4 Hz, 1H), 1.55 (s, 2H), 1.19 (d, *J* = 11.4 Hz, 1H), 1.09 (t, *J* = 7.0 Hz, 6H). $^{13}$C NMR (151 MHz, $CDCl_3$) δ 170.17, 152.67, 152.28, 149.29, 147.05, 136.78, 134.49, 134.34, 129.92, 129.44, 129.23, 128.93, 128.54, 127.61, 125.12, 124.91, 123.51, 108.68, 107.47, 104.79, 97.34, 44.43, 27.26, 23.05, 22.46, 21.29, 12.59. TOF (m/z) Anal. Calc'd for $C_{32}H_{32}NO_3^+$: 478.2377, Found: 478.2365.

(Z)-4-benzylidene-9-(2-carboxyphenyl)-6-(diethylamino)-1,2,3,4-tetrahydroxanthylium **(A3)**:

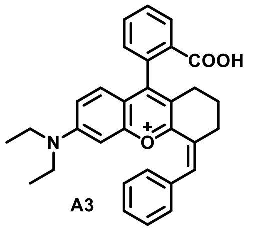


A3: $^{1}$H NMR (600 MHz, Chloroform-*d*) δ 8.12 (d, *J* = 7.2 Hz, 1H), 7.75 (s, 1H), 7.64 (t, *J* = 6.7 Hz, 1H), 7.60 – 7.52 (m, 1H), 7.47 (d, *J* = 6.7 Hz, 2H), 7.44 – 7.38 (m, 2H), 7.36 – 7.26 (m, 1H), 7.15 (d, *J* = 7.0 Hz, 1H), 6.77 (d, *J* = 8.9 Hz, 1H), 6.70 (s, 1H), 6.65 (d, *J* = 8.6 Hz, 1H), 3.54 – 3.45 (m, 4H), 2.80 (d, *J* = 24.2 Hz, 2H), 2.26 (d, *J* = 15.8 Hz, 1H), 1.97 (d, *J* = 15.8 Hz, 1H), 1.74 – 1.61 (m, 2H), 1.23 (s, 6H). $^{13}$C NMR (151 MHz, $CDCl_3$) δ 169.14, 155.60, 154.40, 152.57, 143.05, 136.31, 133.17, 131.17, 130.32, 130.15, 129.68, 129.60, 128.45, 128.25, 125.80, 115.84, 113.22, 111.51, 96.35, 45.41, 27.05, 24.51, 21.92, 12.63. TOF (m/z) Anal. Calc'd for $C_{31}H_{30}NO_3^+$: 464.2220, Found: 464.2236.

(Z)-9-(2-carboxyphenyl)-6-(diethylamino)-4-(4-iodobenzylidene)-1,2,3,4-tetrahydroxanthylium **(A4)**:

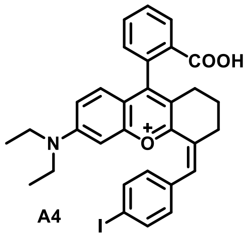

A4

A4: $^{1}$H NMR (600 MHz, Chloroform-*d*) δ 7.96 (d, *J* = 7.7 Hz, 1H), 7.70 (d, *J* = 8.0 Hz, 2H), 7.64 (t, *J* = 7.4 Hz, 1H), 7.55 (t, *J* = 7.5 Hz, 1H), 7.31 (s, 1H), 7.22 (d, *J* = 7.6 Hz, 1H), 7.13 (d, *J* = 8.0 Hz, 2H), 6.50 (d, *J* = 8.9 Hz, 1H), 6.43 (s, 1H), 6.37 (d, *J* = 6.5 Hz, 1H), 3.36 (q, *J* = 7.1 Hz, 4H), 2.76 (dd, *J* = 16.0, 6.5 Hz, 1H), 2.59 (t, *J* = 7.9 Hz, 1H), 2.09 – 2.01 (m, 1H), 1.64 (d, *J* = 4.6 Hz, 1H), 1.25 (s, 1H), 1.17 (t, *J* = 7.0 Hz, 6H). $^{13}$C NMR (151 MHz, $CDCl_3$) δ 170.05, 137.30, 136.69, 134.46, 131.34, 131.29, 129.32, 128.61, 125.13, 123.58, 97.22, 92.49, 44.49, 29.72, 29.67, 27.17, 23.09, 22.33, 12.58, 12.50. TOF (m/z) Anal. Calc'd for $C_{31}H_{29}INO_3^{+}$: 590.1187, Found: 509.1174.

(Z)-4-(4-bromobenzylidene)-9-(2-carboxyphenyl)-6-(diethylamino)-1,2,3,4-tetrahydroxanthylium **(A5)**:

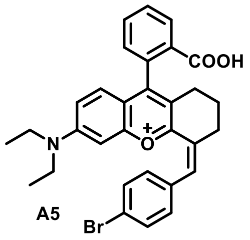

A5

A5: $^{1}$H NMR (600 MHz, Chloroform-*d*) δ 7.95 (d, *J* = 7.7 Hz, 1H), 7.72 (dd, *J* = 5.7, 3.3 Hz, 1H), 7.65 (t, *J* = 7.4 Hz, 1H), 7.57 – 7.53 (m, 1H), 7.50 (dd, *J* = 8.7, 2.5 Hz, 2H), 7.31 (s, 1H), 7.28 – 7.25 (m, 2H), 7.23 (d, *J* = 7.6 Hz, 1H), 6.49 (d, *J* = 8.8 Hz, 1H), 6.41 (d, *J* = 2.6 Hz, 1H), 6.35 (dd, *J* = 9.0, 2.6 Hz, 1H), 4.31 (t, *J* = 6.7 Hz, 1H), 3.35 (q, *J* = 7.2 Hz, 4H), 2.80 – 2.72 (m, 1H), 2.62 – 2.55 (m, 1H), 2.05 (d, *J* = 10.5 Hz, 1H), 1.72 (dd, *J* = 8.6, 6.3 Hz, 1H), 1.64 – 1.62 (m, 1H), 1.47 – 1.41 (m, 1H), 1.17 (t, *J* = 7.0 Hz, 6H). $^{13}$C NMR (151 MHz, $CDCl_3$) δ 170.14, 152.17, 149.27, 136.14, 134.59, 131.33, 131.27, 131.07, 130.99, 129.32, 128.86, 128.59, 127.51, 124.98, 123.94, 123.49, 120.85, 108.69, 97.16, 65.63, 44.45, 30.58, 27.18, 23.02, 22.37, 19.23, 13.82, 12.59. TOF (m/z) Anal. Calc'd for $C_{31}H_{29}BrNO_3^{+}$: 542.1325, Found: 542.1341.

(Z)-9-(2-carboxyphenyl)-4-(4-chlorobenzylidene)-6-(diethylamino)-1,2,3,4-tetrahydroxanthylium **(A6)**:

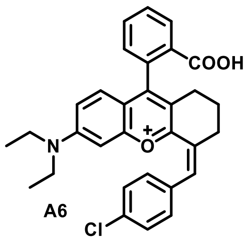

A6

A6: $^{1}$H NMR (400 MHz, Chloroform-*d*) δ 7.86 (d, *J* = 7.5 Hz, 1H), 7.55 (t, *J* = 7.3 Hz, 1H), 7.46 (t, *J* = 7.4 Hz, 1H), 7.24 (s, 5H), 7.14 (d, *J* = 7.5 Hz, 1H), 6.40 (d, *J* = 8.8 Hz, 1H), 6.33 (s, 1H), 6.27 (d, *J* = 8.6 Hz, 1H), 3.26 (q, *J* = 6.9 Hz, 4H), 2.67 (d, *J* = 15.0 Hz, 1H), 2.51 (d, *J* = 7.6 Hz, 1H), 1.95 (d, *J* = 12.0 Hz, 1H), 1.55 (s, 2H), 1.17 (s, 1H), 1.08 (t, *J* = 6.9 Hz, 6H). $^{13}$C NMR (101 MHz, $CDCl_3$) δ 170.11, 149.37, 135.74, 134.57, 132.69, 131.22, 130.77, 129.33, 128.59, 128.41, 124.98, 123.96, 123.52, 108.81, 97.29, 44.47, 27.21, 23.05, 22.42, 12.62, 1.09. TOF (m/z) Anal. Calc'd for $C_{31}H_{29}ClNO_3^{+}$: 498.1830, Found: 498.1808.

(Z)-9-(2-carboxyphenyl)-6-(diethylamino)-4-(4-fluorobenzylidene)-1,2,3,4-tetrahydroxanthylium **(A7)**:

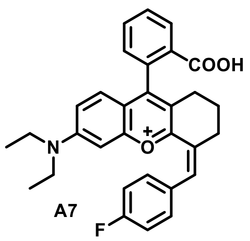


A7: $^{1}$H NMR (600 MHz, Chloroform-*d*) δ 7.95 (d, *J* = 7.6 Hz, 1H), 7.64 (t, *J* = 8.1 Hz, 1H), 7.55 (t, *J* = 7.5 Hz, 1H), 7.38 – 7.33 (m, 3H), 7.23 (d, *J* = 7.6 Hz, 1H), 7.07 (t, *J* = 8.7 Hz, 2H), 6.49 (d, *J* = 8.9 Hz, 1H), 6.41 (d, *J* = 2.5 Hz, 1H), 6.35 (dd, *J* = 8.9, 2.6 Hz, 1H), 3.35 (q, *J* = 7.1 Hz, 4H), 2.76 (dd, *J* = 13.7, 5.2 Hz, 1H), 2.58 (t, *J* = 13.0 Hz, 1H), 2.05 (dt, *J* = 11.8, 5.9 Hz, 1H), 1.64 (dd, *J* = 11.9, 7.5 Hz, 2H), 1.24 (t, *J* = 7.0 Hz, 1H), 1.17 (t, *J* = 7.1 Hz, 6H). $^{13}$C NMR (151 MHz, $CDCl_3$) δ 170.12, 152.66, 152.22, 149.32, 146.75, 134.54, 133.31, 133.29, 131.11, 131.06, 130.52, 129.28, 128.57, 127.58, 124.95, 124.03, 123.49, 115.24, 115.09, 108.72, 107.90, 104.70, 97.28, 44.43, 27.12, 23.00, 22.41, 12.58. TOF (m/z) Anal. Calc'd for $C_{31}H_{29}FNO_3^+$: 482.2126, Found: 482.2105.

(Z)-9-(2-carboxyphenyl)-6-(diethylamino)-4-(4-(methoxycarbonyl)benzylidene)-1,2,3,4-tetrahydroxanthylium **(A8)**:

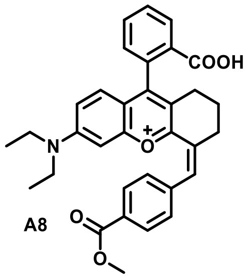


A8: $^{1}$H NMR (400 MHz, Chloroform-*d*) δ 8.13 – 8.02 (m, 3H), 7.77 (s, 1H), 7.70 – 7.64 (m, 1H), 7.55 (d, *J* = 7.7 Hz, 3H), 7.18 (d, *J* = 7.3 Hz, 1H), 6.82 – 6.72 (m, 2H), 6.69 (d, *J* = 9.1 Hz, 1H), 3.94 (s, 3H), 3.58 – 3.43 (m, 4H), 2.79 (d, *J* = 19.1 Hz, 2H), 2.25 (d, *J* = 12.3 Hz, 1H), 1.96 (d, *J* = 16.8 Hz, 1H), 1.70 (s, 2H), 1.25 (t, *J* = 6.5 Hz, 6H). $^{13}$C NMR (101 MHz, MeOD) δ 171.16, 166.28, 157.00, 154.27, 140.82, 131.59, 131.33, 130.69, 129.92, 129.86, 129.77, 129.48, 129.23, 128.45, 126.30, 115.05, 95.63, 60.96, 45.19, 44.06, 29.37, 26.79, 24.83, 21.55, 13.26, 11.56, 11.50, 11.42. TOF (m/z) Anal. Calc'd for $C_{33}H_{32}NO_5^+$: 522.2275, Found: 522.2255.

(Z)-4-(4-carboxybenzylidene)-9-(2-carboxyphenyl)-6-(diethylamino)-1,2,3,4-tetrahydroxanthylium **(A9)**:

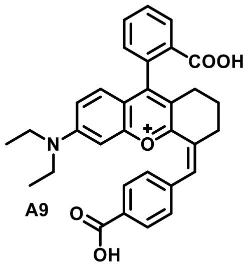


A9: $^{1}$H NMR (600 MHz, Methanol-*d*$_4$) δ 8.29 (d, *J* = 7.8 Hz, 1H), 8.17 (s, 1H), 8.07 (d, *J* = 8.3 Hz, 2H), 7.83 (t, *J* = 7.4 Hz, 1H), 7.75 (t, *J* = 7.7 Hz, 1H), 7.71 (d, *J* = 8.3 Hz, 2H), 7.34 (d, *J* = 7.5 Hz, 1H), 7.24 (dd, *J* = 4.8, 2.8 Hz, 2H), 7.11 (d, *J* = 10.2 Hz, 1H), 3.72 (q, *J* = 7.2 Hz, 4H), 2.99 – 2.94 (m, 2H), 2.40 (q, *J* = 6.4 Hz, 2H), 1.81 (dq, *J* = 19.1, 6.5 Hz, 2H), 1.33 (t, *J* = 7.1 Hz, 6H). $^{13}$C NMR (151 MHz, $CDCl_3$) δ 170.07, 137.31, 136.71, 134.48, 131.36, 131.31, 129.33, 128.62, 125.14, 123.59, 108.99, 97.23, 92.50, 44.50, 29.72, 27.17, 23.09, 22.34, 12.59, 12.50. TOF (m/z) Anal. Calc'd for $C_{32}H_{30}NO_5^+$: 508.2118, Found: 508.2122.

(Z)-9-(2-carboxyphenyl)-4-(4-cyanobenzylidene)-6-(diethylamino)-1,2,3,4-tetrahydroxanthylium **(A10)**:

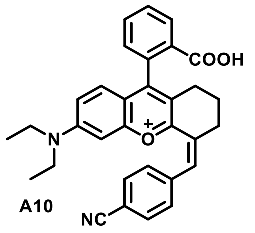


A10: $^{1}$H NMR (600 MHz, Chloroform-*d*) δ 7.96 (d, *J* = 7.7 Hz, 1H), 7.66 (dd, *J* = 7.1, 5.1 Hz, 3H), 7.56 (t, *J* = 7.6 Hz, 1H), 7.48 (d, *J* = 8.2 Hz, 2H), 7.37 (s, 1H), 7.23 (d, *J* = 7.6 Hz, 1H), 6.49 (d, *J* = 8.9 Hz, 1H), 6.42 (d, *J* = 2.5 Hz, 1H), 6.36 (dd, *J* = 8.9, 2.6 Hz, 1H), 3.36 (q, *J* = 7.1 Hz, 4H), 2.83 – 2.72 (m, 1H), 2.64 – 2.57 (m, 1H), 2.07 (dd, *J* = 11.1, 5.4 Hz, 1H), 1.66 (dt, *J* = 7.3, 4.2 Hz, 2H), 1.30 – 1.25 (m, 1H), 1.17 (t, *J* = 7.1 Hz, 6H). $^{13}$C NMR (151 MHz, $CDCl_3$) δ 170.02, 152.47, 152.08, 149.38, 146.26, 142.10, 134.65, 133.49, 132.08, 131.96, 130.02, 129.81, 129.42, 128.59, 127.48, 125.00, 123.46, 123.40, 119.06, 110.15, 109.68, 108.86, 104.50, 97.17, 44.43, 27.25, 22.99, 22.33, 12.57. TOF (m/z) Anal. Calc'd for $C_{32}H_{29}N_2O_3^+$: 489.2173, Found: 489.2180.

(Z)-9-(2-carboxyphenyl)-6-(diethylamino)-4-(4-nitrobenzylidene)-1,2,3,4-tetrahydroxanthylium **(A11)**:

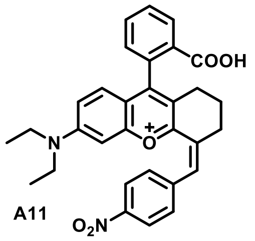


A11: $^{1}$H NMR (400 MHz, Chloroform-*d*) δ 8.15 (d, *J* = 8.8 Hz, 2H), 7.88 (d, *J* = 7.6 Hz, 1H), 7.58 (td, *J* = 7.5, 1.1 Hz, 1H), 7.52 – 7.48 (m, 1H), 7.45 (d, *J* = 8.7 Hz, 2H), 7.34 (s, 1H), 7.20 – 7.13 (m, 1H), 6.42 (d, *J* = 8.9 Hz, 1H), 6.34 (d, *J* = 2.5 Hz, 1H), 6.29 (dd, *J* = 8.9, 2.6 Hz, 1H), 3.28 (q, *J* = 7.1 Hz, 4H), 2.75 – 2.66 (m, 1H), 2.60 – 2.51 (m, 1H), 2.00 (dd, *J* = 11.0, 5.4 Hz, 1H), 1.59 (s, 2H), 1.21 (s, 1H), 1.10 (t, *J* = 7.0 Hz, 6H). $^{13}$C NMR (151 MHz, $CDCl_3$) δ 169.96, 152.37, 152.03, 149.36, 146.21, 146.18, 144.13, 134.61, 134.05, 130.05, 129.40, 128.56, 127.43, 125.00, 123.49, 123.43, 123.03, 110.05, 108.88, 104.45, 97.12, 44.42, 27.32, 22.98, 22.30, 12.55. TOF (m/z) Anal. Calc'd for $C_{31}H_{29}N_2O_5^+$: 509.2071, Found: 509.2054.

(Z)-9-(2-carboxyphenyl)-6-(diethylamino)-4-(4-(trifluoromethyl)benzylidene)-1,2,3,4-tetrahydroxanthylium **(A12)**:

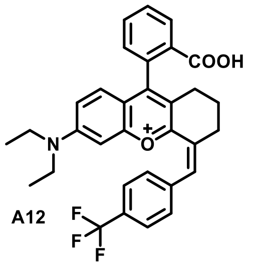


A12: $^{1}$H NMR (400 MHz, Chloroform-*d*) δ 7.97 (d, *J* = 7.6 Hz, 1H), 7.65 (dd, *J* = 13.6, 7.8 Hz, 3H), 7.56 (t, *J* = 7.5 Hz, 1H), 7.49 (d, *J* = 8.1 Hz, 2H), 7.43 (s, 1H), 7.27 – 7.19 (m, 1H), 6.52 (d, *J* = 8.9 Hz, 1H), 6.46 (d, *J* = 2.4 Hz, 1H), 6.39 (dd, *J* = 9.0, 2.5 Hz, 1H), 3.37 (q, *J* = 7.0 Hz, 4H), 2.78 (d, *J* = 15.7 Hz, 1H), 2.63 (d, *J* = 7.9 Hz, 1H), 2.14 – 2.03 (m, 1H), 1.66 (d, *J* = 7.6 Hz, 3H), 1.18 (t, *J* = 7.0 Hz, 6H). $^{13}$C NMR (101 MHz, $CDCl_3$) δ 169.97, 152.47, 149.68, 134.49,132.51, 129.69, 129.40, 128.69, 127.76, 125.32, 125.15,125.11, 124.25, 123.70, 109.71, 109.27, 97.22, 44.56, 27.20,23.17, 22.36, 12.60. TOF (m/z) Anal. Calc'd for $C_{32}H_{29}F_3NO_3^+$: 532.2094, Found: 532.2075.

(Z)-9-(2-carboxyphenyl)-6-(diethylamino)-4-(pyridin-4-ylmethylene)-1,2,3,4-tetrahydroxanthylium **(B1)**:

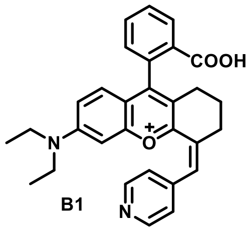


B1

B1: $^{1}$H NMR (400 MHz, MeOD) δ 8.61 (d, *J* = 6.0 Hz, 2H), 8.17 (d, *J* = 7.7 Hz, 1H), 7.91 (s, 1H), 7.74 (td, *J* = 7.5, 1.3 Hz, 1H), 7.68 (td, *J* = 7.5, 1.2 Hz, 1H), 7.57 (d, *J* = 5.9 Hz, 2H), 7.26 (d, *J* = 7.2 Hz, 1H), 7.13 – 7.06 (m, 2H), 7.02 (d, *J* = 10.1 Hz, 1H), 5.48 (s, 1H), 3.66 (q, *J* = 7.1 Hz, 4H), 2.91 (dd, *J* = 12.6, 5.5 Hz, 2H), 2.38 (dt, *J* = 16.5, 5.8 Hz, 1H), 2.26 (dt, *J* = 10.9, 6.0 Hz, 1H), 1.80 (dd, *J* = 12.4, 8.1 Hz, 2H), 1.30 – 1.27 (m, 6H). $^{13}$C NMR (151 MHz, METHANOL-$D_4$) δ 169.99, 164.44, 158.00, 154.60, 150.06, 140.52, 134.82, 134.72, 131.87, 130.81, 129.54, 129.37, 128.56, 123.27, 122.11, 121.83, 115.97, 115.65, 111.47, 95.35, 64.35, 45.26, 44.31, 43.16, 30.60, 30.48, 30.40, 29.44, 29.38, 27.37, 25.79, 21.09, 18.80, 12.67, 11.64, 11.50. TOF (m/z) Anal. Calc'd for $C_{30}H_{29}N_2O_3^+$: 465.2173, Found: 465.2158.

(Z)-9-(2-carboxyphenyl)-6-(diethylamino)-4-(quinolin-2-ylmethylene)-1,2,3,4-tetrahydroxanthylium **(B2)**:

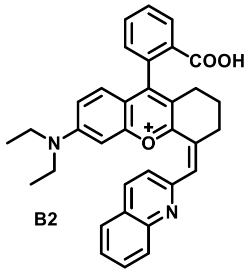


B2

B2: $^{1}$H NMR (400 MHz, Methanol-$d_4$) δ 8.32 (d, *J* = 8.5 Hz, 1H), 8.04 (d, *J* = 8.5 Hz, 2H), 7.94 – 7.88 (m, 2H), 7.79 – 7.75 (m, 1H), 7.73 (d, *J* = 6.2 Hz, 1H), 7.71 – 7.62 (m, 3H), 7.59 (d, *J* = 7.8 Hz, 1H), 7.20 (d, *J* = 7.0 Hz, 1H), 6.93 (s, 3H), 3.66 – 3.54 (m, 4H), 3.37 – 3.33 (m, 1H), 3.20 (dd, *J* = 16.0, 6.4 Hz, 1H), 2.34 (t, *J* = 11.1 Hz, 1H), 2.18 – 2.11 (m, 1H), 1.82 – 1.76 (m, 2H), 1.26 (t, *J* = 7.0 Hz, 6H). $^{13}$C NMR (151 MHz, METHANOL-$D_4$) δ 171.02, 155.64, 153.41, 147.80, 136.58, 134.88, 131.96, 129.95, 129.73, 129.57, 128.75, 128.55, 127.99, 127.59, 127.07, 126.99, 126.04, 123.64, 114.41, 95.77, 45.10, 29.44, 26.95, 24.64, 21.57, 11.55. TOF (m/z) Anal. Calc'd for $C_{34}H_{31}N_2O_3^+$: 515.2329, Found: 515.2310.

(Z)-9-(2-carboxyphenyl)-6-(diethylamino)-4-(naphthalen-2-ylmethylene)-1,2,3,4-tetrahydroxanthylium **(B3)**:

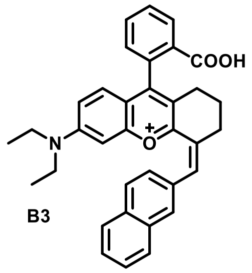


B3

B3: $^{1}$H NMR (600 MHz, Chloroform-*d*) δ 7.96 (d, *J* = 7.7 Hz, 1H), 7.86 – 7.80 (m, 4H), 7.64 (t, *J* = 7.1 Hz, 1H), 7.57 – 7.50 (m, 3H), 7.50 – 7.44 (m, 2H), 7.24 (d, *J* = 7.6 Hz, 1H), 6.50 (d, *J* = 8.9 Hz, 1H), 6.46 (d, *J* = 2.5 Hz, 1H), 6.35 (dd, *J* = 9.0, 2.5 Hz, 1H), 3.35 (q, *J* = 7.1 Hz, 4H), 2.90 (d, *J* = 15.5 Hz, 1H), 2.72 (dd, *J* = 14.3, 9.8 Hz, 1H), 2.08 (dd, *J* = 10.6, 5.3 Hz, 1H), 1.65 (dd, *J* = 10.9, 4.6 Hz, 3H), 1.17 (t, *J* = 7.1 Hz, 6H). $^{13}$C NMR (151 MHz, $CDCl_3$) δ 170.15, 152.64, 152.29, 149.34, 146.99, 134.81, 134.52, 133.29, 132.33, 130.99, 129.27, 128.57, 128.39, 128.03, 127.69, 127.65, 127.63, 126.22, 126.03, 125.24, 124.95, 123.52, 108.76, 108.04, 104.82, 97.37, 44.45, 27.39, 23.11, 22.50, 12.61. TOF (m/z) Anal. Calc'd for $C_{35}H_{32}NO_3^+$: 514.2377, Found: 514.2365.

(Z)-4-(benzo[b]thiophen-2-ylmethylene)-9-(2-carboxyphenyl)-6-(diethylamino)-1,2,3,4-tetrahydroxanthylium **(B4)**:

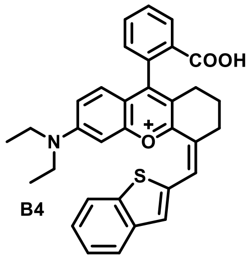


B4: $^{1}$H NMR (400 MHz, Methanol-$d_4$) δ 8.28 (s, 1H), 8.11 (dd, $J$ = 5.8, 3.2 Hz, 1H), 7.90 (dd, $J$ = 9.1, 4.1 Hz, 2H), 7.81 (s, 1H), 7.63 (dd, $J$ = 5.7, 3.3 Hz, 2H), 7.40 (dd, $J$ = 6.0, 3.2 Hz, 2H), 7.15 (dd, $J$ = 5.6, 3.2 Hz, 1H), 7.10 (d, $J$ = 5.3 Hz, 3H), 5.36 (dd, $J$ = 9.6, 4.4 Hz, 1H), 3.67 (q, $J$ = 7.1 Hz, 4H), 3.14 – 2.97 (m, 2H), 2.54 – 2.46 (m, 1H), 2.36 – 2.30 (m, 1H), 1.94 – 1.85 (m, 2H), 1.32 (dd, $J$ = 10.2, 3.1 Hz, 6H). $^{13}$C NMR (151 MHz, METHANOL-$D_4$) δ 171.44, 157.98, 155.10, 142.04, 139.31, 139.15, 130.43, 130.28, 129.44, 129.39, 128.04, 127.56, 127.12, 125.91, 124.90, 124.30, 121.92, 121.79, 116.16, 95.34, 45.41, 44.12, 29.44, 26.79, 25.22, 20.94, 11.57, 11.53. TOF (m/z) Anal. Calc'd for $C_{33}H_{30}NO_3S^+$: 520.1941, Found: 520.1920.

(Z)-4-((1H-indol-3-yl)methylene)-9-(2-carboxyphenyl)-6-(diethylamino)-1,2,3,4-tetrahydroxanthylium **(B5)**:

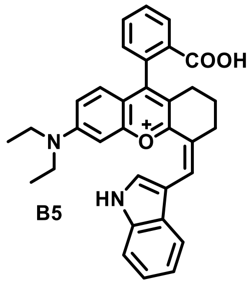


B5: $^{1}$H NMR (400 MHz, MeOD) δ 8.65 (s, 1H), 8.35 – 8.15 (m, 2H), 8.00 (s, 1H), 7.79 – 7.67 (m, 2H), 7.53 (d, $J$ = 5.8 Hz, 1H), 7.30 (dd, $J$ = 21.5, 11.6 Hz, 4H), 7.12 (s, 2H), 5.53 (s, 1H), 3.73 (d, $J$ = 6.7 Hz, 4H), 2.97 (s, 2H), 2.52 (s, 2H), 1.98 (d, $J$ = 17.9 Hz, 2H), 1.38 – 1.34 (m, 6H). $^{13}$C NMR (151 MHz, $CDCl_3$) δ 170.09, 154.99, 154.74, 152.31, 152.12, 149.49, 146.93, 134.57, 131.62, 129.40, 128.90, 128.63, 127.69, 125.10, 124.76, 123.61, 123.02, 121.00, 113.26, 111.02, 109.77, 108.98, 107.79, 104.88, 97.25, 44.51, 27.59, 23.02, 21.92, 12.62. TOF (m/z) Anal. Calc'd for $C_{33}H_{31}N_2O_3^+$: 503.2329, Found: 503.2337.

(Z)-4-((1H-imidazol-2-yl)methylene)-9-(2-carboxyphenyl)-6-(diethylamino)-1,2,3,4-tetrahydroxanthylium **(B6)**:

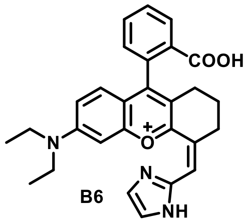


B6: $^{1}$H NMR (400 MHz, Methanol-$d_4$) δ 8.15 – 8.09 (m, 1H), 7.72 – 7.59 (m, 4H), 7.30 (s, 2H), 7.24 – 7.15 (m, 2H), 7.04 (s, 2H), 6.96 (s, 1H), 3.63 (q, $J$ = 7.0 Hz, 4H), 3.23 – 3.15 (m, 2H), 2.47 – 2.35 (m, 1H), 2.33 – 2.16 (m, 1H), 1.81 (p, $J$ = 6.2 Hz, 2H), 1.28 (d, $J$ = 2.7 Hz, 6H). $^{13}$C NMR (101 MHz, MeOD) δ 170.98, 157.86, 157.43, 154.72, 144.06, 131.21, 130.99, 130.03, 129.55, 129.16, 129.05, 128.50, 127.02, 124.78, 121.06, 118.68, 115.79, 115.63, 115.31, 95.28, 65.30, 30.46, 30.35, 29.38, 26.62, 25.07, 22.37, 20.92, 18.89, 11.50. TOF (m/z) Anal. Calc'd for $C_{28}H_{28}N_3O_3^+$: 454.2125, Found: 454.2107.

(Z)-9-(2-carboxyphenyl)-6-(diethylamino)-4-(thiazol-4-ylmethylene)-1,2,3,4-tetrahydroxanthylium **(B7)**:

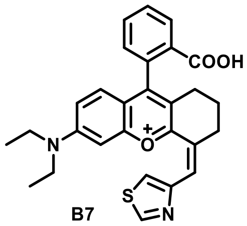

B7

B7: $^1$H NMR (400 MHz, $CD_3OD$_SPE) δ 9.07 (d, *J* = 1.5 Hz, 1H), 8.14 – 8.06 (m, 1H), 7.95 (d, *J* = 16.2 Hz, 2H), 7.66 – 7.58 (m, 2H), 7.16 (dd, *J* = 6.7, 2.0 Hz, 1H), 7.01 (s, 2H), 6.99 (s, 1H), 3.61 (q, *J* = 7.0 Hz, 4H), 3.18 (dd, *J* = 35.1, 11.4 Hz, 2H), 2.40 (dt, *J* = 15.9, 5.8 Hz, 1H), 2.21 (dd, *J* = 16.5, 6.6 Hz, 1H), 1.78 (s, 2H), 1.26 (t, *J* = 7.1 Hz, 6H). $^{13}$C NMR (151 MHz, $CDCl_3$) δ 170.17, 154.04, 152.48, 152.25, 152.04, 149.35, 146.84, 134.56, 131.73, 129.31, 128.54, 127.59, 124.97, 123.54, 117.74, 117.48, 108.93, 108.72, 104.67, 97.33, 44.46, 27.49, 22.93, 22.03, 12.61, 12.57. TOF (m/z) Anal. Calc'd for $C_{28}H_{27}N_2O_3S^+$: 471.1737, Found: 471.1715.

(Z)-9-(2-carboxyphenyl)-6-(diethylamino)-4-(thiophen-2-ylmethylene)-1,2,3,4-tetrahydroxanthylium **(B8)**:

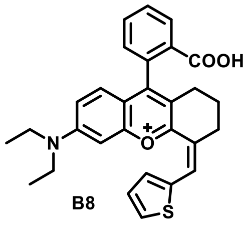

B8

B8: $^1$H NMR (400 MHz, Methanol-$d_4$) δ 8.32 (s, 1H), 8.10 (dd, *J* = 6.0, 3.0 Hz, 1H), 7.79 (d, *J* = 5.1 Hz, 1H), 7.64 – 7.58 (m, 3H), 7.26 (dd, *J* = 5.1, 3.8 Hz, 1H), 7.17 – 7.11 (m, 4H), 3.67 (q, *J* = 7.1 Hz, 4H), 3.03 – 2.88 (m, 2H), 2.57 – 2.47 (m, 1H), 2.42 – 2.33 (m, 1H), 1.94 – 1.84 (m, 2H), 1.30 (t, *J* = 7.1 Hz, 6H). $^{13}$C NMR (151 MHz, $CDCl_3$) δ 170.14, 152.59, 152.23, 149.32, 146.90, 140.56, 134.51, 129.26, 128.95, 128.56, 128.01, 127.61, 127.21, 126.45, 124.92, 123.50, 118.15, 108.71, 107.94, 104.71, 97.30, 44.43, 27.51, 22.87, 21.98, 12.60. TOF (m/z) Anal. Calc'd for $C_{29}H_{28}NO_3S^+$: 470.1784, Found: 470.1768.

(E)-9-(2-carboxyphenyl)-4-(4-((4-((2-(2-((6-chlorohexyl)oxy)ethoxy)ethyl)carbamoyl)cyclohexyl)carbamoyl)benzylidene)-6-(diethylamino)-1,2,3,4-tetrahydroxanthylium **(A9-Halo)** :

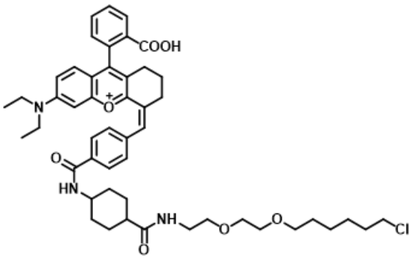

A9-Halo

A9-Halo: $^1$H NMR (600 MHz, Chloroform-*d*) δ 8.07 (d, *J* = 7.8 Hz, 1H), 7.81 (d, *J* = 8.0 Hz, 2H), 7.66 (t, *J* = 7.5 Hz, 1H), 7.59 (dd, *J* = 16.9, 9.3 Hz, 2H), 7.46 (d, *J* = 8.1 Hz, 2H), 7.17 (d, *J* = 7.6 Hz, 1H), 6.69 (d, *J* = 9.0 Hz, 1H), 6.66 – 6.62 (m, 1H), 6.57 (d, *J* = 8.4 Hz, 2H), 6.28 (d, *J* = 5.6 Hz, 1H), 3.96 (s, 1H), 3.65 – 3.61 (m, 2H), 3.60 – 3.58 (m, 2H), 3.57 – 3.55 (m, 2H), 3.53 (d, *J* = 6.7 Hz, 2H), 3.50 – 3.42 (m, 8H), 3.10 (q, *J* = 7.3 Hz, 1H), 2.77 – 2.63 (m, 2H), 2.13 (d, *J* = 11.7 Hz, 3H), 1.94 (d, *J* = 11.9 Hz, 2H), 1.78 (dt, *J* = 14.3, 6.8 Hz, 2H), 1.62 (dt, *J* = 14.6, 6.8 Hz, 6H), 1.48 – 1.43 (m, 2H), 1.37 (q, *J* = 8.5, 7.4 Hz, 5H), 1.22 (t, *J* = 7.0 Hz, 6H). $^{13}$C NMR (151 MHz, $CDCl_3$) δ 170.11, 167.08, 152.27, 149.39, 140.40, 134.44, 132.66, 131.95, 129.52, 129.30, 128.58, 127.69, 126.89, 125.08, 124.43, 123.54, 108.86, 97.08, 71.26, 70.21, 69.97, 69.73, 45.10, 45.07, 44.43, 39.65, 32.45, 32.43, 29.41, 27.23, 26.64, 26.61, 25.37, 23.05, 22.32, 12.54. TOF (m/z) Anal. Calc'd for $C_{49}H_{61}ClN_3O_7{}^+$:838.4193, Found: 838.4180.

**HRMS data**

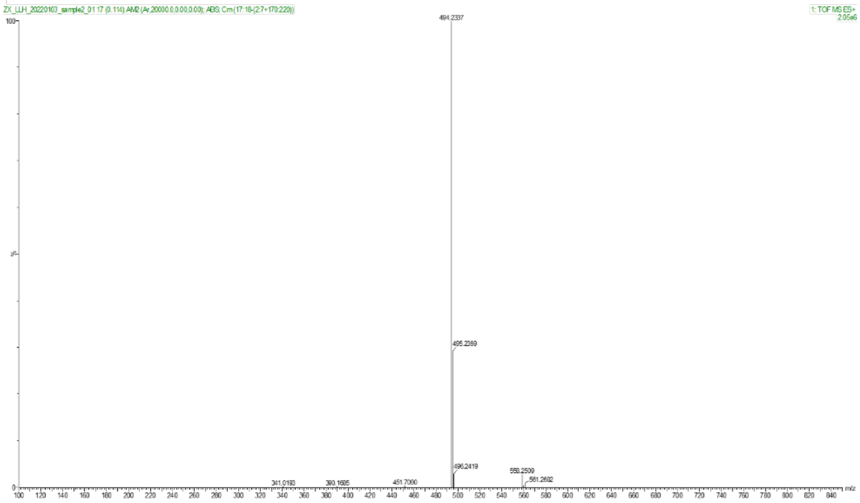

HRMS of A1

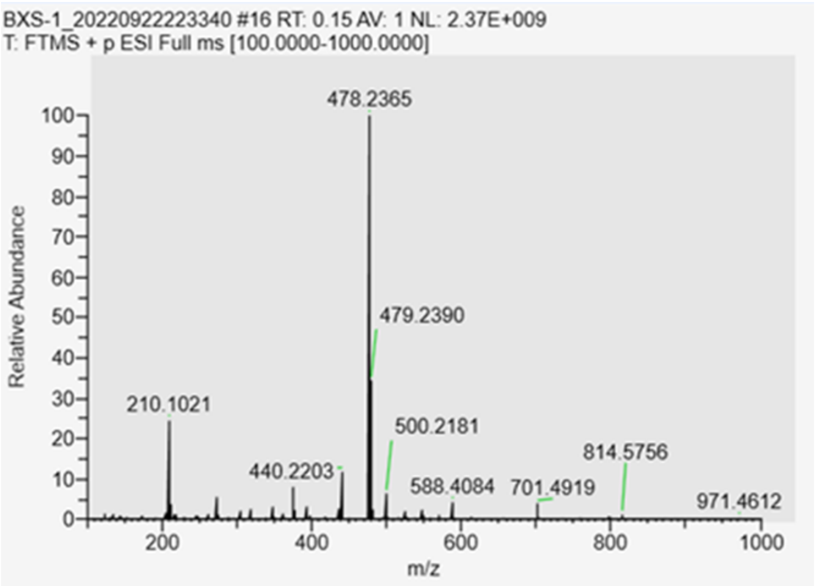


HRMS of A2

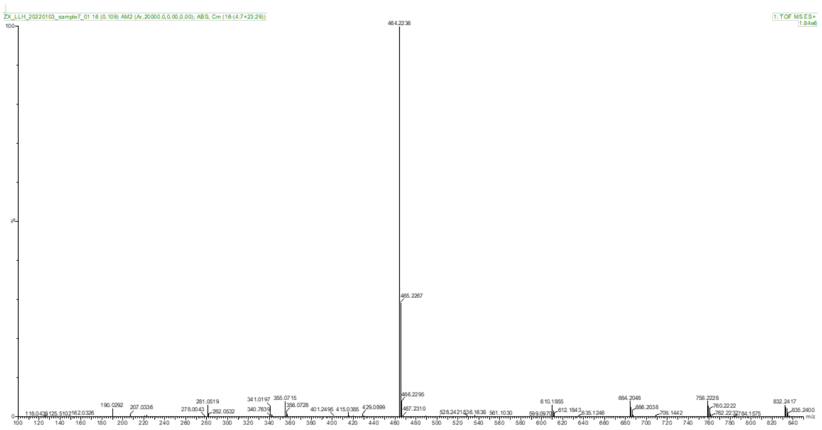

HRMS of A3

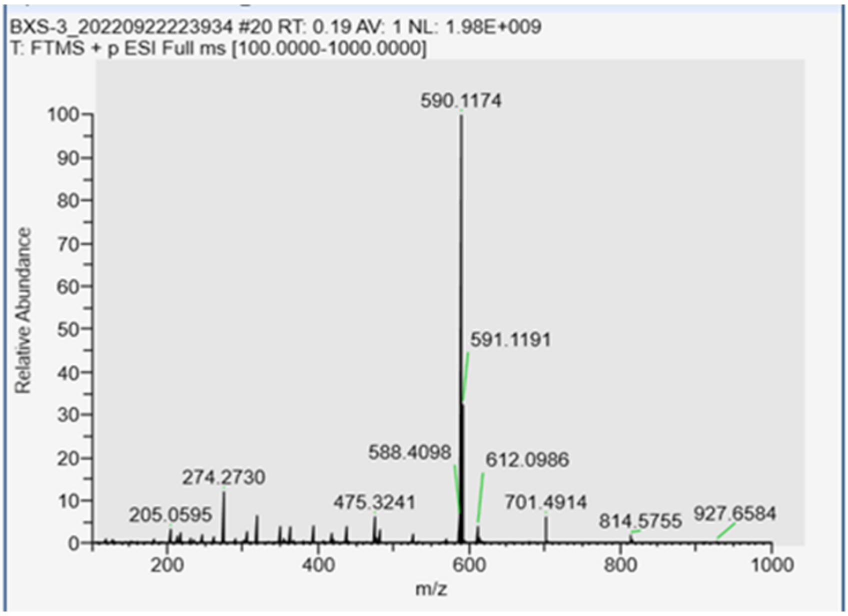


HRMS of A4

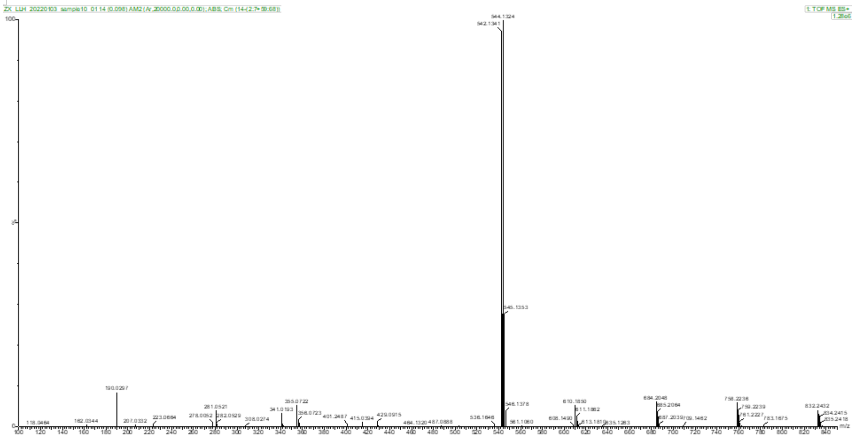

HRMS of A5

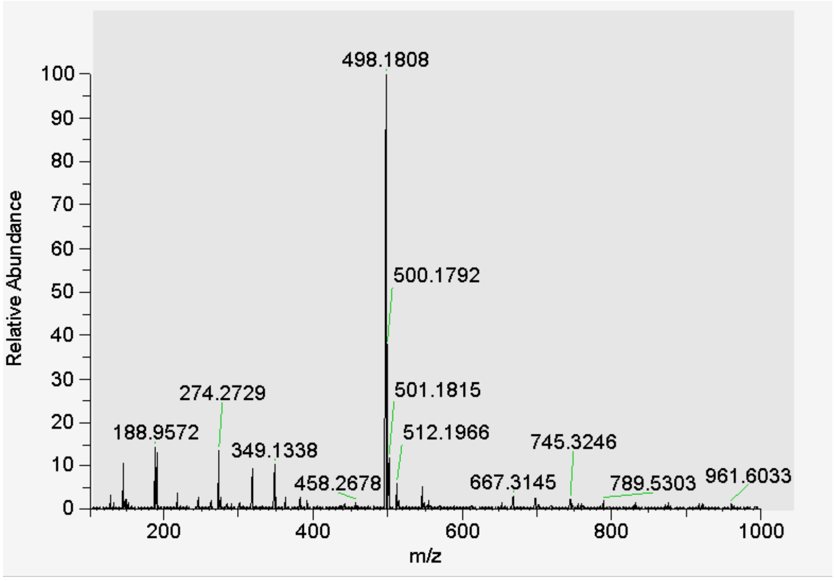


HRMS of A6

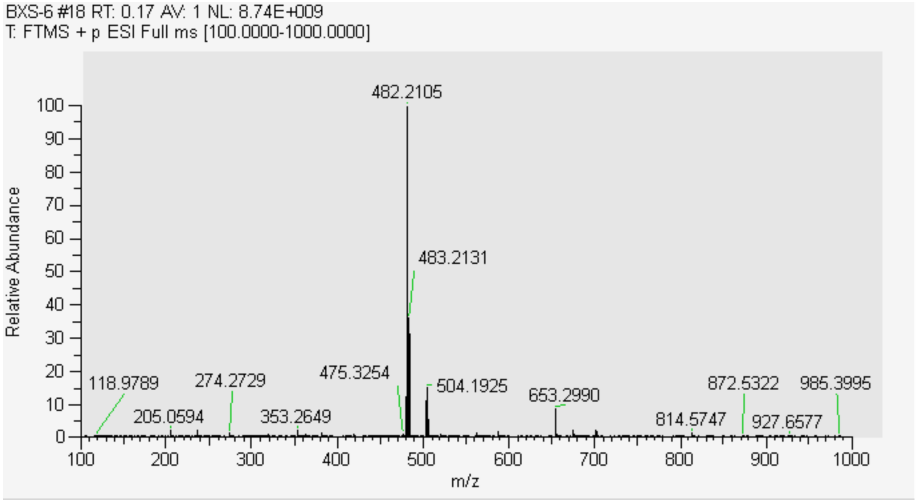


HRMS of A7

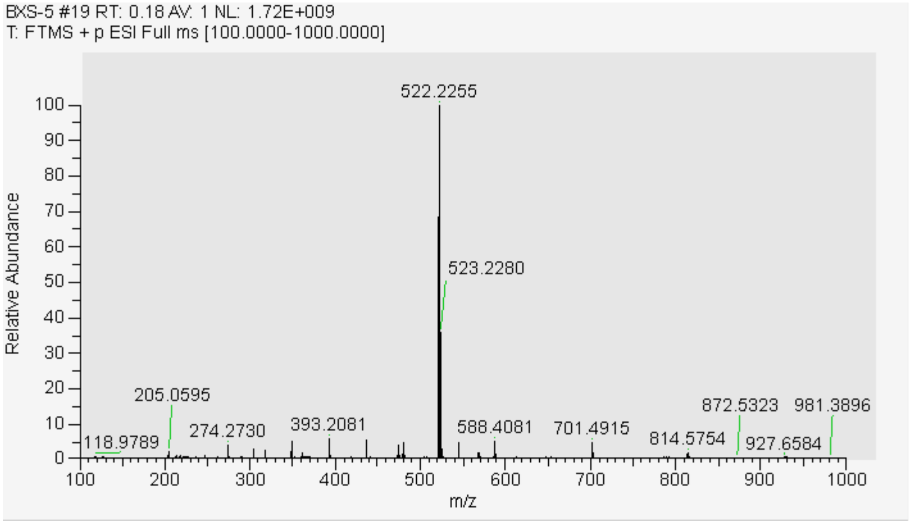


HRMS of A8

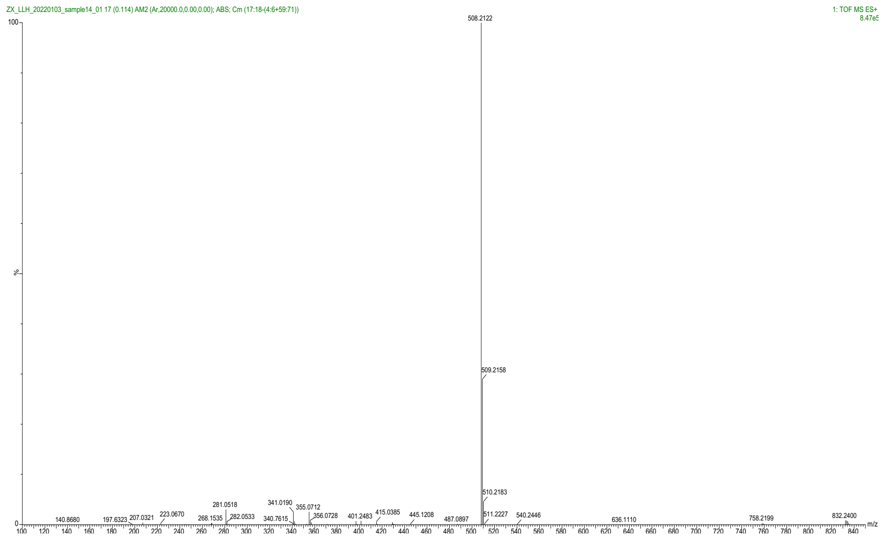


HRMS of A9

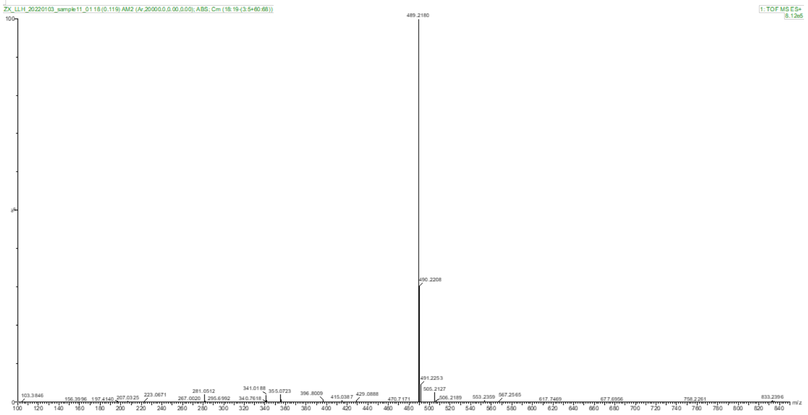


HRMS of A10

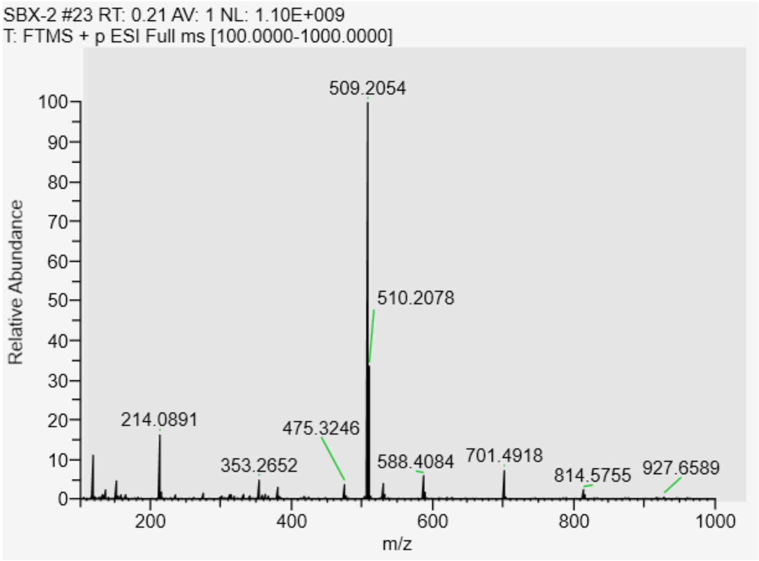


HRMS of A11

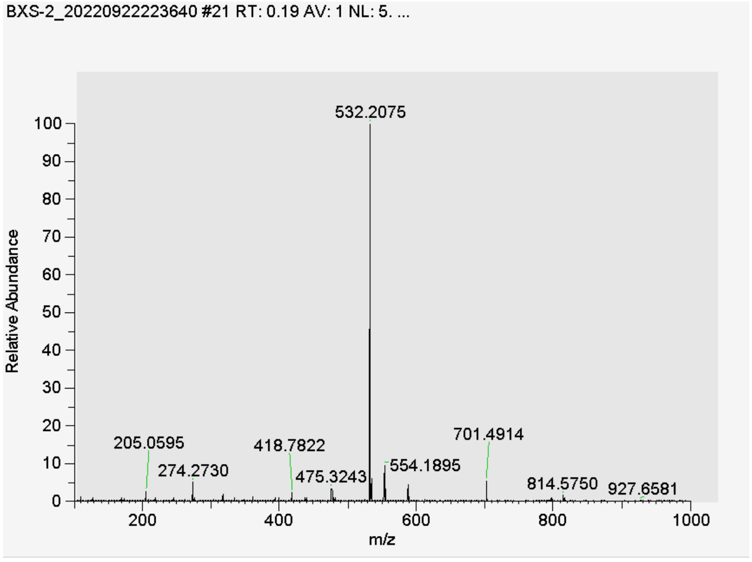


HRMS of A12

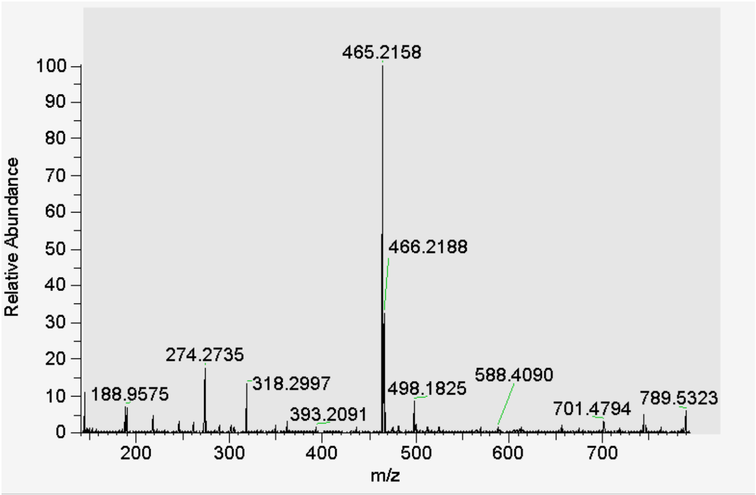


HRMS of B1

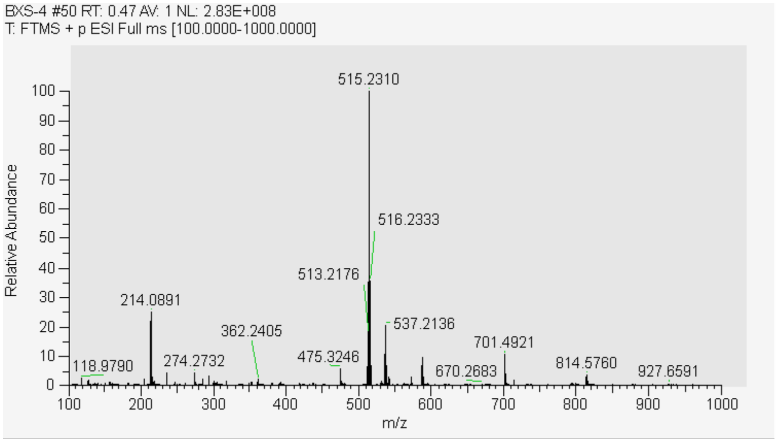


HRMS of B2

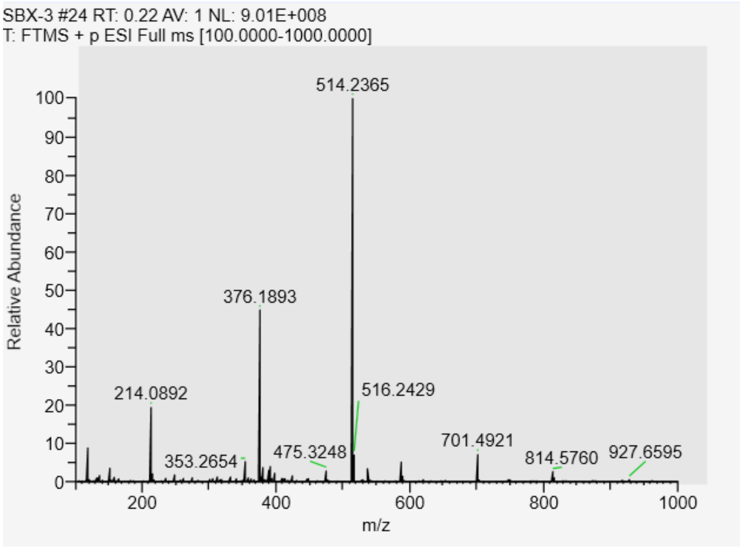


HRMS of B3

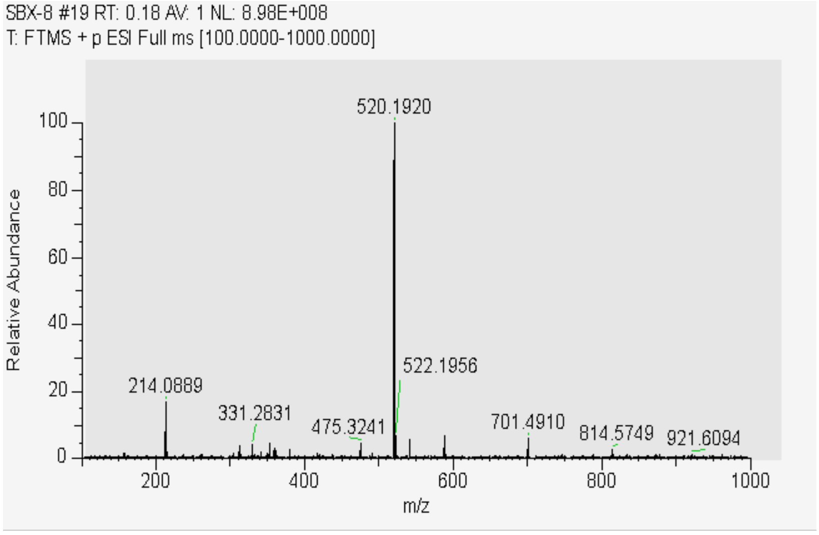


HRMS of B4

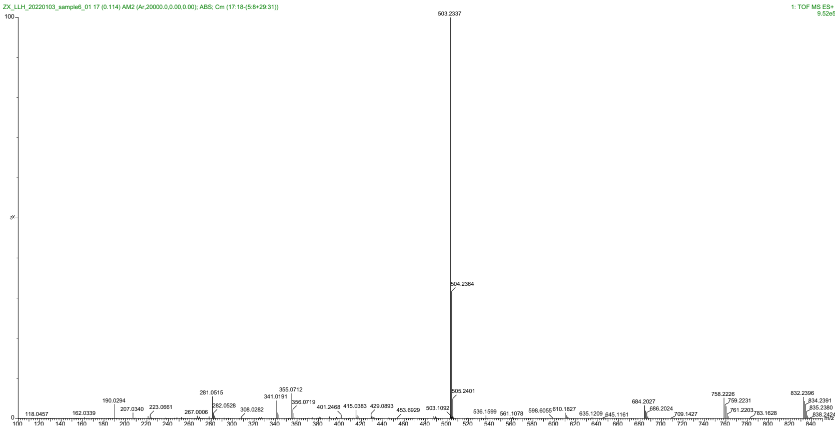


HRMS of B5

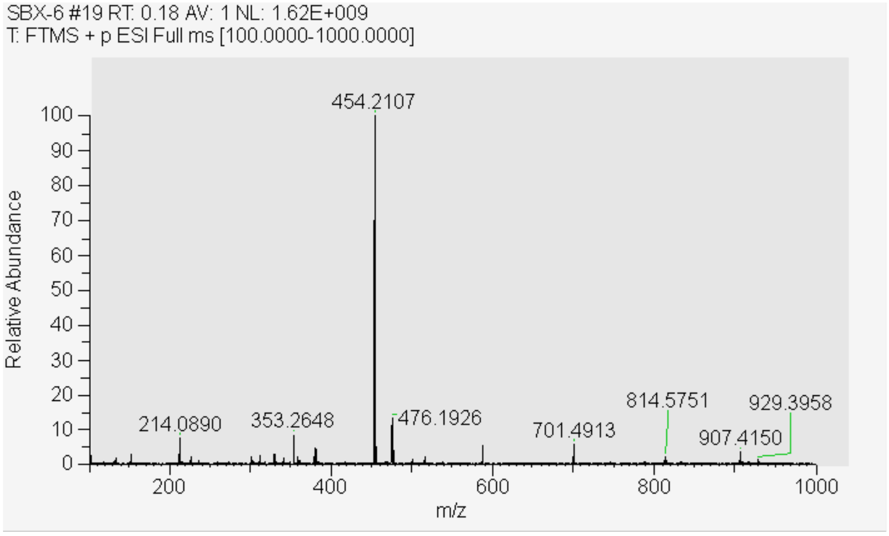


HRMS of B6

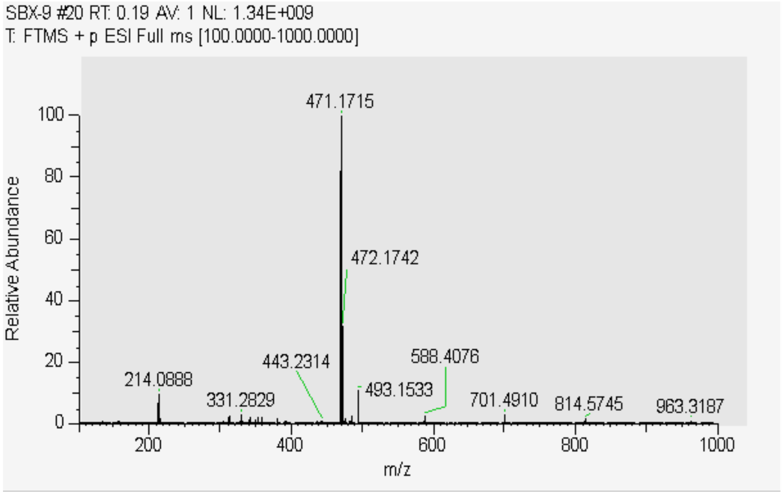


HRMS of B7

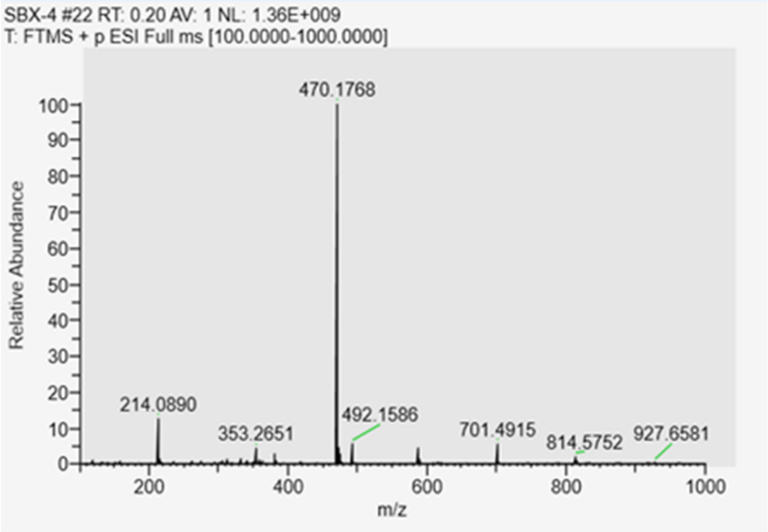


HRMS of B8

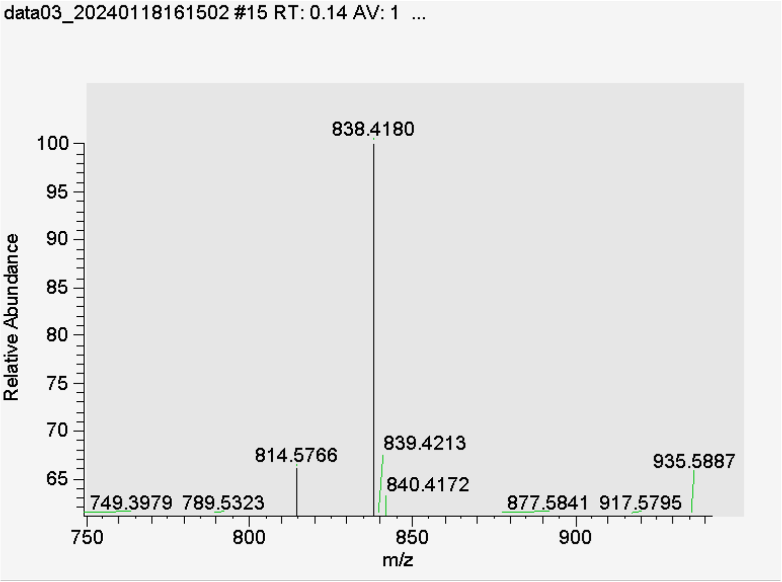


HRMS of A9-Halo

**NMR Spectra**

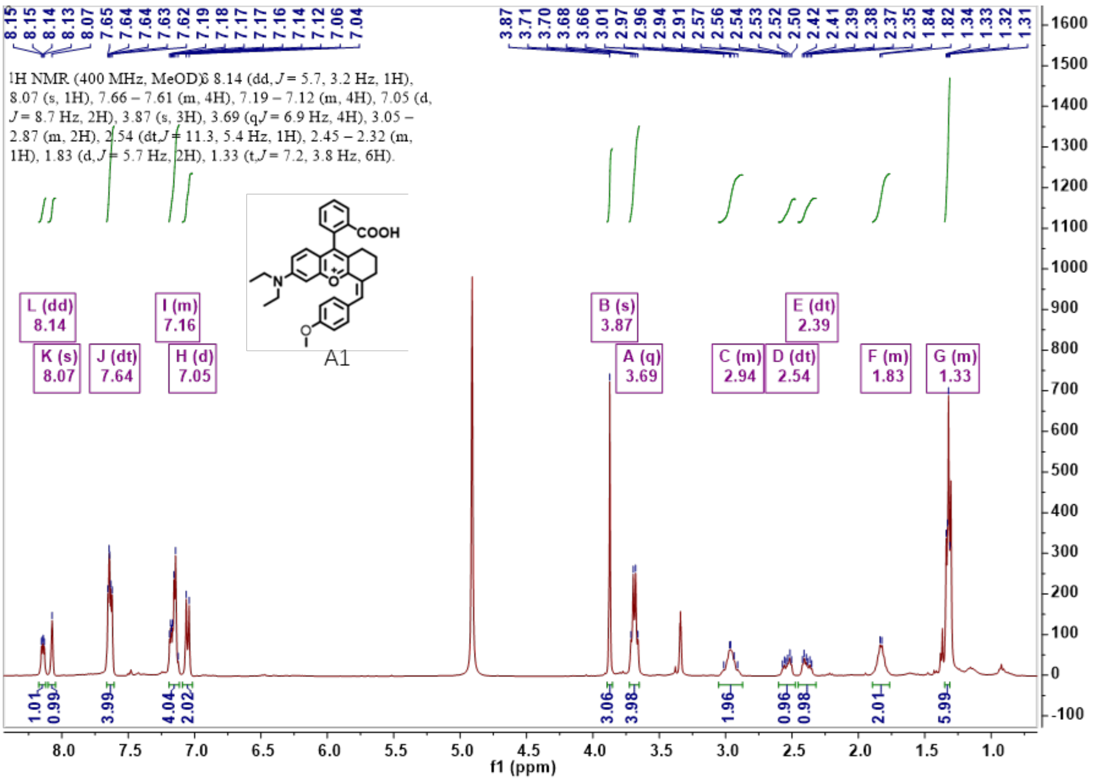


$^{1}$H NMR Spectra of A1 (MeOD)

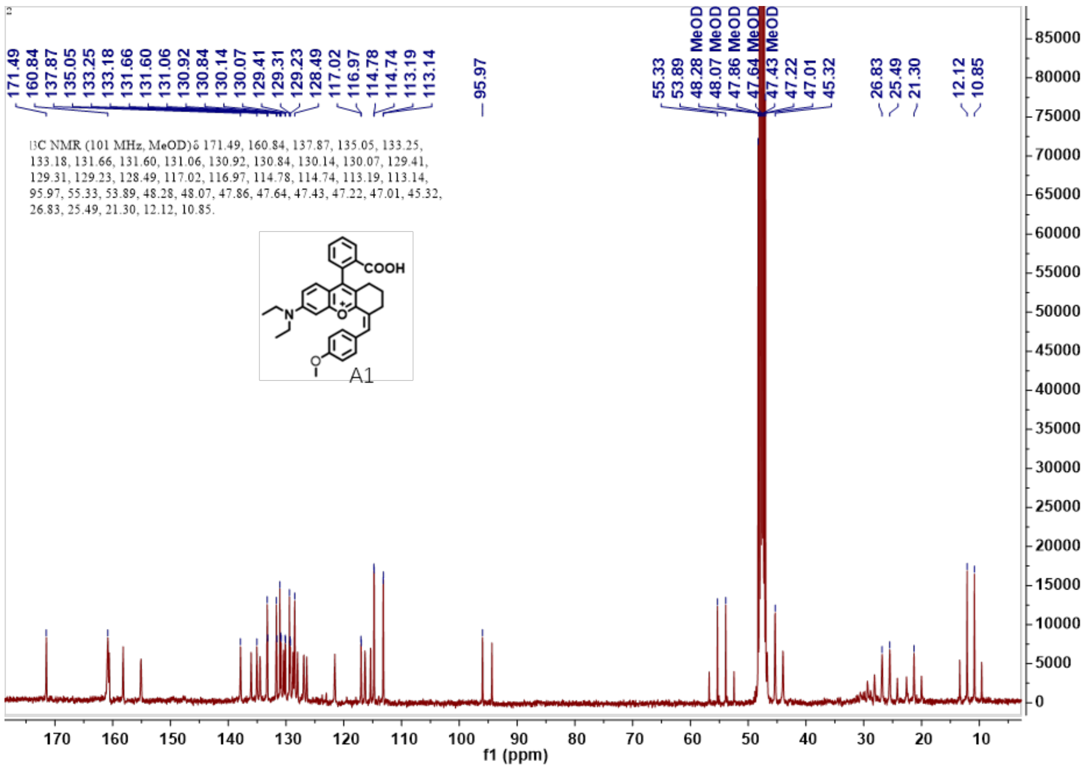


$^{13}$C NMR Spectra of A1 (MeOD)

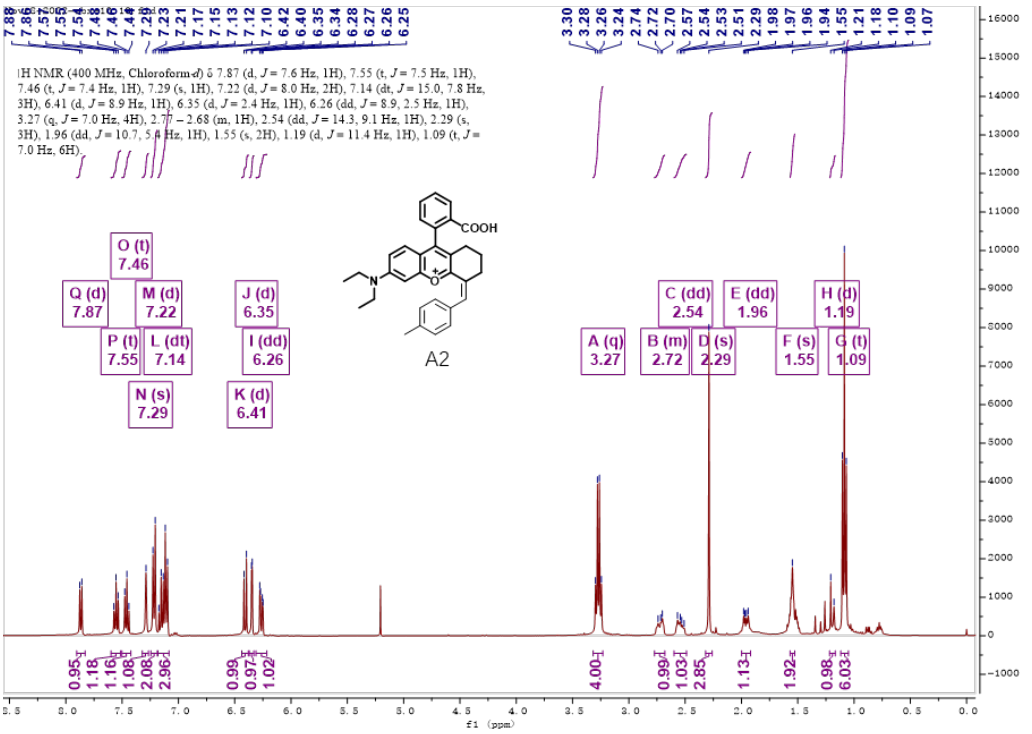


$^{1}$H NMR Spectra of A2 ($CDCl_3$)

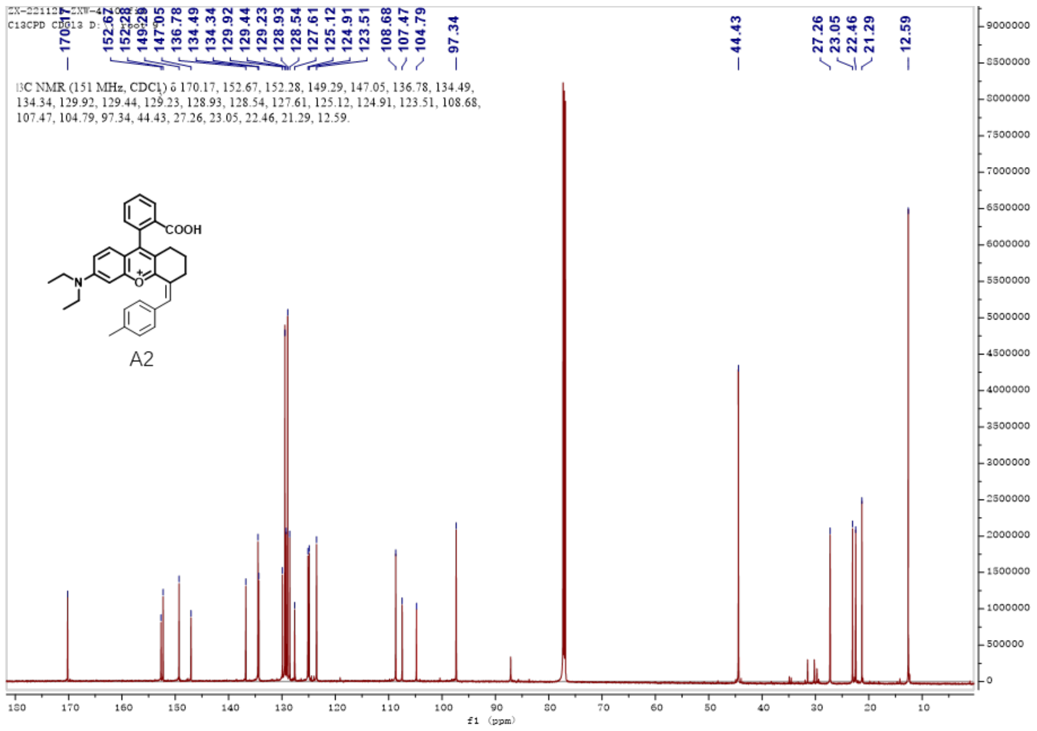


$^{13}$C NMR Spectra of A2 ($CDCl_3$)

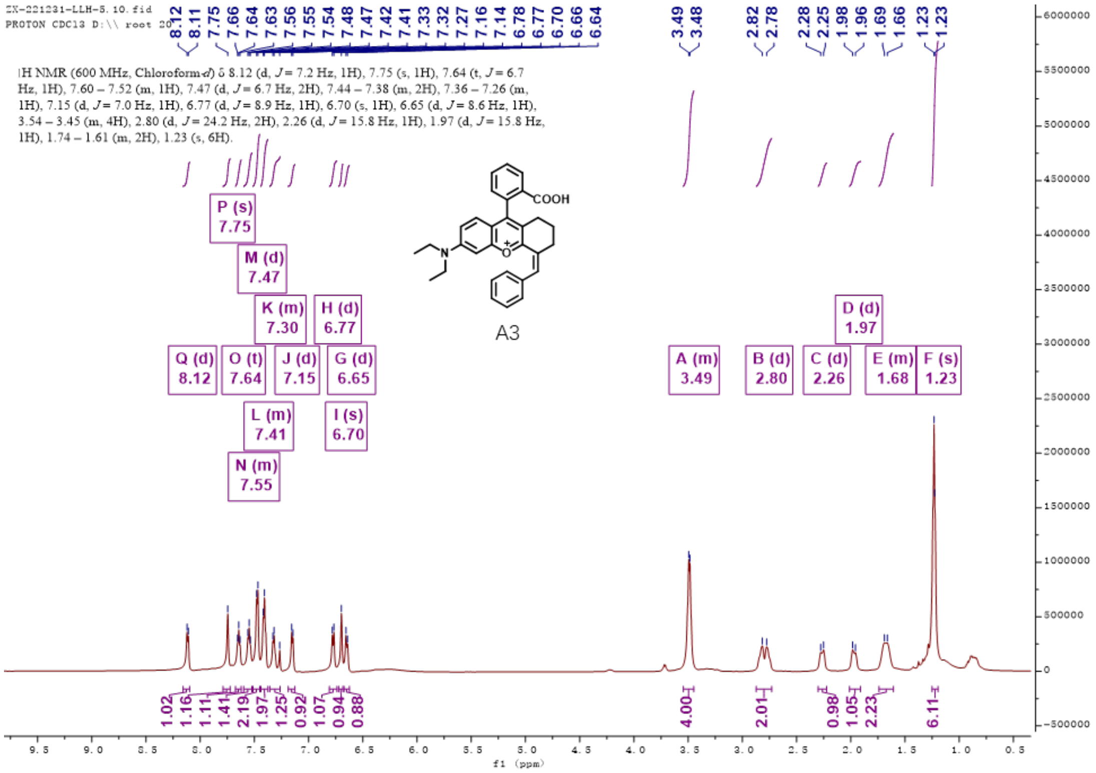


$^{1}$H NMR Spectra of A3 ($CDCl_3$)

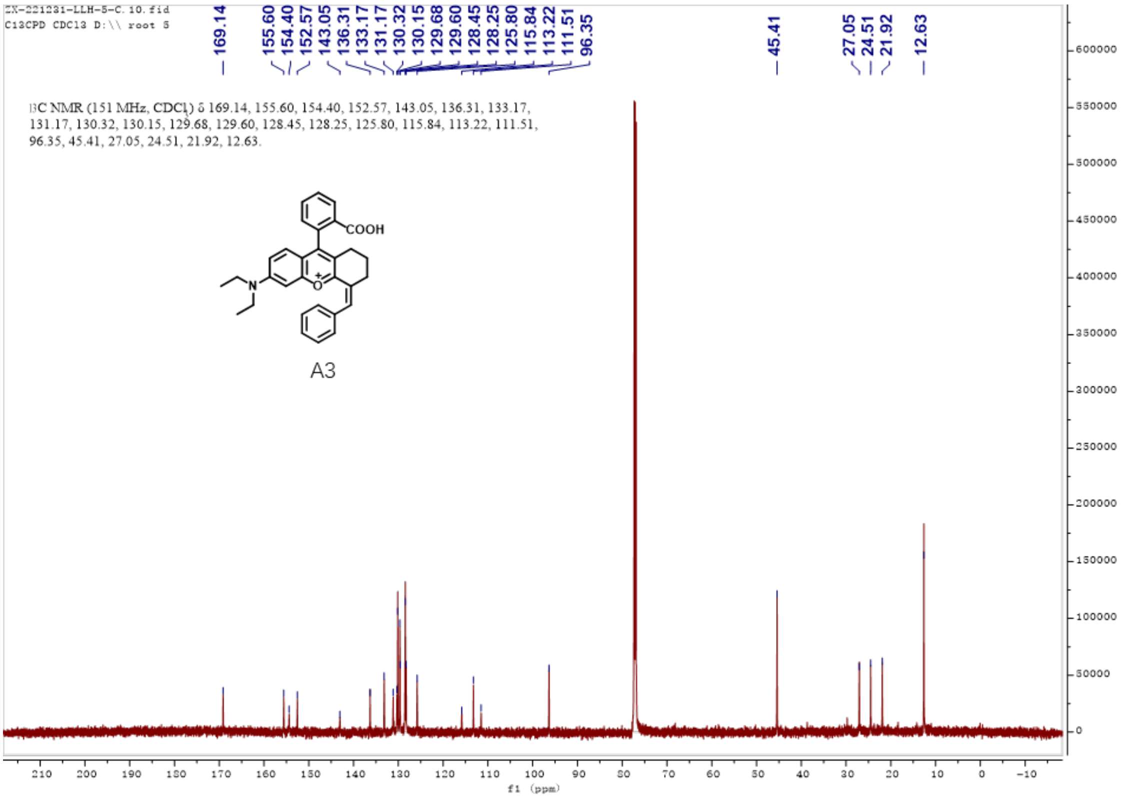


$^{13}$C NMR Spectra of A3 ($CDCl_3$)

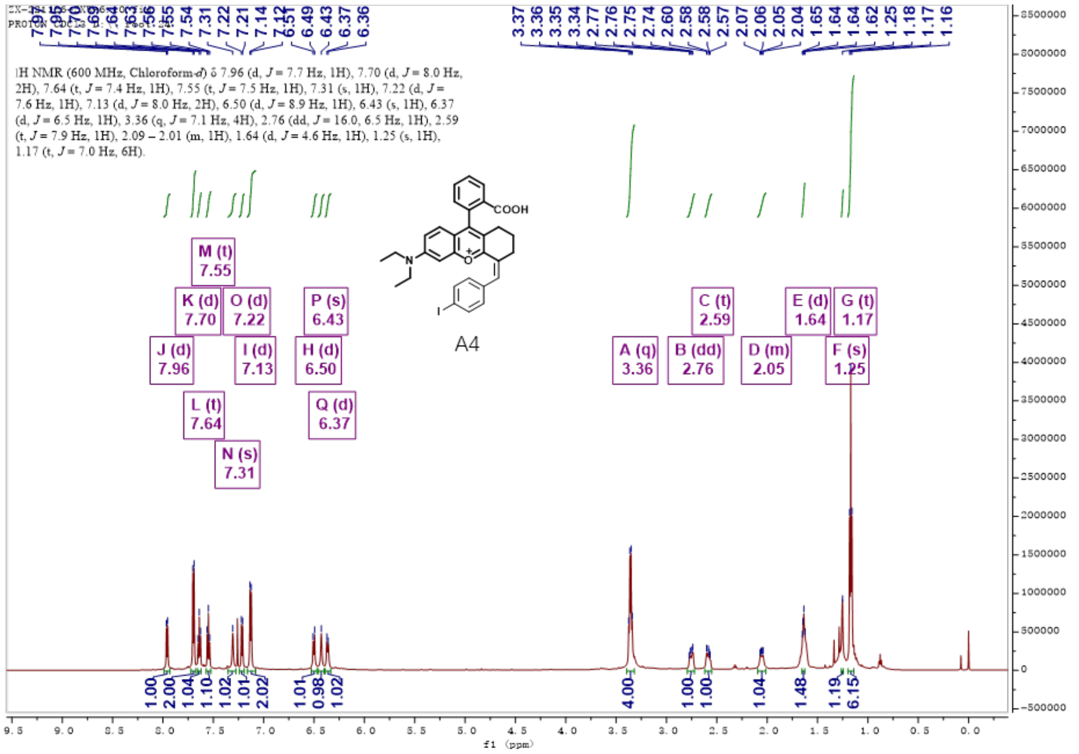


$^{1}$H NMR Spectra of A4 ($CDCl_3$)

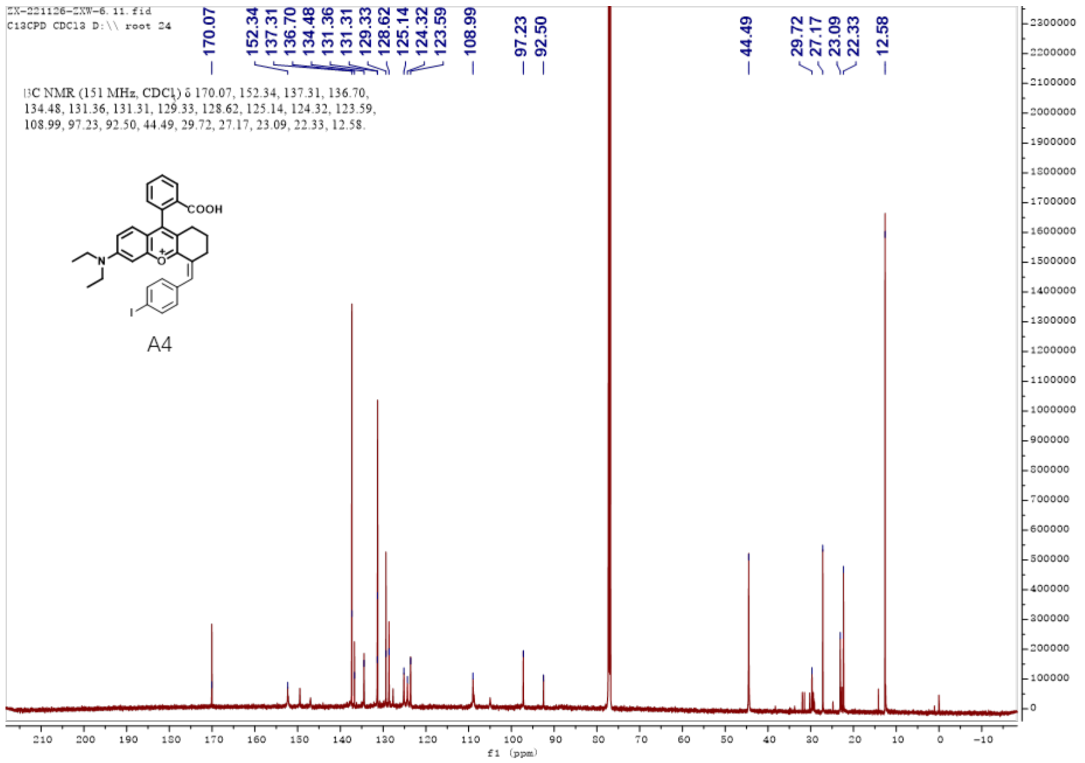


$^{13}$C NMR Spectra of A4 ($CDCl_3$)

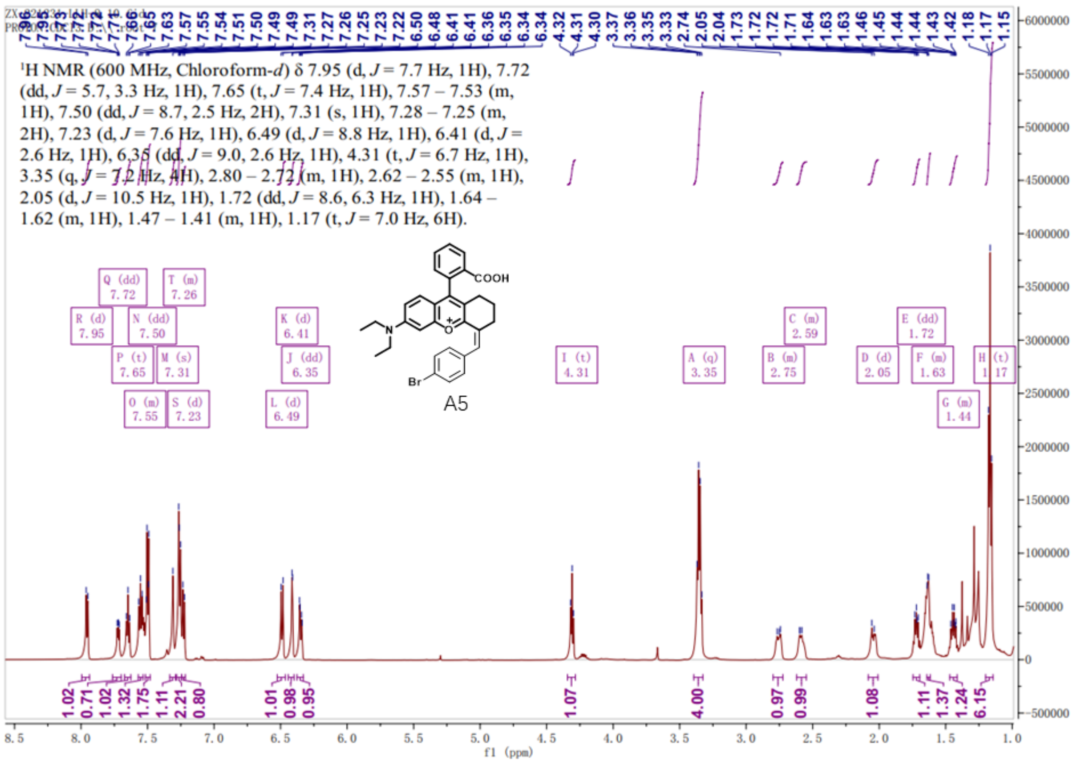


$^{1}H$ NMR Spectra of A5 ($CDCl_3$)

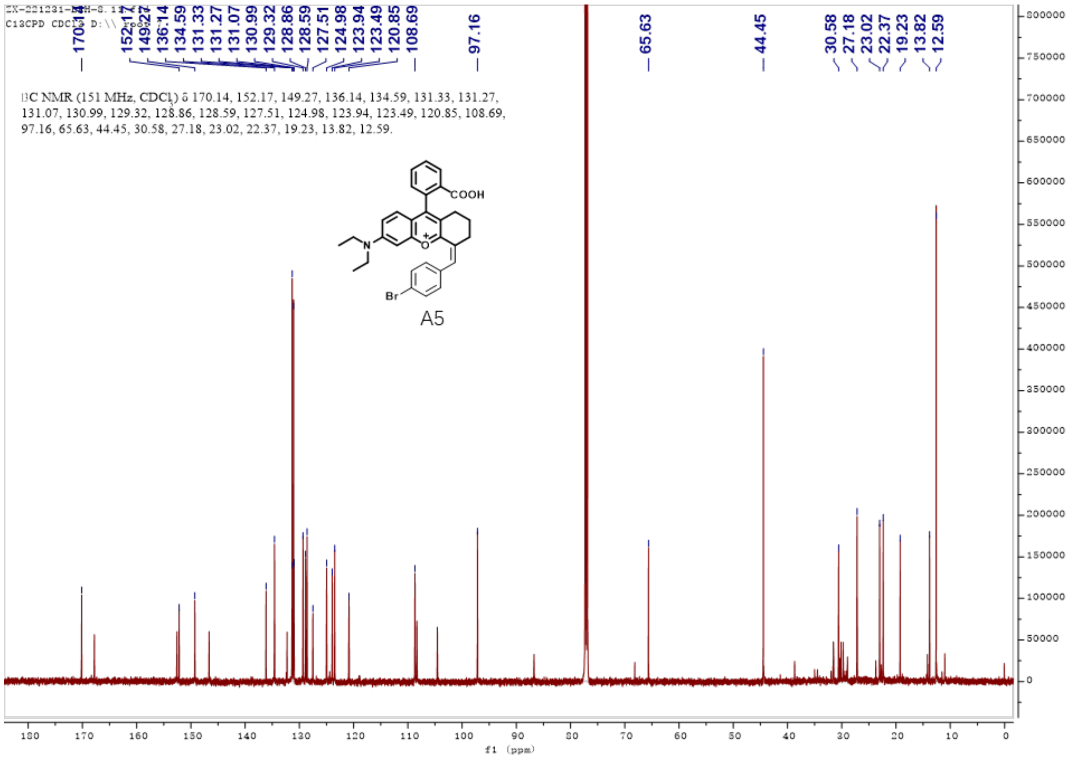


$^{13}C$ NMR Spectra of A5 ($CDCl_3$)

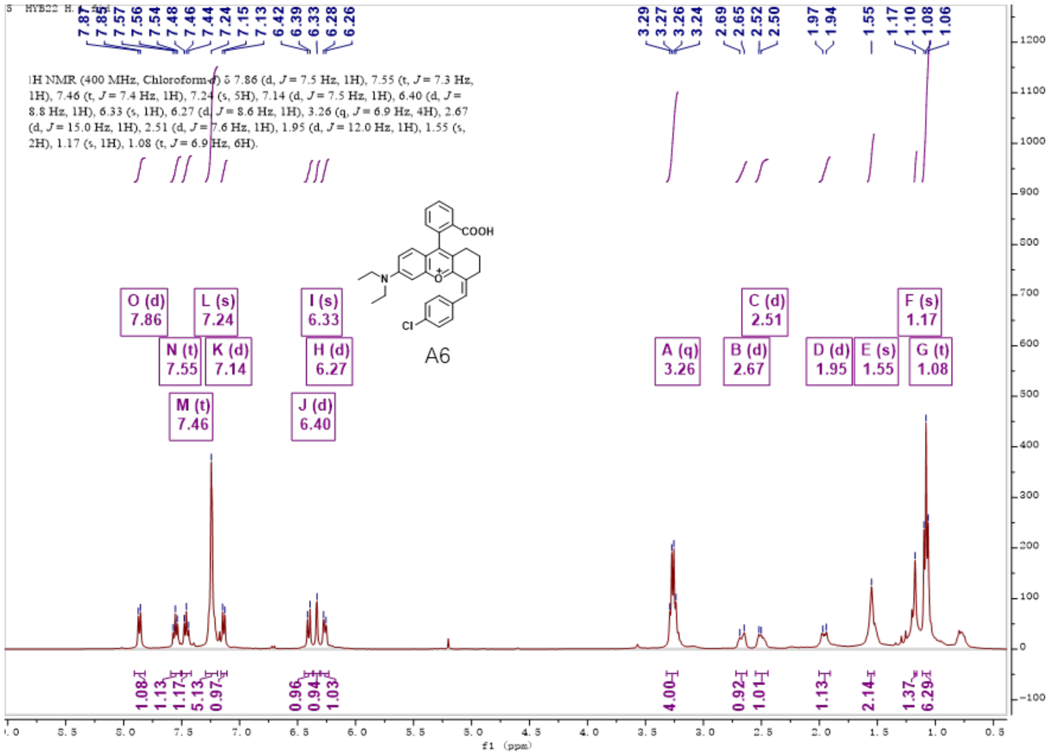


$^{1}$H NMR Spectra of A6 ($CDCl_3$)

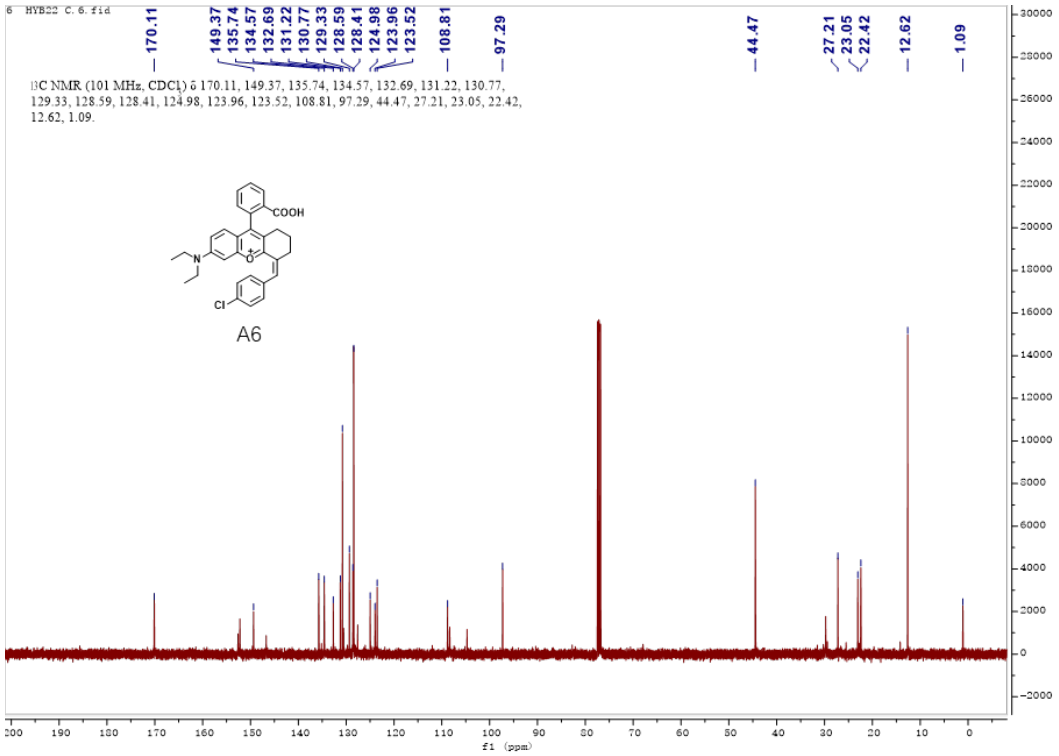


$^{13}$C NMR Spectra of A6 ($CDCl_3$)

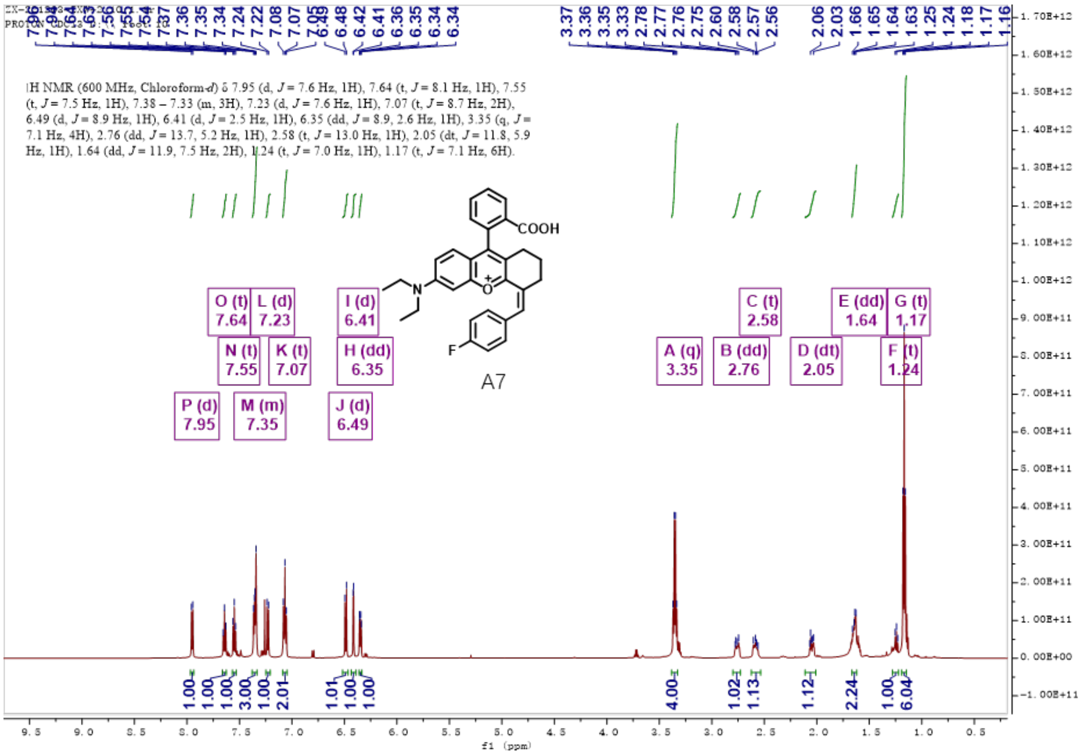


$^{1}$H NMR Spectra of A7 ($CDCl_3$)

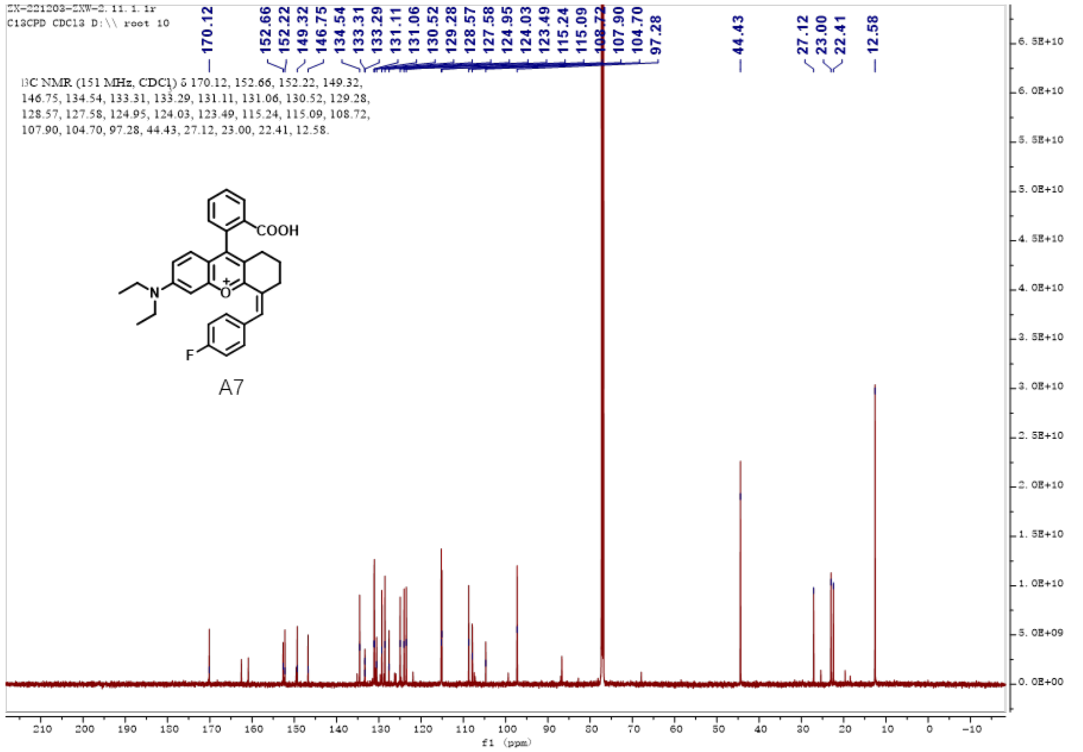


$^{13}$C NMR Spectra of A7 ($CDCl_3$)

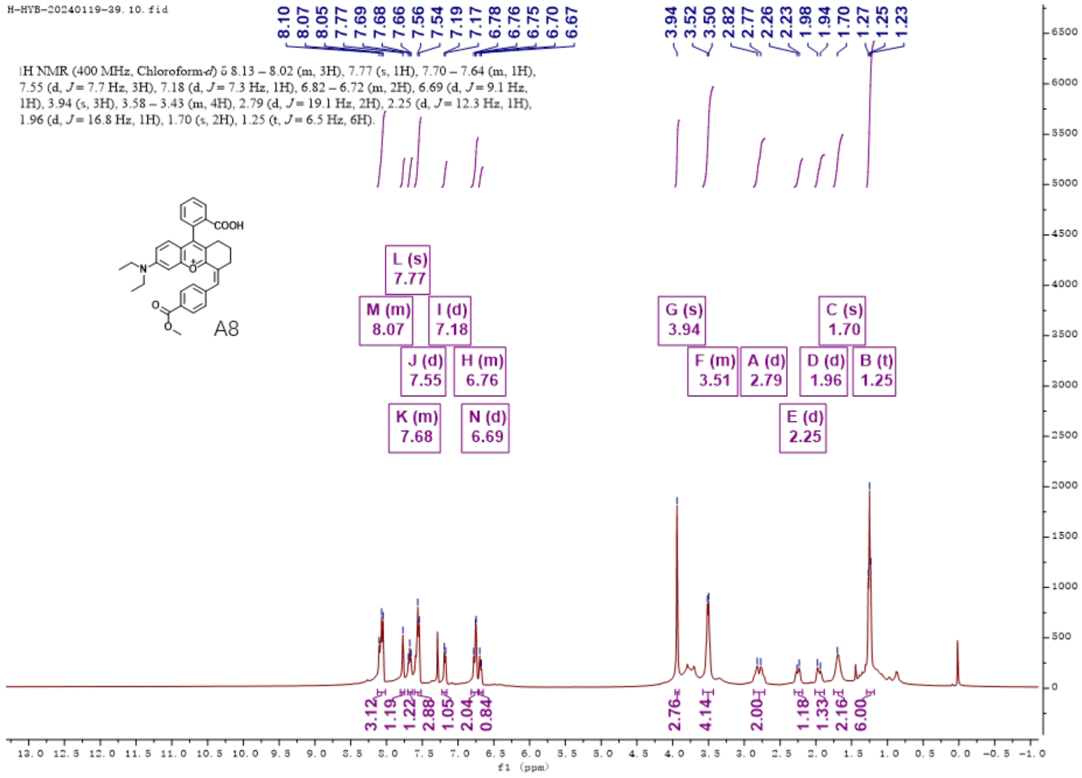


$^{1}$H NMR Spectra of A8 (MeOD)

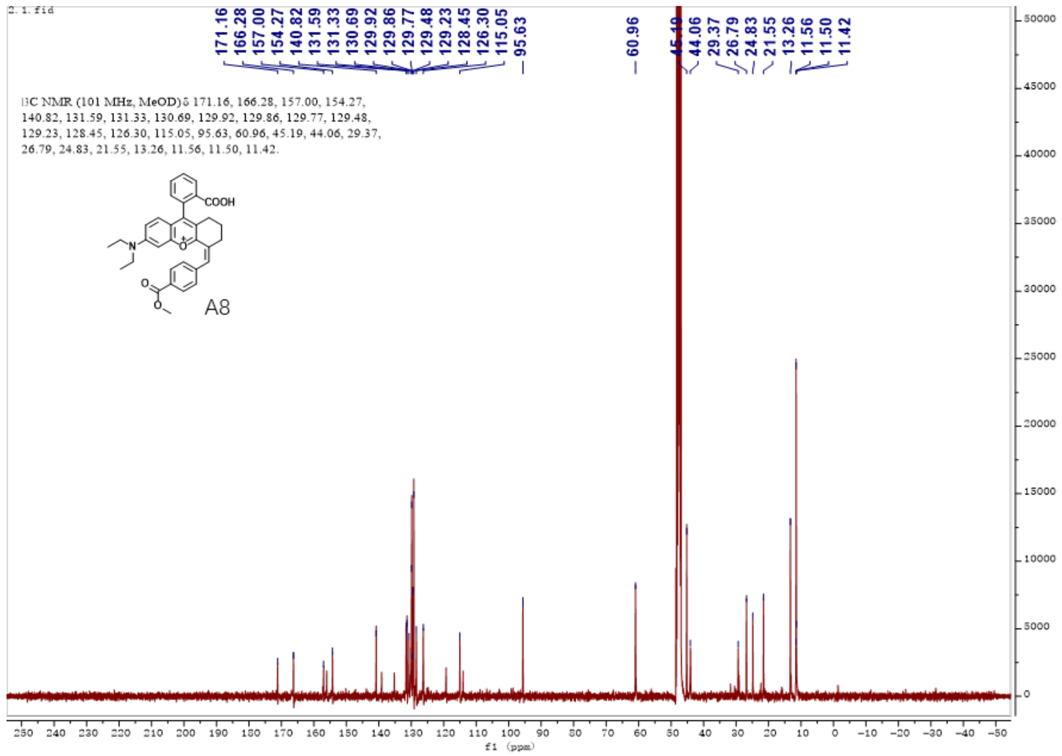


$^{13}$C NMR Spectra of A8 (MeOD)

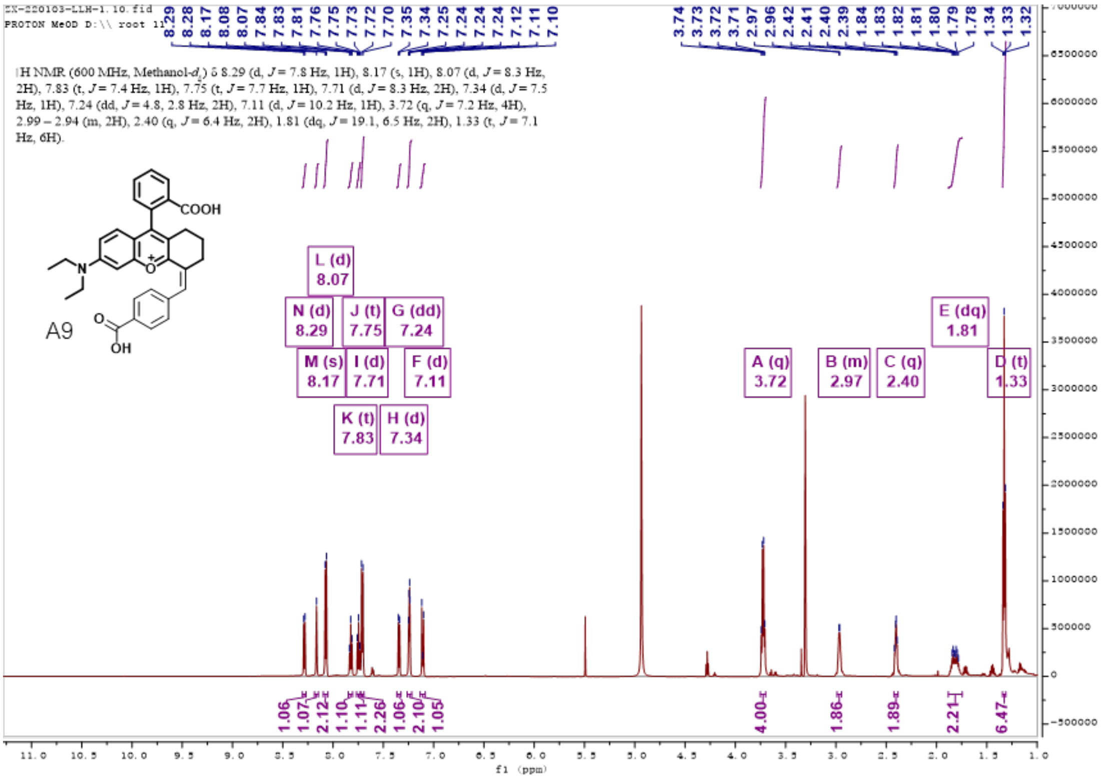


$^{1}$H NMR Spectra of A9 (MeOD)

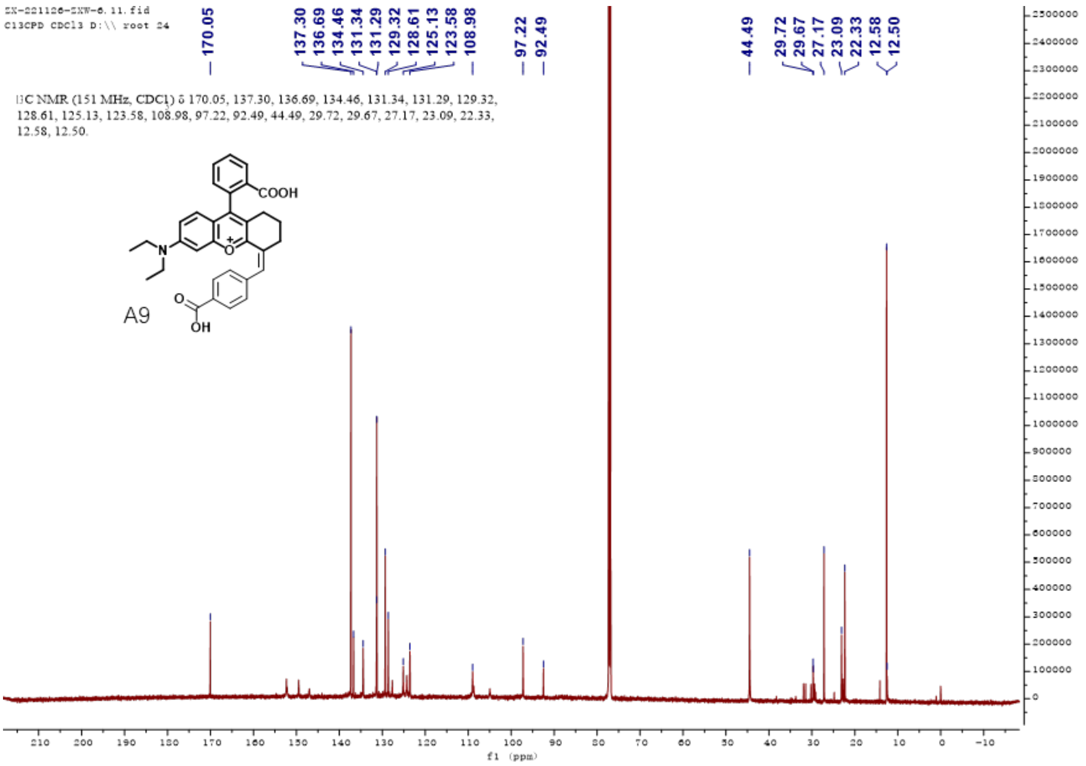


$^{13}$C NMR Spectra of A9 ($CDCl_3$)

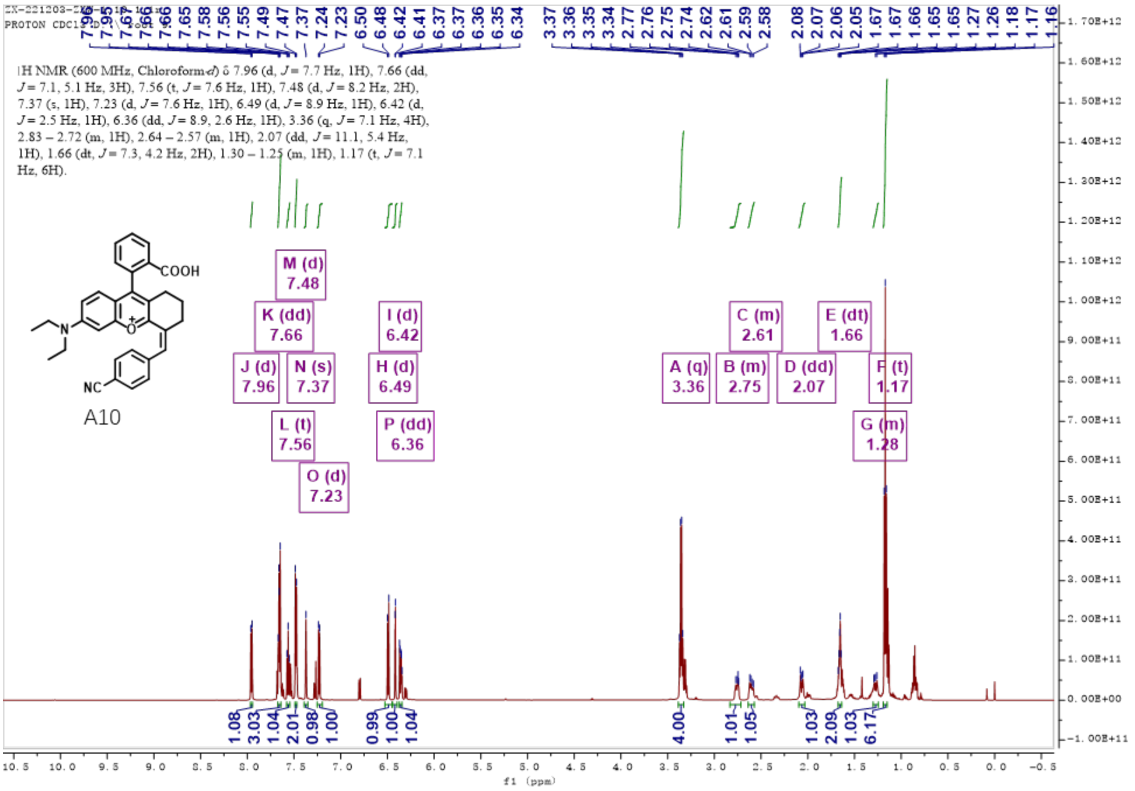


$^{1}$H NMR Spectra of A10 ($CDCl_3$)

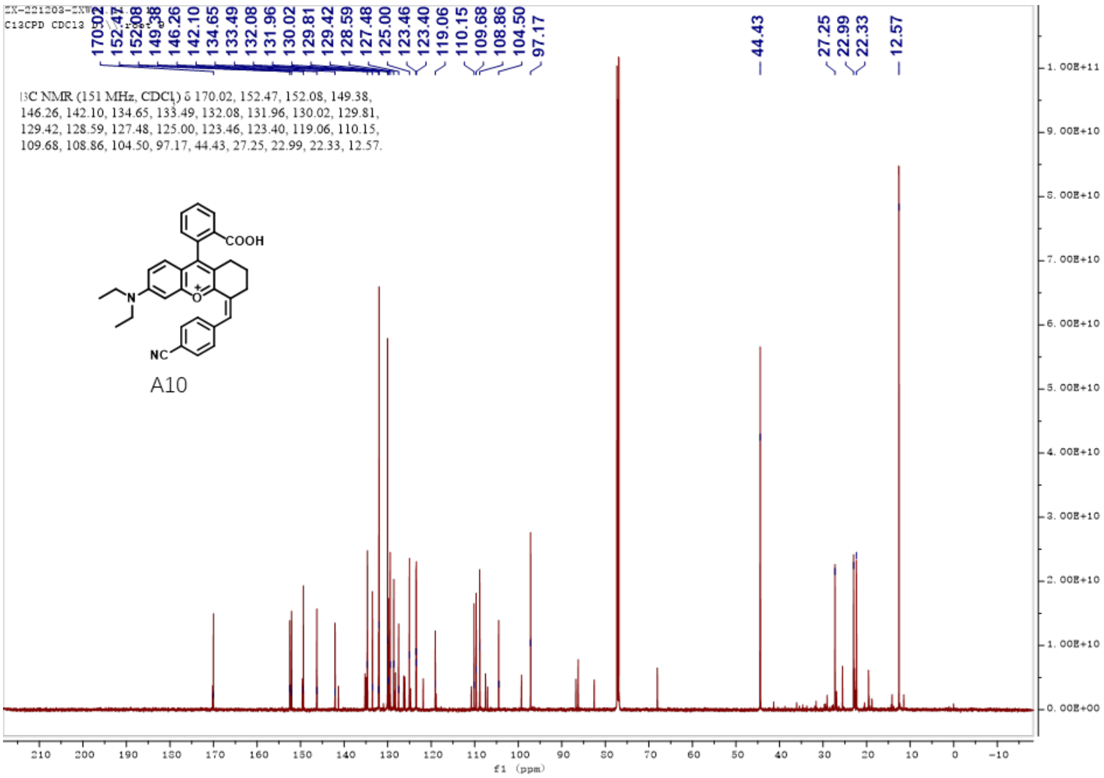


$^{13}$C NMR Spectra of A10 ($CDCl_3$)

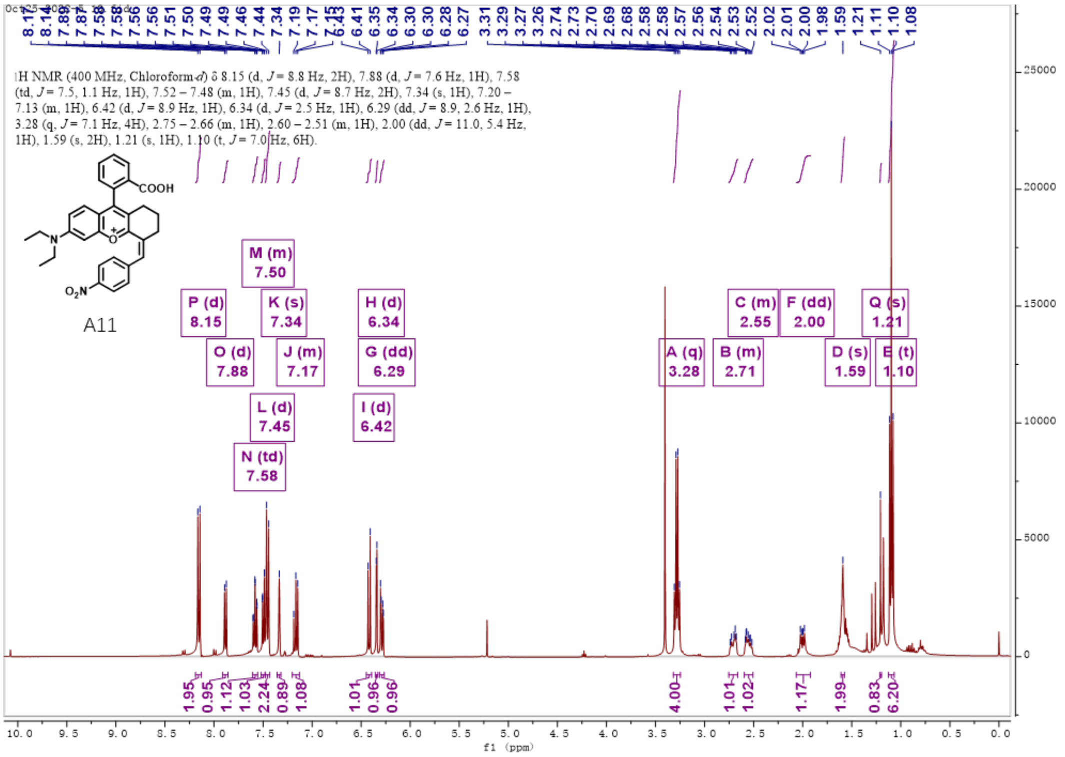

$^{1}$H NMR Spectra of A11 ($CDCl_3$)

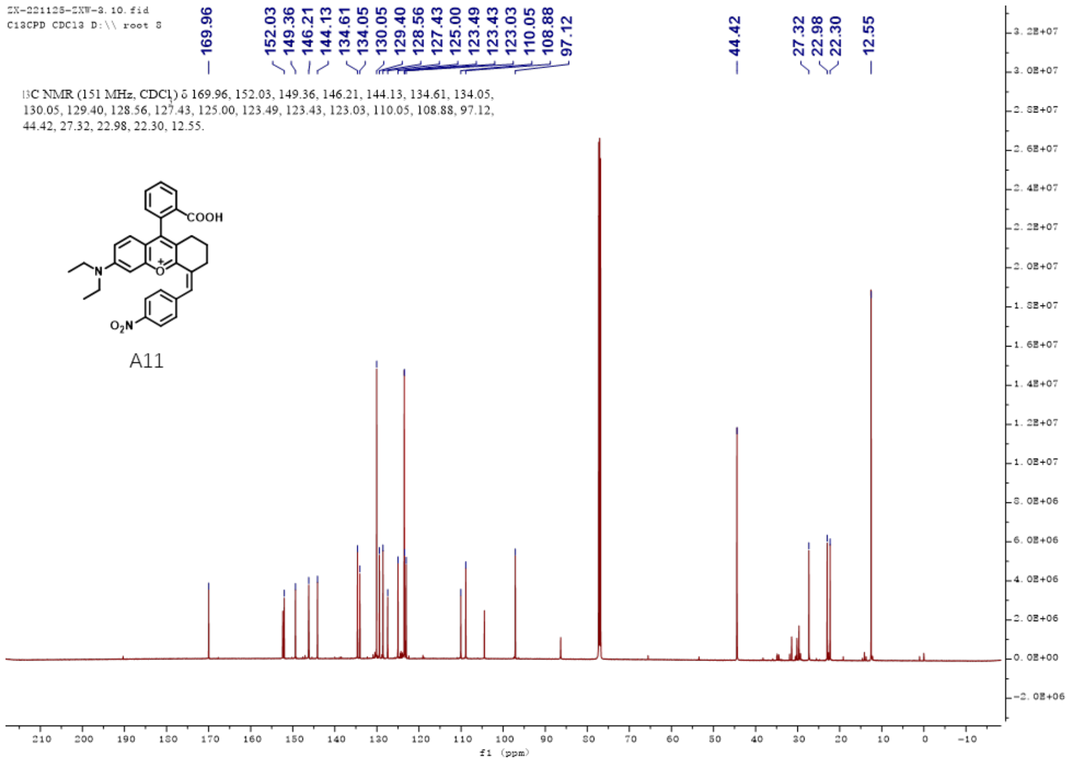

$^{13}$C NMR Spectra of A11 ($CDCl_3$)

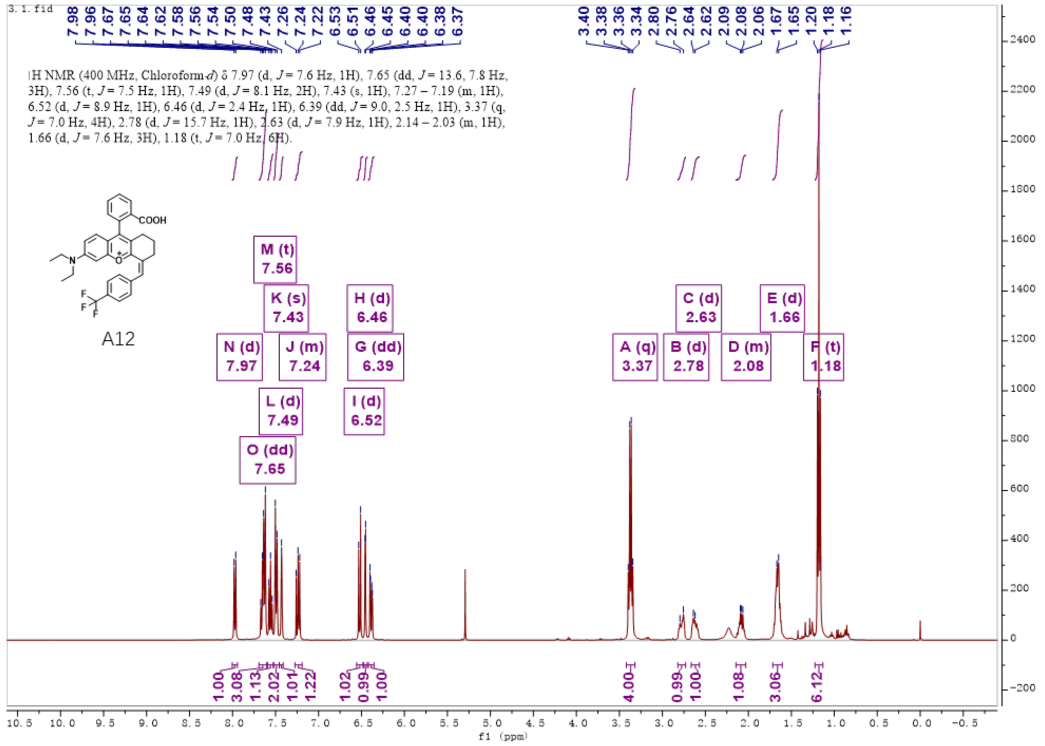


$^{1}$H NMR Spectra of A12 ($CDCl_3$)

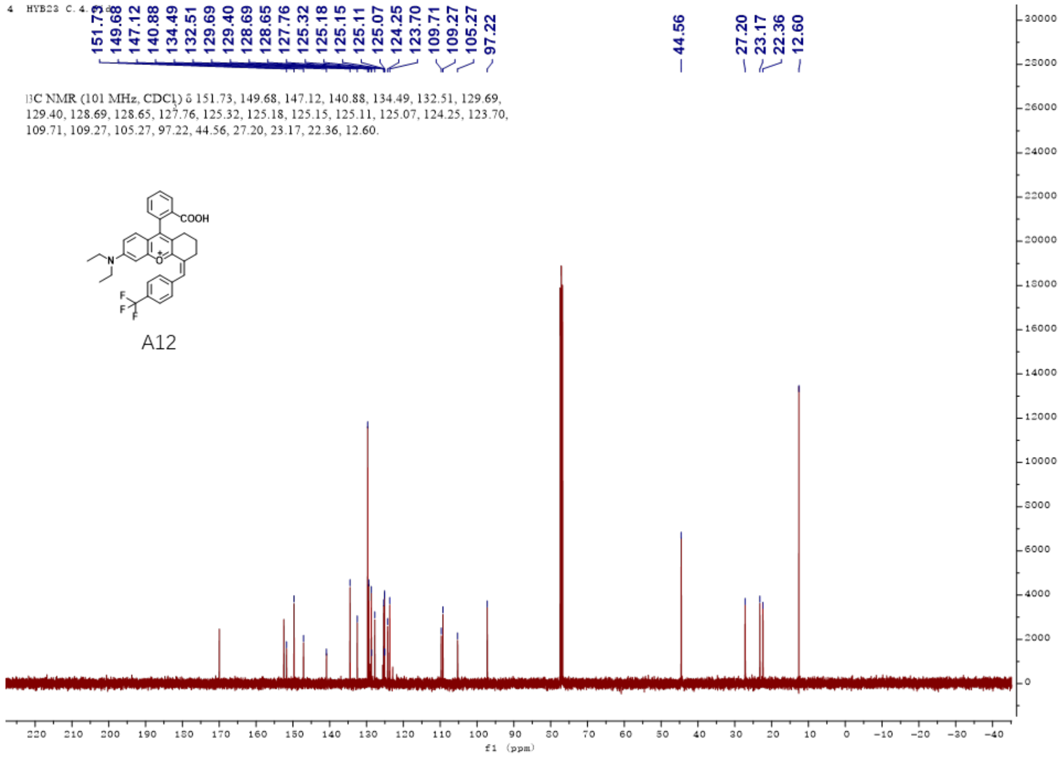


$^{13}$C NMR Spectra of A12 ($CDCl_3$)

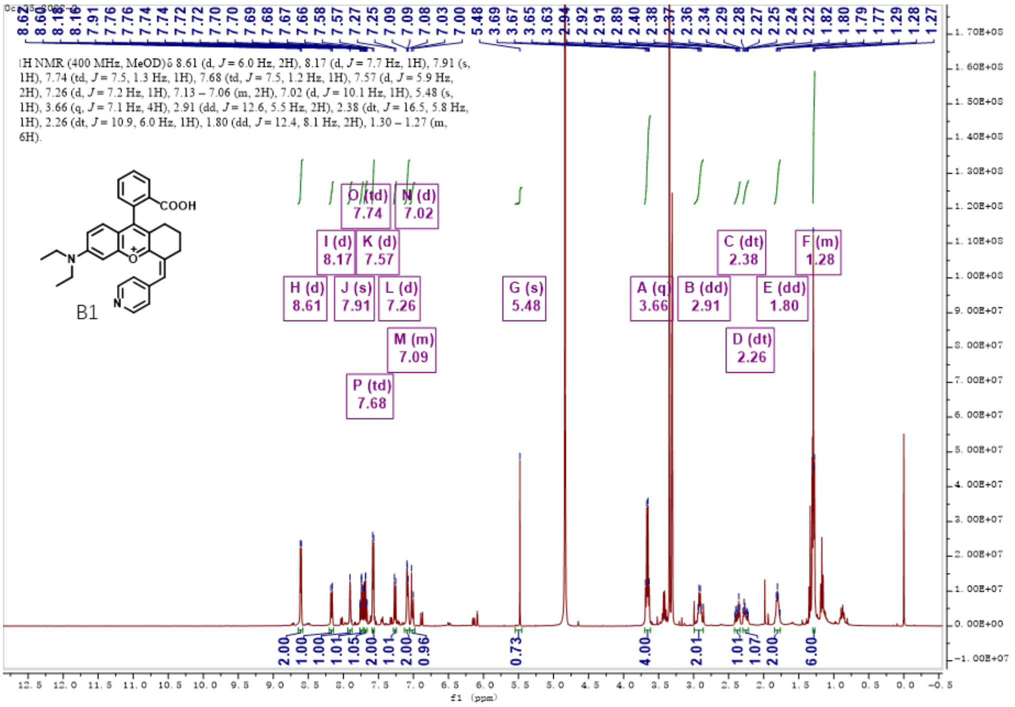


$^{1}$H NMR Spectra of B1 (MeOD)

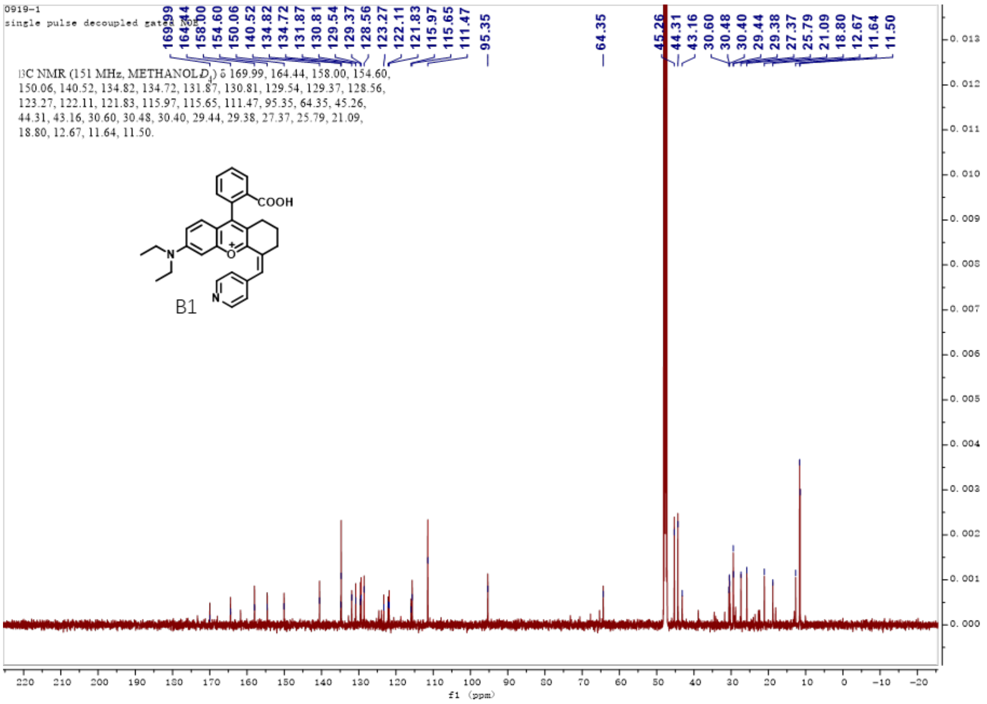


$^{13}$C NMR Spectra of B1 (MeOD)

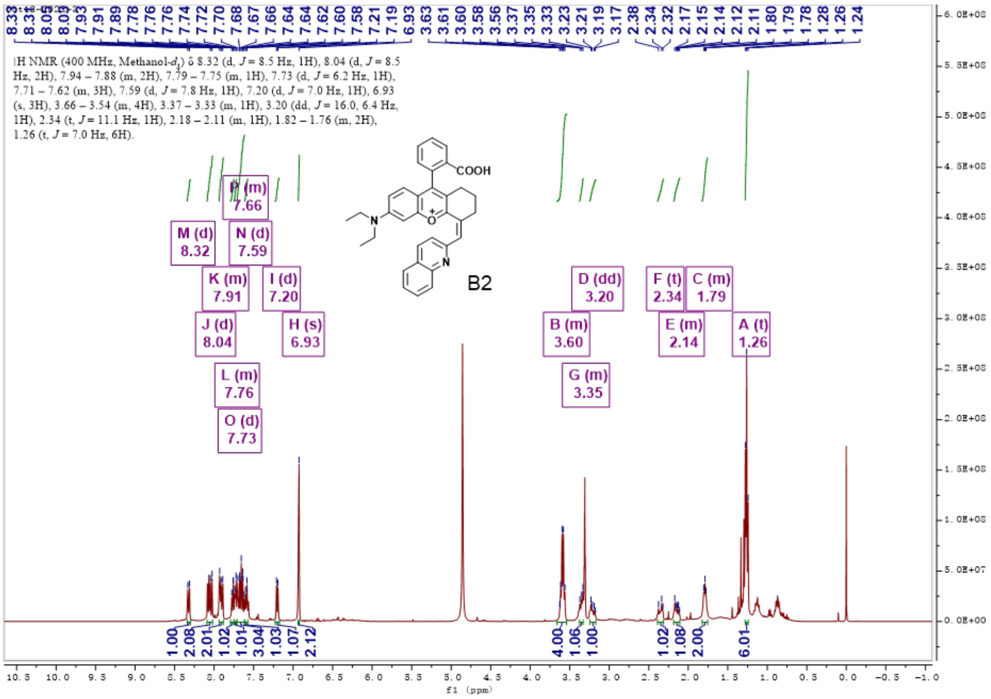


$^{1}$H NMR Spectra of B2 (MeOD)

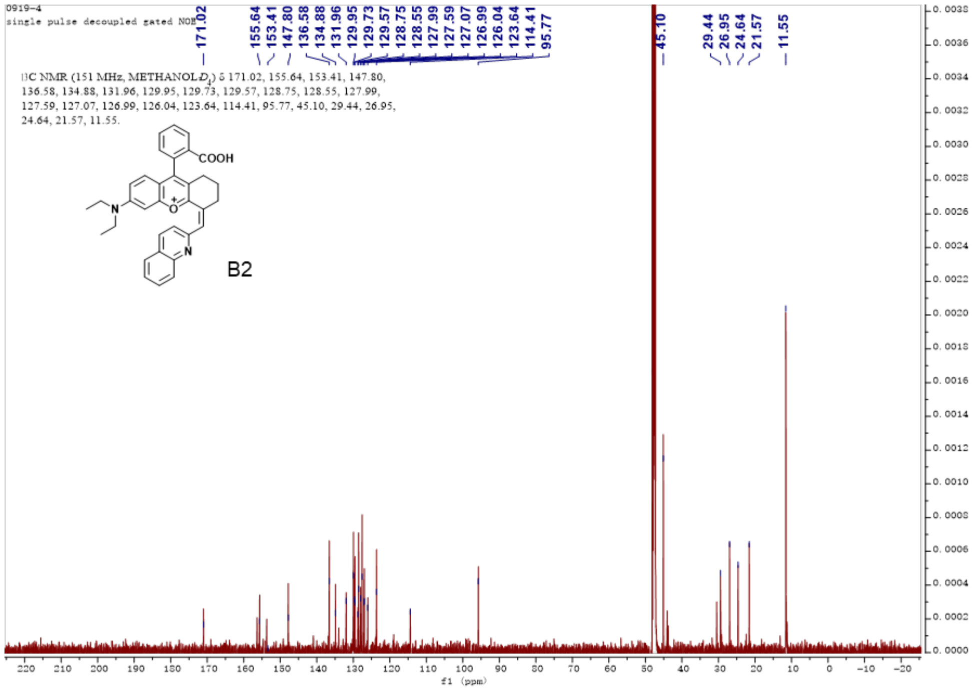


$^{13}$C NMR Spectra of B2 (MeOD)

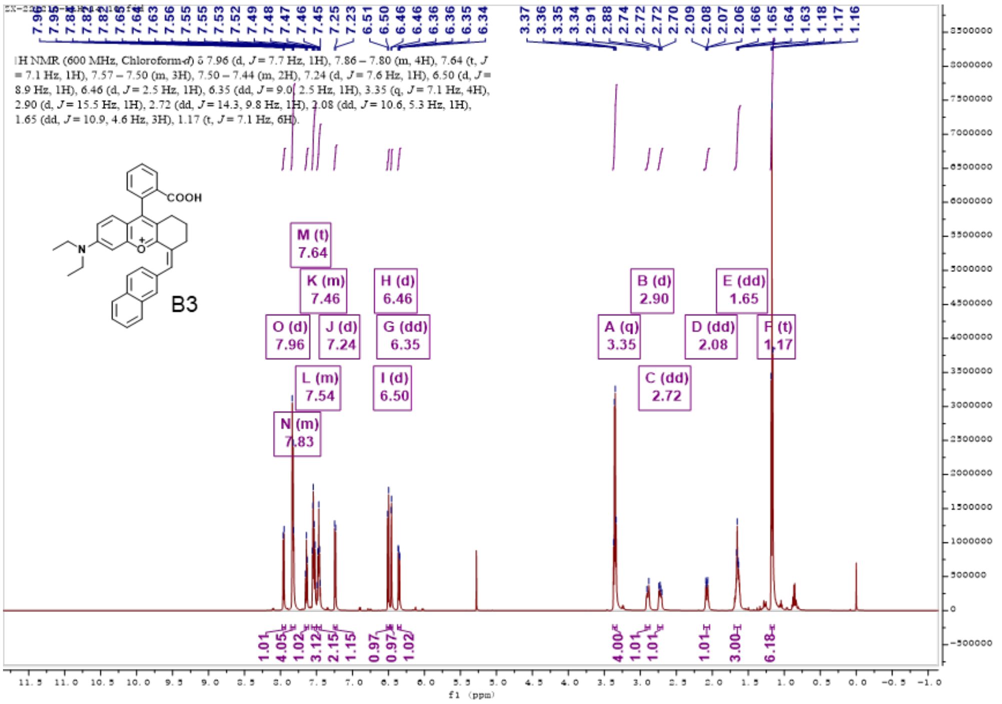


$^{1}$H NMR Spectra of B3 ($CDCl_3$)

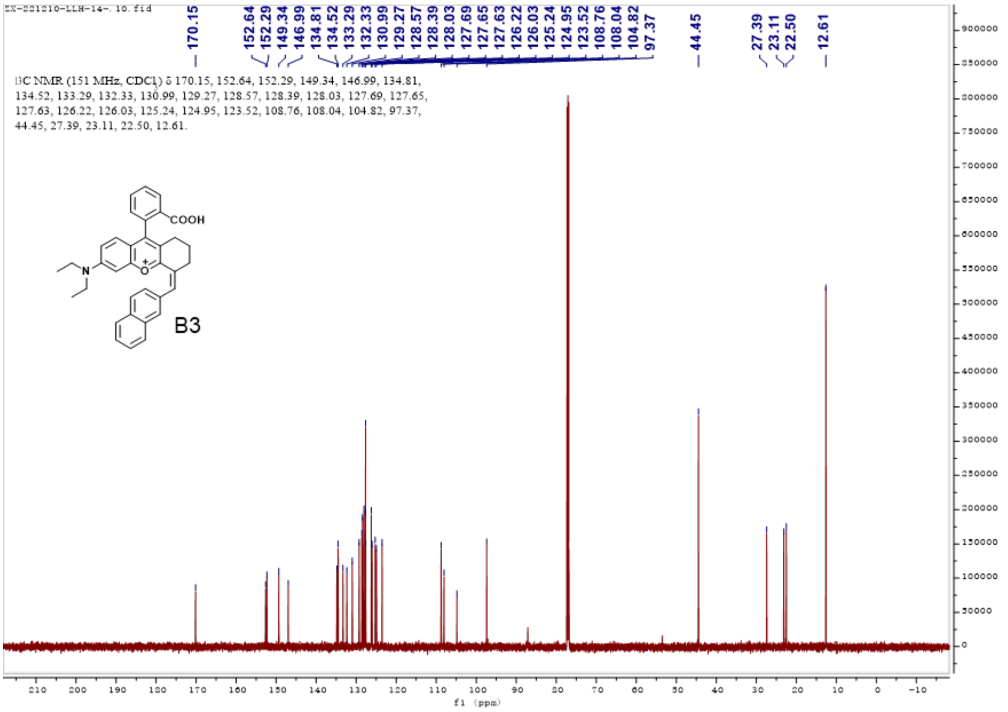


$^{13}$C NMR Spectra of B3 ($CDCl_3$)

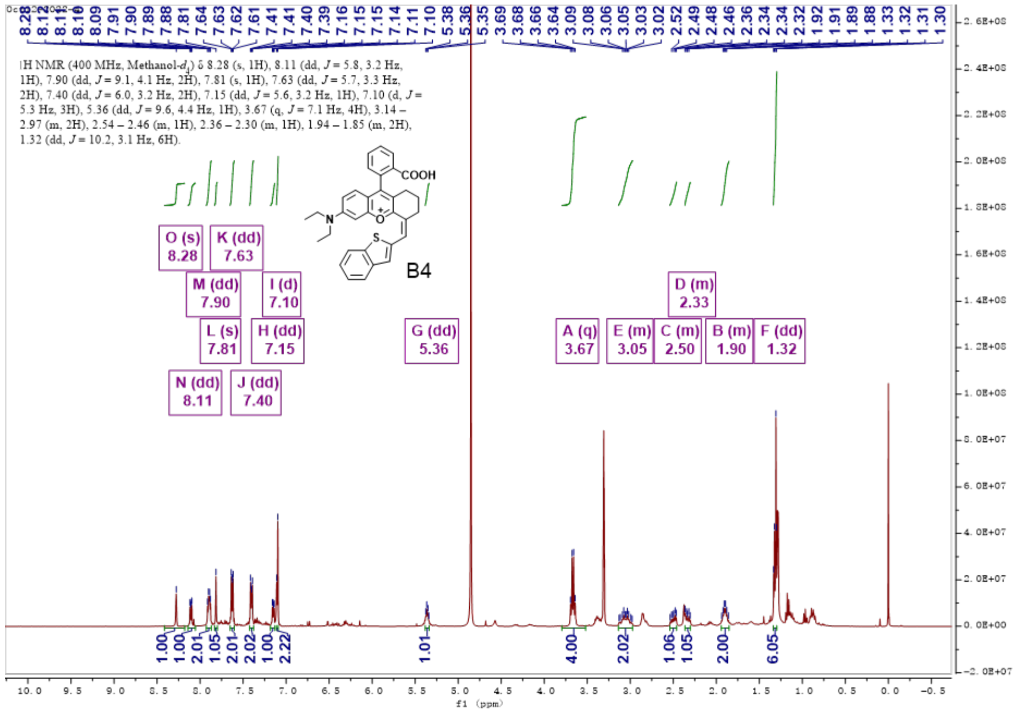


$^{1}$H NMR Spectra of B4 (MeOD)

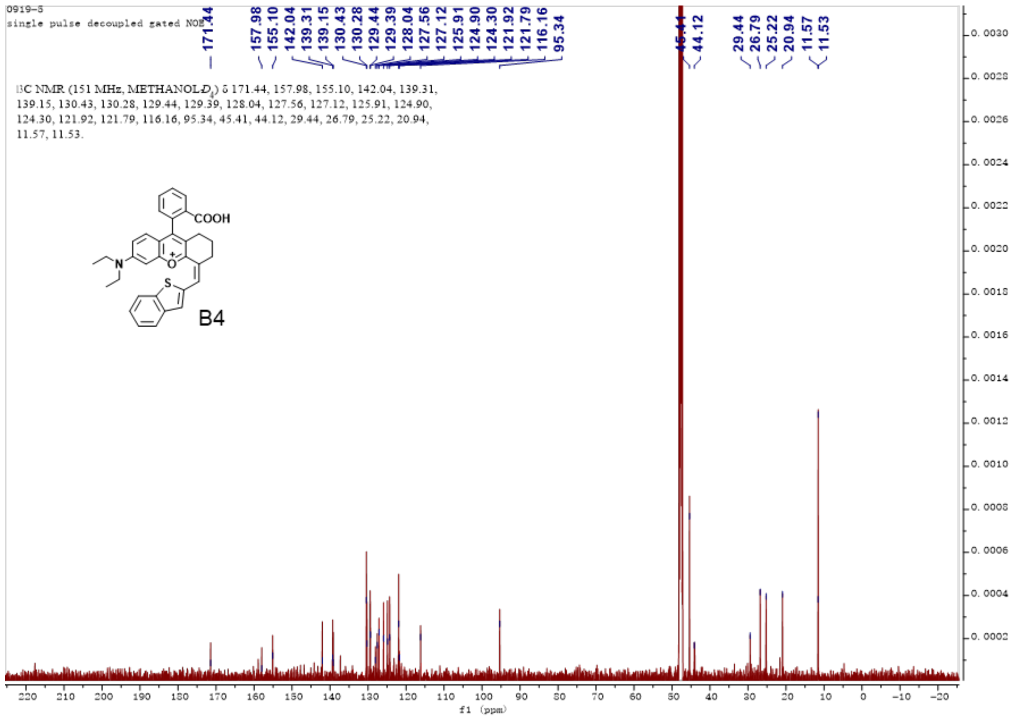


$^{13}$C NMR Spectra of B4 (MeOD)

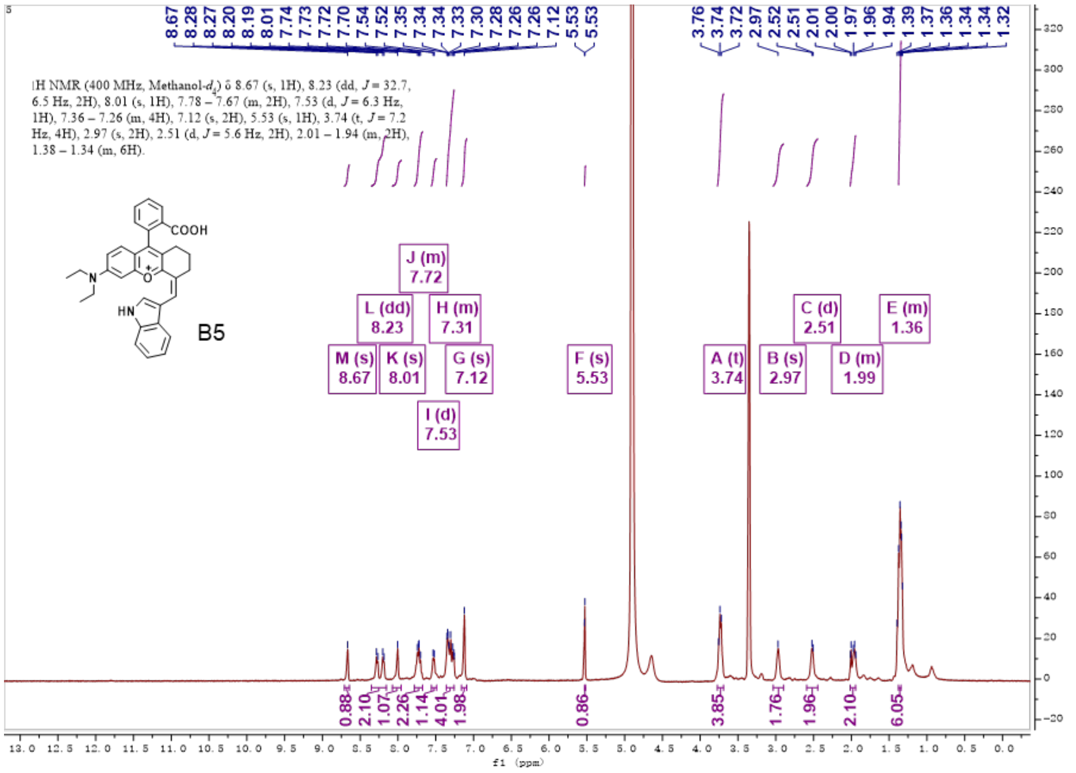


$^1$H NMR Spectra of B5 (MeOD)

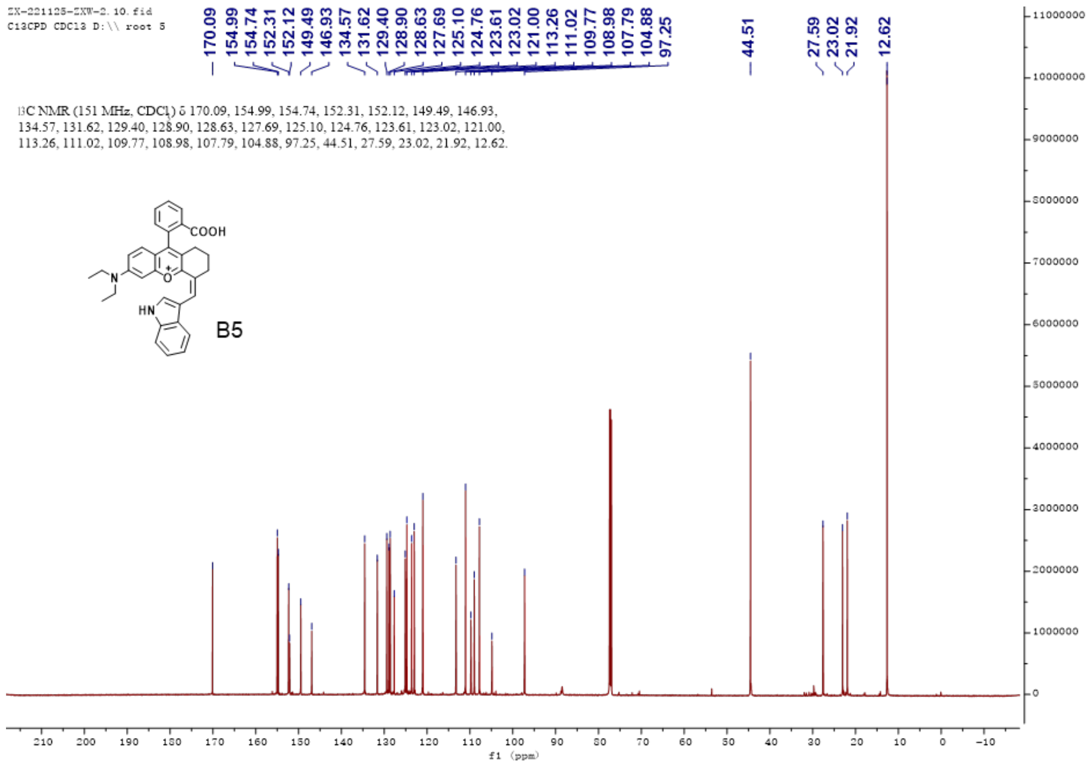


$^{13}$C NMR Spectra of B5 (MeOD)

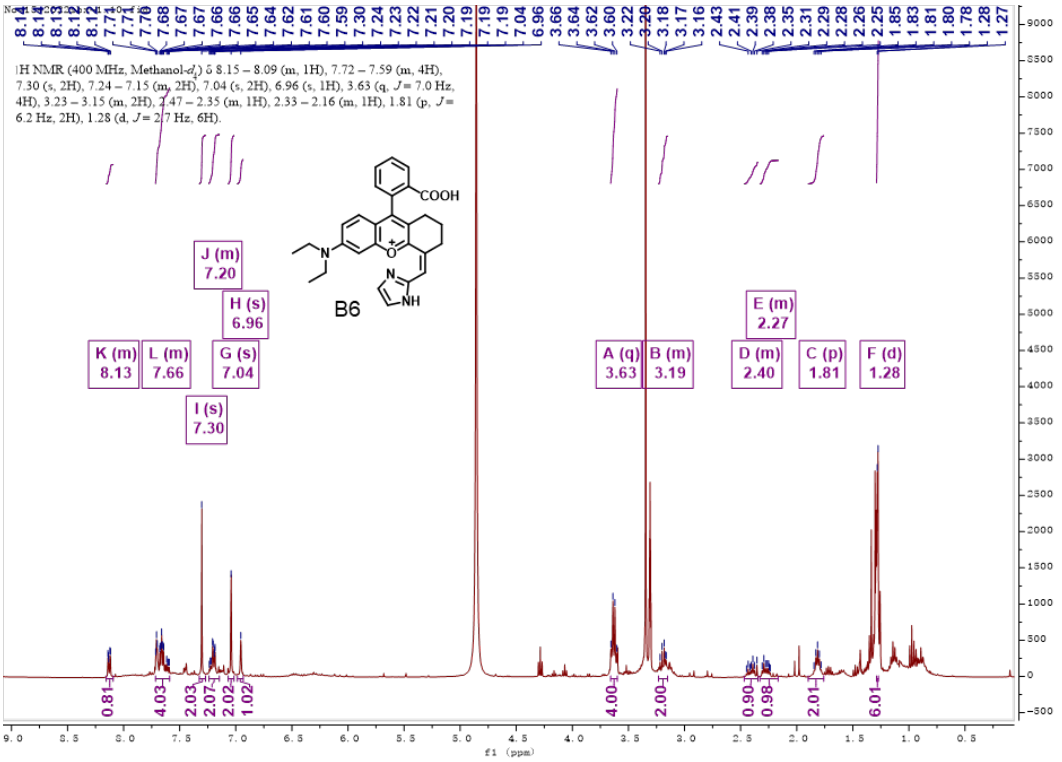


$^{1}$H NMR Spectra of B6 (MeOD)

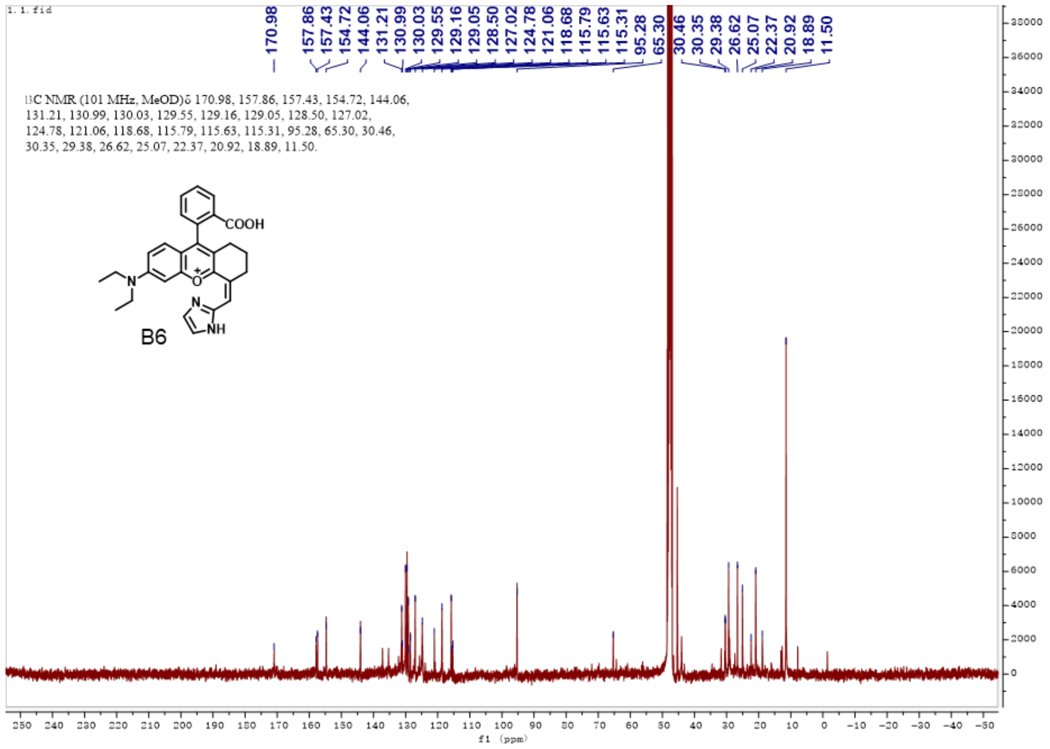


$^{13}$C NMR Spectra of B6 (MeOD)

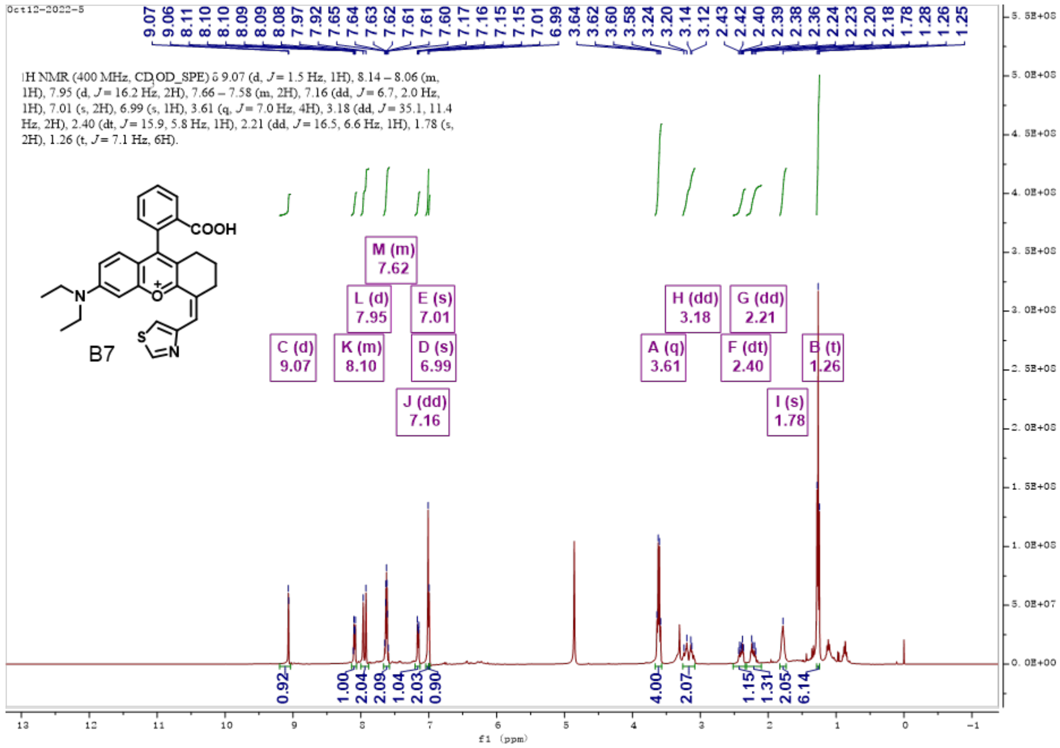


$^{1}$H NMR Spectra of B7 ($CD_3OD$)

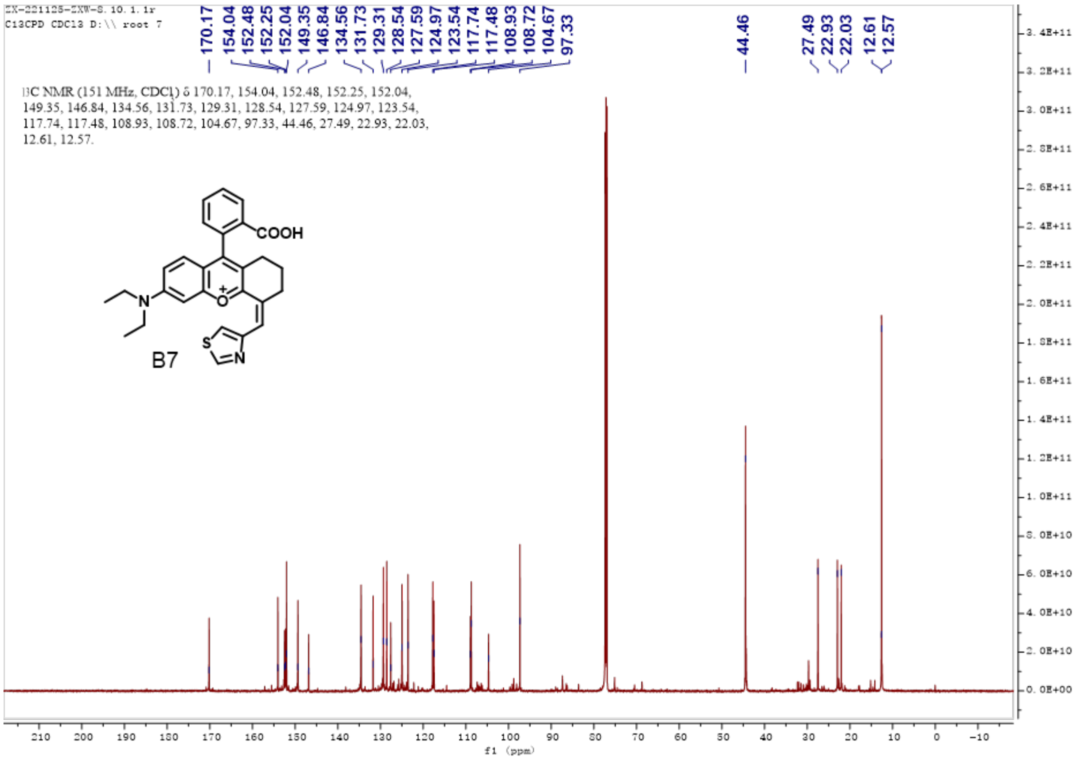


$^{13}$C NMR Spectra of B7 ($CDCl_3$)

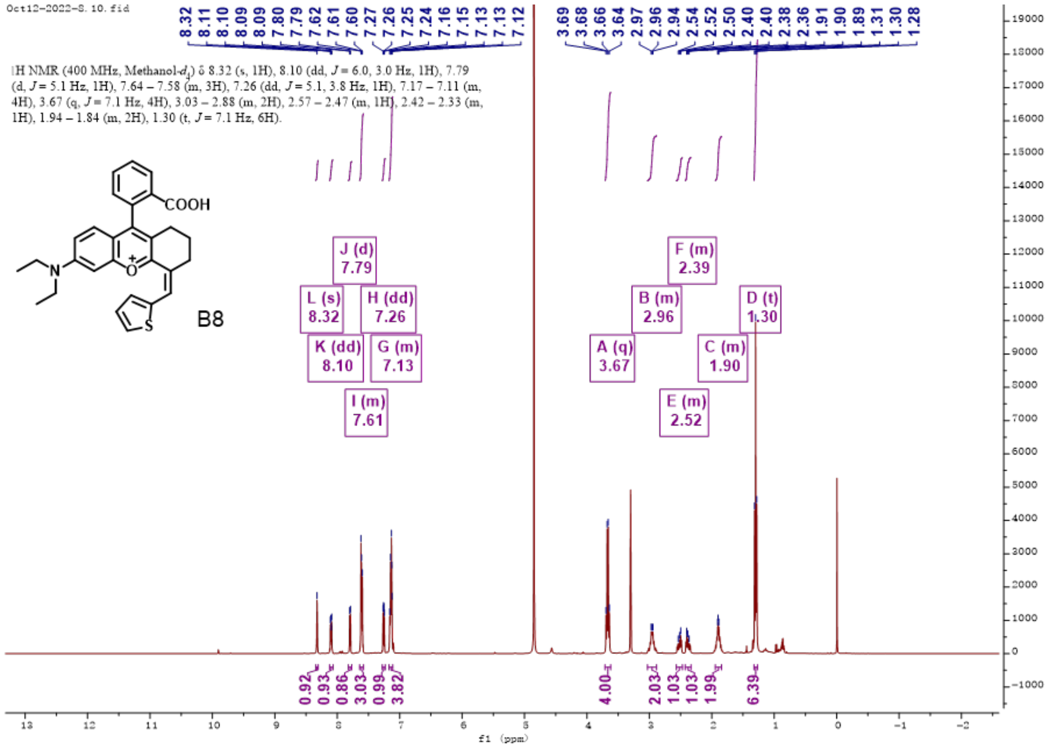


$^{1}$H NMR Spectra of B8 (MeOD)

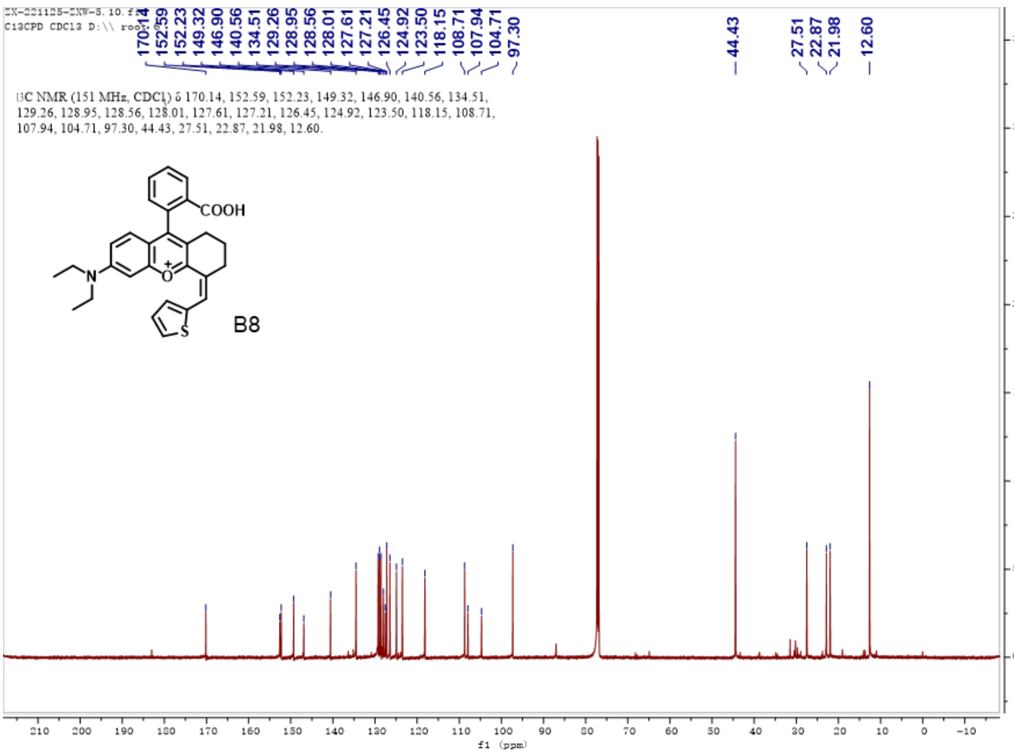


$^{13}$C NMR Spectra of B8 ($CDCl_3$)

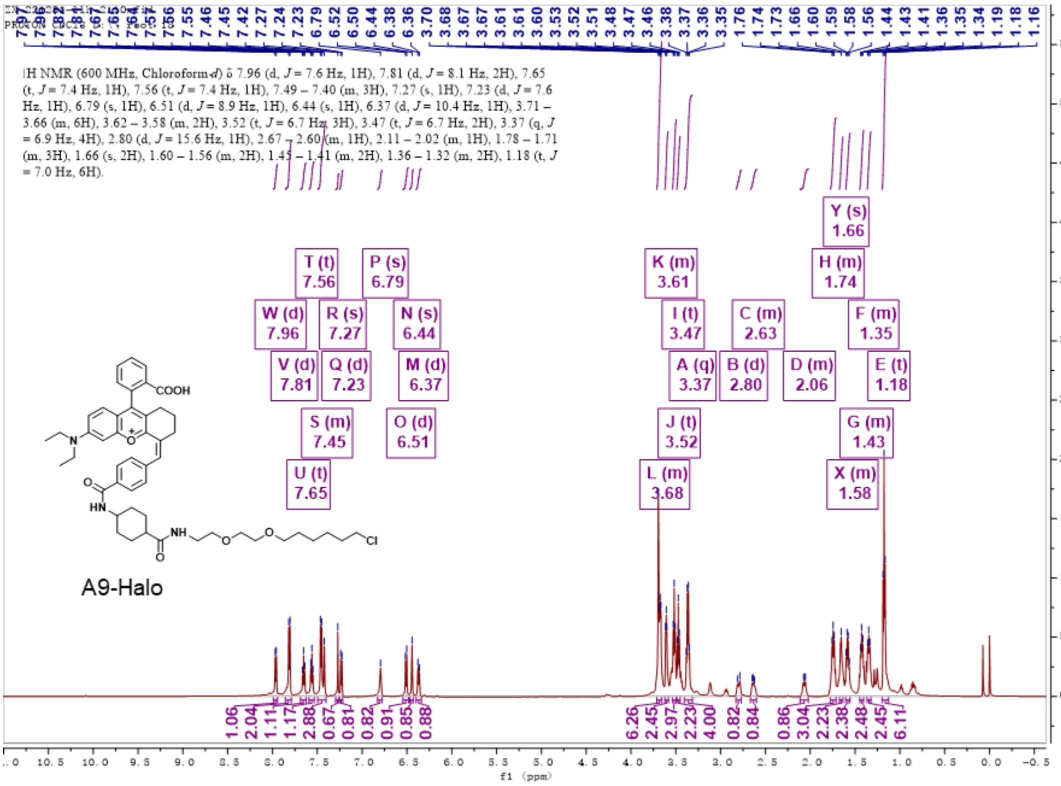


$^{1}$H NMR Spectra of A9-Halo ($CDCl_3$)

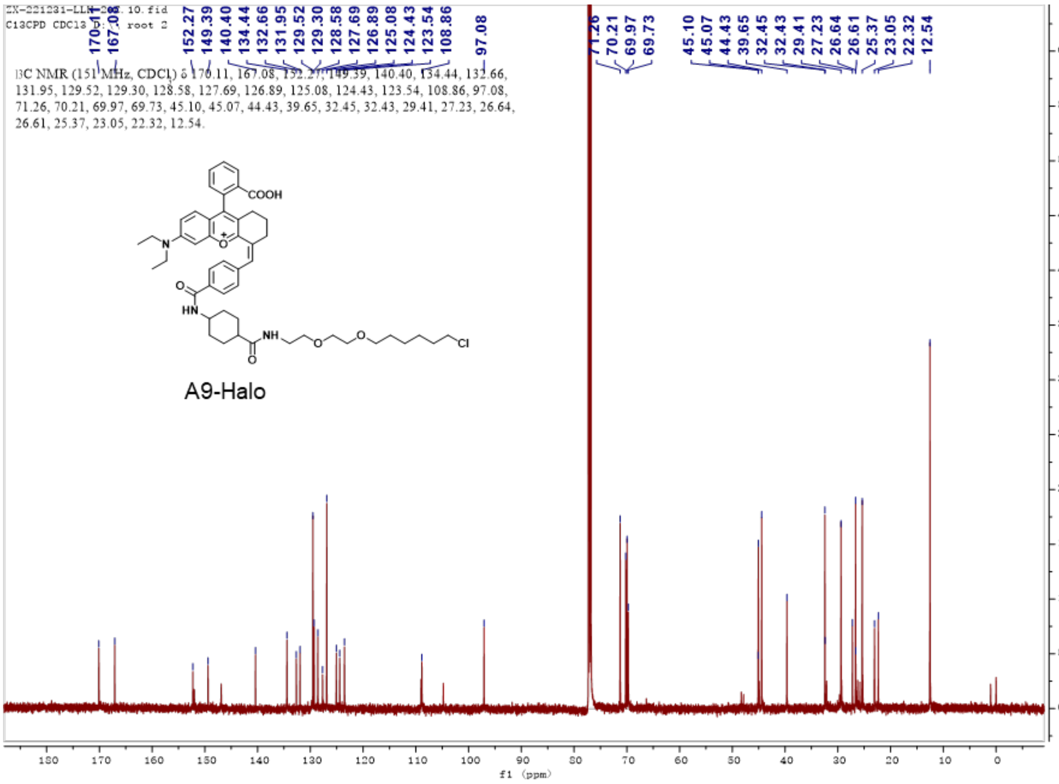


$^{13}$C NMR Spectra of A9-Halo ($CDCl_3$)

## 6. References

[1] Y. Liu, C.H. Wolstenholme, G.C. Carter, H. Liu, H. Hu, L.S. Grainger, K. Miao, M. Fares, C.A. Hoelzel, H.P. Yennawar, G. Ning, M. Du, L. Bai, X. Li, X. Zhang, Modulation of Fluorescent Protein Chromophores To Detect Protein Aggregation with Turn-On Fluorescence, Journal of the American Chemical Society, 140 (2018) 7381-7384.

[2] K. H. Jung, S. F. Kim, Y. Liu and X. Zhang, Chembiochem, 2019, 20, 1078-1087

[3]. Yuan, L.; Lin, W.; Yang, Y.; Chen, H., A Unique Class of Near-Infrared Functional Fluorescent Dyes with Carboxylic-Acid-Modulated Fluorescence ON/OFF Switching: Rational Design, Synthesis, Optical Properties, Theoretical Calculations, and Applications for Fluorescence Imaging in Living Animals. Journal of the American Chemical Society 2012, 134 (2), 1200-1211.
